# The effects of resistance training with blood flow restriction on muscle strength, muscle hypertrophy and functionality in patients with osteoarthritis and rheumatoid arthritis: A systematic review with meta-analysis

**DOI:** 10.1371/journal.pone.0259574

**Published:** 2021-11-10

**Authors:** Leonardo Peterson dos Santos, Rafaela Cavalheiro do Espírito Santo, Thiago Rozales Ramis, Juliana Katarina Schoer Portes, Rafael Mendonça da Silva Chakr, Ricardo Machado Xavier

**Affiliations:** 1 Autoimmune Diseases Laboratory, Universidade Federal do Rio Grande do Sul (UFRGS), Porto Alegre, Rio Grande do Sul, Brazil; 2 Division of Rheumatology, Hospital de Clínicas de Porto Alegre (HCPA), Porto Alegre, Rio Grande do Sul, Brazil; 3 Medical School, Universidade Federal do Rio Grande do Sul (UFRGS), Porto Alegre, Rio Grande do Sul, Brazil; 4 Exercise Research Laboratory (LAPEX), Universidade Federal do Rio Grande do Sul (UFRGS), Porto Alegre, Rio Grande do Sul, Brazil; Universita degli Studi di Milano, ITALY

## Abstract

**Introduction:**

Rheumatoid arthritis(RA) and osteoarthritis(OA) patients showed systemic manifestations that may lead to a reduction in muscle strength, muscle mass and, consequently, to a reduction in functionality. On the other hand, moderate intensity resistance training(MIRT) and high intensity resistance training(HIRT) are able to improve muscle strength and muscle mass in RA and OA without affecting the disease course. However, due to the articular manifestations caused by these diseases, these patients may present intolerance to MIRT or HIRT. Thus, the low intensity resistance training combined with blood flow restriction(LIRTBFR) may be a new training strategy for these populations.

**Objective:**

To perform a systematic review with meta-analysis to verify the effects of LIRTBFR on muscle strength, muscle mass and functionality in RA and OA patients.

**Materials and methods:**

A systematic review with meta-analysis of randomized clinical trials(RCTs), published in English, between 1957–2021, was conducted using MEDLINE(PubMed), Embase and Cochrane Library. The methodological quality was assessed using Physiotherapy Evidence Database scale. The risk of bias was assessed using RoB2.0. Mean difference(MD) or standardized mean difference(SMD) and 95% confidence intervals(CI) were pooled using a random-effects model. A P<0.05 was considered statistically significant.

**Results:**

Five RCTs were included. We found no significant differences in the effects between LIRTBFR, MIRT and HIRT on muscle strength, which was assessed by tests of quadriceps strength(SMD = -0.01[-0.57, 0.54], *P =* 0.96; I² = 58%) and functionality measured by tests with patterns similar to walking(SMD = -0.04[-0.39, 0.31], *P =* 0.82; I² = 0%). Compared to HIRT, muscle mass gain after LIRTBFR was reported to be similar. When comparing LIRTBFR with low intensity resistance training without blood flow restriction(LIRT), the effect LIRTBFR was reported to be higher on muscle strength, which was evaluated by the knee extension test.

**Conclusion:**

LIRTBFR appears to be a promising strategy for gains in muscle strength, muscle mass and functionality in a predominant sample of RA and OA women.

## Introduction

Rheumatoid arthritis (RA) and osteoarthritis (OA) are examples of chronic arthropathies. RA is an autoimmune, chronic and progressive disease with systemic inflammation that affects mainly large joints. RA patients often have changes in body composition [1–3], such as decrease of fat free mass, specially appendicular skeletal mass, with stable or increase of fat mass [4, 5]. In addition, RA patients showed a reduction of muscle strength [6], which, along with the changes in body composition, contribute to the increase of physical disability [4, 5, 7, 8], also increasing the risk of comorbidities and mortality [9–11].

On the other hand, OA is a chronic, inflammatory and highly prevalent joint disease [12–15] with cartilage degeneration, mild to moderate inflammation of the synovial lining and radiological changes [12–16]. Although OA shows local inflammation, the reduced strength and muscle mass also are observed in OA patients, as well as in RA patients. Due to theses alterations, OA patients may show reduction of functionality [12, 15, 17, 18], increase in risk of falls and fractures [18].

Resistance training is an important strategy to increase muscle strength and muscle mass in healthy people [19]. According to the American College of Sports Medicine (ACSM), moderate intensity resistance training (MIRT) with 60–70% of 1 maximum repetition (1RM) is sufficient to increase muscle strength and muscle mass in individuals from the beginner to the intermediate level in training [20]. On the other hand, high intensity resistance training (HIRT), which is performed with intensities between 70–85% 1RM, is also able to create such benefits, this protocol being used more frequently [21]. In RA and OA patients, studies demonstrated that both MIRT [22] and HIRT [23–25] are capable of promoting increases on muscle strength, muscle mass and functionality. However, due to the articular and extra-articular manifestations caused by RA and OA and to the high overload necessary on HIRT to obtain such benefits, these patients may present intolerance to resistance training [26–29].

Thus, low intensity resistance training combined with blood flow restriction (LIRTBFR) may be a new strategy for these populations. LIRTBFR consists of training performed with low intensities, between 15%-30% 1RM, with blood flow restriction conducted by a cuff located in the proximal region of the lower or upper limb [30–33]. According to the literature, LIRTBFR is able to provide gains in muscle strength and muscle mass, as well as HIRT [30, 34–37], being extremely important for populations that are not capable to tolerate HIRT.

Although LIRTBFR has been studied for a long time and some meta-analysis have already investigated this protocol based on rehabilitation [38, 39]. There are still few studies in the literature that have evaluated LIRTBFR in chronic arthropathies patients. Thus, there is still no consensus on the effect of LIRTBFR on muscle strength, muscle mass and functionality in these populations. Therefore, this systematic review aims to summarize the current evidence on the effects of LIRTBFR on muscle strength, muscle mass and functionality in RA and OA patients (chronic arthropathies).

## Materials and methods

We conducted this systematic review with meta-analysis in accordance with PRISMA [40] (see S1 File) guidelines after registering the protocol with PROSPERO platform (CRD42020200261).

### PICOS/PECOS format

This systematic review with meta-analysis was based on a focused question described in a PICO/PECO format [41]. We established: Patient/Problem/Population = Rheumatoid Arthritis and Osteoarthritis patients, Intervention/Exposure = low intensity resistance training combined with blood flow restriction, Comparison = Moderate and high intensity training or training without blood flow restriction, Outcomes = muscle strength, muscle mass and functionality and Study = randomized clinical trials.

#### Data sources

The electronic databases used were: Cochrane Library, PubMed and Embase in July/2021. We used a comprehensive search strategy tailored to each database. We contacted the authors, when necessary, for more information on the statistical methodology of the articles chosen as a reference.

#### Search terms

Keywords and medical subject headings (MeSH) for the terms “Osteoarthritis”, “rheumatoid arthritis”, “kaatsu”, “blood flow restriction”, “training” and related terms were selected. The term OR was used for Union of MeSH terms and “entry terms”, and the term AND was used to attach the terms. Complete search is available below:

((Osteoarthritis[MeSH] OR osteoarthritis[All Fields] OR "Arthritis, Degenerative"[All Fields] OR "degenerative arthritis" OR Arthroses[All Fields] OR Arthrosis[All Fields] OR Osteoarthrosis[All Fields] OR "Osteoarthrosis Deformans"[All Fields]) OR (Arthritis, Rheumatoid[MeSH] OR "arthritis, rheumatoid"[All Fields] OR "rheumatoid arthritis"[All Fields])) AND (((resistance[All Fields] OR strength[All Fields] OR resistance[All Fields] OR "high intensity"[All Fields] OR exercise[All Fields]) AND (training[All Fields] OR exercise[All Fields]))) AND ((kaatsu[All Fields] OR "blood flow restriction"[All Fields] OR "vascular occlusion"[All Fields] OR "blood flow occlusion"[All Fields] OR ischemic[All Fields] OR "low load resistance"[All Fields] OR "partial vascular"[All Fields] OR "restriction blood flow"[All Fields]) AND (train[All Fields] OR training[All Fields] OR strength[All Fields] OR exercise[All Fields]).

#### Inclusion/exclusion criteria

We included: randomized clinical trials with the intervention of low intensity resistance training combined with blood flow restriction (20–50% 1RM) and moderate to high intensity exercise (> 60% 1RM) or low intensity exercise without blood flow restriction (20–50% 1RM), training with 2 weeks of intervention or more, patients diagnosed with RA and OA, and articles which were written in English language. No restriction on publication date was imposed. In addition, included studies were required to report at least one of the following assessments: maximum muscle strength = isometric dynamometer, isokinetic dynamometer and RM tests (specific tests for quadriceps strength); muscle mass = computed tomography or magnetic resonance imaging (muscle quantity); functionality objectively = Time Up and Go test and 400 Meters for Walking (tests with patterns similar to walking).

We excluded: articles reporting data from patients < 18 years old, meta-analysis articles, manuscripts written in a language other than English, experiments performed in animal studies or studies in patients without a diagnosis of RA and OA.

### Study selection and data extraction

Title, abstract, and full-text screening were performed in pairs by two independent reviewers (Santos, LP and Portes, JKS). The reviewers extracted the data from the studies independently, using a pre-established data sheet, which is available in S2 File. All data from the study were screened using a bibliographic management program (Mendeley®, version1.17.9). Disagreements about data abstraction were resolved by discussion between the two reviewers. If no agreement could be reached, a third and fourth reviewers (Santo, RCE, and Ramis, T) provided the final decision. The information extracted during the data abstraction, included authors’ names, date of publication, Journal of publication, number of participants in the study, the age group of the population, type of population, protocol training, training time, type of occlusion, results obtained for muscle strength, muscle mass and physical function. After agreement between the two evaluators and inclusion of the articles in our systematic review with meta-analysis (n = 5), data were extracted from each study. When available, data were extracted in the form of delta mean (mean_change_), delta standard deviation (SD_change_), and sample size of the studies to perform the meta-analysis. When data were not available in the expected format, we contacted the respective authors requesting information about missing data. If after our contact the authors did not return or if the data provided by them were not completely clear, means and SD from the figures provided in the article were extrapolated by the Image J program. On the other hand, when the article reported baseline and post-intervention outcomes, however, without mean_change_ and SD_change_, we used the equation (Delta mean = post-training mean–baseline mean) to calculate the delta value. To calculate the SD_change_, we used the correlation estimation formula, provided by the Cochrane handbook [42], using data from the study by Bryk et al. [25], who provided their baseline, post-intervention and delta data, which are required for this calculation. The following formula was used to estimate the SD_change_ of other articles based on the correlation found. The equations are available below [42]:

$${\text{Corr}}_{\text{E}}\text{=}\frac{{\text{SD}}_{\text{E},\text{baseline}}^{2}+{\text{SD}}_{\text{E},\text{final}}^{2}-{\text{SD}}_{\text{E},\text{change}}^{2}}{2\;\text{x}\;{\text{SD}}_{\text{E},\text{baseline}}\;\text{x}\;{\text{SD}}_{\text{E},\text{final}}}$$ (1)

$${\text{SD}}_{\text{E},\text{change}}=\sqrt{{\text{SD}}_{\text{E},\text{baseline}}^{2}+{\text{SD}}_{\text{E},\text{final}}^{2}-(2\;\text{x}\;\text{Corr}\;\text{x}\;{\text{SD}}_{\text{E},\text{baseline}}\;\text{x}\;}{\text{SD}}_{\text{E},\text{final}}$$ (2)

Where Corr_E_ is correlation coefficient in the experimental group, SD_E,baseline_ is baseline standard deviation in the experimental group, SD_E,final_ is final standard deviation in the experimental group and SD_E,change_ is standard deviation of the changes in the experimental group. When data were presented by interquartile range (IQR), it was decided to transform these data in order to standardize the results of all studies in mean_change_ and SD_change_. The equation used to calculate the mean_change_ is available below [43]:

$$\bar{\text{x}}\approx \frac{{\text{q}}_{1}+\text{m}+{\text{q}}_{3}}{3}$$ (3)

Where q_1_ is the first quartile, m is the median and q_3_ is the third quartile. Finally, to find the SD_change_ presented by IQR, we use the calculation available below [43]:

$$\text{S}\approx \frac{{\text{q}}_{3}-{\text{q}}_{1}}{1.35}$$ (4)

### Methodological quality assessment

The Physiotherapy Evidence Database (PEDro) scale was used to assess methodological quality [44]. PEDro scale was performed in pairs by two independent reviewers (Santo, RCE and Ramis, T). Disagreements about methodological quality were resolved by a third reviewer (Portes, JKS). PEDro scale is composed of: external validity (item 1), internal validity (items 2–9), and statistical reports (items 10–11) [44]. The maximum possible score was 10 points. Authors suggest that scores <4 points are considered "bad", 4–5 are considered "regular", 6–8 are considered "good" and 9–10 are considered "excellent" by PEDro scale [45, 46]. Studies were included independently of the methodological quality calculated.

### Risk of bias

The risk of bias of the studies was assessed using the risk of bias tool 2.0 (RoB2) from Cochrane to randomized clinical trials [47]. RoB2 was also performed in pairs by two independent reviewers (Santo, RCE and Ramis, T). If there was disagreement between the two evaluators about the risk of bias analyzed, a third reviewer performed the consensus (Portes, JKS). The evaluators examined the randomization process, deviations from intended interventions, missing outcome data, measurement of the outcome, and selection of the reported results. Thus, the studies were classified into low, moderate, or high risk of bias.

### Statistical analysis

The meta-analysis was conducted using mean_change_ and SD_change_ from each study. All outcome measures were continuous variables. Two meta-analyses, representing the effects of interventions, were performed: the random-effects model with the mean difference (MD) or standardized mean difference (SMD). MD was performed when studies reported outcomes using the same assessment scale or assessment instrument. On the other hand, the SMD was performed when the same outcomes between studies are evaluated, however, analyzed by different scales or instruments [42]. The calculation of SMD is represented by dividing the difference in mean outcome between groups by the standard deviation of the result within the groups. The formula between groups within each study used is available below [48]:

$${\text{S}}_{\text{within}}=\sqrt{\frac{(n_{1}-1)S_{1}^{2}+(n_{2}-1)S_{2}^{2}}{n_{1}+n_{2}-2}}$$ (5)

n_1_ and n_2_ are the sample sizes in the two groups and S_1_ and S_2_ are the standard deviations in the two groups. Thus, by combining both SD estimates, greater precision in estimating their common value is achieved. The square root of variation (VD) of SMD is determined by the standard error (SE). The VD was calculated using the formula below [48]:

$${\text{V}}_{\text{d}}=\frac{n_{1}+n_{2}}{n_{1}n_{2}}+\frac{SMD^{2}}{2(n_{1}+n_{2})}$$ (6)

The 95% confidence intervals (CI) were used and the heterogeneity of the studies included in the meta-analysis was assessed using the inconsistency test (I²). We considered low, moderate and high inconsistence the approximated values to 25%, 50% and 75%, respectively [42, 49]. The software used for statistical analysis was RevMan (Review Manager 5.4.1, The Cochrane Collaboration, 2020), and we considered significant statistically when P < 0.05.

## Results

### Search strategy

**Fig 1:**
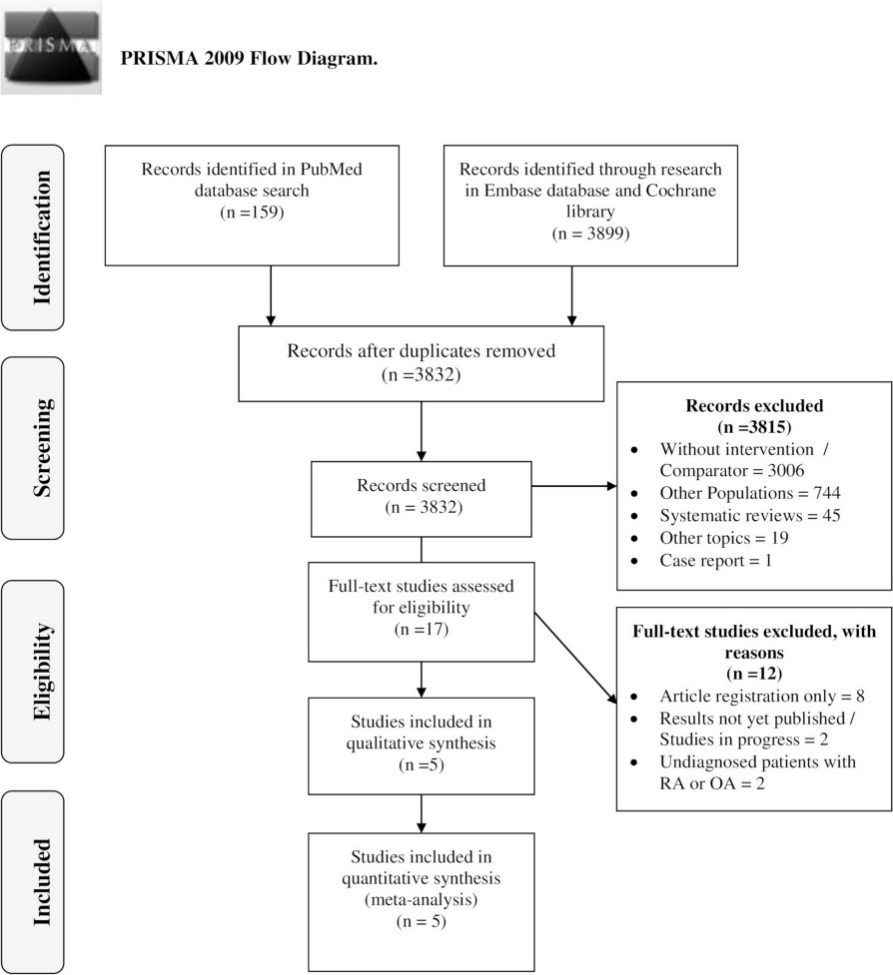
PRISMA. Flow diagram of search results and study selection.

We identified 3832 studies (226 duplicate publication) based on our search strategy (see Fig 1). First, the title and abstract of the 3832 studies included were screened. After this process, the remaining articles were included or excluded according to the analysis of the full text. In this process, only five studies, between 1957–2021, were included in the review and incorporated into the meta-analysis.

### Characteristics of studies

From the five studies included, three were performed in knee OA [22, 24, 25] patients and two were performed in RA [23, 50] patients. Four studies included evaluation of women only [23–25, 50], and one study assessed both genders (gender, female: LIRTBFR, 62.5%; MIRT, 78.9%) [22]. The mean age between all studies was 59.88 ± 5.73 years old.

In regarding to disease activity, Rodrigues et al. [23] demonstrated that Disease Activity Score 28 (DAS-28) was similar between HIRT (2.76 ± 0.79), LIRTBFR (2.72 ± 1.0) and untrained groups (2.66 ± 0.8) of RA patients (p = 0.819) [23] at baseline. Jønsson et al. [50] demonstrated that DAS-28 was also similar between LIRTBFR (2.43 ± 0.67) and low intensity resistance training without blood flow restriction (LIRT) (2.53 ± 1.33). On the other hand, Harper et al. [22], Bryk et al. [25] and Ferraz et al. [24] did not assess disease activity. However, both studies included OA patients using the Kellgren and Lawrence method disease severity classification. The reported score was 2–3 in the studies by Bryk et al. [25] and Ferraz et al. [24], and ≥ 2 in the study by Harper et al. [22]. Harper et al. [22] showed means and SD of 2.9 ± 0.8 for MIRT and 2.8 ± 0.8 for LIRTBFR at baseline.

### Characteristics of resistance training

Most studies had a frequency of training of three times per week [22, 25, 50] and total duration time of twelve weeks of training [22–24]. The intensities used for LIRTBFR [22–25] and LIRT were 20–30% 1RM in most studies. Jønsson et al. [50], on the other hand, used intensities between 30–50% 1RM for the LIRTBFR and LIRT protocol. MIRT and HIRT groups showed an intensity range of 60–80% 1RM [22–25]. In the training protocol, the most used exercises were leg press [22–24, 50] and knee extension [22–25, 50], with three to five sets for each exercise. Regarding to load adjustment during the training protocol, one study [25] adjusted the load of training weekly, one study [22] adjusted the load of training each three weeks and two studies [23, 24] adjusted the load of training each four weeks. On the other hand, one study [50] did not adjust the load of training during the training protocol. Characteristic of the included studies are described in Table 1. Details on the training methodology of the included studies are described in Table 2.

**Table 1:** Characteristic of the included studies.

Authors	Year	Disease	Sample Size	Age (LIRTBFR)	Age (MIRT/HIRT)	Age (LIRT)	Gender	Occlusion Location	Occlusion pressure	Cuff size
Rodrigues et al. [23]	2020	RA	n = 48	59.6 ± 3.9	58.0 ± 6.6	58.1 ± 5.9 (Untrained group)	Women	Cuff placed at the inguinal fold	108.9±14.6 mmHg	175 mm × 920 mm
Bryk et al. [25]	2016	OA	n = 34	62.3 ± 7.0	60.4 ± 6.7	-	Women	Cuff applied to the upper third of the thigh	200 mmHg	**NR:** Not reported
Jønsson et al. [50]	2020	RA	n = 17	57.33 ± 5.19	-	45.67 ± 17.04	Women	Cuffs were placed horizontally, close to the groin	155 ± 6.1 mmHg	7 cm wide occlusion cuff
Ferraz et al. [24]	2018	OA	n = 48	60.3 ± 3.0	59.9 ± 4.0	60.7 ± 4.0	Women	Air cuff was attached to the patients thigh (Inguinal fold region)	97.4±7.6 mmHg	175 mm x 920 mm
Harper et al. [22]	2019	OA	n = 27	67.2 ± 5.2	69.1 ± 7.1	-	Women and men	External compression applied to the proximal thigh of both legs.	Individual Pressure trough equation §	**NR:** Not reported

LIRTBFR = low intensity resistance training combined with blood flow restriction; MIRT = Moderate intensity resistance training; HIRT = High intensity resistance training; LIRT = low intensity resistance training without blood flow restriction. RA = Rheumatoid arthritis; OA = Osteoarthritis. mmHg = millimeters of mercury; mm = millimeters; § = [Pressure mmHg = 0.5 (Resting systolic blood pressure) + 2(thigh circumference) + 5]. NR = Not reported by study. Values are reported as Mean ± SD.

**Table 2:** Training methodology of the included studies.

Authors	Training period	Training frequency (LIRTBFR)	Training frequency (MIRT/HIRT)	Training frequency (LIRT)	Training intensity (LIRTBFR)	Training intensity (MIRT/HIRT)	Training intensity (LIRT)	Training Protocol (LIRTBFR)	Training Protocol (MIRT/HIRT)	Training Protocol (LIRT)
Rodrigues et al. [23]	12 week	2 time/week	2 time/week	-	20%-30% 1RM	70% 1RM	Untrained group	Bilateral leg press AND knee extension exercises: **1-4/week:** 4 sets of 15 repetitions; **5/week until the end of the protocol:** 5 sets of 15 repetitions for each exercise. (1-minute rest)	Bilateral leg press AND knee extension exercises: **1-4/week:** 4 sets of 10 repetitions; **5/week until the end of the protocol:** 5 sets of 10 repetitions for each exercise. (1-minute rest)	Instructed to maintain their habitual daily living activities.
Bryk et al. [25]	6 week	3 time/week	3 time/week	-	30% 1RM	70% 1RM	-	**(A)** Hamstrings stretching; Isometric bridge; Sensory-motor training; **(B)** Calf rise; Calm exercise; Knee extension.	**(A)** Hamstrings stretching; Isometric bridge; Sensory-motor training; **(B)** Calf rise; Calm exercise; Knee extension.	-
								**(A)** 3 sets of 30 seconds; **(B)** 3 sets of 10 repetitions. (NR the time of rest)	**(A)** 3 sets of 30 seconds; **(B)** 3 sets of 10 repetitions. (NR the time of rest)	
Jønsson et al. [50]	4 week	3 time/week	-	3 time/week	30–50% 1RM	-	30–50% 1RM	Leg extension and prone leg curl machine (30–40% 1RM) + Leg press machine (50% 1RM). 3 sets of each exercise to volitional failure. (45seconds of rest)	**-**	Leg extension and prone leg curl machine (30–40% 1RM) + Leg press machine (50% 1RM). 3 sets of each exercise to volitional failure. (45seconds of rest)
Ferraz et al. [24]	12 week	2 time/week	2 time/week	2 time/week	20%-30% 1RM	80% 1RM	20–30% 1RM	Bilateral leg press and knee extension: **1-4/week:** 4 sets of 15 repetitions; **5/week until the end of the protocol:** 5 sets of 15 repetitions. (1-minute rest)	Bilateral leg press and knee extension: **1-4/week:** 4 sets of 10 repetitions; **5/week until the end of the protocol:** 5 sets of 10 repetitions. (1-minute rest)	Bilateral leg press and knee extension: **1-4/week:** 4 sets of 15 repetitions; **5/week until the end of the protocol:** 5 sets of 15 repetitions. (1-minute rest)
Harper et al. [22]	12 week	3 time/week	3 time/week	-	20% 1RM	60% 1RM	-	**Limb exercises**(leg press, leg extension, calf flexion, leg curl)	**Limb exercises**(leg, press, leg extension, calf flexion, leg curl)	-
								**NR: Not reported**	**NR: Not reported**	
								**Sets x Reps x Res**t	**Sets x Reps x Rest**	

LIRTBFR = low intensity resistance training combined with blood flow restriction; MIRT = Moderate intensity resistance training; HIRT = High intensity resistance training; LIRT = low intensity resistance training without blood flow restriction; RM = repetition maximum; Reps = repetitions. NR = Not reported by study.

### Methods of assessment of the muscle strength, muscle mass and functionality

Several protocols were used for muscle strength assessment. The leg press 1RM test and knee extension 1RM test were evaluated in two studies [23, 24]. Other methods of measures for muscle strength were used: isometric voluntary contraction [25], isokinetic knee extensor [22], leg press 3RM test and knee extension 3RM test [50].

In regarding to muscle mass assessment, two studies evaluated it by Computed Tomography Imaging [23, 24]. Regarding to functional assessments, the tests used were: Timed stands test (TST) [23, 24], Timed up and go test (TUG) [23–25], Walking speed of 400 meters [22], Health Assessment Questionnaire (HAQ) [23], Western Ontario and McMaster Universities questionnaire (WOMAC) [24], Lequesne questionnaire [25], and Late Life Function and Disability Instrument (LLFDI) [22].

In this sense, in studies where multiple modes of muscle strength and functionality measurements were reported, the test modality with the highest test-retest reliability was included, prioritized in the following order: Muscle strength = (1) Isometric dynamometer, (2) Isokinetic dynamometer and (3) RM-test (knee extension > leg press). The choice to prioritize the knee extensor test over the leg press was due to the impossibility of restricting blood flow to the gluteal muscles involved in this exercise [51]. Objective functionality = (1) TUG test and (2) Walking speed of 400 meters. Such prioritization was chosen because movement patterns were similar between tests.

### Synthesis of results

The changes in mean deltas of muscle strength, muscle mass and functionality among the included studies are presented in Table 3.

**Table 3:** Description of changes in mean deltas in muscle strength, muscle mass and functionality among included studies.

First author	Change in mean deltas in LIRTBFR group	Change in mean deltas in MIRT/HIRT group	Change in mean deltas in LIRT group
Rodrigues et al. [23]	*Muscle strength*:	*Muscle strength*:	Untrained group
	**Knee extension-1RM (kg): 6.04 ± 4.93** ↑	**Knee extension-1RM (kg): 8.43 ± 3.88** ↑	
	Leg press-1RM (kg): 25.44 ± 14.5 ↑	Leg press-1RM (kg): 27.58 ± 19.66 ↑	
	*Muscle mass*:	*Muscle mass*:	
	**CT (mm²): 414.5 ± 218.72** ↑	**CT (mm²): 480.2 ± 230.14** ↑	
	*Functionality*:	*Functionality*:	
	**TUG (s): 0.5** ± **0.67** ↓	**TUG (s): 0.62 ± 0.46** ↓	
	TST (repetition): 1.63 ± 1.13 ↑	TST (repetition): 1.95 ± 1.26 ↑	
	HAQ (score): 0.2 ± 0.41 ↓	HAQ (score): 0.16 ± 0.27 ↓	
Bryk et al. [25]	*Muscle strength*:	*Muscle strength*:	NR
	**Isometric voluntary contraction (kg): 16.8 ± 10.3** ↑	**Isometric voluntary contraction (kg): 9.4 ± 8.3** ↑	
	*Functionality*:	*Functionality*:	
	**TUG (s): 1.2 ± 1.8** ↓	**TUG (s): 1.6 ± 3.5** ↓	
	Lequesne (score): 5 ± 4.5 ↓	Lequesne (score): 6 ± 7.5 ↓	
Jønson et al. [50]	*Muscle strength*:	NR	*Muscle strength*:
	**Knee extension-3RM (kg): 11.43 ± 2.37** ↑		**Knee extension-3RM (kg): 8.77 ± 5.11** ↑
	Leg press-3RM (kg): 16.27 ± 9.41 ↑		Leg press-3RM (kg): 9.2 ± 6.81 ↑
	Prone leg Curl-3RM (kg): 4.20 ± 1.85 ↑		Prone leg Curl-3RM (kg): 3.33 ± 2.89 ↑
Ferraz et al. [24]	*Muscle strength*:	*Muscle strength*:	*Muscle strength*:
	**Knee extension-1RM (kg): 7.27 ± 1.34** ↑	**Knee extension-1RM (kg): 7.73 ± 3.64** ↑	**Knee extension-1RM (kg): 2.03 ± 3.14** ↑
	Leg press-1RM (kg): 31.69 ± 14.8 ↑	Leg press-1RM (kg): 44.4 ± 11.58 ↑	Leg press-1RM (kg): 9.01 ± 7.72 ↑
	*Muscle mass*:	*Muscle mass*:	*Muscle mass*:
	**CT (mm²): 310.26 ± 161.56** ↑	**CT (mm²): 366.68 ± 215.38** ↑	CT (mm²): 97.44 ± 148.74 ↑
	*Functionality*:	*Functionality*:	*Functionality*:
	**TUG (s): 0.36** ± **0.29** ↓	**TUG (s): 0.20 ± 0.81** ↓	TUG (s): 0.07 ± 0.35 ↓
	TST (repetition): 1.13 ± 0.95 ↑	TST (repetition): 1.98 ± 1.36 ↑	TST: 0.66 ± 0.97 ↑
	WOMAC-total (score): 14.4 ± 16.53 ↓	WOMAC (score): 15.4 ± 17.4 ↓	WOMAC (score): 16.7 ± 15.3 ↓
Harper et al. [22]	*Muscle strength*:	*Muscle strength*:	NR
	**Isokinetic knee extensor (Nm): 9.13 ± 11.8** ↑	**Isokinetic knee extensor (Nm): 11 ± 12.1** ↑	
	*Functionality*:	*Functionality*:	
	**400m walk gait speed (m/s): 0.04 ± 0.12** ↑	**400m walk gait speed (m/s): 0.05 ± 0.15** ↑	
	LLFDI (score): 1 ± 13.1 ↑	LLFDI (score): 7.6 ± 16.1 ↑	

**Note: Bold text indicates the test/values included on Forest plots.** Abbreviations: LIRTBFR: Low intensity resistance training combined with blood flow restriction; MIRT: Moderate intensity resistance training; HIRT: High intensity resistance training; LIRT: Low intensity resistance training without blood flow restriction; RM: Repetition maximum; kg: Kilogram; CT: Computed tomography; mm²: Square millimeter; TUG: Time up and go test; TST: Timed stands test; WOMAC: Western Ontario and McMaster Universities Osteoarthritis Index; HAQ: Health Assessment Questionnaire; Lequesne: Lequesne questionnaire; Nm: Newton meter; 400m walk gait speed: walking speed of 400 meters; m/s: Meters per seconds; LLFDI: Late Life Function and Disability Instrument.

#### Muscle strength outcome

**Fig 2:**
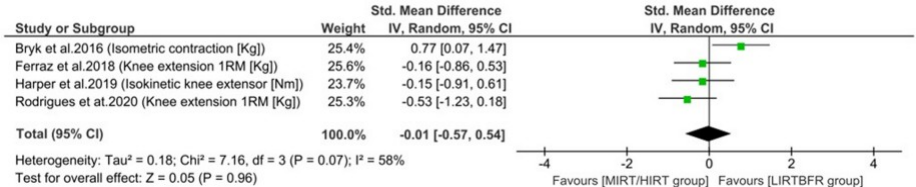
Forest plot of the comparison between LIRTBFR, MIRT and HIRT on muscle strength assessed by specific tests for quadriceps strength (n = 4 studies). LIRTBFR: Low intensity resistance training combined with blood flow restriction; MIRT: Moderate intensity resistance training; HIRT: High intensity resistance training; 1RM: 1 maximum repetition; Kg: kilogram; Nm: Newton-meter; I^2^: Heterogeneity of studies; SD: standard deviation; MD: mean difference; SMD: standardized mean difference; 95% CI: 95% confidence interval; IV: inverse variance; Random: random effects model.

From the five included studies, four studies had data extracted for meta-analysis comparing LIRTBFR with MIRT or HIRT on muscle strength in patients with chronic arthropathies (OA and RA). Three of these four studies measured muscle strength using the unit of measurement in kilograms (Kg) and one study used the unit of measurement in Newton-meter (Nm). In our study, we chose to perform the meta-analysis between the LIRTBFR and MIRT or HIRT protocols, prioritizing a general analysis of specific tests for quadriceps strength. No statistically significant difference was observed between LIRTBFR and MIRT or HIRT protocols (SMD = - 0.01, 95% CI, -0.57 to 0.54, I² = 58%; *P* = 0.96) [22–25] (Fig 2).

Few of the studies performed evaluations to measure muscle strength between LIRTBFR and LIRT. Ferraz et al. [24] showed increases in knee extension muscle strength within the group LIRTBFR (+ 23%, effect sizes (ES) = 0.86, *P* < 0.0001), but the values for LIRT group remained unaltered after the intervention (+ 7%, ES = 0.21, *P* = 0.23) in OA patients. Jonson et al. [50] showed increases of knee extension strength within the group LIRTBFR (+ 23.2%) and LIRT (+ 17.8%) in RA patients. However, LIRTBFR group had a statistically significant improvement compared to LIRT (*P* = 0.0342).

**Fig 3:**
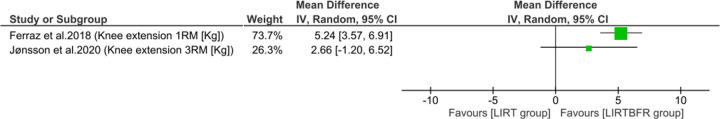
Forest plot of the comparison between LIRTBFR and LIRT on muscle strength assessed by knee extension (n = 2 studies). LIRTBFR: Low intensity resistance training combined with blood flow restriction; LIRT: Low intensity resistance training without blood flow restriction; 1RM: 1 maximum repetition; 3RM: 3 maximum repetition test; Kg: kilogram; I^2^: Heterogeneity of studies; SD: standard deviation; MD: mean difference; 95% CI: 95% confidence interval; IV: inverse variance; Random: random effects model.

While a quantitative evaluation of muscle strength comparing LIRTBFR and LIRT across studies was not possible due to the small amount of studies [24, 50], a descriptive presentation of the single effects is given in Fig 3. Additionally, the pooled effect size was omitted to avoid misleading interpretation of the results.

#### Muscle mass

Few of the studies performed evaluations to measure muscle mass. In a comparison between HIRT and LIRTBFR, Ferraz et al. [24] showed increases of muscle mass within the group HIRT (+ 8%, ES = 0.54, *P* < 0.0001) and LIRTBFR (+ 7%, ES = 0.39, *P* < 0.0001) in OA patients. Rodrigues et al. [23] also showed increases of muscle mass within the group HIRT (+ 10.8%, ES = 2.09, *P* < 0.0001) and LIRTBFR (+ 9.5%, ES = 1.89, *P* < 0.0001) in RA patients. Additionally, these same studies showed that LIRTBFR and HIRT had greater gains in muscle mass when compared to LIRT in OA patients (LIRTBFR, *P* = 0.02; HIRT, *P* = 0.007) [24] and when compared to untrained group AR patients (LIRTBFR, *P* < 0,0001; HIRT, *P* < 0,0001) [23].

**Fig 4:**
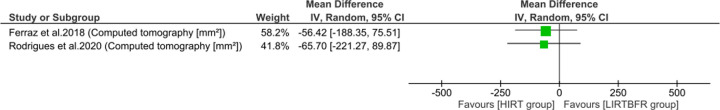
Forest plot of the comparison between LIRTBFR and HIRT on muscle mass (n = 2 studies). LIRTBFR: Low intensity resistance training combined with blood flow restriction; HIRT: High intensity resistance training; mm²: square millimeter; I^2^: Heterogeneity of studies; SD: standard deviation; MD: mean difference; 95% CI: 95% confidence interval; IV: inverse variance; Random: random effects model.

Although a quantitative assessment of muscle mass comparing LIRTBFR and HIRT between studies was not possible due to the small number of studies, a descriptive presentation of the single effects is given in Fig 4. Therefore, as well as for the analysis of muscle strength between LIRTBFR and LIRT, the pooled effect size was omitted to avoid misleading interpretation of the results. Finally, there are not enough data in the literature comparing muscle mass gains between the LIRTBFR and LIRT protocols in patients with chronic arthropathies.

#### Functionality

**Fig 5:**
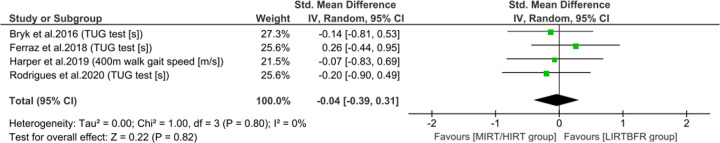
Forest plot of the comparison between LIRTBFR, MIRT and HIRT on functionality assessed by tests with patterns similar to walking (n = 4 studies). LIRTBFR: Low intensity resistance training combined with blood flow restriction; MIRT: Moderate intensity resistance training; HIRT: High intensity resistance training; TUG test: Time Up and Go test; [s]: seconds; [m/s]: meters per seconds; I^2^: Heterogeneity of studies; SD: standard deviation; MD: mean difference; SMD: standardized mean difference; 95% CI: 95% confidence interval; IV: inverse variance; Random: random effects model.

Regarding to functionality, four studies [22–25] compared LIRTBFR and MIRT or HIRT. These studies assessed functionality objectively and subjectively. For our study, we prioritized objective functionality analysis. Thus, we opted for a general analysis of tests with patterns similar to walking. No statistically significant differences were found between the LIRTBFR and MIRT or HIRT protocols (SMD = -0.04, 95% CI, -0.39 to 0.31, I² = 0%; *P* = 0.82). The results of the objective analyses are shown in Fig 5.

However, as well as for muscle mass, there are not enough data in the literature comparing LIRTBFR with LIRT protocols in chronic arthropathies patients.

### Methodological quality of the studies

The studies were classified as quality 6–8, being considered "good". The detailed methodological quality of included studies is described in Table 4. Individual analysis of methodological quality performed by reviewers is presented in S3 File.

**Table 4:** Description of quality assessment using the Physiotherapy Evidence Database (PEDro).

Studies	item 1	item 2	item 3	item 4	item 5	item 6	item 7	item 8	item 9	item 10	item 11	Sum
Rodrigues et al. [23]	-	1	0	1	0	0	1	1	1	1	1	**7**
Bryk et al. [25]	-	1	1	1	0	0	1	1	1	1	1	**8**
Jønsson et al. [50]	-	0	1	1	0	0	0	1	1	1	1	**6**
Ferraz et al. [24]	-	1	0	1	0	0	0	1	1	1	1	**6**
Harper et al. [22]	-	0	0	1	0	0	1	1	1	1	1	**6**

0 = Did not score; 1 = Scored; Represents the number of “points” of quality The Physiotherapy Evidence Database (PEDro). The maximum possible score was 10 points.

### Risk of bias

The articles were evaluated according to the randomization of process, deviations from intended interventions, missing outcome data, measurement of the outcome, and selection of the reported result. In conclusion, the most studies showed high risk of bias (Table 5). Individual analysis performed by reviewers in RoB2 is presented in S4 File.

**Table 5:** Methodological quality of the studies using the tool RoB 2.0.

First author name	Randomization process	Deviations from the intended interventions	Missing results data	The measurement result	Selection of the result reported	General trend
Rodrigues et al. [23]	High	Some concerns	Some concerns	Low	Low	High
Bryk et al. [25]	High	Some concerns	Low	Low	Low	High
Jønsson et al. [50]	Low	Low	Low	Low	Low	Low
Ferraz et al. [24]	High	Some concerns	Low	Some concerns	Low	High
Harper et al. [22]	High	Some concerns	Low	Low	Low	High

## Discussion

Rheumatoid arthritis (RA) and osteoarthritis (OA) are two of the most common types of arthritis. Patients diagnosed with RA or OA often suffer from joint damage [52]. OA is characterized by cartilage degeneration, bone remodeling, and mild to moderate inflammation of the synovial lining [1–3, 12]. RA is a chronic, autoimmune and systemic inflammatory disease that mainly affects large joints. Although the pathophysiology of AR and OA is distinct, some articular and extra-articular manifestation such as reduction of muscle strength, muscle mass and functionality are similar [4–6, 12, 15, 17, 18]. Such complications lead many of these patients to adopt sedentary lifestyles, corroborating with high levels of physical inactivity and, consequently, accentuating problems associated with body composition. It is known that the main strategies to reduce the impact of these muscle losses are moderate intensity resistance training (MIRT) and high intensity resistance training (HIRT). However, in some cases, neither of the protocols (> 60% 1RM) is tolerated or prescribed for this population. Thus, strategies such as low intensity resistance training combined with blood flow restriction (LIRTBFR) and its results have been discussed in the literature and indicated for chronic arthropathies patients. Therefore, summarizing the effects across training protocols through our systematic review with meta-analysis is necessary and important.

The main findings of this systematic review with meta-analysis were that LIRTBFR showed no differences in effects compared to MIRT and HIRT for muscle strength, muscle mass and functionality in a predominantly sample of RA women. On the other hand, the number of articles comparing LIRTBFR with low intensity resistance training without blood flow restriction (LIRT) is still small, however their comparisons on muscle strength assessed by knee extension seem to indicate favorable effects for LIRTBFR. Therefore, these results points to LIRTBFR as a promising training strategy in female patients with RA and OA (chronic arthropathies).

Regarding to physical training and muscle strength, physiologically, the increase of muscle strength is related to recruitment of motors units, stimulation frequency [53] and neuromuscular adaptations caused by high loads of resistance training [54, 55]. On the other hand, LIRTBFR, with 20–30% 1RM, can also increase the muscle strength. We believe that this increase occur due to stressed metabolic environment and hypoxia generated by the cuff [35, 56]. This stressed metabolic environment and hypoxia develop a subsequent increase in anabolic growth stimuli, increase recruitment of fast fibers [37, 56] and lead to neuromuscular adaptation [35]. However, when low intensity training was performed without blood flow restriction, LIRTBFR demonstrated a favorable effect compared to LIRT on knee extension strength. So, it is speculated that the restriction of blood flow generated by cuff has fundamental role in increases of muscle strength. Considering that some patients with chronic arthropathies did not tolerate MIRT or HIRT due to clinical manifestation [26–29] and that low muscle strength is a key characteristic of sarcopenia [57], LIRTBFR appears be a therapeutic strategy important to the maintenance and improvement of muscle strength in these patients.

The gains in muscle mass are directly related to the increase in muscle tension, collaborating with a primary stimulus triggering the process of muscle hypertrophy [58]. During resistance training, ruptures occur in the Z lines of the sarcomeres and remodeling of proteins that constitute muscle fiber. This leads to protein degradation and micro-injuries in muscle fibers [58]. After resistance training, in response to the catabolic process in initial phases, there is an increase in protein synthesis [59, 60] and proliferation of satellite cells [61, 62], essential for the adaptation process to promote increased muscle mass. On the other hand, the mechanisms involving LIRTBFR do not seem to depend on the load used to promote increases on muscle mass [63]. Like muscle strength, the hypoxia generated by blood flow restriction is directly related to muscle hypertrophy [36, 63–65]. This decrease in oxygen in muscle tissue leads to an accumulation of metabolites, leading to an increase in the plasma concentration of growth hormone (GH), as well as an increase in lactate levels and the proliferation of satellite cells. This hypoxia also seems to be involved with stimulation of mammalian target of rapamycin (mTOR), inhibition of myostatin and increase in shock proteins [36, 63–65].These reactions are associated with the process of muscle hypertrophy.

It is noteworthy that, in RA [66] and OA [67], patients suffer from a reduction in muscle mass, leading them to conditions of sarcopenia. The systemic mechanisms of muscle wasting in RA and OA (chronic arthropathies) patients are related to several factors. Among these factors, we highlight the increase in myostatin [68], which is known as a negative regulator of muscle mass growth [69], and the deficiency in the activation of muscle satellite cells [69, 70]. On the other hand, the mechanisms that involve LIRTBFR show that the training method is able to assist with the inhibition of myostatin and the increase of the proliferation capacity and differentiation of satellite cells [36, 63–65]. Thus, we speculate that LIRTBFR is acting directly on these two pathways leading to an increase in muscle mass in these patients. Therefore, considering that there are muscular deficit of both chronic arthropathies and difficulty in some patients in tolerating the practice of physical training with significant loads, LIRTBFR appears as a potential training methodology for increasing muscle mass, as well as MIRT and HIRT.

This increase in muscle mass is already well established and shown in the literature in young [19] and old healthy adults [71] and are now extended to populations with chronic arthropathies [23, 24]. Although it was not possible to perform a meta-analysis comparing LIRTBFR and LIRT, we believe that LIRTBFR is more efficient than LIRT in promoting muscle mass gains, as well as Ferraz et al. [24] showed in their study with twelve weeks of intervention on OA patients. On the other hand, Segal et al. [72], who analyzed women at risk for OA and, widely used in systematic reviews referring to people with OA, demonstrated similar effects on muscle volume assessed by magnetic resonance imaging between LIRTBFR and LIRT. However, we believe that this finding among interventions in the study by Segal et al. [72] may have happened due to the short time intervention of four weeks or due to the evaluation method, which randomly selected only six individuals from each group to assess muscle mass. Therefore, this increase is not related to the load used, but to the method of vascular occlusion. These findings are of clinical relevance, due to the fact that patients with RA and OA present low of muscle mass [6, 73] compared to healthy people, showing the LIRTBFR also as an effective therapeutic strategy in increasing muscle mass for patients with chronic arthropathies. Consequently, both the increase in muscle strength and the increase in muscle mass for these populations will be important for decreasing the risk of falls, improving aspects of muscle fatigue and also improving the quality of life of these patients.

Functionality is an extremely important parameter in chronic arthropathies patients and it is directly influenced by aspects of muscle strength and muscle mass [4, 74, 75]. In addition, the functionality presents itself as a potential predictor for sarcopenia [57], a frequent condition in these populations [76, 77]. Our results showed that LIRTBFR was similar when compared to MIRT and HIRT for functionality in tests with patterns similar to walking (TUG test and 400m walk gait speed). This finding is extremely important, considering the management of both chronic arthropathies. Therefore, the maintenance of functionality allows an improved quality of life and greater independence in the activities performed on a daily basis by patients with OA and RA. In this sense, LIRTBFR shows itself as a tool capable of promoting gains in functionality, as well as MIRT and HIRT, being extremely important for these patients. On the other hand, only one study evaluated the functionality between LIRTBFR and LIRT, and it was not possible to perform a meta-analysis for functionality in patients with chronic arthropathies.

It is noteworthy that the moderate or high intensity resistance training protocols are extremely sufficient and effective for promoting gains on muscle strength and muscle mass [20, 21], which will, consequently, lead to improvement in the functionality. In addition, the strengthening of lower limb muscles plays an important role in muscle strength, muscle mass, functionality [22–25] and pain relief [78] for these populations. On the other hand, it is known that some patients with chronic arthropathies usually avoid physical activities for having fear of exacerbating the symptoms of the disease [79] or for ending up getting discomfort when performing high-load exercises [32, 80]. For this reason, in some cases, MIRT or HIRT prescriptions for chronic arthropathies patients may be challenging and difficult to adhere to. Therefore, considering that some patients do not tolerate performing exercises with high loads due to articular manifestation and, because we have an effective method that is performed with lower loads, patients with RA and OA may find it easier to perform it. In this sense, mobility, combined with handling loads during LIRTBFR, may lead these patients to a greater frequency and efficiency in training and, consequently, may improve muscle strength, muscle mass and functionality. Thus, LIRTBFR protocol becomes relevant and promising in this population, since the results found in our systematic review with meta-analysis are positive.

In our study, we used PEDro scale to describe the quality of each study included in our systematic review with meta-analysis. The majority of studies were identified with good quality. However, when analyzing the risks of bias in these studies, most studies were classified as high risk of bias. This high risk of bias occurred due to failures in the process of randomization and deviations from the intended interventions.

Finally, this study systematic review with meta-analysis has some limitations. First, there was a small number of studies included, and it is necessary to group the meta-analysis between patients with OA and RA (chronic arthropathies). However, considering articular and extra-articular manifestation, this analysis is still important. In addition, the selected studies present high risk of bias. However, as much as there is this high risk of bias between the articles, these are the articles that currently exist in the literature on the subject LIRTBFR in these populations. Second, we found high heterogeneity in the included studies comparing LIRTBFR group with MIRT and HIRT groups for the outcomes of muscle strength on general analysis of specific tests for quadriceps strength (I² = 58%). We speculate that these findings may be related to variations in tests used between LIRTBFR, MIRT or HIRT protocols and, although isometric, isokinetic and 1RM tests are widely used for strength analysis, they are different. In addition, in the clinical context, for these populations diagnosed with RA and OA, an isometric test may be better performed and obtain greater tolerance for these patients, taking into account their articular manifestations. Thus, an isometric test performed with high load tends to have a lower mechanical load on the articulation compared to a dynamics test with high load. So, considering that joint movement performed in a dynamic test can cause greater joint overload, we consider the performance of an isometric test more clinically relevant for this population. Therefore, we believe that it is interesting to standardize muscle strength analyzes in patients with chronic arthropathies.

In addition, it was not possible to compare LIRTBFR and LIRT for knee extension muscle strength results due to the number of studies included (n = 2). In this case, we decided to omit the size of the pooled effect in order to avoid misinterpreting the forest plot (Fig 3). Therefore, based on a descriptive presentation of these data, we draw attention to some still conflicting findings among current studies. This data omission analysis was extended to muscle mass results comparing LIRTBFR and HIRT (Fig 4). Furthermore, it was not possible to perform a meta-analysis for the outcome of muscle mass and functionality comparing LIRTBFR group with LIRT group, reinforcing the need for further studies addressing the vascular occlusion protocol in patients with chronic arthropathies. Finally, these findings were based on a majority sample of women, limiting a general statement of effect. However, although limiting, these findings are still important considering that the prevalence of cases of rheumatoid arthritis and osteoarthritis is higher in females.

## Conclusion

The low intensity resistance training combined with blood flow restriction (LIRTBFR) appears to be a promising strategy when compared to moderate and high intensity resistance training (> 60% 1RM) in terms of gaining muscle strength, muscle mass and functionality in 6–12 weeks in a predominantly sample of women with RA and OA. In addition, LIRTBFR appears to be better than low intensity resistance training without blood flow restriction in terms of gaining knee extension muscle strength in 4–12 weeks in a predominantly sample of women with RA and OA. So, the LIRTBFR method may appear as a therapeutic strategy for female patients with chronic arthropathies. However, more studies with low risk of bias, with similar test procedures and studies comparing both protocols about muscle strength, muscle mass and functionality results are needed to better elucidate the effects of LIRTBRF, since the number of articles found with patients diagnosed with OA and RA is still low.

## Supporting information

S1 File PRISMA checklist. (PDF)

S2 File Data extraction between reviewers. (PDF)

S3 File Individual analysis of methodological quality by reviewers. (PDF)

S4 File Individual analysis performed by reviewers in RoB2. (PDF)
