# Supplementary material for: The effects of resistance training with blood flow restriction on muscle strength, muscle hypertrophy and functionality in patients with osteoarthritis and rheumatoid arthritis: A systematic review with meta-analysis
Source: PLoS One. 2021 Nov 10;16(11):e0259574. doi: 10.1371/journal.pone.0259574 (PMC8580240; doi:10.1371/journal.pone.0259574)
Supplement: S2 File — (PDF) [file pone.0259574.s002.pdf]

n	Year	Title	Decision (Y / N)	Remarks / reason (Reviewer #1)	Remarks / reason (Reviewer #2)	Consensus (Reviewers #3 and #4)
1	2010	Acupuncture	No	Other topics	Other topics	-
2	2010	Other complementary therapies	No	Without intervention/comparator	Without intervention/comparator	-
3	2010	Treatment of Kunxian capsule in rheumatoid arthritis patients: a multi-center trial	No	Without intervention/comparator	Without intervention/comparator	-
4	2011	The Effect of Omega-3 Fatty Acid Supplementation on the Inflammatory Response to eccentric s	No	Without intervention/comparator	Without intervention/comparator	-
5	2012	Safety and pharmacokinetics of CDP6038, an ANTI-IL-6 monoclonal antibody, administered by	No	Other populations/diseases	Other populations/diseases	-
6	2013	2013 SYR Accepted Poster Abstracts	No	Other topics	Other topics	-
7	2013	Undenatured type II collagen (UC-II) for joint support: a randomized, double-blind, placebo-con	No	Other populations/diseases	Other populations/diseases	-
8	2014	Two different sets of handexercises improved grip strength after eight weeks in patients with artl	No	Without intervention/comparator	Without intervention/comparator	-
9	2015	A low-fat yoghurt supplemented with a rooster comb extract on muscle joint function in adults w	No	Without intervention/comparator	Without intervention/comparator	-
10	2015	Global progressive resistance training improved functional capacity in patients with rheumatoid	No	Without intervention/comparator	Without intervention/comparator	-
11	2015	Internet-based implementation of non-pharmacological interventions of the "people getting a gri	No	Without intervention/comparator	Without intervention/comparator	-
12	2015	Is there a role for rehabilitation streaming following total knee arthroplasty? Preliminary insights	No	Without intervention/comparator	Without intervention/comparator	-
13	2015	Randomized exercise trial of aromatase inhibitor-induced arthralgia in breast cancer survivors	No	Other populations/diseases	Other populations/diseases	-
14	2015	The Incremental Effects of Manual Therapy or Booster Sessions in Addition to Exercise Therapy	No	Without intervention/comparator	Without intervention/comparator	-
15	2016	A randomised trial comparing the efficacy and safety of topical ketoprofen in Transfersome() gel	No	Other populations/diseases	Other populations/diseases	-
16	2016	Clinical and radiographic outcomes of the Yespliciti canal-sparing shoulder arthroplasty system	No	Without intervention/comparator	Without intervention/comparator	-
17	2016	Efficacy of Preoperative Progressive Resistance Training on Postoperative Outcomes in Patients	No	Other populations/diseases	Other populations/diseases	-
18	2016	Preoperative Hypoalgesia after Cold Pressor Test and Aerobic Exercise is Associated with Pain I	No	Without intervention/comparator	Without intervention/comparator	-
19	2016	Reporting of sex effects by systematic reviews on interventions for depression, diabetes, and chr	No	Review/Systematic Review/Meta-Analy:	Review/Systematic Review/Meta-Analys	-
20	2016	Selected summaries: exercise or surgery for meniscal tears: do we have an answer?	No	Other populations/diseases	Other populations/diseases	-
21	2016	The effect of cardiac rehabilitation program on disease activity, functional outcome and cardiova	No	Without intervention/comparator	Without intervention/comparator	-
22	2016	The effects of closed kinetic chain exercise on articular cartilage morphology: myth or reality? A	No	Without intervention/comparator	Without intervention/comparator	-
23	2016	Total knee replacement or non-surgical therapy for osteoarthritis of the knee?	No	Without intervention/comparator	Without intervention/comparator	-
24	2016	Ultrashort versus Conventional Anatomic Cementless Femoral Stems in the Same Patients Youn	No	Without intervention/comparator	Without intervention/comparator	-
25	2017	Baseline lower limb muscle strength in younger women and its change in over 12 years indepenc	No	Without intervention/comparator	Without intervention/comparator	-
26	2017	Blood flow restricted resistance training in older adults at risk of mobility limitation	No	Other populations/diseases	Other populations/diseases	-
27	2017	Clinical relevance commentary in response to: effectiveness of a fine motor skills rehabilitation p	No	Without intervention/comparator	Without intervention/comparator	-
28	2017	Comparing the effectiveness of laser treatment with extracorporeal shock wave treatment (ESWT	No	Other populations/diseases	Other populations/diseases	-
29	2017	Effect of circuit training in osteoarthritis of knee	No	Without intervention/comparator	Without intervention/comparator	-
30	2017	Effectiveness of an overall progressive resistance strength program for improving the functional	No	Without intervention/comparator	Without intervention/comparator	-
31	2017	Effectiveness of low-dose radiation therapy on symptoms in knee osteoarthritis: first results of a	No	Without intervention/comparator	Without intervention/comparator	-
32	2017	Effects of two exercise programs on posture and back pain in community-dwelling elderly with l	No	Other populations/diseases	Other populations/diseases	-
33	2017	Effects of underwater ultrasound therapy on pain, inflammation, hand function and quality of lif	No	Without intervention/comparator	Without intervention/comparator	-
34	2017	Effects ofa giant exercising board game intervention on ambulatory physical activity among nurs	No	Without intervention/comparator	Without intervention/comparator	-
35	2017	Exercise-induced pain reductions in knee osteoarthritis are associated with changes in muscle pe	No	Without intervention/comparator	Without intervention/comparator	-
36	2017	Forearm grip strength is maintained by 1000 IU of supplemental vitamin D over 6 months and n	No	Without intervention/comparator	Without intervention/comparator	-
37	2017	Hydrotherapy improves pain and function in older women with knee osteoarthritis: a randomized	No	Without intervention/comparator	Without intervention/comparator	-
38	2017	Impact of treatment strategy and physical performance on knee self-efficacy in individuals with a	No	Other populations/diseases	Other populations/diseases	-
39	2017	Performance comparison of single-radius versus multiple-curve femoral component in total knee	No	Without intervention/comparator	Without intervention/comparator	-
40	2017	Physical Activity Is Related with Cartilage Quality in Women with Knee Osteoarthritis	No	Without intervention/comparator	Without intervention/comparator	-
41	2017	Rehabilitation and quality of life in females with rheumatoid arthritis and osteoporosis	No	Without intervention/comparator	Without intervention/comparator	-

42	2017	Response to the comments on "Effects of high intensity aquatic resistance training on body comp	No	Without intervention/comparator	Without intervention/comparator	-
43	2017	The effect of nerve mobilization exercise in patients with rheumatoid arthritis: a pilot study	No	Without intervention/comparator	Without intervention/comparator	-
44	2018	A randomized trial of a motivational interviewing intervention to increase lifestyle physical activ	No	Without intervention/comparator	Without intervention/comparator	-
45	2018	A scoping review of preoperative intervention in patients waiting for total knee arthroplasty	No	Review/Systematic Review/Meta-Analys	Review/Systematic Review/Meta-Analys	-
46	2018	Change in physical activity level and clinical outcomes in older adults with knee pain: a seconda	No	Without intervention/comparator	Without intervention/comparator	-
47	2018	Comparative impact of nonpharmacological interventions on pain of knee osteoarthritis patients	No	Without intervention/comparator	Without intervention/comparator	-
48	2018	Comparing the effectiveness of kinesio taping and hand exercise on pain, range of motion and gr	No	Without intervention/comparator	Without intervention/comparator	-
49	2018	Comparison of efficiency between corticosteroid and platelet rich plasma injection therapies in p	No	Without intervention/comparator	Without intervention/comparator	-
50	2018	Comparison of patellar distraction with patellar glides in female patients with patellofemoral pai	No	Other populations/diseases	Other populations/diseases	-
51	2018	Does frontal knee kinematics predict treatment outcomes? Exploratory analyses from the Intensiv	No	Without intervention/comparator	Without intervention/comparator	-
52	2018	Effect of continuous passive motion (CPM) exercises on post total knee arthroplasty (TKA) reha	No	Without intervention/comparator	Without intervention/comparator	-
53	2018	Effectiveness of self-management program on arthritis symptoms among older women: a random	No	Without intervention/comparator	Without intervention/comparator	-
54	2018	Effectiveness of superficial radial nerve block on pain, function and quality of life in patients wi	No	Without intervention/comparator	Without intervention/comparator	-
55	2018	Effects of a massage-like essential oil application procedure using Copaiba and Deep Blue oils in	No	Without intervention/comparator	Without intervention/comparator	-
56	2018	Effects of isokinetic, isometric, and aerobic exercises on clinical variables and knee cartilage vol	No	Without intervention/comparator	Without intervention/comparator	-
57	2018	Effects of nurse-led lower extremity strength training on knee function recovery in patients who	No	Other populations/diseases	Other populations/diseases	-
58	2018	Evaluation of the self management of osteoarthritis and low back pain through activity and skills	No	Without intervention/comparator	Without intervention/comparator	-
59	2018	Feasibility of enabling self-management and coping with arthritic pain using exercise (ESCAPE-	No	Without intervention/comparator	Without intervention/comparator	-
60	2018	Feasibility of Training Physical Therapists to Deliver the Theory-Based Self-Management of Ost	No	Without intervention/comparator	Without intervention/comparator	-
61	2018	Influence of isometric exercise on pressure pain sensitivity in knee osteoarthritis	No	Without intervention/comparator	Without intervention/comparator	-
62	2018	Is high-intensity interval cycling feasible and more beneficial than continuous cycling for knee o	No	Without intervention/comparator	Without intervention/comparator	-
63	2018	Knee extensor strength gains mediate symptom improvement in knee osteoarthritis: secondary an	No	Without intervention/comparator	Without intervention/comparator	-
64	2018	Muscular strength and function after total hip arthroplasty performed with three different surgica	No	Other populations/diseases	Other populations/diseases	-
65	2018	Preliminary findings of a novel physical therapist administered physical activity intervention afte	No	Other populations/diseases	Other populations/diseases	-
66	2018	The effect of a 12 week prehabilitation program on pain and function for patients undergoing tot	No	Other populations/diseases	Other populations/diseases	-
67	2018	The effect of education and supervised exercise on physical activity, pain, quality of life and self	No	Without intervention/comparator	Without intervention/comparator	-
68	2018	The effect of self-management training on pain intensity in patients with knee osteoarthritis refer	No	Without intervention/comparator	Without intervention/comparator	-
69	2018	The effects of exercise on synovitis and bone marrow lesions in knee osteoarthritis: secondary ou	No	Without intervention/comparator	Without intervention/comparator	-
70	2018	To compare the effectiveness between LASER and neuromuscular electrical stimulation in knee	No	Without intervention/comparator	Without intervention/comparator	-
71	2018	Training with Hybrid Assistive Limb for walking function after total knee arthroplasty	No	Other populations/diseases	Other populations/diseases	-
72	2018	Trajectories of adherence to home-based exercise programs among people with knee osteoarthrit	No	Without intervention/comparator	Without intervention/comparator	-
73	2019	"I could do it in my own time and when I really needed it": perceptions of online pain coping ski	No	Without intervention/comparator	Without intervention/comparator	-
74	2019	2019 AOSSM Annual Meeting Abstracts	No	Other topics	Without intervention/comparator	Other topics
75	2019	A comparative randomized controlled trial study: the Effects of Court-Type Thai Traditional Ma	No	Without intervention/comparator	Without intervention/comparator	-
76	2019	A comparative study on the effectiveness of PNF stretching versus static stretching on Pain and I	No	Without intervention/comparator	Without intervention/comparator	-
77	2019	Aquatic Exercise Program for Individuals With Osteoarthritis: pain, Stiffness, Physical Function	No	Without intervention/comparator	Without intervention/comparator	-
78	2019	Association between baseline meniscal extrusion on MRI and long-term incident knee osteoarthr	No	Without intervention/comparator	Without intervention/comparator	-
79	2019	Comparison of efficacy of different rehabilitation approaches in individuals with knee osteoarthr	No	Without intervention/comparator	Without intervention/comparator	-
80	2019	Comparison of low-profile locking plate fixation versus antegrade intramedullary nailing for uns	No	Other populations/diseases	Other populations/diseases	-
81	2019	Comparison of radial extracorporeal shock wave therapy and traditional physiotherapy in rotator	No	Other populations/diseases	Other populations/diseases	-
82	2019	Does proprioception of knee improve after various forms of training in osteoarthritis of knee?	No	Without intervention/comparator	Without intervention/comparator	-
83	2019	Early functional recovery of direct anterior approach versus anterolateral approach for total hip a	No	Other populations/diseases	Other populations/diseases	-
84	2019	Effect of deep slow breathing on pain-related variables in osteoarthritis	No	Without intervention/comparator	Without intervention/comparator	-
85	2019	Effect of ground level reverse treadmill walking versus incline reverse treadmill walking as an ac	No	Without intervention/comparator	Without intervention/comparator	-

86	2019	Effects of low-intensity pulsed ultrasound on recovery of physical impairments, functional perfo	No	Other populations/diseases	Other populations/diseases	-
87	2019	Efficacy of exercise rehabilitation in patients with knee osteoarthritis	No	Without intervention/comparator	Without intervention/comparator	-
88	2019	Efficacy of short foot exercise on medial compartment osteoarthritis knee among subjects with o	No	Without intervention/comparator	Without intervention/comparator	-
89	2019	Efficacy of total knee arthroplasty on patients with severe knee osteoarthritis	No	Without intervention/comparator	Without intervention/comparator	-
90	2019	Evaluation of the effectiveness of complex treatment of Obesity on the clinical manifestations of	No	Without intervention/comparator	Without intervention/comparator	-
91	2019	IL-6: a hack to body fat and exercise?	No	Without intervention/comparator	Other populations/diseases	Other populations/dis
92	2019	Maternal Epidural Steroids to Prevent Neonatal Exposure to Hyperthermia and Inflammation	No	Other populations/diseases	Other populations/diseases	-
93	2019	Monitoring the clinical response to an innovative transdermal delivery system for ibuprofen	No	Other populations/diseases	Without intervention/comparator	Other populations/dis
94	2019	Multinutrient supplements found to have no benefit in preventing depression onset	No	Other populations/diseases	Other populations/diseases	-
95	2019	Novel modification of abductor pollicis longus suspension arthroplasty with trapeziectomy for th	No	Without intervention/comparator	Without intervention/comparator	-
96	2019	Outcomes of children receiving Group-Early Start Denver Model in an inclusive versus autism-s	No	Other populations/diseases	Other populations/diseases	-
97	2019	Pain relief and gain of function from low dose radiotherapy for epicondylitis, finger osteoarthriti	No	Without intervention/comparator	Other populations/diseases	Without intervention/c
98	2019	Prognostic factors for knee pain, symptoms, function and quality of life 32-37 years after surgica	No	Other populations/diseases	Other populations/diseases	-
99	2019	The Impact of Aspirin on Ultrasound Markers of Uteroplacental Flow in Low-Risk Pregnancy: s	No	Other populations/diseases	Other populations/diseases	-
100	2019	Twenty-four-week hospital-based progressive resistance training on functional recovery in femal	No	Other populations/diseases	Other populations/diseases	-
101	2019	Muscle strength and muscle mass one year after an initial 16 week intense weight loss: a random	No	Without intervention/comparator	Without intervention/comparator	-
102	2014	The effect of quadriceps-strengthening exercise on quadriceps and knee biomichanics during wa	No	Without intervention/comparator	Without intervention/comparator	-
103	2018	Perioperative platelet rich plasma (PRP) in total hip arthroplasty through the Hardinge approach:	No	Without intervention/comparator	Without intervention/comparator	-
104	2011	The effectiveness of physiotherapeutic rehabilitation and issues of outcome prediction after lumb	No	Other populations/diseases	Other populations/diseases	-
105	2012	Exercise therapy, manual therapy, or both, for management of osteoarthritis of the hip or knee: e	No	Without intervention/comparator	Without intervention/comparator	-
106	2013	Manual therapy, exercise therapy, or both, in addition to usual care, for osteoarthritis of the hip c	No	Without intervention/comparator	Without intervention/comparator	-
107	2014	Exercise therapy and/or manual therapy for hip or knee osteoarthritis: 2-year follow-up of a rand	No	Without intervention/comparator	Without intervention/comparator	-
108	2014	Exercise therapy, manual therapy, or both, for management of osteoarthritis of the hip or knee: 2	No	Without intervention/comparator	Without intervention/comparator	-
109	2014	Predictive validity of the pain belief screening instrument for estimating pain responsiveness to t	No	Without intervention/comparator	Without intervention/comparator	-
110	2015	Do manual therapy or booster sessions in addition to exercise therapy for knee osteoarthritis prov	No	Without intervention/comparator	Without intervention/comparator	-
111	2015	The Incremental Effects of Manual Therapy or Booster Sessions in Addition to Exercise Therapy	No	Without intervention/comparator	Without intervention/comparator	-
112	2016	Clinical predictors of response to exercise & manual therapy interventions for patients with hip c	No	Without intervention/comparator	Without intervention/comparator	-
113	2018	Incremental clinical effectiveness and cost effectiveness of providing supervised physiotherapy i	No	Without intervention/comparator	Without intervention/comparator	-
114	2019	Long-term cost-effectiveness of exercise therapy and/or manual therapy for hip or knee osteoarth	No	Without intervention/comparator	Without intervention/comparator	-
115	2015	The Incremental Effects of Manual Therapy or Booster Sessions in Addition to Exercise Therapy	No	Without intervention/comparator	Without intervention/comparator	-
116	2013	Effects of physical activity on inflammation, skeletal muscle strength/function (sarcopenia) and	No	Without intervention/comparator	Without intervention/comparator	-
117	2017	Does a lack of physical activity explain the rheumatoid arthritis lipid profile?	No	Without intervention/comparator	Without intervention/comparator	-
118	2019	Perineural injection therapy; a new modality in management of mechanical low back pain; a con	No	Other populations/diseases	Other populations/diseases	-
119	2005	Muscle Strength vs. Muscle Endurance Training for Osteoarthritis of the Knee in Older Adults;	No	Without intervention/comparator	Without intervention/comparator	-
120	2006	Clinical Outcomes of Increased Flexion Total Knee Replacement	No	Other populations/diseases	Other populations/diseases	-
121	2006	Telerehabilitation for patients with total knee replacement	No	Other populations/diseases	Other populations/diseases	-
122	2007	A lifestyle intervention to improve function and health outcomes in older adults with knee osteo	No	Without intervention/comparator	Without intervention/comparator	-
123	2007	Effectiveness of Neuromuscle electrical stimulation in rehabilitation of knee osteoarthritis patien	No	Without intervention/comparator	Without intervention/comparator	-
124	2009	A randomised controlled trial on the effects of Aqua Aerobics on improving the falls risk and ph	No	Without intervention/comparator	Without intervention/comparator	-
125	2010	Effectiveness of physiotherapy resources in knee osteoarthritis: a randomized controlled trial wit	No	Without intervention/comparator	Without intervention/comparator	-
126	2010	The effects of a physiotherapy program on pain and physical function in individuals with hip joi	No	Without intervention/comparator	Without intervention/comparator	-
127	2010	The effects of adding neuromuscular electrical stimulation to standard inpatient rehabilitation on	No	Other populations/diseases	Other populations/diseases	-
128	2011	Mindfulness, cognitive processes and coping in chronic illness: insights from a study of joint rep	No	Other populations/diseases	Other populations/diseases	-
129	2012	Eccentric resistance ergometry in knee osteoarthritis rehabilitation: a randomized clinical trial	No	Without intervention/comparator	Without intervention/comparator	-

130	2012	Prospective Evaluation of Vibryntâs Single Port Access Restrictive Therapy to Improve Life for t	No	Other populations/diseases	Other populations/diseases	-
131	2012	Yoga for rheumatoid arthritis â A pilot randomised controlled trial	No	Without intervention/comparator	Without intervention/comparator	-
132	2013	Online treatment for depression in people with osteoarthritis: a randomised controlled trial	No	Without intervention/comparator	Without intervention/comparator	-
133	2014	Multimorbidity rehabilitation in chronic disease: disease-specific compared to generic rehabilita	No	Other populations/diseases	Other populations/diseases	-
134	2014	Multimorbidity rehabilitation in chronic disease: general rehabilitation compared to usual care	No	Without intervention/comparator	Without intervention/comparator	-
135	2014	The Foot Orthosis versus Hip eXercises (FOHX) trial: predicting success in patellofemoral pain	No	Other populations/diseases	Other populations/diseases	-
136	2014	The implementation of effective non-pharmacological interventions of the People Getting a Grip	No	Without intervention/comparator	Without intervention/comparator	-
137	2015	A pilot double-blind randomised controlled trial comparing two physiotherapy interventions to tr	No	Other populations/diseases	Other populations/diseases	-
138	2015	Can combined exercise and manual therapy alter the need for surgery in patients with advanced	No	Without intervention/comparator	Without intervention/comparator	-
139	2015	Full randomised controlled trial of Arthroscopic Surgery for Hip Impingement versus best coNve	No	Other populations/diseases	Other populations/diseases	-
140	2015	Prescribing the maximum tolerated dose of walking for people with severe knee osteoarthritis: a	No	Without intervention/comparator	Without intervention/comparator	-
141	2016	A multi-faceted podiatry intervention for first metatarsophalangeal joint osteoarthritis: a pilot ran	No	Without intervention/comparator	Without intervention/comparator	-
142	2016	A Randomised Controlled Study on the Maxm Skate: a Lower Limb Rehabilitation Device for us	No	Other populations/diseases	Other populations/diseases	-
143	2016	A randomised trial of krill oil for osteoarthritis of the knee	No	Without intervention/comparator	Without intervention/comparator	-
144	2016	A web-support intervention to promote recovery following Anterior Cruciate Ligament Reconstr	No	Other populations/diseases	Other populations/diseases	-
145	2016	Accelerated Rehabilitation After Anterior Cruciate Ligament (ACL) Hamstring Reconstruction	No	Other populations/diseases	Other populations/diseases	-
146	2016	Early aquatic physiotherapy after total hip and total knee replacement surgery	No	Other populations/diseases	Other populations/diseases	-
147	2016	Effect of combined conservative therapies on clinical outcomes in patients with thumb base oste	No	Without intervention/comparator	Without intervention/comparator	-
148	2016	Effectiveness of Aerobic Exercise in Sitting Posture versus Aerobic Exercise in Standing Posture	No	Without intervention/comparator	Without intervention/comparator	-
149	2016	Efficacy of health promotion interventions in adults with lifestyle and health risk factors for dem	No	Other populations/diseases	Other populations/diseases	-
150	2016	Evaluating transcatheter arterial embolisation for improvement of pain in osteoarthritis (OA) of t	No	Without intervention/comparator	Without intervention/comparator	-
151	2016	Evaluation of Preoperative Physiotherapy and Rehabilitation on functional Outcomes in Patients	No	Other populations/diseases	Other populations/diseases	-
152	2016	Extracorporeal shock wave therapy (ESWT) and kinesiotherapy (KIN) on women with bilateral k	No	Without intervention/comparator	Without intervention/comparator	-
153	2016	Feasibility and potential benefits of high-intensity interval cycling for knee osteoarthritic patient	No	Without intervention/comparator	Without intervention/comparator	-
154	2016	High speed resistance training and balance training for people with knee osteoarthritis to reduce	No	Without intervention/comparator	Without intervention/comparator	-
155	2016	Is targeted physiotherapy effective in improving outcomes, quality of life and return to activity in	No	Without intervention/comparator	Without intervention/comparator	-
156	2016	Shoe stiffening inserts for pain of the big toe joint of the foot	No	Without intervention/comparator	Without intervention/comparator	-
157	2017	A double-blind randomised controlled trial comparing two physiotherapy interventions to treat h	No	Other populations/diseases	Other populations/diseases	-
158	2017	A pedaling-based three exercise protocol compared to a non-pedaling ten exercise protocol for in	No	Other populations/diseases	Other populations/diseases	-
159	2017	App-Based Supplemental Exercise During Inpatient Orthopaedic Rehabilitation Increases Activi	No	Without intervention/comparator	Without intervention/comparator	-
160	2017	App-Based Supplemental Exercise During Inpatient Rehabilitation Increases Activity Levels: a F	No	Without intervention/comparator	Without intervention/comparator	-
**161**	**2017**	**Blood flow restriction training for hand osteoarthritis: a randomised controlled trial**	**Yes**	**Full**	**Full**	**-**
162	2017	BRAIN Training Trial: balance, Resistance, or INterval Training Trial: a Randomised Controlled	No	Other populations/diseases	Other populations/diseases	-
163	2017	METHODS - A randomised controlled trial of METhotrexate to treat Hand Osteoarthritis with S	No	Without intervention/comparator	Without intervention/comparator	-
164	2017	Targeted gluteal exercise program versus sham exercise program for people with hip osteoarthrit	No	Without intervention/comparator	Without intervention/comparator	-
165	2017	The HIHO 2 Study: hospital Inpatient versus Home-based Rehabilitation after Total Hip Replace	No	Other populations/diseases	Other populations/diseases	-
166	2018	A pilot study of a psychologically informed physiotherapy for people awaiting knee replacement	No	Other populations/diseases	Other populations/diseases	-
167	2018	Better Knee, Better Me: effectiveness of two scalable health care interventions supporting self-m	No	Without intervention/comparator	Without intervention/comparator	-
168	2018	Biofeedback-assisted exercise in the rehabilitation of patients after total knee arthroplasty Ã¢â¬â	No	Other populations/diseases	Other populations/diseases	-
169	2018	Coaching and Exercise for Better Walking (ComeBACK) trial	No	Without intervention/comparator	Without intervention/comparator	-
170	2018	DICKENS - A randomised controlled trial of diacerein to treat knee osteoarthritis with effusion-s	No	Without intervention/comparator	Without intervention/comparator	-
171	2018	Evaluating the effects of tourniquet use in total knee knee arthroplasty	No	Other populations/diseases	Other populations/diseases	-
172	2018	Exercise-therapy following anterior cruciate ligament reconstruction: a pilot randomised clinical	No	Other populations/diseases	Other populations/diseases	-
173	2018	My Knee Exercise: a 6 month electronically delivered intervention to support self-management f	No	Without intervention/comparator	Other populations/diseases	Without intervention/c

174	2018	Study of ZYN002 (cannabidiol gel) in 40 Healthy Volunteers	No	Without intervention/comparator	Without intervention/comparator	-
175	2018	This study looks into the effects of FDY-5301 in relation to skeletal muscle loss after full knee re	No	Without intervention/comparator	Other populations/diseases	Other populations/dis
176	2018	WALK - A pilot randomised controlled trial evaluating community walking for knee osteoarthrit	No	Without intervention/comparator	Without intervention/comparator	-
177	2019	Effectiveness of an expert-moderated peer-to-peer online support group versus an information we	No	Without intervention/comparator	Without intervention/comparator	-
178	2019	Exercise for hip osteoarthritis symptoms: the PHOENIX study	No	Without intervention/comparator	Without intervention/comparator	-
179	2019	Exercise-therapy and education for knee osteoarthritis: comparison of telerehabilitation with face	No	Without intervention/comparator	Without intervention/comparator	-
180	2019	Face-to-face physiotherapy compared to a supported home exercise program for the managemen	No	Without intervention/comparator	Without intervention/comparator	-
181	2019	Investigating an innovative means of delivering musculoskeletal primary healthcare to improve p	No	Without intervention/comparator	Without intervention/comparator	-
182	2019	Novel exercise to maintain bone strength in postmenopausal women undergoing weight loss: the	No	Other populations/diseases	Other populations/diseases	-
183	2019	Testosterone to treat men with painful osteoarthritis of the knee	No	Without intervention/comparator	Without intervention/comparator	-
184	2019	The PEAK Study: physiotherapy, Exercise and physical Activity for Knee osteoarthritis	No	Without intervention/comparator	Without intervention/comparator	-
185	2020	Comparing effects of an online exercise plus education program to online education only in peop	No	Without intervention/comparator	Without intervention/comparator	-
186	2011	Comparative study of five therapeutic modalities applied to degenerative osteoarticular conditior	No	Without intervention/comparator	Discussion	Without intervention/c
187	2019	Ecological momentary assessment of using food to soothe during infancy in the INSIGHT trial	No	Other populations/diseases	Other populations/diseases	-
188	2019	A novel, hybrid, single- and multi-site clinical trial design for CLN3 disease, an ultra-rare lysoso	No	Other populations/diseases	Other populations/diseases	-
189	2012	Strengthening and stretching for rheumatoid arthritis of the hand (SARAH): design of a randomi	No	Without intervention/comparator	Without intervention/comparator	-
190	2019	The Osteoarthritis Thumb Therapy (OTTER) II Trial: a study protocol for a three-arm multi-cent	No	Without intervention/comparator	Without intervention/comparator	-
191	2005	Transcutaneous electrical nerve stimulation and interferential current combined with exercise for	No	Without intervention/comparator	Without intervention/comparator	-
192	2019	Squatting versus squatting with hip adduction in management of patellofemoral osteoarthritis: a	No	Without intervention/comparator	Without intervention/comparator	-
193	2016	In patients with rheumatoid arthritis in clinical remission undergoing treatment tapering tenosyn	No	Without intervention/comparator	Without intervention/comparator	-
194	2018	A clinicaltrial comparing functional outcomes inmedial compartment OA knee patients treated v	No	Without intervention/comparator	Discussion	Without intervention/c
195	2013	Effects of neuromuscular training (NEMEX-TJR) on patient-reported outcomes and physical fur	No	Without intervention/comparator	Without intervention/comparator	-
196	2019	Gonarthrosis concurrent with chronic venous insufficiency: a new look at therapy	No	Other populations/diseases	Other populations/diseases	-
197	2009	Change of quality of life due to exercise training in knee osteoarthritis: sF-36 and Womac	No	Without intervention/comparator	Without intervention/comparator	-
198	2019	Evaluation of the Interaction Model of Client Health Behavior-based multifaceted intervention o	No	Without intervention/comparator	Without intervention/comparator	-
199	2018	Effects of mediterranean diet and physical therapy in early stages of oligoarticular juvenile idiop	No	Other populations/diseases	Without intervention/comparator	Other populations/dis
200	2019	Knee osteoarthritis antecipates redutions in muscle mass attenuation and muscle strength since tl	No	Without intervention/comparator	Without intervention/comparator	-
201	2020	Is a periodized circuit training delivered by telerehabilitation effective for patients with knee oste	No	Without intervention/comparator	Without intervention/comparator	-
202	2014	A pilot randomized controlled trial of a tailored smoking cessation intervention for rheumatoid a	No	Without intervention/comparator	Without intervention/comparator	-
203	2017	Efficacy of a Rheumatoid Arthritis-Specific Smoking Cessation Program: a Randomized Contro	No	Without intervention/comparator	Without intervention/comparator	-
204	2018	Exploring perceptions of a rheumatoid arthritis-specific smoking cessation programme	No	Without intervention/comparator	Without intervention/comparator	-
205	2018	A protocol for a multicentre, randomised, double-blind, placebo-controlled trial to compare the e	No	Without intervention/comparator	Without intervention/comparator	-
206	2017	Efficacy of platelet rich plasma (prp) in severe knee osteoarthritis: can PRP injections delayarthr	No	Without intervention/comparator	Without intervention/comparator	-
207	2018	Efficacy of platelet-rich plasma administration in patients with severe knee osteoarthritis: can pla	No	Without intervention/comparator	Without intervention/comparator	-
208	2016	Intensive supervision of rehabilitation programme improves balance and functionality in the sho	No	Other populations/diseases	Other populations/diseases	-
209	2015	Matrix-induced autologous mesenchymal stem cell implantation versus matrix-induced autologo	No	Other populations/diseases	Other populations/diseases	-
210	2012	Efficacy of electromyographic biofeedback and electrical stimulation following arthroscopic par	No	Other populations/diseases	Other populations/diseases	-
211	2017	The efficacy of peloid therapy in management of hand osteoarthritis	No	Without intervention/comparator	Without intervention/comparator	-
212	2018	Short-term efficacy of paraffin therapy and home-based exercise programs in the treatment of sy	No	Without intervention/comparator	Without intervention/comparator	-
213	2010	Does short-wave diathermy increase the effectiveness of isokinetic exercise on pain, function, kr	No	Without intervention/comparator	Without intervention/comparator	-
214	2013	The efficacy of low level laser therapy in knee osteorthritis	No	Without intervention/comparator	Without intervention/comparator	-
215	2014	Efficacy of low-level laser therapy applied at acupuncture points in knee osteoarthritis: a random	No	Without intervention/comparator	Without intervention/comparator	-
216	2020	Cross-Cultural Adaptation and Validation of the Arabic Version of the Intermittent and Constant	No	Without intervention/comparator	Without intervention/comparator	-
217	2016	Efficacy of high intensity laser therapy on bone mineral density and fracture risk in male with os	No	Without intervention/comparator	Other populations/diseases	Other populations/dis

218	2017	Efficacy of pulsed Nd: YAG laser in the treatment of patients with knee osteoarthritis: a random	No	Without intervention/comparator	Without intervention/comparator	-
219	2017	Effect of aquatic physical therapy on pain perception, functional capacity and quality of life in o	No	Without intervention/comparator	Without intervention/comparator	-
220	2016	Effects of proprioceptive exercises on ankle dynamic joint stiffness	No	Other populations/diseases	Other populations/diseases	-
221	2016	Effectiveness of movement imagery on hip abductors muscle strength: results from a randomisec	No	Without intervention/comparator	Without intervention/comparator	-
222	2018	The effect of eicosapentaenoic and docosahexaenoic acids on physical function, exercise, and jo	No	Other populations/diseases	Other populations/diseases	-
223	2019	Geotherapy combined with kinesiotherapy is efficient in reducing pain in patients with osteoarth	No	Without intervention/comparator	Without intervention/comparator	-
224	2012	Efficacy of low level laser therapy associated with exercises in knee osteoarthritis: a randomized	No	Without intervention/comparator	Without intervention/comparator	-
225	2018	Long-term results of a randomized, controlled, double-blind study of low-level laser therapy befo	No	Without intervention/comparator	Without intervention/comparator	-
226	2020	Efficacy of continuous and pulsed therapeutic ultrasound combined with exercises for knee osteo	No	Without intervention/comparator	Without intervention/comparator	-
227	2016	Effect of retro and forward walking on quadriceps muscle strength, pain, function, and mobility	No	Without intervention/comparator	Without intervention/comparator	-
228	2019	Effect of 6-week retro or forward walking program on pain, functional disability, quadriceps mu	No	Without intervention/comparator	Without intervention/comparator	-
229	2014	Comparing the effects of manual therapy versus electrophysical agents in the management of kn	No	Without intervention/comparator	Without intervention/comparator	-
230	2013	Aerobic exercise training improves cardiovascular risk in patients with rheumatoid arthritis	No	Without intervention/comparator	Without intervention/comparator	-
231	2016	Improved Function and Reduced Pain after Swimming and Cycling Training in Patients with Os	No	Without intervention/comparator	Without intervention/comparator	-
232	2019	The effect of mobilization with movement on pain and function in patients with knee osteoarthri	No	Without intervention/comparator	Without intervention/comparator	-
233	2018	The effect of siwan therapy in management of patients with rheumatoid arthritis: a single blind r	No	Without intervention/comparator	Without intervention/comparator	-
234	2017	Physical therapy Vs. Internet-based exercise training for patients with knee osteoarthritis: results	No	Without intervention/comparator	Without intervention/comparator	-
235	2013	Group physical therapy for veterans with knee osteoarthritis: study design and methodology	No	Without intervention/comparator	Without intervention/comparator	-
236	2016	Group Versus Individual Physical Therapy for Veterans With Knee Osteoarthritis: randomized C	No	Without intervention/comparator	Without intervention/comparator	-
237	2017	Patient, Provider, and Combined Interventions for Managing Osteoarthritis in Primary Care: a C	No	Without intervention/comparator	Without intervention/comparator	-
238	2018	Associations of number of physical therapy visits and frequency of use of an internet-based exer	No	Without intervention/comparator	Without intervention/comparator	-
239	2018	Identifying subgroups of patients with differential benefit from physical therapy or internet-base	No	Without intervention/comparator	Without intervention/comparator	-
240	2018	Physical therapy vs internet-based exercise training for patients with knee osteoarthritis: results o	No	Without intervention/comparator	Without intervention/comparator	-
241	2019	STepped exercise program for patients with knee OsteoArthritis (STEP-KOA): protocol for a ran	No	Without intervention/comparator	Without intervention/comparator	-
242	2018	More than my RA: a randomized trial investigating body image improvement among women wit	No	Without intervention/comparator	Without intervention/comparator	-
243	2018	Gluteal tendinopathy and hip osteoarthritis: different pathologies, different hip biomechanics	No	Without intervention/comparator	Without intervention/comparator	-
244	2018	Dose-response relationship between neuromuscular electrical stimulation and muscle function in	No	Without intervention/comparator	Without intervention/comparator	-
245	2018	Effect of changes in physical activity on cartilage degradation in knee osteoarthritis	No	Without intervention/comparator	Without intervention/comparator	-
246	2019	Dose-Response Relationship Between Neuromuscular Electrical Stimulation and Muscle Functio	No	Without intervention/comparator	Without intervention/comparator	-
247	2011	Associations between changes in physical function and physical activity in response to an exerci	No	Without intervention/comparator	Without intervention/comparator	-
248	2012	The effect of progressive strengthening programs on function and gait mechanics after unilateral	No	Without intervention/comparator	Without intervention/comparator	-
249	2018	Low-level laser therapy and interferential current in patients with knee osteoarthritis: a randomiz	No	Without intervention/comparator	Without intervention/comparator	-
250	2020	The effect of physical therapy and rehabilitation modalities on sleep quality in patients with prim	No	Without intervention/comparator	Without intervention/comparator	-
251	2010	Effects of TENS on pain, disabiliy, quality of life and depression in patients with knee osteoarth	No	Without intervention/comparator	Without intervention/comparator	-
252	2018	Osteopathic manipulative treatment improves function and relieves pain in knee osteoarthritis: a	No	Without intervention/comparator	Without intervention/comparator	-
253	2020	Exergaming as an Additional Tool in Rehabilitation of Young Patients with Rheumatoid Arthriti	No	Without intervention/comparator	Without intervention/comparator	-
254	2015	Alpine Skiing With total knee ArthroPlasty (ASWAP): physical self-concept, pain, and life satis	No	Without intervention/comparator	Without intervention/comparator	-
255	2017	Improving walking ability in degenerative lumbar spinal stenosis: a randomized trial comparing	No	Other populations/diseases	Other populations/diseases	-
256	2017	Development and validation of a flourescence optical imaging rheumatoid arthritis scoring syste	No	Without intervention/comparator	Without intervention/comparator	-
257	2013	Effects of baduanjin exercise on knee osteoarthritis: a one-year study	No	Without intervention/comparator	Without intervention/comparator	-
258	2014	Efficacy of kinesio taping on isokinetic quadriceps torque in knee osteoarthritis: a double blinde	No	Without intervention/comparator	Without intervention/comparator	-
259	1999	Changes in muscle function after knee arthroplasty	No	Without intervention/comparator	Without intervention/comparator	-
260	2003	Comparison of Rheumatoid Arthritis Clinical Trial Outcome Measures: a Yesulation Study	No	Without intervention/comparator	Without intervention/comparator	-
261	2019	Fall Risk and Utilization of Balance Training for Adults With Symptomatic Knee Osteoarthritis:	No	Without intervention/comparator	Without intervention/comparator	-

262	2006	Serum levels of Cartilage Oligomeric Matrix Protein (COMP) increase temporarily after physica	No	Without intervention/comparator	Without intervention/comparator	-
263	2018	Intense aerobic and resistance exercise reduces the frequency of peripheral blood regulatory cell	No	Without intervention/comparator	Without intervention/comparator	-
264	2020	Peer-Delivered Cognitive Behavioral Training to Improve Functioning in Patients With Diabetes	No	Other populations/diseases	Other populations/diseases	-
265	2018	Does reduction in mechanical knee joint loading explain the beneficial effects of weight loss in c	No	Without intervention/comparator	Without intervention/comparator	-
266	2013	Long-term benefits of radon spa therapy in rheumatic diseases: results of the randomised, multi-	No	Without intervention/comparator	Without intervention/comparator	-
267	2017	Erratum: does occupational therapy reduce the need for surgery in carpometacarpal osteoarthritis	No	Without intervention/comparator	Without intervention/comparator	-
268	2019	A Tailored Behavioral Intervention to Promote Adherence to the DASH Diet	No	Other populations/diseases	Without intervention/comparator	Without intervention/c
269	2019	Structured total knee replacement rehabilitation programme and quality of life following two dif	No	Without intervention/comparator	Other populations/diseases	Without intervention/c
270	2018	Effectiveness of self-management program on arthritis symptoms among older women: a random	No	Without intervention/comparator	Without intervention/comparator	-
271	2011	Effectiveness of electromyographic biofeedback training on quadriceps muscle strength in osteo	No	Without intervention/comparator	Without intervention/comparator	-
272	2013	Effect of gender on strength gains after isometric exercise coupled with electromyographic biofe	No	Without intervention/comparator	Without intervention/comparator	-
273	2009	Home stretching exercise is effective for improving knee range of motion and gait in patients wi	No	Without intervention/comparator	Without intervention/comparator	-
274	2017	A comparative study on the effectiveness of neuromuscular training and proprioceptive exercises	No	Without intervention/comparator	Without intervention/comparator	-
275	2017	Effectiveness of stabilization exercises and conventional physiotherapy in subjects with knee ost	No	Without intervention/comparator	Without intervention/comparator	-
276	2017	The effect of thumb splinting on thenar muscles atrophy, pain, and function in subjects with thu	No	Without intervention/comparator	Without intervention/comparator	-
277	2018	Pain and sensitization after total knee replacement or nonsurgical treatment in patients with knee	No	Without intervention/comparator	Other populations/diseases	Without intervention/c
278	2015	Investigation of the effect of exercise on functional status in patients with osteoarthritis of the kn	No	Without intervention/comparator	Without intervention/comparator	-
279	2014	A combination of Scutellaria baicalensis and Acacia catechu extracts for short-term symptomati	No	Without intervention/comparator	Without intervention/comparator	-
280	2012	Inhalation therapy of calcitonin relieves osteoarthritis of the knee	No	Without intervention/comparator	Without intervention/comparator	-
281	2015	Comparison of the symptomatic and chondroprotective effects of glucosamine sulphate and exer	No	Without intervention/comparator	Without intervention/comparator	-
282	2017	The effect of task-oriented training with video-based games on activity performance and particip	No	Other populations/diseases	Without intervention/comparator	Other populations/dis
283	2019	Effects of Video Games-Based Task-Oriented Activity Training (Xbox 360 Kinect) on Activity I	No	Other populations/diseases	Without intervention/comparator	Other populations/dis
284	2015	Design and acceptance of Rheumates@Work, a combined internet-based and in person instructi	No	Other populations/diseases	Without intervention/comparator	Other populations/dis
285	2017	Internet Program for Physical Activity and Exercise Capacity in Children With Juvenile Idiopath	No	Other populations/diseases	Without intervention/comparator	Other populations/dis
286	2008	Fall risk in older adults with hip osteoarthritis: decreasing risk through education and aquatic ex	No	Without intervention/comparator	Without intervention/comparator	-
287	2010	The effect of aquatic exercise and education on lowering fall risk in older adults with hip osteoar	No	Without intervention/comparator	Without intervention/comparator	-
288	2019	Effect of Denosumab on Femoral Periprosthetic BMD and Early Femoral Stem Subsidence in Po	No	Other populations/diseases	Other populations/diseases	-
289	2011	ACCF/AHA 2011 expert consensus document on hypertension in the elderly: A Report of the Ar	No	Other populations/diseases	Without intervention/comparator	Other populations/dis
290	2017	Analgesic potential of NEO6860, a modality selective TRPV1 antagonist, in osteoarthritis knee p	No	Without intervention/comparator	Without intervention/comparator	-
291	2017	Comparison of group-based outpatient physiotherapy with usual care after total knee replacemen	No	Other populations/diseases	Other populations/diseases	-
292	2017	Report of the Clinical and Functional Primary Outcomes in Men of the ACL-SPORTS Trial: Yes	No	Other populations/diseases	Other populations/diseases	-
293	2016	Resistance of articular cartilage photochemical bonds to interleukin-1-induced degradation	No	Without intervention/comparator	Without intervention/comparator	-
294	2017	The effect of combined training (hip abductor and external rotators strengthening + balance) on p	No	Other populations/diseases	Other populations/diseases	-
295	2010	The use of a composite femoral component in primary tha: a prospective, randomized controlled	No	Without intervention/comparator	Without intervention/comparator	-
296	2013	Efficacy and tolerability of celecoxib in patients with osteoarthritis who previously did not respo	No	Without intervention/comparator	Without intervention/comparator	-
297	2019	Additional benefit of local infiltration of analgesia to femoral nerve block in total knee arthropla	No	Other populations/diseases	Other populations/diseases	-
298	2020	The effect of Total resistance exercise vs. aquatic training on self-reported knee instability, pain,	No	Without intervention/comparator	Without intervention/comparator	-
299	2011	Implementing one standardized rehabilitation protocol following autologous chondrocyte implan	No	Other populations/diseases	Other populations/diseases	-
300	2012	Comparison of the efficacy of transcutaneous electrical nerve stimulation, interferential currents,	No	Without intervention/comparator	Without intervention/comparator	-
301	2019	A novel behavioural INTErvention to REduce Sitting Time in older adults undergoing orthopaed	No	Other populations/diseases	Other populations/diseases	-
302	2020	A novel behavioural INTErvention to REduce Sitting Time in older adults undergoing orthopaed	No	Without intervention/comparator	Without intervention/comparator	-
303	2017	Formal Physical Therapy After Total Hip Arthroplasty Is Not Required: a Randomized Controlle	No	Without intervention/comparator	Other populations/diseases	Other populations/dis
304	2011	The effect of adding whole-body vibration to squat training on the functional performance and s	No	Without intervention/comparator	Without intervention/comparator	-
305	2014	Pain and recovery after total knee arthroplasty: a 12-month follow-up after a prospective random	No	Other populations/diseases	Other populations/diseases	-

306	2011	Does electric stimulation of the vastus medialis muscle influence rehabilitation after total knee re	No	Other populations/diseases	Other populations/diseases	-
307	2013	Is there an effective way to prescribe a home-based exercise program in patients with knee osteo	No	Without intervention/comparator	Without intervention/comparator	-
308	2017	Clinical outcomes of kinesio taping applied in patients with knee osteoarthritis: a randomized co	No	Without intervention/comparator	Without intervention/comparator	-
309	2018	HPR effects of short-term neuromuscular electrical stimulation on pain, quadriceps muscle stren	No	Without intervention/comparator	Without intervention/comparator	-
310	2019	Mobile-phone-based home exercise training program in patients with knee osteoarthritis	No	Without intervention/comparator	Without intervention/comparator	-
311	2020	The Effects of Tourniquet Application in Total Knee Arthroplasty on the Recovery of Thigh Mu	No	Other populations/diseases	Other populations/diseases	-
312	2014	Effects of exercise on body composition, cardiovascular fitness, muscle strength, and cognition i	No	Without intervention/comparator	Without intervention/comparator	-
313	2020	Benefits of exercise in patients with rheumatoid arthritis: a randomized controlled trial of a patie	No	Without intervention/comparator	Without intervention/comparator	-
314	2016	A Water Rehabilitation Program in Patients with Hip Osteoarthritis Before and After Total Hip I	No	Without intervention/comparator	Other populations/diseases	Without intervention/c
315	2014	The Effectiveness of Aquatic Exercises in the Treatment of Rheumatoid Arthritis	No	Without intervention/comparator	Without intervention/comparator	-
316	2019	Comparison of Interactive Education Versus Fluorescent Concretization on Hand Hygiene Comp	No	Other populations/diseases	Other populations/diseases	-
317	2013	Efficacy of strength and aerobic exercise on patient-reported outcomes and structural changes in	No	Without intervention/comparator	Without intervention/comparator	-
318	2014	Exercises with telephone follow-up versus usual care in people with hand osteoarthritis: a rando	No	Without intervention/comparator	Without intervention/comparator	-
319	2014	Is postoperative exercise therapy necessary in patients with degenerative meniscus? A randomize	No	Other populations/diseases	Other populations/diseases	-
320	2017	Effects of medical exercise therapy in patients with hip osteoarthritis: a randomized controlled tr	No	Without intervention/comparator	Without intervention/comparator	-
321	2014	Exercise programme with telephone follow-up for people with hand osteoarthritis - protocol for a	No	Without intervention/comparator	Without intervention/comparator	-
322	2014	Limited effects of exercises in people with hand osteoarthritis: results from a randomized control	No	Without intervention/comparator	Without intervention/comparator	-
323	2015	Implementing international osteoarthritis treatment guidelines in primary health care: study proto	No	Without intervention/comparator	Without intervention/comparator	-
324	2019	Implementing a structured model for osteoarthritis care in primary healthcare: a stepped-wedge c	No	Without intervention/comparator	Without intervention/comparator	-
325	2019	A comparison of ultrasound guided corticosteroid injection versus ozone injection in grade 3 kne	No	Without intervention/comparator	Without intervention/comparator	-
326	2011	Effectiveness of leech therapy in chronic lateral epicondylitis: a randomized controlled trial	No	Other populations/diseases	Other populations/diseases	-
327	2017	Testosterone supplementation increases lean mass in men undergoing anterior cruciate ligament	No	Other populations/diseases	Other populations/diseases	-
328	2014	Predicting functional performance and range of motion outcomes after total knee arthroplasty	No	Other populations/diseases	Other populations/diseases	-
329	2017	Early High-Intensity Versus Low-Intensity Rehabilitation After Total Knee Arthroplasty: a Rand	No	Other populations/diseases	Other populations/diseases	-
330	2020	Movement pattern biofeedback training after total knee arthroplasty: randomized clinical trial pr	No	Other populations/diseases	Other populations/diseases	-
331	2009	Comparison of therapeutic effects of sodium hyaluronate and corticosteroid injections on trapezi	No	Without intervention/comparator	Without intervention/comparator	-
332	2014	Improvement of outcomes with nonconcurrent strength and cardiovascular-endurance rehabilitat	No	Other populations/diseases	Other populations/diseases	-
333	2020	Does Blood Flow Restriction Therapy in Patients Older Than Age 50 Result in Muscle Hypertro	No	Review/Systematic Review/Meta-Analy	Review/Systematic Review/Meta-Analys	-
334	2013	Automated telephone-linked communication: a novel approach to enhance long-term adherence	No	Without intervention/comparator	Without intervention/comparator	-
335	2016	A randomized trial of automated telephone-linked communication to improve exercise adherenc	No	Without intervention/comparator	Without intervention/comparator	-
336	2019	Efficacy of Computer-based Telephone Counseling on Long-term Adherence to Strength Trainin	No	Without intervention/comparator	Without intervention/comparator	-
337	2019	Does the presence of self-reported knee instability impact the response to walking surface transla	No	Without intervention/comparator	Without intervention/comparator	-
338	2012	Performance of a multi-biomarker score measuring rheumatoid arthritis disease activity in the C	No	Without intervention/comparator	Without intervention/comparator	-
339	2019	Exercise-induced pain changes associate with changes in muscle perfusion in knee osteoarthritis	No	Without intervention/comparator	Without intervention/comparator	-
340	2014	Self-report treatment profile: healthcare utilization among knee osteoarthritis and low back pain	No	Without intervention/comparator	Without intervention/comparator	-
341	2013	Comparison of custom-made and prefabricated neoprene splinting in patients with the first carp	No	Without intervention/comparator	Without intervention/comparator	-
342	2020	Adverse events associated with analgesics used for osteoarthritis pain: analysis of post-marketin	No	Without intervention/comparator	Without intervention/comparator	-
343	2018	Effect of therapeutic exercise on knee osteoarthritis after intra-articular injection of botulinum to	No	Without intervention/comparator	Without intervention/comparator	-
344	2019	Blood Flow-Restricted Training for Lower Extremity Muscle Weakness due to Knee Pathology:	No	Review/Systematic Review/Meta-Analy	Review/Systematic Review/Meta-Analys	-
345	2016	Jumping into the deep-end: results from a pilot impact evaluation of a community-based aquatic	No	Other populations/diseases	Without intervention/comparator	Without intervention/c
346	2013	Recovery of function following hip resurfacing: a randomised controlled trial comparing a tailor	No	Without intervention/comparator	Without intervention/comparator	-
347	2013	Recovery of function following hip resurfacing arthroplasty: a randomized controlled trial compa	No	Without intervention/comparator	Without intervention/comparator	-
348	2015	A systematic review of the use of spinal orthoses in the management of vertebral osteoporosis an	No	Review/Systematic Review/Meta-Analy	Review/Systematic Review/Meta-Analys	-
349	2015	Physiotherapy rehabilitation for osteoporotic vertebral fracture (prove): study protocol for an ada	No	Other populations/diseases	Other populations/diseases	-

350	2010	A randomized trial of three psychosocial treatments for the symptoms of rheumatoid arthritis	No	Without intervention/comparator	Without intervention/comparator	-
351	2017	Relationship between weight loss in obese knee osteoarthritis patients and serum biomarkers of c	No	Without intervention/comparator	Without intervention/comparator	-
352	2001	Pool exercise therapy of rheumatoid arthritis	No	Without intervention/comparator	Without intervention/comparator	-
353	2016	A Standardized "Rescue" Exercise Program for Symptomatic Flare-up of Knee Osteoarthritis: de	No	Without intervention/comparator	Without intervention/comparator	-
354	2017	HPR assessment of local disease activity after an intensive hand exercise program in patients wit	No	Without intervention/comparator	Without intervention/comparator	-
355	2013	Measurement of therapeutic effect of ultrasound on knee osteoarthritis; double blind study	No	Without intervention/comparator	Without intervention/comparator	-
356	2019	Arthroscopic repair of chronic lateral ankle instability	No	Without intervention/comparator	Other populations/diseases	Other populations/dis
357	2016	Are Pulley Exercises Initiated 6 Weeks After Rotator Cuff Repair a Safe and Effective Rehabilit	No	Other populations/diseases	Without intervention/comparator	Other populations/dis
358	2016	Walking is a Feasible Physical Activity for People with Rheumatoid Arthritis: a Feasibility Rand	No	Without intervention/comparator	Without intervention/comparator	-
359	2011	Effectiveness of leech therapy in chronic lateral epicondylitis: a randomized controlled trial	No	Other populations/diseases	Other populations/diseases	-
360	2012	Exercise therapy in the management of upper limb dysfunction in rheumatoid arthritis	No	Without intervention/comparator	Without intervention/comparator	-
361	2011	Feasibility of an exercise-based rehabilitation programme for chronic hip pain	No	Without intervention/comparator	Without intervention/comparator	-
362	2012	A brief exercise and self management programme improves upper limb disability in people with	No	Without intervention/comparator	Without intervention/comparator	-
363	2015	Economic evaluation of a brief education, self-management and upper limb exercise training pro	No	Without intervention/comparator	Without intervention/comparator	-
364	2017	Clinical relevance commentary in response to: effectiveness of a fine motor skills rehabilitation p	No	Without intervention/comparator	Without intervention/comparator	-
365	2013	Evaluation of the impact of a 6-month training by whole body vibration on the risk of falls amon	No	Other populations/diseases	Other populations/diseases	-
366	2014	The independent and combined effects of intensive weight loss and exercise training on bone mi	No	Without intervention/comparator	Without intervention/comparator	-
367	2015	Effects of total and regional fat loss on plasma CRP and IL-6 in overweight and obese, older adu	No	Without intervention/comparator	Without intervention/comparator	-
368	2017	Brief high intensity exercise improvesbone, posture and functional risk factors for falling in post	No	Without intervention/comparator	Without intervention/comparator	-
369	2019	Sports Therapy Interventions Following Total Hip Replacement	No	Other populations/diseases	Other populations/diseases	-
370	2013	A prospective randomized comparison of neoprene vs thermoplast hand-based thumb spica splin	No	Other populations/diseases	Other populations/diseases	-
371	2017	Exercise in knee osteoarthritis: do treatment outcomes relate to bone marrow lesions? A randomi	No	Without intervention/comparator	Without intervention/comparator	-
372	2014	Effectiveness of exercise in knee osteoarthritis: should we take a patient's health status into acco	No	Without intervention/comparator	Without intervention/comparator	-
373	2015	Exercise in knee osteoarthritis - preliminary findings: exercise-induced pain and health status dif	No	Without intervention/comparator	Without intervention/comparator	-
374	2017	Exercise-induced pain and health status differ between drop-outs and retainers of an exercise pro	No	Without intervention/comparator	Without intervention/comparator	-
375	2015	Immediate effects of different elastic taping techniques on pain, isokinetic muscle strength, prop	No	Without intervention/comparator	Without intervention/comparator	-
376	2010	Efficacy and safety of MerivaÂ®, a curcumin-phosphatidylcholine complex, during extended ad	No	Without intervention/comparator	Without intervention/comparator	-
377	1998	A randomized controlled trial to evaluate the efficacy of community based physical therapy in th	No	Without intervention/comparator	Without intervention/comparator	-
378	2014	The effects of combining viscosupplementation and knee bracing on pain reduction and increase	No	Without intervention/comparator	Without intervention/comparator	-
379	2019	Abductor muscle strengthening in THA patients operated with minimally invasive anterolateral a	No	Other populations/diseases	Other populations/diseases	-
380	2010	An investigation of motor learning during side-step cutting: design of a randomised controlled tr	No	Without intervention/comparator	Without intervention/comparator	-
381	2018	Effects of isokinetic, isometric, and aerobic exercises on clinical variables and knee cartilage vol	No	Without intervention/comparator	Without intervention/comparator	-
382	2013	Comparison of neuromuscular and quadriceps strengthening exercise in people with medial knee	No	Without intervention/comparator	Without intervention/comparator	-
383	2013	Physiotherapist-delivered exercise and pain coping skills training is more effective than either in	No	Without intervention/comparator	Without intervention/comparator	-
384	2013	Type of exercise and presence of varus thrust influences pain outcomes in people with medial kn	No	Without intervention/comparator	Without intervention/comparator	-
385	2016	Telephone coaching to enhance a physiotherapistprescribed home-based physical activity progra	No	Without intervention/comparator	Without intervention/comparator	-
386	2020	Personalized exercise therapy for people with knee osteoarthritis and obesity: a randomized contr	No	Without intervention/comparator	Without intervention/comparator	-
387	2005	Effect of experimentally induced knee pain on standing balance in healthy older individuals	No	Without intervention/comparator	Without intervention/comparator	-
388	2010	Efficacy of a multimodal physiotherapy treatment program for hip osteoarthritis: a randomised p	No	Without intervention/comparator	Without intervention/comparator	-
389	2010	Hip strengthening reduces symptoms but not knee load in people with medial knee osteoarthritis	No	Without intervention/comparator	Without intervention/comparator	-
390	2011	Comparison of neuromuscular and quadriceps strengthening exercise in the treatment of varus m	No	Without intervention/comparator	Without intervention/comparator	-
391	2011	Lateral wedge insoles for medial knee osteoarthritis: 12 month randomised controlled trial	No	Without intervention/comparator	Without intervention/comparator	-
392	2012	A physiotherapist-delivered integrated exercise and pain coping skills training intervention for ir	No	Without intervention/comparator	Without intervention/comparator	-
393	2012	Addition of telephone coaching to a physiotherapist-delivered physical activity program in peopl	No	Without intervention/comparator	Without intervention/comparator	-

394	2014	Effect of physical therapy on pain and function in patients with hip osteoarthritis: a randomized	No	Without intervention/comparator	Without intervention/comparator	-
395	2014	Effects of two physiotherapy booster sessions on outcomes with home exercise in people with kr	No	Without intervention/comparator	Without intervention/comparator	-
396	2014	Neuromuscular versus quadriceps strengthening exercise in patients with medial knee osteoarthr	No	Without intervention/comparator	Without intervention/comparator	-
397	2014	Physical therapy for hip osteoarthritis: randomised, placebo-controlled trial	No	Without intervention/comparator	Without intervention/comparator	-
398	2014	Physiotherapist-delivered exercise and pain coping skills training is more effective than either in	No	Without intervention/comparator	Without intervention/comparator	-
399	2015	Effects of Adding an Internet-Based Pain Coping Skills Training Protocol to a Standardized Edu	No	Without intervention/comparator	Without intervention/comparator	-
400	2015	Influence of Biomechanical Characteristics on Pain and Function Outcomes From Exercise in M	No	Without intervention/comparator	Without intervention/comparator	-
401	2016	Physical Therapist-Delivered Pain Coping Skills Training and Exercise for Knee Osteoarthritis: ı	No	Without intervention/comparator	Without intervention/comparator	-
402	2017	Effectiveness of an Internet-Delivered Exercise and Pain-Coping Skills Training Intervention for	No	Without intervention/comparator	Without intervention/comparator	-
403	2017	Telephone Coaching to Enhance a Home-Based Physical Activity Program for Knee Osteoarthrit	No	Without intervention/comparator	Without intervention/comparator	-
404	2018	Effects of internet-based pain coping skills training before home exercise for individuals with hi	No	Without intervention/comparator	Without intervention/comparator	-
405	2019	Comparison of weight bearing functional exercise and non-weight bearing quadriceps strengther	No	Without intervention/comparator	Without intervention/comparator	-
406	2020	Better Knee, Better MeÃ¢â‚¬Â¢: effectiveness of two scalable health care interventions supporting	No	Without intervention/comparator	Without intervention/comparator	-
407	2020	What type of exercise is most effective for people with knee osteoarthritis and co-morbid obesity	No	Without intervention/comparator	Without intervention/comparator	-
408	2020	Development of osteoarthritis in patients with degenerative meniscal tears treated with exercise t	No	Without intervention/comparator	Without intervention/comparator	-
409	2020	Knee osteoarthritis development five years following arthroscopic partial meniscectomy or exerc	No	Without intervention/comparator	Without intervention/comparator	-
410	2012	Sex differences in quadriceps strength in OA	No	Without intervention/comparator	Without intervention/comparator	-
411	2015	Prospective multi-centre study on a composite ceramic femoral component in total knee arthropl	No	Without intervention/comparator	Without intervention/comparator	-
412	2016	Vertebral fractures in an osteoporotic perspective, symptoms and what to do	No	Without intervention/comparator	Without intervention/comparator	-
413	1996	Multiple-outcome meta-analysis of clinical trials	No	Other topics	Other topics	-
414	1991	The use of music as purposeful activity: a preliminary investigation	No	Other populations/diseases	Other populations/diseases	-
415	2019	Effects of Virtual Exercise Rehabilitation In-Home Therapy Compared with Traditional Care Af	No	Without intervention/comparator	Without intervention/comparator	-
416	2011	Accelerated versus nonaccelerated rehabilitation after anterior cruciate ligament reconstruction: a	No	Without intervention/comparator	Without intervention/comparator	-
417	2010	The effect of a group education programme on pain and function through knowledge acquisition	No	Without intervention/comparator	Without intervention/comparator	-
418	2009	What is the "exercise prescription" for patients with knee osteoarthritis?	No	Without intervention/comparator	Without intervention/comparator	-
419	2010	The role of helplessness, outcome expectation for exercise and literacy in predicting disability ar	No	Without intervention/comparator	Without intervention/comparator	-
420	2017	Clinical and gait outcomes of novel pneumatic knee brace with extension assist	No	Without intervention/comparator	Without intervention/comparator	-
421	2014	Intra-rater reliability and agreement of muscle strength, power and functional performance measu	No	Without intervention/comparator	Without intervention/comparator	-
422	2014	Supervised strength training, NORDIC walking or unsupervised home based exercise in older pe	No	Without intervention/comparator	Without intervention/comparator	-
423	2016	Even in the long run nordic walking is superior to strength training and home based exercise for	No	Without intervention/comparator	Without intervention/comparator	-
424	2017	In hip osteoarthritis, Nordic Walking is superior to strength training and home-based exercise fo	No	Without intervention/comparator	Without intervention/comparator	-
425	2018	Exercise induced effects on muscle function and range of motion in patients with hip osteoarthri	No	Without intervention/comparator	Without intervention/comparator	-
426	2015	MRI volume and signal intensity of ACL graft predict clinical, functional, and patient-oriented c	No	Other populations/diseases	Other populations/diseases	-
427	2011	Randomized controlled trial of a home based exercise and balance training programme in elderly	No	Without intervention/comparator	Without intervention/comparator	-
428	2012	Effects of exercises on back pain, depression and quality of life to osteoporotic patients	No	Without intervention/comparator	Without intervention/comparator	-
429	2012	Efficacy of physical exercice in patients with osteoporosis	No	Without intervention/comparator	Without intervention/comparator	-
430	2013	Supervised and unsupervised rehabilitation after osteoporotic wrist fracture	No	Without intervention/comparator	Without intervention/comparator	-
431	2013	The effectiveness of combined exercises and therapeutic ultrasound to patients with knee osteoar	No	Without intervention/comparator	Without intervention/comparator	-
432	2016	Effects of Leg-Press Training With Moderate Vibration on Muscle Strength, Pain, and Function	No	Without intervention/comparator	Without intervention/comparator	-
433	2013	Improvement in some, but not all, surrogate measures of cardiovascular disease following intens	No	Without intervention/comparator	Without intervention/comparator	-
434	2012	Precompetition medical assessment of referees and assistant referees selected for the 2010 FIFA	No	Other populations/diseases	Other populations/diseases	-
435	1998	CPH-82 (Reumacon) versus auranofin (Ridaura): a 36-week study of their respective onset of ac	No	Without intervention/comparator	Without intervention/comparator	-
436	2020	Moderate weight bearing and minimal weight bearing exercise induce acute impact on collagen l	No	Without intervention/comparator	Without intervention/comparator	-
437	2010	Ginger (Zingiber officinale) reduces muscle pain caused by eccentric exercise	No	Other topics	Other topics	-

438	2014	Whole body and local muscle vibration reduce artificially induced quadriceps arthrogenic inhibi	No	Without intervention/comparator	Without intervention/comparator	-
439	2019	The effects of preoperative balance training on balance and functional outcome after total knee r	No	Other populations/diseases	Other populations/diseases	-
440	2019	A reliable method of determining glenohumeral offset in anatomic total shoulder arthroplasty	No	Other populations/diseases	Other populations/diseases	-
441	2011	Hand appearance as a patient motivation for surgery and a determinant of satisfaction with meta	No	Without intervention/comparator	Without intervention/comparator	-
442	2018	Effectiveness of proprioceptive neuromuscular facilitation patterns in comparison with general p	No	Without intervention/comparator	Without intervention/comparator	-
443	2016	The effect of adding whole body vibration training to strengthening training in the treatment of k	No	Without intervention/comparator	Without intervention/comparator	-
444	2018	Physical therapy and natural eggshell membrane in the management of osteoarthritic knee sympt	No	Without intervention/comparator	Without intervention/comparator	-
445	2010	A comparison of subvastus and midvastus approaches in minimally invasive total knee arthropla	No	Other populations/diseases	Other populations/diseases	-
446	2019	Acceptability of locallyâproduced ReadyâtoâUse Supplementary Food (RUSF) for children unde	No	Other populations/diseases	Other populations/diseases	-
447	2013	PReS-FINAL-2148: rheumates@work a cognitive behavioural internet based intervention promo	No	Other populations/diseases	Other populations/diseases	-
448	2017	Internet program for physical activity and exercise-capacity in children with Juvenile Idiopathic	No	Other populations/diseases	Other populations/diseases	-
449	2015	Effect of adapted physical activity on bone mineral density and life quality of postmenopausal w	No	Other populations/diseases	Other populations/diseases	-
450	2016	The impact of adapted kinesitherapy program on disability and back pain in women with postme	No	Other populations/diseases	Other populations/diseases	-
451	2013	Effectiveness of a web-based physical activity intervention in patients with knee and/or hip oste	No	Without intervention/comparator	Without intervention/comparator	-
452	2015	The association between psychological factors and physical activity levels in patients with knee	No	Without intervention/comparator	Without intervention/comparator	-
453	2013	Defining optimal nhs occupational therapy treatment, individualized splint and placebo splint fo	No	Without intervention/comparator	Without intervention/comparator	-
454	2016	Does receiving physical therapy for knee osteoarthritis impact downstream healthcare utilization	No	Without intervention/comparator	Without intervention/comparator	-
455	2018	Exercise, Manual Therapy, and Booster Sessions in Knee Osteoarthritis: cost-Effectiveness Anal	No	Without intervention/comparator	Without intervention/comparator	-
456	2018	Exercise on balance and function for knee osteoarthritis: a randomized controlled trial	No	Without intervention/comparator	Without intervention/comparator	-
457	2016	Knee Extensor Strength and Gait Characteristics After Minimally Invasive Unicondylar Knee Ar	No	Without intervention/comparator	Without intervention/comparator	-
**458**	**2018**	**Benefits of Resistance Training with Blood Flow Restriction in Knee Osteoarthritis**	**Yes**	**Full**	**Full**	**-**
459	2009	Functional improvement with hylan G-F 20 in patients with knee osteoarthritis	No	Without intervention/comparator	Without intervention/comparator	-
460	2018	Impact of a tailored activity counselling intervention during inpatient rehabilitation after knee an	No	Other populations/diseases	Other populations/diseases	-
461	2012	Full kinetic chain manual and manipulative therapy plus exercise compared with targeted manua	No	Without intervention/comparator	Without intervention/comparator	-
462	2011	Effects of a group-based exercise and educational program on physical performance and disease	No	Without intervention/comparator	Without intervention/comparator	-
463	2018	The effect of targeted exercise on knee-muscle function in patients with persistent hamstring def	No	Other populations/diseases	Other populations/diseases	-
464	2015	A yoga strengthening program designed to minimize the knee adduction moment for women wit	No	Without intervention/comparator	Without intervention/comparator	-
465	2014	High-intensity interval training on an aquatic treadmill in adults with osteoarthritis: effect on pai	No	Without intervention/comparator	Without intervention/comparator	-
466	2020	The benefits and harms of therapeutic exercise on physical and psychosocial outcomes in people	No	Review/Systematic Review/Meta-Analy	Review/Systematic Review/Meta-Analys	-
467	2016	Effect of deep slow breathing on pain-related variables in osteoarthritis	No	Without intervention/comparator	Without intervention/comparator	-
468	2019	A mixed-methods process evaluation of Family Navigation implementation for autism spectrum	No	Other populations/diseases	Other populations/diseases	-
469	2011	Treatment expectation for pain coping skills training: relationship to osteoarthritis patients' basel	No	Without intervention/comparator	Without intervention/comparator	-
470	2014	Nurse practitioners can effectively deliver pain coping skills training to osteoarthritis patients wi	No	Without intervention/comparator	Without intervention/comparator	-
471	2014	Two different sets of handexercises improved grip strength after eight weeks in patients with artl	No	Without intervention/comparator	Without intervention/comparator	-
472	2011	Effects of prehabilitation on early rehabilitation outcomes following total knee arthroplasty in pa	No	Without intervention/comparator	Without intervention/comparator	-
473	2000	Low level laser therapy for osteoarthritis and rheumatoid arthritis: a metaanalysis	No	Review/Systematic Review/Meta-Analy	Review/Systematic Review/Meta-Analys	-
474	2012	The implementation of a community-based aerobic walking program for mild to moderate knee	No	Without intervention/comparator	Without intervention/comparator	-
475	2012	The implementation of a community-based aerobic walking program for mild to moderate knee	No	Without intervention/comparator	Without intervention/comparator	-
476	2014	The knowledge-to-action cycle (KTAC): a Canadian model to guide the dissemination and the in	No	Without intervention/comparator	Without intervention/comparator	-
477	2012	Prehabilitation and quality of life three months after total knee arthroplasty: a pilot study	No	Other populations/diseases	Other populations/diseases	-
478	2014	Prehabilitation influences exercise-related psychological constructs such as self-efficacy and out	No	Without intervention/comparator	Without intervention/comparator	-
479	2012	Effects of home-based resistance training and neuromuscular electrical stimulation in knee osteo	No	Without intervention/comparator	Without intervention/comparator	-
480	2013	The immediate and long-term effects of a walking-skill program compared to usual physiotherap	No	Other populations/diseases	Other populations/diseases	-
481	2008	Evaluation of symptomatic slow-acting drugs in osteoarthritis using the GRADE system	No	Without intervention/comparator	Without intervention/comparator	-

482	2014	Can physical therapists deliver a pain coping skills program? An examination of training process	No	Other populations/diseases	Without intervention/comparator	Other populations/dis
483	2011	Immediate effect of the elastic knee sleeve use on individuals with osteoarthritis	No	Without intervention/comparator	Without intervention/comparator	-
**484**	**2016**	**Exercises with partial vascular occlusion in patients with knee osteoarthritis: a randomize**	**Yes**	**Full**	**Full**	**-**
485	2019	RESTORING KNEE EXTENSOR STRENGTH AFTER ANTERIOR CRUCIATE LIGAMENT	No	Other populations/diseases	Other populations/diseases	-
**486**	**2015**	**Kaatsu training to enhance physical function of older adults with knee osteoarthritis: desig**	**Yes**	**Full**	**Full**	**-**
487	2016	Use of an Anti-Gravity Treadmill for Early Postoperative Rehabilitation After Total Knee Replac	No	Other populations/diseases	Other populations/diseases	-
488	2010	Surgical compared with conservative treatment for acute nondisplaced or minimally displaced sc	No	Review/Systematic Review/Meta-Analys	Review/Systematic Review/Meta-Analys	-
489	2013	Platelet gels	No	Without intervention/comparator	Without intervention/comparator	-
490	2014	Acute resistance exercise and pressure pain sensitivity in knee osteoarthritis: a randomised cross	No	Without intervention/comparator	Without intervention/comparator	-
491	2018	An evaluation of trak physiotherapy self management intervention development and delivery for	No	Without intervention/comparator	Without intervention/comparator	-
492	2011	Piriformis syndrome in knee osteoarthritis patients after wearing rocker bottom shoes	No	Without intervention/comparator	Without intervention/comparator	-
493	2015	Effects of physical activity and anti-TNF-alpha disease control on lipid profile of rheumatoid art	No	Without intervention/comparator	Without intervention/comparator	-
494	2010	Community-based aquatic exercise and quality of life in persons with osteoarthritis	No	Without intervention/comparator	Without intervention/comparator	-
495	2013	Effect of ischemic compression on trigger points in the neck and shoulder muscles in office worl	No	Other populations/diseases	Other populations/diseases	-
496	2019	Effective conservative care targeting mechanical markers as risk factors for knee osteoarthritis p	No	Without intervention/comparator	Without intervention/comparator	-
497	2019	Impact of a personalized home exercise program for knee osteoarthritis patients on 3d kinematic	No	Without intervention/comparator	Without intervention/comparator	-
498	2020	A multi-arm cluster randomized clinical trial of the use of knee kinesiography in the managemen	No	Without intervention/comparator	Without intervention/comparator	-
499	2020	Impact of a personalized care approach on 3D gait impairments in knee osteoarthritis patients (a	No	Without intervention/comparator	Without intervention/comparator	-
500	2014	Efficacy of therapeutic ultrasound for the management of knee osteoarthritis: a randomized, cont	No	Without intervention/comparator	Without intervention/comparator	-
501	2014	The effectiveness of aquatic exercises in the treatment of rheumatoid arthritis	No	Without intervention/comparator	Without intervention/comparator	-
502	2016	Isokinetic exercise improves concentric knee flexion torque better than isometric exercise in pati	No	Without intervention/comparator	Without intervention/comparator	-
503	2017	High-intensity preoperative training improves physical and functional recovery in the early post-	No	Other populations/diseases	Other populations/diseases	-
504	2011	The effectiveness of TENS, interferential current and short-wave diathermy on knee osteoarthriti	No	Without intervention/comparator	Without intervention/comparator	-
505	2016	The Effect of Knee Braces on Quadriceps Strength and Inhibition in Subjects With Patellofemor	No	Without intervention/comparator	Without intervention/comparator	-
506	2016	Low income is associated with lower functional capacity and higher disease burden in RA patier	No	Without intervention/comparator	Without intervention/comparator	-
507	2009	Evidence of effectiveness of herbal medicinal products in the treatment of arthritis - Part 1: osteo	No	Without intervention/comparator	Without intervention/comparator	-
508	2010	Effectiveness and cost-effectiveness of arthroscopic lavage in the treatment of osteoarthritis of th	No	Without intervention/comparator	Without intervention/comparator	-
509	2019	Effects of High-Intensity Laser Therapy on Pain Sensitivity and Motor Performance in Patients v	No	Without intervention/comparator	Without intervention/comparator	-
510	2017	Immunosenescence and impact of exercise on cellular markers	No	Without intervention/comparator	Without intervention/comparator	-
511	2012	Vitamin D supplementation in the management of knee osteoarthritis: study protocol for a rando	No	Without intervention/comparator	Without intervention/comparator	-
512	2015	Effects of cholecalciferol and alphacalcidol on the grip strength in women with postmenopausal	No	Without intervention/comparator	Without intervention/comparator	-
513	2015	Physical performance in women with postmenopausal osteopenia or osteoporosis: effects of shor	No	Without intervention/comparator	Without intervention/comparator	-
514	2016	Improvement of balance in vitamin-D deficient women after 6 months treatment with either chol	No	Other populations/diseases	Other populations/diseases	-
515	2017	Report of the Primary Outcomes for Gait Mechanics in Men of the ACL-SPORTS Trial: seconda	No	Other populations/diseases	Without intervention/comparator	Other populations/dis
516	2018	Gait mechanics and tibiofemoral loading in men of the ACL-SPORTS randomized control trial	No	Other populations/diseases	Other populations/diseases	-
517	2019	Gait Mechanics in Women of the ACL-SPORTS Randomized Control Trial: interlimb Symmetry	No	Other populations/diseases	Other populations/diseases	-
518	2019	Superior 2-Year Functional Outcomes Among Young Female Athletes After ACL Reconstructio	No	Other populations/diseases	Other populations/diseases	-
519	2019	Stimulating versus non-stimulating catheter for lumbar plexus continuous infusion after total hip	No	Other populations/diseases	Other populations/diseases	-
520	2017	Efficacy and Safety of Diacerein in Patients With Inadequately Controlled Type 2 Diabetes: a Ra	No	Other populations/diseases	Other populations/diseases	-
521	2012	Effect of pulsed ultrasound and continuous ultrasound linked to exercise in patients with knee os	No	Without intervention/comparator	Without intervention/comparator	-
522	2015	Eficácia e custo-efetividade de uma intervenção de coaching de saúde para melhorar o estilo de v	No	Other topics	Without intervention/comparator	Other topics
523	2018	Randomized feasibility trial of the Scleroderma patient-centered intervention network hand exer	No	Other populations/diseases	Other populations/diseases	-
524	2019	Preoperative high-intensity strength training improves postural control after TKA: randomized-c	No	Without intervention/comparator	Other populations/diseases	Other populations/dis
525	2019	Effect of a multicomponent exercise programme (VIVIFRAIL) on functional capacity in frail cor	No	Other populations/diseases	Other populations/diseases	-

526	2013	Testing a novel bioactive marine nutraceutical on osteoarthritis patients	No	Without intervention/comparator	Without intervention/comparator	-
527	2017	Does the Dual-Mobility Hip Prosthesis Produce Better Joint Kinematics During Extreme Hip Fle	No	Other populations/diseases	Other populations/diseases	-
528	2015	TKR-power-patient outcomes using wii enhanced rehabilitation after a total knee replacement	No	Other populations/diseases	Other populations/diseases	-
529	2018	A specific protocol of autologous bone marrow concentrate and platelet products versus exercise	No	Without intervention/comparator	Without intervention/comparator	-
530	2016	Effectiveness of dry needling for chronic nonspecific neck pain: a randomized, single-blinded, c	No	Other populations/diseases	Other populations/diseases	-
**531**	**2019**	**Effects of blood flow restriction exercise with very low load and low volume in patients wit**	**Yes**	**Full**	**Full**	**-**
532	2009	No difference between home-based strength training and home-based balance training on pain in	No	Without intervention/comparator	Without intervention/comparator	-
533	2015	Effects of exercise interventions to improve sarcopenic indices among community-dwelling olde	No	Without intervention/comparator	Without intervention/comparator	-
534	2013	Acute postoperative pain following hospital discharge after total knee arthroplasty	No	Other populations/diseases	Other populations/diseases	-
535	2017	Associations between pre-intervention physical activity levels and treatment response to exercise	No	Without intervention/comparator	Without intervention/comparator	-
536	2019	Association between Pre-intervention Physical Activity Level and Treatment Response to Exerci	No	Without intervention/comparator	Without intervention/comparator	-
537	2017	Combining nutritional supplementation and progressive physical activity program improves func	No	Without intervention/comparator	Without intervention/comparator	-
538	2018	Detecting functional change in response to exercise in knee osteoarthritis: a comparison of two c	No	Without intervention/comparator	Without intervention/comparator	-
539	2014	Improving physical activity in arthritis clinical trial (IMPAACT): study design, rationale, recruit	No	Without intervention/comparator	Without intervention/comparator	-
540	2016	Exercise Alters Gait Pattern but Not Knee Load in Patients with Knee Osteoarthritis	No	Without intervention/comparator	Without intervention/comparator	-
541	2012	Effects of elastic-band exercise on lower-extremity function among female patients with osteoart	No	Without intervention/comparator	Without intervention/comparator	-
542	2015	Combined exercise and transcranial direct current stimulation intervention for knee osteoarthritis	No	Without intervention/comparator	Without intervention/comparator	-
543	2017	Addition of transcranial direct current stimulation to quadriceps strengthening exercise in knee c	No	Without intervention/comparator	Without intervention/comparator	-
544	2020	Effect of Systematic Exercise Rehabilitation on Patients With Knee Osteoarthritis: a Randomize	No	Without intervention/comparator	Without intervention/comparator	-
545	2018	How does frequency of manual therapy influence outcome for people with knee osteoarthritis? A	No	Without intervention/comparator	Without intervention/comparator	-
546	2014	Does the length of incision in the quadriceps affect the recovery of strength after total knee repla	No	Other populations/diseases	Other populations/diseases	-
547	2019	Transcranial direct current stimulation in combination with tens: effectiveness on pain and funct	No	Other populations/diseases	Other populations/diseases	-
548	2014	A comparison of home-based exercise programs with and without self-manual therapy in individ	No	Without intervention/comparator	Without intervention/comparator	-
549	2008	Effect of magnetic knee warp on quadriceps strength in patients with symptomatic knee osteoart	No	Without intervention/comparator	Without intervention/comparator	-
550	2018	Is it necessary to perform prehabilitation exercise for patients undergoing total knee arthroplasty:	No	Review/Systematic Review/Meta-Analys	Review/Systematic Review/Meta-Analys	-
551	2019	The effects of a home-based exercise intervention on elderly patients with knee osteoarthritis: a c	No	Without intervention/comparator	Without intervention/comparator	-
552	2020	Benefits of a transtheoretical model-based program on exercise adherence in older adults with kr	No	Without intervention/comparator	Without intervention/comparator	-
553	2013	Integrating acupuncture with exercise-based physical therapy for knee osteoarthritis: a randomize	No	Without intervention/comparator	Without intervention/comparator	-
554	2016	Influence of structured telephone follow-up on patient compliance with rehabilitation after total l	No	Other populations/diseases	Other populations/diseases	-
555	2016	Clinical efficacy of multi-pattern detumescence after total knee arthroplasty treated with acupoin	No	Other populations/diseases	Other populations/diseases	-
556	2019	Effects of Resistance Exercise on Glycated Hemoglobin and Functional Performance in Older Pa	No	Without intervention/comparator	Without intervention/comparator	-
557	2018	Treatment of Rheumatoid Arthritis by Bee-venom Acupuncture	No	Without intervention/comparator	Without intervention/comparator	-
558	2014	The efficacy of shock wave therapy in patients with knee osteoarthritis and popliteal cyamella	No	Without intervention/comparator	Without intervention/comparator	-
559	2014	Placebo effect in fibromyalgia-a systematic review of randomised controlled trials	No	Review/Systematic Review/Meta-Analys	Review/Systematic Review/Meta-Analys	-
560	2010	Efficacy of Shenshi Qianghuo Dihuang Decoction in rheumatoid arthritis: a randomized controll	No	Without intervention/comparator	Without intervention/comparator	-
561	2015	Clinical study of Xianling Gubao Capsule in the treatment of knee osteoarthritis	No	Without intervention/comparator	Without intervention/comparator	-
562	2015	Prospective Case Series of NMES for Quadriceps Weakness and Decrease Function in Patients v	No	Without intervention/comparator	Without intervention/comparator	-
563	2015	Strength and Functional Improvement Using Pneumatic Brace with Extension Assist for End-Sta	No	Without intervention/comparator	Other populations/diseases	Without intervention/c
564	2015	Use of Transcutaneous Electrical Nerve Stimulation Device in Early Osteoarthritis of the Knee	No	Without intervention/comparator	Without intervention/comparator	-
565	2016	Knee Osteoarthritis: does Transcutaneous Electrical Nerve Stimulation Work?	No	Without intervention/comparator	Without intervention/comparator	-
566	2012	Is yoga effective for knee osteoarthritis in older women?	No	Without intervention/comparator	Without intervention/comparator	-
567	2014	Yoga for managing knee osteoarthritis in older women: a pilot randomized controlled trial	No	Without intervention/comparator	Without intervention/comparator	-
568	2017	Managing knee osteoarthritis with yoga or aerobic/strengthening exercise programs in older adul	No	Without intervention/comparator	Without intervention/comparator	-
569	2016	Is yoga better than aerobic/strengthening exercises for managing knee osteoarthritis in older adul	No	Without intervention/comparator	Without intervention/comparator	-

570	2018	Immediate and short-term effects of gait retraining on the knee joint moments and symptoms in	No	Without intervention/comparator	Without intervention/comparator	-
571	2017	The phase Ia clinical trial of Proximod	No	Without intervention/comparator	Without intervention/comparator	-
572	2016	Tourniquet versus no tourniquet on rehabilitation after fast-track total knee arthroplasty	No	Other populations/diseases	Other populations/diseases	-
573	2016	Two methods of treatment for knee osteoarthritis	No	Without intervention/comparator	Without intervention/comparator	-
574	2016	Two methods of treatment of women with bilateral	No	Without intervention/comparator	Without intervention/comparator	-
575	2016	Two methods of treatment of women with bilateral knee osteoarthritis	No	Without intervention/comparator	Without intervention/comparator	-
576	2017	Conservative therapies on hip osteoarthritis	No	Without intervention/comparator	Without intervention/comparator	-
577	2016	Effect of high-dose postoperative dexamethasone on pain and recovery after total knee and hip a	No	Other populations/diseases	Other populations/diseases	-
578	2016	The early analgesia effects and functional rehabilitation of multi-site infiltration analgesia comp	No	Without intervention/comparator	Without intervention/comparator	-
579	2017	Application of Ultrasound guided Nerve Block in Arthroplasty	No	Without intervention/comparator	Without intervention/comparator	-
580	2017	The early analgesia effects and functional rehabilitation of different analgesia in fast-track total	No	Other populations/diseases	Other populations/diseases	-
581	2014	Combined application of celecoxib and exercise therapy in people with knee osteoarthritis: rand	No	Without intervention/comparator	Without intervention/comparator	-
582	2016	Comparison of femoral nerve block versus adductor canal block for analgesia after total knee art	No	Other populations/diseases	Other populations/diseases	-
583	2016	Effect of early exercise intervention patients underwent anterior cruciate ligament reconstruction	No	Other populations/diseases	Other populations/diseases	-
584	2016	Effects of whole body vibration exercise on neuromuscular function for individuals with knee os	No	Without intervention/comparator	Without intervention/comparator	-
585	2016	Explore Tai Chi exercise in improving knee osteoarthritis symptoms based on fMRI: a clinical st	No	Without intervention/comparator	Without intervention/comparator	-
586	2016	Sleep quality affects early knee function recovery and pain relief following total knee arthroplast	No	Other populations/diseases	Other populations/diseases	-
587	2016	Sleep quality affects early recovery following total hip arthroplasty, A Randomized, Double-blin	No	Other populations/diseases	Other populations/diseases	-
588	2016	The effect of hip muscle training on knee osteoarthritis	No	Without intervention/comparator	Without intervention/comparator	-
589	2016	Total knee arthroplasty: to applicate tourniquet or not with drainage? a prospective randomized	No	Other populations/diseases	Other populations/diseases	-
590	2017	Study on the clinical intervention of hip joint training in the treatment of knee osteoarthritis	No	Without intervention/comparator	Without intervention/comparator	-
591	2017	The effect of extracorporeal shock wave therapy on cartilage in knee osteoarthritis	No	Without intervention/comparator	Without intervention/comparator	-
592	2017	The effect of local infiltration anesthesia on pain and function in Yesultaneously bilateral total k	No	Other populations/diseases	Other populations/diseases	-
593	2015	Effectiveness of patella mobilization therapy versus waitlist control in knee osteoarthritis: a prag	No	Without intervention/comparator	Without intervention/comparator	-
594	2015	Rehabilitation following Total Knee Replacement for Sarcopenic Obesity in Elderly	No	Other populations/diseases	Other populations/diseases	-
595	2017	Effect of Additional Balance Training on Blood Biomachenical Value and Functional Fitness aft	No	Other populations/diseases	Other populations/diseases	-
596	2017	Efficacy of Core Muscle Training on the Walking of Incomplete Spinal Cord injury	No	Other populations/diseases	Other populations/diseases	-
597	2017	Managing knee osteoarthritis with Shi's exercise programs in older adults: a pilot randomized co	No	Without intervention/comparator	Without intervention/comparator	-
598	2017	Subarachnoid anesthesia combined with propofol TCI sedation: a more favorable choice for post	No	Without intervention/comparator	Without intervention/comparator	-
599	2017	Effect of Three Different Physical Therapy Approaches on Function and Disability of The Knee	No	Without intervention/comparator	Without intervention/comparator	-
600	2011	A Prospective Randomized Study of A New Minimally Invasive Total Knee Arthroplasty Syster	No	Without intervention/comparator	Without intervention/comparator	-
601	2011	Clinical trial to evaluate the effect of a glucosamine containing functional food on joint health in	No	Without intervention/comparator	Without intervention/comparator	-
602	2013	A community applied research of traditional Chinese medicine rehabilitation scheme on balance	No	Other populations/diseases	Other populations/diseases	-
603	2013	Effectiveness and safety of Chinese herbal medicine in low responders in patient population of r	No	Without intervention/comparator	Without intervention/comparator	-
604	2013	Effects of tai chi program on neuromuscular function for patients with knee Osteoarthritis	No	Without intervention/comparator	Without intervention/comparator	-
605	2014	Rehabilitation following Total Knee Replacement	No	Other populations/diseases	Other populations/diseases	-
606	2014	Rehabilitation protocol of the Traditional Chinese Medicine on Patients with Dyskinesia of the K	No	Without intervention/comparator	Without intervention/comparator	-
607	2018	Outcome expectations and fibromyalgia: perceived benefits of exercise are associated with self-e	No	Other populations/diseases	Other populations/diseases	-
608	2012	Comparison of quadriceps-sparing minimally invasive and medial parapatellar total knee arthrop	No	Other populations/diseases	Other populations/diseases	-
609	2018	Analgesic effect of local infiltration anesthesia in different layers after total hip arthroplasty: a ra	No	Other populations/diseases	Other populations/diseases	-
610	2018	Effect of comprehensive rehabilitation on muscle strength, electromyography, muscle status, pro	No	Without intervention/comparator	Without intervention/comparator	-
611	2018	Effect of exercise therapy with remote monitoring on pain, physical function and quality of life i	No	Without intervention/comparator	Without intervention/comparator	-
612	2018	Effect of protein supplement combined with resistance exercise on body composition and physic	No	Without intervention/comparator	Without intervention/comparator	-
613	2018	Fenestration and debridement combined with percutaneous minimally invasive fibula implantati	No	Other populations/diseases	Other populations/diseases	-

614	2018	Tai chi chuan exercise improves postoperative rehabilitation in patients with primary total knee a	No	Without intervention/comparator	Without intervention/comparator	-
615	2018	The effect of YIJINJING on Knee Stability in knee osteoarthritis	No	Without intervention/comparator	Without intervention/comparator	-
616	2018	The Efficacy Outcome of Exercise Rehabilitation Training in Patients with Knee Osteoarthritis:	No	Without intervention/comparator	Without intervention/comparator	-
617	2019	A prospective, randomized controlled study of combined nerve block for postoperative analgesia	No	Other populations/diseases	Other populations/diseases	-
618	2019	A randomized controlled study for extracorporeal shock wave combined with peri-hip muscle tra	No	Other populations/diseases	Other populations/diseases	-
619	2019	Clinical research for physiotherapy intervention for knee osteoarthritis	No	Without intervention/comparator	Without intervention/comparator	-
620	2019	Outcome of different alignment in total knee arthroplasty in Chinese population: a randomized c	No	Other populations/diseases	Other populations/diseases	-
621	2019	Ropivacaine combined with dexamethasone was used in ACB and IPACK block to improve ana	No	Other populations/diseases	Other populations/diseases	-
622	2019	Ultrasound-guided quadratus lumborum block on postoperative pain management in posterior to	No	Without intervention/comparator	Without intervention/comparator	-
623	2020	Effects of morphine hydrochloride on periarticular infiltration analgesia in total knee arthroplast	No	Other populations/diseases	Other populations/diseases	-
624	2020	Efficacy of combined multiple nerve block on postoperative pain and functional outcome after to	No	Other populations/diseases	Other populations/diseases	-
625	2016	Quadriceps muscle weakness and the risk of knee cartilage loss on MRI in a population-based co	No	Without intervention/comparator	Without intervention/comparator	-
626	2015	Heterogeneity of physical function responses to exercise training in older adults	No	Without intervention/comparator	Other populations/diseases	Without intervention/c
627	2016	Does cruciate-retaining total knee arthroplasty show better quadriceps recovery than posterior-sta	No	Other populations/diseases	Other populations/diseases	-
628	2017	Randomized Controlled Trial Investigating the Role of Exercise in the Workplace to Improve Wo	No	Without intervention/comparator	Without intervention/comparator	-
629	2010	Active, passive and proprioceptive neuromuscular facilitation stretching are comparable in impro	No	Other populations/diseases	Other populations/diseases	-
630	2012	Cardiovascular risk factor changes following three different maintenance programs in obese kne	No	Without intervention/comparator	Without intervention/comparator	-
631	2012	Cardiovascular risk factors following three different maintenance programs in older obese indivi	No	Other populations/diseases	Other populations/diseases	-
632	2013	Comparison of three weight maintenance programs on cardiovascular risk, bone and vitamins in	No	Other populations/diseases	Other populations/diseases	-
633	2014	Long-term intervention with weight loss in patients with concomitant obesity and knee osteoarth	No	Without intervention/comparator	Without intervention/comparator	-
634	2010	Efficacy of dieting or exercise versus control in obese osteoarthritis patients after a clinically sig	No	Without intervention/comparator	Without intervention/comparator	-
635	2015	Effect of weight maintenance on symptoms of knee osteoarthritis in obese patients: a twelve-mon	No	Without intervention/comparator	Without intervention/comparator	-
636	2018	A Physical Therapist-Administered Physical Activity Intervention After Total Knee Replacemen	No	Other populations/diseases	Other populations/diseases	-
637	2016	Clinical Outcomes of a Pneumatic Unloader Brace for Kellgren-Lawrence Grades 3 to 4 Osteoar	No	Without intervention/comparator	Without intervention/comparator	-
638	2012	Long-term followup for rheumatoid arthritis patients in a multicenter outcomes study of silicone	No	Other populations/diseases	Other populations/diseases	-
639	2017	Seven-Year Outcomes of the Silicone Arthroplasty in Rheumatoid Arthritis Prospective Cohort S	No	Without intervention/comparator	Without intervention/comparator	-
640	2016	Cross-sectional and longitudinal construct validity for the improved health assessment questionn	No	Without intervention/comparator	Without intervention/comparator	-
641	2013	Strengthening exercises to improve hand strength and functionality in rheumatoid arthritis with l	No	Without intervention/comparator	Without intervention/comparator	-
642	2017	An 8-Week Neuromuscular Exercise Program for Patients With Mild to Moderate Knee Osteoart	No	Without intervention/comparator	Without intervention/comparator	-
643	2019	Effects of capacitive and resistive electric transfer therapy in patients with knee osteoarthritis: a	No	Without intervention/comparator	Without intervention/comparator	-
644	2014	Effectiveness of phototherapy incorporated into an exercise program for osteoarthritis of the kne	No	Without intervention/comparator	Without intervention/comparator	-
645	1986	Evaluation of arthritis self-management courses led by laypersons and by professionals	No	Without intervention/comparator	Without intervention/comparator	-
646	2011	An osteoarthritis of the knee self-management education program delivered by multidisciplinary	No	Without intervention/comparator	Without intervention/comparator	-
647	2011	Osteoarthritis of the knee; Self-management utilising multidisciplinary health professionals or la	No	Without intervention/comparator	Without intervention/comparator	-
648	2012	A randomised controlled trial of a self-management education program for osteoarthritis of the k	No	Without intervention/comparator	Without intervention/comparator	-
649	2019	Young to middle-aged adults with persistent patellofemoral pain demonstrate Yesilar pain, disab	No	Other populations/diseases	Other populations/diseases	-
650	2018	2018 Consensus statement on exercise therapy and physical interventions (orthoses, taping and r	No	Other topics	Without intervention/comparator	Other topics
651	2016	A comparison of multidisciplinary team residential rehabilitation with conventional outpatient ca	No	Without intervention/comparator	Without intervention/comparator	-
652	2016	Interposition Arthroplasty Versus Hematoma and Distraction for the Treatment of Osteoarthritis	No	Without intervention/comparator	Without intervention/comparator	-
653	2019	Corticosteroids injections versus corticosteroids with hyaluronic acid injections in rhizarthrosis:	No	Without intervention/comparator	Without intervention/comparator	-
654	2018	No effect of creatine monohydrate supplementation on inflammatory and cartilage degradation b	No	Without intervention/comparator	Without intervention/comparator	-
655	2014	Effectiveness of massage therapy as co-adjuvant treatment to exercise in osteoarthritis of the kne	No	Without intervention/comparator	Without intervention/comparator	-
656	2010	Discrete weight loss does not decrease the pain more than strength training exercises for the qua	No	Without intervention/comparator	Without intervention/comparator	-
657	2016	Effect of Teriparatide on Bone Formation in the Human Femoral Neck	No	Other populations/diseases	Other populations/diseases	-

658	2010	Effects of exercise intervention on knee morphology in middle-aged women: a longitudinal analy	No	Without intervention/comparator	Without intervention/comparator	-
659	2016	Supervised or Unsupervised Rehabilitation After Total Hip Replacement Provides Yesilar Impro	No	Other populations/diseases	Other populations/diseases	-
660	2017	Supervised or Unsupervised Rehabilitation After Total Hip Replacement Provides Yesilar Impro	No	Other populations/diseases	Other populations/diseases	-
661	2007	The cost effectiveness of behavioural graded activity in patients with osteoarthritis of hip and/or	No	Without intervention/comparator	Without intervention/comparator	-
662	1999	Responsiveness of endpoints in osteoporosis clinical trials - An update	No	Other populations/diseases	Other populations/diseases	-
663	2016	Pain and sensitization in women with aromatase inhibitor-associated arthralgias	No	Without intervention/comparator	Without intervention/comparator	-
664	2019	Effectiveness of Peer-Delivered Trauma Treatment in a Rural Community: a Randomized Non-ir	No	Other populations/diseases	Other populations/diseases	-
665	2010	Predictors of functional ambulation and patient perception following total knee replacement and	No	Without intervention/comparator	Other populations/diseases	Other populations/dis
666	2015	Incision in the Quadriceps of >4 cm Delayed Recovery of Strength After Total Knee Replacemer	No	Other populations/diseases	Other populations/diseases	-
667	2012	A randomised clinical trial of targeted physiotherapy for patellofemoral osteoarthritis	No	Without intervention/comparator	Without intervention/comparator	-
668	2014	Targeted physiotherapy treatment for patellofemoral osteoarthritis: a randomised clinical trial	No	Without intervention/comparator	Other populations/diseases	Without intervention/c
669	2015	Exercise, education, manual-therapy and taping compared to education for patellofemoral osteoa	No	Without intervention/comparator	Without intervention/comparator	-
670	2011	GI-REASONS: a novel 6-month, prospective, randomized, open-label, blinded end point (PROB	No	Without intervention/comparator	Other populations/diseases	Without intervention/c
671	2010	A clinical study to evaluate the efficacy and safety of FDC of Tolperisone and Paracetamol in pa	No	Other populations/diseases	Other populations/diseases	-
672	2010	A clinical trial to study the effects of two drugs, in fixed dose combination tablet - Aceclofenac a	No	Other populations/diseases	Without intervention/comparator	Other populations/dis
673	2010	A clinical trial to study the effects of two drugs, Tolperisone and Diclofenac in patients suffering	No	Other populations/diseases	Other populations/diseases	-
674	2010	A clinical trial to study the efficacy and safety of FDC tablet containing Tolperisone 150 mg and	No	Other populations/diseases	Other populations/diseases	-
675	2012	Benefits of Naturopathy and Yoga in the Treatment of Rheumatoid Arthritis Patients	No	Without intervention/comparator	Without intervention/comparator	-
676	2013	effect of taping on quadriceps strength in patients with osteoarthritis	No	Without intervention/comparator	Without intervention/comparator	-
677	2014	Bioequivalence study of Azathioprine to measure the pharmacokinetic profiles, safety and tolera	No	Without intervention/comparator	Without intervention/comparator	-
678	2014	Manual Therapy Versus Exercise Therapy in management of Osteoarthritis Knee	No	Without intervention/comparator	Without intervention/comparator	-
679	2014	Trial of 2 conservative therapies for knee arthritis patients	No	Without intervention/comparator	Without intervention/comparator	-
680	2015	To check the effect of strengthening exercises and manual therapy in subjects with knee osteoart	No	Without intervention/comparator	Without intervention/comparator	-
681	2016	Multi-centre, randomized, double-blind, two-arm, parallel group, comparative clinical study to e	No	Without intervention/comparator	Without intervention/comparator	-
682	2017	An experiment involving people with Rheumatoid arthritis (joint pain illness) to see the changes	No	Without intervention/comparator	Without intervention/comparator	-
683	2017	Does platelet rich plasma help in early osteoarthritis knee? If so does more than one dose help m	No	Without intervention/comparator	Without intervention/comparator	-
684	2017	Effect of exercise in knee pain	No	Without intervention/comparator	Without intervention/comparator	-
685	2017	exercise for hip muscle strengthening in total knee replacement surgery	No	Other populations/diseases	Other populations/diseases	-
686	2017	IMPROVEMENT IN FUNCTIONAL OUTCOMES WITH A PNEUMATIC UNLOADING BRA	No	Without intervention/comparator	Discussion	Without intervention/c
687	2017	Sugar Water injection Versus Steroid Injection in Knee pain	No	Without intervention/comparator	Without intervention/comparator	-
688	2017	To study the difference between strength of two suture materials used in the closure of wound af	No	Other populations/diseases	Other populations/diseases	-
689	2017	To study the effect of Yoga and Meditation on Rheumatoid Arthritis	No	Without intervention/comparator	Without intervention/comparator	-
690	2017	Yoga and Osteoarthritis	No	Without intervention/comparator	Without intervention/comparator	-
691	2018	A clinical trial to study the effect of two drugs, Rasnadidashmool kwatha and Erandadi kwath in	No	Without intervention/comparator	Without intervention/comparator	-
692	2018	A comparative study on hip muscle strength in patients with Osteoarthritis of knee joint, having	No	Without intervention/comparator	Without intervention/comparator	-
693	2018	Comparison of Effect of Adalimumab versus Hyaluronic Acid in Knee Osteoarthritis	No	Without intervention/comparator	Without intervention/comparator	-
694	2018	Effect of Electrical Stimulation on Pain and Function in Knee Pain	No	Without intervention/comparator	Without intervention/comparator	-
695	2018	Effect of two different electrical stimulation on quadriceps for pain and function in patients after	No	Other populations/diseases	Other populations/diseases	-
696	2018	Effect of Water Based Exercises Versus Artificially Created Environment In Form Of Game By (	No	Without intervention/comparator	Without intervention/comparator	-
697	2018	EFFECTIVENESS OF TELEPHONICALLY REINFORCED HOME EXERCISE PROGRAM F	No	Other populations/diseases	Other populations/diseases	-
698	2018	Effects of Whole Body Vibration and Wobble board Exercises in Knee osteoarhritis	No	Without intervention/comparator	Without intervention/comparator	-
699	2018	Immediate effect of release of myofascia on knee pain	No	Without intervention/comparator	Without intervention/comparator	-
700	2018	Long term benefits of specific physiotherapy for patients with osteoarthritis of knee	No	Without intervention/comparator	Without intervention/comparator	-
701	2018	Role of exercise and shoe modification in medial compartment knee osteoarthtritis	No	Without intervention/comparator	Without intervention/comparator	-

702	2018	Role of USG in therapeutic intervention of knee osteoarthritis	No	Without intervention/comparator	Without intervention/comparator	-
703	2018	Study to compare the effects of Knee strengthening exercises and Yoga on Osteoarthritis Knee	No	Without intervention/comparator	Without intervention/comparator	-
704	2019	A study to assess the effectiveness of a mobile app based intervention to improve activity, pain a	No	Without intervention/comparator	Without intervention/comparator	-
705	2019	Comparison of mobilization technique in two positions on functional mobility in elderly with kn	No	Without intervention/comparator	Without intervention/comparator	-
706	2019	Effect of Isokinetic knee muscle training in University football players with post-traumatic osteo	No	Without intervention/comparator	Without intervention/comparator	-
707	2019	Homeopathic medicines for knee pain	No	Without intervention/comparator	Without intervention/comparator	-
708	2019	Pain Science Education in patients with Chronic Knee Pain	No	Without intervention/comparator	Without intervention/comparator	-
709	2019	To check the effect of different mobilization on osteoathritis of knee on hamtring/ quadriceps rat	No	Without intervention/comparator	Without intervention/comparator	-
710	2019	To compare the effect of routienly done strengthening exercises versus joint position sence trinin	No	Without intervention/comparator	Without intervention/comparator	-
711	2019	Using a type of machine with current to reduce pain in patients with knee pain	No	Without intervention/comparator	Without intervention/comparator	-
712	2018	Vitamin D supplements for trunk muscle morphology in older adults: secondary analysis of a rar	No	Without intervention/comparator	Without intervention/comparator	-
713	2016	Effectiveness of a multicomponent physical training integrated into a multimodal primary care p	No	Other populations/diseases	Other populations/diseases	-
714	2015	The optimal frequency of aquatic physiotherapy for individuals with chronic musculoskeletal pa	No	Other populations/diseases	Other populations/diseases	-
715	2019	Efficacy of a self-management program in patients with chronic viral hepatitis in China	No	Other populations/diseases	Other populations/diseases	-
716	2015	Randomized trial of the effectiveness of a non-pharmacological multidisciplinary face-to-face tr	No	Without intervention/comparator	Without intervention/comparator	-
717	2016	Cost-Utility and Cost-Effectiveness Analyses of Face-to-Face Versus Telephone-Based Nonphar	No	Without intervention/comparator	Without intervention/comparator	-
718	2007	Prevention and treatment of glucocorticoid-induced osteoporosis	No	Other populations/diseases	Other populations/diseases	-
719	2010	Goal setting for managing rheumatoid arthritis fatigue: a qualitative exploration	No	Without intervention/comparator	Without intervention/comparator	-
720	2020	The addition of blood flow restriction to resistance exercise in individuals with knee pain: a syst	No	Review/Systematic Review/Meta-Analy	Review/Systematic Review/Meta-Analys	-
721	2017	Effect of standardized training on the reliability of the Cochrane risk of bias assessment tool: a p	No	Review/Systematic Review/Meta-Analy	Review/Systematic Review/Meta-Analys	-
722	2015	Efficacy of Yesple integrated group rehabilitation program for patients with knee osteoarthritis: s	No	Without intervention/comparator	Without intervention/comparator	-
723	2019	Additional effect of neuromuscular electrical stimulation on knee extension lag, pain and knee ra	No	Other populations/diseases	Other populations/diseases	-
724	2012	A prospective, randomized comparison of 3 types of proximal interphalangeal joint arthroplasty	No	Other populations/diseases	Other populations/diseases	-
725	2009	Treatment after anterior cruciate ligament injury: comment on the article by Ageberg et al	No	Other populations/diseases	Other populations/diseases	-
726	2013	Effect of extended release of posterior clearance on flexion clearence and range of motion of the	No	Other populations/diseases	Other populations/diseases	-
727	2007	Combination therapy for rheumatoid arthritis: methotrexate and sulfasalazine together or with ot	No	Without intervention/comparator	Without intervention/comparator	-
728	2019	Profitieren BeschÃ¤ftigte des Baugewerbes mit Kniegelenksbeschwerden vom Kniekolleg?	No	Other topics	Other topics	-
729	2017	Cryotherapy short-term use relieves pain, improves function and quality of life in individuals wi	No	Without intervention/comparator	Without intervention/comparator	-
730	2019	Short-term cryotherapy did not substantially reduce pain and had unclear effects on physical fun	No	Without intervention/comparator	Without intervention/comparator	-
731	2013	Comparison of mini-squats and straight leg raises in patients with knee osteoarthritis: a randomi	No	Without intervention/comparator	Without intervention/comparator	-
732	2018	The effects of aerobic exercise training on pain and disability from osteoarthritis of the knee in p	No	Without intervention/comparator	Without intervention/comparator	-
733	2012	Pilot randomized controlled trial comparing specific dynamic stability exercises with general exe	No	Without intervention/comparator	Without intervention/comparator	-
734	2015	The efficacy of a Mediterranean type diet on symptoms of osteoarthritis - A pilot study	No	Without intervention/comparator	Without intervention/comparator	-
735	2017	Lean muscle volume of the thigh has a stronger relationship with muscle power than muscle stre	No	Without intervention/comparator	Without intervention/comparator	-
736	2016	The effectiveness of galvanic electrotherapy and a conservative hand exercise program in a rheu	No	Without intervention/comparator	Without intervention/comparator	-
737	2012	Impact of educational and patient decision aids on decisional conflict associated with total knee	No	Other populations/diseases	Other populations/diseases	-
738	2018	Influence of a periodized circuit training protocol on intermuscular adipose tissue of patients wit	No	Without intervention/comparator	Without intervention/comparator	-
739	2019	A periodized training attenuates thigh intermuscular fat and improves muscle quality in patients	No	Without intervention/comparator	Without intervention/comparator	-
740	2012	Effectiveness of the exercise in groups in patients with osteoarthritis: pilot quasi-experimental st	No	Without intervention/comparator	Without intervention/comparator	-
741	2011	Can psychological interventions alter the physiological stress response of patients with rheumato	No	Without intervention/comparator	Without intervention/comparator	-
742	2017	A study evaluating bariatric surgery as primary therapy for patients with morbid obesity and sev	No	Without intervention/comparator	Without intervention/comparator	-
743	2015	Symptom onset and help-seeking trajectory of patients with rheumatoid arthritis	No	Without intervention/comparator	Without intervention/comparator	-
744	2019	Can radiomics help to predict skeletal muscle response to chemotherapy in stage IV non-small c	No	Other populations/diseases	Other populations/diseases	-
745	2004	Slowing of bone loss in patients with rheumatoid arthritis by long-term high-intensity exercise: r	No	Without intervention/comparator	Without intervention/comparator	-

746	2009	Long-term follow-up of a high-intensity exercise program in patients with rheumatoid arthritis	No	Without intervention/comparator	Without intervention/comparator	-
747	2019	The effect of low-level laser therapy and physical exercise on pain, stiffness, function, and spatic	No	Without intervention/comparator	Without intervention/comparator	-
748	2017	Effects of a therapeutic exercises program associated with pompage technique on pain, balance a	No	Without intervention/comparator	Without intervention/comparator	-
749	2018	Web-based multimedia education for people with patellofemoral pain: a preliminary analysis of a	No	Other populations/diseases	Other populations/diseases	-
750	2012	Impact of exercise on the functional capacity and pain of patients with knee osteoarthritis: a ranc	No	Without intervention/comparator	Without intervention/comparator	-
751	2018	Incorporation of photobiomodulation therapy into a therapeutic exercise program for knee osteoa	No	Without intervention/comparator	Without intervention/comparator	-
752	2020	Exercise program combined with electrophysical modalities in subjects with knee osteoarthritis:	No	Without intervention/comparator	Without intervention/comparator	-
753	2016	One-Year Results of an Educational Program on Osteoarthritis: a Prospective Randomized Contr	No	Without intervention/comparator	Without intervention/comparator	-
754	2014	Development of comorbidity-adapted exercise protocols for patients with knee osteoarthritis	No	Without intervention/comparator	Without intervention/comparator	-
755	2016	Effectiveness of tailored exercise therapy in patients with knee osteoarthritis and comorbidity: a	No	Without intervention/comparator	Without intervention/comparator	-
756	2016	Efficacy of tailored exercise therapy in patients with knee osteoarthritis and comorbidity: a rando	No	Without intervention/comparator	Without intervention/comparator	-
757	2017	Efficacy of Tailored Exercise Therapy on Physical Functioning in Patients With Knee Osteoarthr	No	Without intervention/comparator	Without intervention/comparator	-
758	2016	Long-term effects of a randomized, controlled, tailor-made weight-loss intervention in primary c	No	Without intervention/comparator	Without intervention/comparator	-
759	2019	The effect of high-intensity resistance training and vitamin d supplementation on muscle strengt	No	Without intervention/comparator	Without intervention/comparator	-
760	2020	The effect of high-intensity resistance training and vitamin d supplementation on muscle strengt	No	Without intervention/comparator	Without intervention/comparator	-
761	2016	Manual therapy combined with dry needling for the management of patients with patellofemoral	No	Other populations/diseases	Other populations/diseases	-
762	2014	A biomechanical therapy program for patients after total knee arthroplasty - A randomized contr	No	Without intervention/comparator	Without intervention/comparator	-
763	2018	Effect of yoga based lifestyle intervention on patients with knee osteoarthritis: a randomized con	No	Without intervention/comparator	Without intervention/comparator	-
764	2016	Baseline medical comorbidities in adults randomized in the STRIDE trial for psychostimulant us	No	Other populations/diseases	Other populations/diseases	-
765	2015	Prolotherapy versus corticosteroid injections and phonophoresis for the treatment of plantar fasc	No	Other populations/diseases	Other populations/diseases	-
766	2008	Is Video-Assisted Thoracic Surgery Lobectomy Better? Quality of Life Considerations	No	Other topics	Other topics	-
767	2017	Effect of continuous femoral nerve block combined with periarticular local infiltration analgesia	No	Other populations/diseases	Other populations/diseases	-
768	2019	A FiftyâTwoâWeek, Randomized, PlaceboâControlled Trial of Certolizumab Pegol in Nonradiog	No	Other populations/diseases	Other populations/diseases	-
769	2014	Impact of telephone reinforcement and negotiated contracts on behavioral predictors of exercise	No	Without intervention/comparator	Without intervention/comparator	-
770	2016	The effects of a novel pilates exercise prescription method on people with non-specific unilateral	No	Without intervention/comparator	Without intervention/comparator	-
771	2017	Efficacy of combined conservative therapies on clinical outcomes in patients with thumb base os	No	Without intervention/comparator	Without intervention/comparator	-
772	2013	Predicting knee joint loads in adults with knee osteoarthritis: the intensive diet and exercise for a	No	Without intervention/comparator	Without intervention/comparator	-
773	2018	Quadriceps-strengthening exercise and quadriceps and knee biomechanics during walking in kne	No	Without intervention/comparator	Without intervention/comparator	-
774	2019	Short-term effects of neuromuscular electrical stimulation and ultrasound therapies on muscle ar	No	Without intervention/comparator	Without intervention/comparator	-
775	2000	Manual physical therapy and exercise improved function in osteoarthritis of the knee	No	Without intervention/comparator	Without intervention/comparator	-
776	2016	A multicenter randomised, 1-year comparative effectiveness, parallel-group trial protocol of a ph	No	Without intervention/comparator	Without intervention/comparator	-
777	2016	A multicentre randomised, 1-year comparative effectiveness, parallel-group trial protocol of a ph	No	Other populations/diseases	Other populations/diseases	-
778	2013	Secondary fracture prevention-a New Delhi, India initiative	No	Other populations/diseases	Other populations/diseases	-
779	2017	Hydrotherapy improves pain and function in older women with knee osteoarthritis: a randomized	No	Without intervention/comparator	Without intervention/comparator	-
780	2011	The efficacy of paraffin bath therapy for hand osteoarthritis, El osteoartritinde parafin banyosu u	No	Without intervention/comparator	Without intervention/comparator	-
781	2013	Efficacy of paraffin bath therapy in hand osteoarthritis: a single-blinded randomized controlled t	No	Without intervention/comparator	Without intervention/comparator	-
782	2008	Comparison of effects of combined physical therapy program and exercise on corrupted balance	No	Without intervention/comparator	Without intervention/comparator	-
783	2016	The effects of closed kinetic chain exercise on articular cartilage morphology: myth or reality? a	No	Without intervention/comparator	Without intervention/comparator	-
784	2008	Long-term effects of kinesthesia/balance and strengthening exercises on patients with knee osteo	No	Without intervention/comparator	Without intervention/comparator	-
785	2019	Effects of serial mud baths on inflammatory rheumatic and degenerative diseases	No	Without intervention/comparator	Without intervention/comparator	-
786	2011	Intraarticular vs. extraarticular ropivacaine infusion following high-dose local infiltration analge	No	Without intervention/comparator	Without intervention/comparator	-
787	2014	Internet-mediated physiotherapy and pain coping skills training for people with persistent knee p	No	Without intervention/comparator	Without intervention/comparator	-
788	2012	Comparison of effects of isotonic and isometric hand exercises on pain, hand functions, dexterity	No	Without intervention/comparator	Without intervention/comparator	-
789	2013	Effects of isotonic and isometric hand exercises on pain, hand functions, dexterity and quality of	No	Without intervention/comparator	Without intervention/comparator	-

790	2018	The âefficacy paradoxâ	No	Without intervention/comparator	Without intervention/comparator	-
791	2017	Evaluating the agreement between self-reported and documented analgesic use in older veterans	No	Without intervention/comparator	Without intervention/comparator	-
792	2020	A randomized controlled trial assessing the effects of preoperative strengthening plus balance tra	No	Without intervention/comparator	Without intervention/comparator	-
793	2019	MULTIDISCIPLINARY REHABILITATION FOR ADULTS WITH NEUROMYELITIS OPTIC	No	Other populations/diseases	Other populations/diseases	-
794	2016	Could a disruptive out patient healthcare delivery model reduce escalating in-hospital healthcare	No	Without intervention/comparator	Without intervention/comparator	-
795	2009	The effect of glucosamine on glucose metabolism in humans: a systematic review of the literatur	No	Review/Systematic Review/Meta-Analys	Review/Systematic Review/Meta-Analys	-
796	2013	Comparison of the effects of isotonic and isometric hand exercises on pain, hand functions, dext	No	Without intervention/comparator	Without intervention/comparator	-
797	2017	Intra-articular steroids may hasten cartilage loss in knee osteoarthritis	No	Without intervention/comparator	Without intervention/comparator	-
798	2018	Effect of low-intensity long-duration ultrasound on the symptomatic relief of knee osteoarthritis:	No	Without intervention/comparator	Without intervention/comparator	-
799	2015	From theory to practice: case study from the SARAH trial	No	Other populations/diseases	Other populations/diseases	-
800	2011	Effects of a treatment for 12 weeks aquatic physical therapy on postural control in women with c	No	Other populations/diseases	Other populations/diseases	-
801	2011	Efficacy of conservative treatment regime for hip osteoarthritis - Evaluation of the therapeutic ex	No	Without intervention/comparator	Without intervention/comparator	-
802	2012	Intensified work-related rehabilitation aftercare	No	Without intervention/comparator	Without intervention/comparator	-
803	2013	Life in motion	No	Without intervention/comparator	Without intervention/comparator	-
804	2014	The influence of collagen hydrolysate on functional knee discomforts	No	Without intervention/comparator	Without intervention/comparator	-
805	2015	Effectiveness and efficiency of a supervised strength-machine based hip and knee training for pa	No	Without intervention/comparator	Without intervention/comparator	-
806	2015	Effects of the Sensorimotor Training Volume on Sensorimotor Function in Patients Following Lo	No	Without intervention/comparator	Without intervention/comparator	-
807	2015	Prehabilitation for patients with osteoarthritis of the hip or knee waiting for a total Joint replacem	No	Without intervention/comparator	Without intervention/comparator	-
808	2015	Prevention and rehabilitation of osteoporotic fractures in disadvantaged populations 2 â subproje	No	Without intervention/comparator	Without intervention/comparator	-
809	2015	Stepped movement-oriented rehabilitation and aftercare in patients with inflammatory and non-i	No	Without intervention/comparator	Without intervention/comparator	-
810	2016	Clinical study of an anthroposophic treatment strategy for early rheumatoid arthritis, compared t	No	Without intervention/comparator	Without intervention/comparator	-
811	2016	Preventing the progression of Osteoarthritis of the knee Grade 2- 3 by mechanical stimulation wi	No	Without intervention/comparator	Without intervention/comparator	-
812	2016	ReMove-It - Efficacy study of rehabilitation with telemedical assisted movement therapy after lo	No	Without intervention/comparator	Without intervention/comparator	-
813	2017	A multi-stage randomised controlled study for the assessment of postoperative therapeutic proce	No	Without intervention/comparator	Without intervention/comparator	-
814	2017	Evaluation of the effectiveness and costs of home tele-rehabilitation with AGT-Reha in comparis	No	Without intervention/comparator	Without intervention/comparator	-
815	2017	Impact of a tailored activity counselling intervention during inpatient rehabilitation after knee an	No	Without intervention/comparator	Without intervention/comparator	-
816	2018	Development and test of feedback loops of a digital application for exercise interventions in OA	No	Without intervention/comparator	Without intervention/comparator	-
817	2018	Influence of collagen hydrolysate on functional knee joint discomforts	No	Other populations/diseases	Other populations/diseases	-
818	2019	Feasibility and effectiveness of an individualized, group-based physical exercise program with e	No	Without intervention/comparator	Without intervention/comparator	-
819	2011	Self-management programs for chronic musculoskeletal pain conditions: a systematic review and	No	Review/Systematic Review/Meta-Analys	Review/Systematic Review/Meta-Analys	-
820	2016	Efficacy of yoga therapy in rheumatoid arthritis patients with normal values on measures of infla	No	Without intervention/comparator	Without intervention/comparator	-
821	2003	Treatment of knee joint arthritis in the middle-aged patient	No	Without intervention/comparator	Without intervention/comparator	-
822	2016	A self-determination theory based intervention to increase levels of cardiorespiratory fitness, self	No	Without intervention/comparator	Without intervention/comparator	-
823	2015	A self-determination theory based intervention to promote autonomous motivation and physical	No	Without intervention/comparator	Without intervention/comparator	-
824	2014	The effectiveness of galvanic current therapy and a conservative hand exercise program in a rheu	No	Without intervention/comparator	Without intervention/comparator	-
825	2016	The effectiveness of galvanic electrotherapy and a conservative hand exercise program in a rheu	No	Without intervention/comparator	Without intervention/comparator	-
826	2012	Effect of regenerative injection therapy on function and pain in patients with knee osteoarthritis:	No	Without intervention/comparator	Without intervention/comparator	-
827	2012	Assessment of the impact of proprioceptive exercises on balance and proprioception in patients v	No	Without intervention/comparator	Without intervention/comparator	-
828	2019	Efficacy of a compulsory homework programme for increasing physical activity and improving r	No	Other populations/diseases	Other populations/diseases	-
829	2018	Periosteal Electrical Dry Needling as an Adjunct to Exercise and Manual Therapy for Knee Oste	No	Without intervention/comparator	Without intervention/comparator	-
830	2019	Joint collapse is associated with increased pain but not reduced function in persons with thumb b	No	Without intervention/comparator	Without intervention/comparator	-
831	2020	Self-reported adherence is not influenced by pain or functional outcomes in thumb base osteoartl	No	Without intervention/comparator	Without intervention/comparator	-
832	2014	The effect of exercise on sleep and fatigue in rheumatoid arthritis: a randomised controlled study	No	Without intervention/comparator	Without intervention/comparator	-
833	2005	Effects of biofeedback assisted isometric exercise and electrical stimulation on pain, anxiety and	No	Without intervention/comparator	Without intervention/comparator	-

834	2012	Assessment of the effect of glucosamine sulfate and exercise on knee cartilage using magnetic re	No	Without intervention/comparator	Without intervention/comparator	-
835	2013	Effects of glucosamine sulfate and exercise therapy on serum leptin levels in patients with knee c	No	Without intervention/comparator	Without intervention/comparator	-
836	2016	The effect of capsaicin phonophoresis in knee osteoarthritis and can it be utilized early in primar	No	Without intervention/comparator	Without intervention/comparator	-
837	2020	Comparison of human versus digital instructions for exercise in patients with hip osteoarthritis: r	No	Without intervention/comparator	Without intervention/comparator	-
838	2015	Manual and manipulative therapy in addition to rehabilitation for osteoarthritis of the knee: asse	No	Without intervention/comparator	Without intervention/comparator	-
839	2016	Effect of a Mediterranean type diet on inflammatory and cartilage degradation biomarkers in pat	No	Without intervention/comparator	Without intervention/comparator	-
840	2011	Reducing selection bias and increasing generalisability of findings from randomised controlled t	No	Without intervention/comparator	Without intervention/comparator	-
841	2012	The clinical effectiveness of joint protection education and exercises in hand osteoarthritis (OA)	No	Without intervention/comparator	Without intervention/comparator	-
842	2015	Implementing osteoarthritis guidelines in UK primary care: mosaics cluster randomised controlle	No	Without intervention/comparator	Without intervention/comparator	-
843	2015	Self-management approaches for osteoarthritis in the hand: a 2Ã2 factorial randomised trial	No	Without intervention/comparator	Without intervention/comparator	-
844	2011	Self management, joint protection and exercises in hand osteoarthritis: a randomised controlled t	No	Without intervention/comparator	Without intervention/comparator	-
845	2017	Implementing core NICE guidelines for osteoarthritis in primary care with a model consultation (	No	Without intervention/comparator	Without intervention/comparator	-
846	2017	Minimum 10 year follow-up of a randomised trial investigating an accelerated weight bearing pr	No	Other populations/diseases	Other populations/diseases	-
847	2012	A randomized trial comparing accelerated and traditional approaches to postoperative weightbea	No	Other populations/diseases	Other populations/diseases	-
848	2017	Two-Year Outcomes of a Randomized Trial Investigating a 6-Week Return to Full Weightbearin	No	Other populations/diseases	Other populations/diseases	-
849	2012	Effects of an integrated approach of hatha yoga therapy on functional disability, pain, and flexib	No	Without intervention/comparator	Without intervention/comparator	-
850	2018	Reduction in infrapatellar fat pad (IPFP) volume during diet and exercise intervention is associat	No	Other populations/diseases	Other populations/diseases	-
851	2016	Biomechanical, neuromuscular and knee pain effects following therapeutic knee taping among p	No	Without intervention/comparator	Without intervention/comparator	-
852	2015	Effect of a model consultation on quality of care of osteoarthritis: a primary care cluster randomi	No	Without intervention/comparator	Without intervention/comparator	-
853	2013	Accelerated weightbearing rehabilitation after matrix-induced autologous chondrocyte implantat	No	Other populations/diseases	Other populations/diseases	-
854	2018	Development of a service delivery intervention and implementation plan for optimising primary	No	Without intervention/comparator	Without intervention/comparator	-
855	2011	Relationship between beliefs, motivation, and worries about physical activity and physical activi	No	Without intervention/comparator	Without intervention/comparator	-
856	2017	Effectiveness of an interactive telerehabilitation system with home-based exercise training in pat	No	Without intervention/comparator	Without intervention/comparator	-
857	2016	Effect of Electromyographic Biofeedback Training on Pain, Quadriceps Muscle Strength, and Fu	No	Without intervention/comparator	Without intervention/comparator	-
858	2011	Gait characteristics and potential effect of supervised exercise therapy in hip osteoarthritis patier	No	Without intervention/comparator	Without intervention/comparator	-
859	2012	Sagittal plane gait characteristics in hip osteoarthritis patients with mild to moderate symptoms c	No	Without intervention/comparator	Without intervention/comparator	-
860	2015	No effects of a 12-week supervised exercise therapy program on gait in patients with mild to mo	No	Without intervention/comparator	Without intervention/comparator	-
861	2017	HPR the effectiveness of the lower dose of laser treatment on knee osteoarthritis	No	Without intervention/comparator	Without intervention/comparator	-
862	2017	Impact of backward treadmill training on balance in children with juvenile rheumatoid arthritis:	No	Other populations/diseases	Without intervention/comparator	Other populations/dis
863	2017	Effects of 12 months of resistance training vs. endurance training on bone mineraldensityinyoun	No	Without intervention/comparator	Without intervention/comparator	-
864	2013	A step forward to close the loop: applying âjoint-fitnessâ and patient reported outcome measures	No	Without intervention/comparator	Without intervention/comparator	-
865	2018	Long-Term Effect of Pulsed Nd: YAG Laser in the Treatment of Children with Juvenile Rheuma	No	Without intervention/comparator	Other populations/diseases	Other populations/dis
866	2017	Effectiveness of Periosteal Stimulation Therapy and Home Exercise Program in the Rehabilitatio	No	Without intervention/comparator	Without intervention/comparator	-
867	2011	Quadriceps femoris muscle fatigue in patients with knee osteoarthritis	No	Without intervention/comparator	Without intervention/comparator	-
868	2011	The effect of electrical stimulation to the quadriceps muscle combined with group exercise on pa	No	Without intervention/comparator	Without intervention/comparator	-
869	2013	Does neuromuscular electrical stimulation enhance the effectiveness of an exercise programme i	No	Without intervention/comparator	Without intervention/comparator	-
870	2018	AbobotulinumtoxinA: a New Therapy for Hip Osteoarthritis. A Prospective Randomized Double	No	Without intervention/comparator	Without intervention/comparator	-
871	2018	Investigation of the effects of balance training on balance and functional status in patients with t	No	Without intervention/comparator	Without intervention/comparator	-
872	2019	Hand exercise for women with rheumatoid arthritis and decreased hand function: an exploratory	No	Without intervention/comparator	Without intervention/comparator	-
873	2019	The effect of 4Ã months exercise training on systemic biomarkers of cartilage and bone turnover	No	Without intervention/comparator	Without intervention/comparator	-
874	2016	Effect of odanacatib on bone density and estimated bone strength in postmenopausal women: a (	No	Other populations/diseases	Other populations/diseases	-
875	2003	Training effects on pain in rheumatoid arthritis	No	Without intervention/comparator	Without intervention/comparator	-
876	2018	Effectiveness of the Yesultaneous Stretching of the Achilles Tendon and Plantar Fascia in Indivi	No	Other populations/diseases	Other populations/diseases	-
877	2019	Is dry heat treatment (fluidotherapy) effective in improving hand function in patients with rheum	No	Without intervention/comparator	Without intervention/comparator	-

878	2011	A comparison of the effects of closed and open kinetic chain exercises on functional status in pa	No	Other populations/diseases	Other populations/diseases	-
879	2013	Lower extremity performance following ACL rehabilitation in the KANON-trial: impact of recor	No	Without intervention/comparator	Without intervention/comparator	-
880	2019	Outcomes of a Problem-Solving Medication Management Intervention for Informal Caregivers	No	Other populations/diseases	Other populations/diseases	-
881	2007	Massage is helpful for knee osteoarthritis: commentary	No	Without intervention/comparator	Without intervention/comparator	-
882	2015	Reduction of sedentary behaviour in patients with rheumatoid arthritis-experiences from an inter	No	Without intervention/comparator	Without intervention/comparator	-
883	1998	What do self-administered joint counts tell us about patients with rheumatoid arthritis?	No	Without intervention/comparator	Without intervention/comparator	-
884	2017	Randomized blinded trial of standardized written patient information before total knee arthropla:	No	Other populations/diseases	Other populations/diseases	-
885	2010	NeuFlex and Swanson metacarpophalangeal implants for rheumatoid arthritis: prospective rando	No	Without intervention/comparator	Without intervention/comparator	-
886	2011	Resection, interposition and suspension arthroplasty for treatment of Basal joint arthritis of the tl	No	Other populations/diseases	Other populations/diseases	-
887	2015	The role of supervised exercises on quality of life and depressive symptoms in postmenopausal v	No	Other populations/diseases	Other populations/diseases	-
888	2017	Effectiveness of Inclusion of Dry Needling in a Multimodal Therapy Program for Patellofemoral	No	Other populations/diseases	Other populations/diseases	-
889	2012	Effects of exercise on the function and quality of life in the institutionalised elderly diagnosed w	No	Without intervention/comparator	Other populations/diseases	Without intervention/c
890	2017	Effectiveness of Inclusion of Dry Needling in a Multimodal Therapy Program for Patellofemoral	No	Other populations/diseases	Other populations/diseases	-
891	2019	Effects of Taping and Balance Exercises on Knee and Lower Extremity Function in Amateur So	No	Other populations/diseases	Without intervention/comparator	Other populations/dis
892	2011	Efficacy of kots russian electroestimulation in quality of life, functional capacity, muscular force	No	Without intervention/comparator	Without intervention/comparator	-
893	2019	Comparison of high, medium and low mobilization forces for reducing pain and improving phys	No	Without intervention/comparator	Without intervention/comparator	-
894	1997	Exercise programs for seniors with knee osteoarthritis	No	Without intervention/comparator	Without intervention/comparator	-
895	2017	Lidocaine/diclofenac epolamine patch (GRT7019) Phase II proof-of-concept trial in patients witl	No	Without intervention/comparator	Without intervention/comparator	-
896	2008	Topical treatment of hand osteoarthritis with glucosamine cream	No	Without intervention/comparator	Without intervention/comparator	-
897	2015	A Two-stage 6-month, Multicentre, Randomised, Double-blind, Controlled Study on the Safety a	No	Without intervention/comparator	Without intervention/comparator	-
898	2015	RANKL-blockade for the treatment of erosive osteoarthritis (OA) of interphalangeal finger joints	No	Without intervention/comparator	Without intervention/comparator	-
899	2005	A phase II randomised, double-blind, multicenter, placebo-controlled, dose-ranging, parallel grou	No	Other populations/diseases	Other populations/diseases	-
900	2005	Effectiveness of leech therapy in epicondylitis humeroradialis - a randomized controlled trial - le	No	Other populations/diseases	Other populations/diseases	-
901	2007	Multiple dose, double-blind, double-dummy, placebo and active controlled study of pharmacoki	No	Other populations/diseases	Other populations/diseases	-
902	2012	study for healthy ageing	No	Other populations/diseases	Other populations/diseases	-
903	2017	Lidocaine/diclofenac epolamine patch (GRT7019) Phase II proof-of-concept trial in patients witl	No	Without intervention/comparator	Without intervention/comparator	-
904	2012	COMBINED INTRA ARTICULAR CORTICOSTEROID AND EXERCISE IN PATIENTS WIT	No	Without intervention/comparator	Without intervention/comparator	-
905	2014	Effect of highdose glucocorticoid on thigh muscle funktion, vesselwall function, immune system	No	Without intervention/comparator	Without intervention/comparator	-
906	2018	Can-Art Effect and safety of using Canabis derivatives for the treatment of pain in patients with	No	Without intervention/comparator	Other populations/diseases	Without intervention/c
907	2018	Medical cannabis for the treatment of pain in patients with hand osteoarthritis and psoriatic arthı	No	Without intervention/comparator	Without intervention/comparator	-
908	2019	Saline injections versus Education and Exercise in knee osteoarthritis	No	Without intervention/comparator	Without intervention/comparator	-
909	2019	Zoledronic acid for prevention of bone loss after bariatric surgery	No	Other populations/diseases	Other populations/diseases	-
910	2014	Study to evaluate anakinra efficacy in patients with hand's osteoarthritis	No	Without intervention/comparator	Without intervention/comparator	-
911	2016	Clinical trial where the treatment allocation is made randomly. Neither investigator nor patient w	No	Without intervention/comparator	Without intervention/comparator	-
912	2017	Treatment of Refractory Patellar Tendinopathy with MSV. Comparative study with P-PRP	No	Other populations/diseases	Other populations/diseases	-
913	2008	A Phase IIIb, open-label, run-in and double-blind, placebo-controlled, randomized study to evalu	No	Other populations/diseases	Other populations/diseases	-
914	2005	A prospective double-blind, randomised, cross-over trial to compare the effects of adding bupre	No	Without intervention/comparator	Without intervention/comparator	-
915	2010	A 24-week randomized placebo-controlled, double-blind multi-center clinical trial evaluating the	No	Other populations/diseases	Other populations/diseases	-
916	2012	Does hydroxychloroquine provide effective pain relief for people with hand osteoarthritis?	No	Without intervention/comparator	Without intervention/comparator	-
917	2009	Open, self-controlled phase IV. clinical study of the Nicoflex ointment on patients with knee artl	No	Without intervention/comparator	Without intervention/comparator	-
918	2013	INTRAMUSCULAR INJECTIONS OF CLODRONIC ACID IN THE FORMULATION OF 200	No	Without intervention/comparator	Without intervention/comparator	-
919	2008	Cost-effectiveness of viscosupplementation therapy for patients with osteoarthritis of the knee: a	No	Without intervention/comparator	Without intervention/comparator	-
920	2014	The effect of intensive muscle strength training and vitamin D supplements in persons with knee	No	Without intervention/comparator	Without intervention/comparator	-
921	2015	Comparison of nerveblock and local infiltration block in the knee after total knee replacement	No	Other populations/diseases	Other populations/diseases	-

922	2015	Randomized placebo-controlled trial to investigate clinical efficacy, anti-inflammatory propertie	No	Without intervention/comparator	Without intervention/comparator	-
923	2010	Home-based exercise and patellar bracig in the treatment of patellofemoral pain syndrome	No	Other populations/diseases	Other populations/diseases	-
924	2013	A randomized, prospective study of the effects of exercise on quality of life in postmenopausal w	No	Other populations/diseases	Other populations/diseases	-
925	2016	Effect of exercise on balance and functional mobility in postmenopausal women with vertebral f	No	Other populations/diseases	Other populations/diseases	-
926	2008	Does transcutaneous electrical nerve stimulation or therapeutic ultrasound increase the effectiver	No	Without intervention/comparator	Without intervention/comparator	-
927	2020	Comparative effectiveness of international osteoarthritis management program clinical cohorts: a	No	Without intervention/comparator	Without intervention/comparator	-
928	2001	Acupuncture for osteoarthritis of the knee: a systematic review	No	Review/Systematic Review/Meta-Analys	Review/Systematic Review/Meta-Analys	-
929	2018	Can we improve cognitive function among adults with osteoarthritis by increasing moderate-to-v	No	Without intervention/comparator	Without intervention/comparator	-
930	2016	Effects of Workloads and Cadences on Frontal Plane Knee Biomechanics in Cycling	No	Without intervention/comparator	Without intervention/comparator	-
931	2019	Effect of kinesio taping on pain, range of motion, hand strength, and functional abilities in patie	No	Without intervention/comparator	Without intervention/comparator	-
932	2015	Analysis and correlation of physical therapy tests, pain, function and quality of life questionnaire	No	Without intervention/comparator	Without intervention/comparator	-
933	2010	Progressive resistance training improves overall physical activity levels in patients with early ost	No	Without intervention/comparator	Without intervention/comparator	-
934	2009	Osteoarthritis and exercise: a review of the literature	No	Review/Systematic Review/Meta-Analys	Review/Systematic Review/Meta-Analys	-
935	2013	Association of severity of coexisting patellofemoral disease with increased impairments and func	No	Without intervention/comparator	Without intervention/comparator	-
936	2015	Policy-into-practice for rheumatoid arthritis: randomized controlled trial and cohort study of e-le	No	Without intervention/comparator	Without intervention/comparator	-
937	2015	Workforce capacity building in management of rheumatoid arthritis: a randomised controlled tria	No	Without intervention/comparator	Without intervention/comparator	-
938	2018	Effects of In-Person and Distance Exercise Training on Outcomes of Knee Injury and Osteoarthr	No	Other populations/diseases	Other populations/diseases	-
939	2014	Comparison of the clinical effectiveness of thermal cure and rehabilitation in knee osteoarthritis.	No	Without intervention/comparator	Without intervention/comparator	-
940	2016	What is the role of gait assessment in people with gluteal tendinopathy? a case controlled study c	No	Without intervention/comparator	Without intervention/comparator	-
941	2017	Pain, not structural impairments may explain activity limitations in people with gluteal tendinop	No	Without intervention/comparator	Without intervention/comparator	-
942	2016	Computer-assisted total knee arthroplasty using mini midvastus or medial parapatellar approach	No	Other populations/diseases	Other populations/diseases	-
943	2010	The use of magnetic-laser therapy in the combined treatment of osteoarthrosis in workers expose	No	Without intervention/comparator	Without intervention/comparator	-
944	2011	The effects of daily weather on accelerometer-measured physical activity	No	Other populations/diseases	Other populations/diseases	-
945	2012	Association of functional status with changes in physical activity: insights from a behavioral inte	No	Without intervention/comparator	Without intervention/comparator	-
946	2016	Effects of Person-Centered Physical Therapy on Fatigue-Related Variables in Persons With Rheu	No	Without intervention/comparator	Without intervention/comparator	-
947	2012	Beneficial effects of strontium ranelate compared to alendronate on bone mass and strength para	No	Other populations/diseases	Other populations/diseases	-
948	2016	The association of light and moderate-to-vigorous walking with incident poor health outcomes o	No	Without intervention/comparator	Without intervention/comparator	-
949	2017	Sedentary behaviour is associated with increased long-term cardiovascular risk in patients with r	No	Without intervention/comparator	Without intervention/comparator	-
950	2018	A self-determination theory based intervention to promote autonomous motivation for, and enga	No	Without intervention/comparator	Without intervention/comparator	-
951	2018	Autonomy support, light physical activity and psychological well-being in Rheumatoid Arthritis	No	Without intervention/comparator	Without intervention/comparator	-
952	2020	Testing a self-determination theory-based process model of physical activity behavior change in	No	Without intervention/comparator	Without intervention/comparator	-
953	2019	Patients' Perceptions of an Exercise Program Delivered Following Discharge From Hospital Afte	No	Without intervention/comparator	Without intervention/comparator	-
954	2010	Efficacy of patient education and supervised exercise vs patient education alone in patients with	No	Without intervention/comparator	Without intervention/comparator	-
955	2015	Supervised neuromuscular exercise prior to hip or knee replacement: cost-utility analysis alongsi	No	Without intervention/comparator	Without intervention/comparator	-
956	2017	Supervised neuromuscular exercise prior to hip and knee replacement: 12-month clinical effect a	No	Without intervention/comparator	Without intervention/comparator	-
957	2002	Effectiveness of acupuncture in the treatment of pain from osteoarthritis of the knee	No	Without intervention/comparator	Without intervention/comparator	-
958	2015	Phase 2 randomized, double blind, placebo controlled trial of myostatin antibody in older fallers	No	Other populations/diseases	Other populations/diseases	-
959	2018	Benefícios do treinamento de resistência com restrição do fluxo sanguíneo na osteoartrite do joel	No	Other topics (Portuguese version)	Full	Other topics (Portug
960	2015	Effect of low-level laser therapy (904 nm) and static stretching in patients with knee osteoarthriti	No	Without intervention/comparator	Without intervention/comparator	-
961	2016	EficÃ¡cia do treino de marcha e de equilÃbrio em pacientes com osteoartrite de joelho	No	Without intervention/comparator	Without intervention/comparator	-
962	2013	Rheumatoid arthritis in upper limbs benefits from moderate pressure massage therapy	No	Without intervention/comparator	Without intervention/comparator	-
963	2009	The evaluation of efficacy and tolerability of Hylan G-F 20 in bilateral thumb base osteoarthritis	No	Without intervention/comparator	Without intervention/comparator	-
964	2011	Long-term effects of comprehensive inpatient rehabilitation on function and disease activity in p	No	Without intervention/comparator	Without intervention/comparator	-
965	2017	CORR Insights: report of the Clinical and Functional Primary Outcomes in Men of the ACL-SP(	No	Other populations/diseases	Other populations/diseases	-

966	2017	Delaying ACL reconstruction and treating with exercise therapy alone may alter prognostic facto	No	Other populations/diseases	Other populations/diseases	-
967	2018	Formal Physical Therapy May Not Be Necessary After Unicompartmental Knee Arthroplasty: a l	No	Without intervention/comparator	Without intervention/comparator	-
968	2003	The exercise prescription in rheumatoid arthritis: primum non nocere	No	Without intervention/comparator	Without intervention/comparator	-
969	2018	Exercise Hemodynamic and Functional Capacity After Mitral Valve Replacement in Patients Wi	No	Other populations/diseases	Other populations/diseases	-
970	2014	Short- and long-term effects of mud-bath treatment on hand osteoarthritis: a randomized clinical	No	Without intervention/comparator	Without intervention/comparator	-
971	2019	The influence of antiresorptive bone medication on the effect of high-intensity resistance and im	No	Other populations/diseases	Other populations/diseases	-
972	2009	Assessing infection risk with biologic agents in RA: methodological challenges	No	Without intervention/comparator	Without intervention/comparator	-
973	2015	Comparative effects of 2 aqua exercise programs on physical function, balance, and perceived qu	No	Without intervention/comparator	Without intervention/comparator	-
974	2019	Batching smartphone notifications can improve well-being	No	Other populations/diseases	Other populations/diseases	-
975	2011	Agility and perturbation training techniques in exercise therapy for reducing pain and improving	No	Without intervention/comparator	Without intervention/comparator	-
976	2012	Associations for change in physical and psychological factors and treatment response following	No	Without intervention/comparator	Without intervention/comparator	-
977	2016	Exercise, manual therapy, and use of booster sessions in physical therapy for knee osteoarthritis:	No	Without intervention/comparator	Without intervention/comparator	-
978	2018	The effectiveness of platelet-rich plasma injections in gluteal tendinopathy-a randomised, double	No	Other populations/diseases	Other populations/diseases	-
979	2016	Self-Reported Knee Instability Before and After Total Knee Replacement Surgery	No	Other populations/diseases	Other populations/diseases	-
980	2019	2018 John N. Insall Award: recovery of Knee Flexion With Unsupervised Home Exercise Is Not	No	Other populations/diseases	Other populations/diseases	-
981	2019	A Phase II Trial of Lutikizumab, an AntiâInterleukinâ1Î±/Î² Dual Variable Domain Immunoglobu	No	Without intervention/comparator	Without intervention/comparator	-
982	2016	No difference in muscle strength and functional performance in middle-aged individuals with kn	No	Without intervention/comparator	Without intervention/comparator	-
983	2009	Assessment of a sixteen-week training program on strength, pain, and function in rheumatoid art	No	Without intervention/comparator	Without intervention/comparator	-
984	2017	Diferenças raciais na função baseada no desempenho e fatores explicativos potenciais entre paci	No	Without intervention/comparator	Without intervention/comparator	-
985	2012	Improving maintenance of physical activity in older, knee osteoarthritis patients trial-pilot (IMP	No	Without intervention/comparator	Without intervention/comparator	-
986	2014	Group-mediated physical activity promotion and mobility in sedentary patients with knee osteoa	No	Without intervention/comparator	Without intervention/comparator	-
987	2017	A group-mediated physical activity intervention in older knee osteoarthritis patients: effects on s	No	Without intervention/comparator	Without intervention/comparator	-
988	2010	External fixation versus closed treatment of displaced distal radial fractures in elderly patients: a	No	Other populations/diseases	Other populations/diseases	-
989	2017	Effects of an aquatic physiotherapy program on cardiovascular outcomes and functional aerobic	No	Without intervention/comparator	Without intervention/comparator	-
990	2017	Novel aquatic physiotherapy programme for elderly Chinese adults with osteoarthritis of the kne	No	Without intervention/comparator	Without intervention/comparator	-
991	2010	Spa therapy in the treatment of knee osteoarthritis: a large randomised multicentre trial	No	Without intervention/comparator	Without intervention/comparator	-
992	2014	Crenobalneotherapy (spa therapy) in patients with knee and generalized osteoarthritis: a post-hoc	No	Without intervention/comparator	Without intervention/comparator	-
993	2014	Spa therapy in the treatment of knee osteoarthritis: THERMARTHROSE a large randomised mu	No	Without intervention/comparator	Without intervention/comparator	-
994	2010	Dynamic alignment and its association with knee adduction moment in medial knee osteoarthriti	No	Without intervention/comparator	Without intervention/comparator	-
995	2011	Lower limb muscle strengthening does not change frontal plane moments in women with knee os	No	Without intervention/comparator	Without intervention/comparator	-
996	2011	Progressive resistance training and dynamic alignment in osteoarthritis: a single-blind randomise	No	Without intervention/comparator	Without intervention/comparator	-
997	2011	Cost-effectiveness of diet and exercise interventions to reduce overweight and obesity	No	Other populations/diseases	Without intervention/comparator	Other populations/dis
998	2007	The value of acupuncture or exercise-based physiotherapy for patients waiting for knee replacem	No	Other populations/diseases	Other populations/diseases	-
999	2015	Improving the effectiveness of exercise therapy for older adults with knee osteoarthritis: a pragm	No	Without intervention/comparator	Without intervention/comparator	-
1000	2010	The relationship between patient and practitioner expectations and preferences and clinical outco	No	Without intervention/comparator	Without intervention/comparator	-
1001	2013	Management of primary care for people with knee osteoarthritis: the beep study (best evidence fo	No	Without intervention/comparator	Without intervention/comparator	-
1002	2014	A multicentre, pragmatic, parallel group, randomised controlled trial to compare the clinical and	No	Without intervention/comparator	Without intervention/comparator	-
1003	2015	Improving the effectiveness of exercise therapy for older adults with knee pain: a pragmatic rand	No	Other populations/diseases	Other populations/diseases	-
1004	2016	Improving the effectiveness of exercise therapy for older adults with knee pain: a pragmatic rand	No	Other populations/diseases	Other populations/diseases	-
1005	2019	Clinical effectiveness of enhanced exercise therapy for adults with knee osteoarthritis. 3 year foll	No	Without intervention/comparator	Without intervention/comparator	-
1006	1999	Home exercise produces small reductions in pain and functional limitation in patients with osteo	No	Without intervention/comparator	Without intervention/comparator	-
1007	2003	Isometric muscle force measurement for clinicians treating patients with osteoarthritis of the kne	No	Without intervention/comparator	Without intervention/comparator	-
1008	2009	Land-based exercise for osteoarthritis of the knee: a metaanalysis of randomized controlled trials	No	Review/Systematic Review/Meta-Analy	Review/Systematic Review/Meta-Analys	-
1009	2010	Does land-based exercise reduce pain and disability associated with hip osteoarthritis? A meta-a	No	Without intervention/comparator	Without intervention/comparator	-

1010	2017	Post-Acute Rehabilitation After Total Knee Replacement: a Multicenter Randomized Clinical Tr	No	Other populations/diseases	Other populations/diseases	-
1011	2018	Blood flow restriction training as a prehabilitation concept in total knee arthroplasty: A narrative	No	Review/Systematic Review/Meta-Analy:	Review/Systematic Review/Meta-Analys	-
1012	2018	Blood flow restriction training as a prehabilitation concept in total knee arthroplasty: A narrative	No	Review/Systematic Review/Meta-Analy:	Review/Systematic Review/Meta-Analys	-
1013	2011	Osteoarthritis	No	Without intervention/comparator	Without intervention/comparator	-
1014	2011	The effectiveness of exercise therapy with and without manual therapy for hip osteoarthritis: a m	No	Without intervention/comparator	Without intervention/comparator	-
1015	2010	Measuring patient satisfaction with exercise therapy for knee osteoarthritis: evaluating the utility	No	Without intervention/comparator	Without intervention/comparator	-
1016	2011	Manual therapy for osteoarthritis of the hip or knee - A systematic review	No	Review/Systematic Review/Meta-Analy:	Review/Systematic Review/Meta-Analys	-
1017	2011	The effectiveness of exercise with and without manual therapy for hip osteoarthritis: a multi-cen	No	Without intervention/comparator	Without intervention/comparator	-
1018	2013	Exercise and manual physiotherapy arthritis research trial (EMPART) for osteoarthritis of the hi	No	Without intervention/comparator	Without intervention/comparator	-
1019	2014	Predictors of short-term outcome to exercise and manual therapy for people with hip osteoarthrit	No	Without intervention/comparator	Without intervention/comparator	-
1020	2015	Biomechanical analysis in patients with knee osteoarthritis after chinese massage combined with	No	Without intervention/comparator	Without intervention/comparator	-
1021	2010	Analgesic effect of raloxifene on back and knee pain in postmenopausal women with osteoporos	No	Without intervention/comparator	Without intervention/comparator	-
1022	2011	Comparison of the effects of elcatonin and risedronate on back and knee pain by electroalgomet	No	Other populations/diseases	Other populations/diseases	-
1023	2011	Pulsed shortwave treatment in women with knee osteoarthritis: a multicenter, randomized, place	No	Without intervention/comparator	Without intervention/comparator	-
1024	2014	Effects of high-velocity resistance training on muscle function, muscle properties, and physical p	No	Without intervention/comparator	Without intervention/comparator	-
1025	2017	Effects of High- and Low-Velocity Resistance Training on Gait Kinematics and Kinetics in Indiv	No	Without intervention/comparator	Without intervention/comparator	-
1026	2013	Viabilidade da suplementação de ácido graxo Ï ?? - 3 como terapia adjuvante para pessoas com c	No	Other populations/diseases	Other populations/diseases	-
1027	2010	Arthroscopic debridement and synovectomy for treating basal joint arthritis	No	Without intervention/comparator	Without intervention/comparator	-
1028	2011	Symptomatic effects of chondroitin 4 and chondroitin 6 sulfate on hand osteoarthritis: a randomi	No	Without intervention/comparator	Without intervention/comparator	-
1029	2015	Denosumab significantly increases BMD compared with alendronate in postmenopausal women	No	Without intervention/comparator	Without intervention/comparator	-
1030	2013	Impact of a supervised muscular training on perceived quality of sleep and health in a populatior	No	Without intervention/comparator	Without intervention/comparator	-
1031	2015	Effect of glucocorticoid, bisphosphonate therapy and disease activity on metacarpal shaft morph	No	Other populations/diseases	Other populations/diseases	-
1032	2016	Does menopausal hormone therapy (MHT), exercise or a combination of both, improve pain and	No	Other populations/diseases	Other populations/diseases	-
1033	2017	Education with exercise improves pain and dysfunction in post-menopausal women with greater	No	Other populations/diseases	Other populations/diseases	-
1034	2018	Gluteal Loading Versus Sham Exercises to Improve Pain and Dysfunction in Postmenopausal W	No	Other populations/diseases	Other populations/diseases	-
1035	2012	Five- to 18-year follow-up for treatment of trapeziometacarpal osteoarthritis: a prospective comp	No	Without intervention/comparator	Without intervention/comparator	-
1036	2017	Comparison of analgesic effects between multimodal and patient-controlled intravenous analges	No	Without intervention/comparator	Without intervention/comparator	-
1037	2002	A randomized controlled clinical trial of actarit in the treatment of rheumatoid arthritis	No	Without intervention/comparator	Without intervention/comparator	-
1038	1997	The use of isokinetic systems at osteoarthritis during in-patient rehabilitation, part I - Isokinetic t	No	Without intervention/comparator	Without intervention/comparator	-
1039	2019	Effect of a Dynamic Exercise Program in Combination With Mediterranean Diet on Quality of L	No	Without intervention/comparator	Without intervention/comparator	-
1040	2017	Effect of personalized diet and exercise recommendations in early inflammatory arthritis: a rand	No	Without intervention/comparator	Without intervention/comparator	-
1041	2018	Personalized diet and exercise recommendations in early rheumatoid arthritis: a feasibility trial	No	Without intervention/comparator	Without intervention/comparator	-
1042	2000	Shoulder arthroplasty with or without resurfacing of the glenoid in patients who have osteoarthri	No	Without intervention/comparator	Without intervention/comparator	-
1043	2019	A qualitative study of the experiences and perceptions of adults with chronic musculoskeletal co	No	Without intervention/comparator	Without intervention/comparator	-
1044	2014	Knee arthroscopic surgery is beneficial to middle-aged patients with meniscal symptoms: a prosp	No	Without intervention/comparator	Without intervention/comparator	-
1045	2017	Knee Arthroscopic Surgery in Middle-Aged Patients With Meniscal Symptoms: a 3-Year Follow	No	Without intervention/comparator	Without intervention/comparator	-
1046	2019	Knee arthroscopic surgery in middle-aged patients with meniscal symptoms: a 5-year follow-up	No	Without intervention/comparator	Without intervention/comparator	-
1047	2017	Occlusion training: pilot study for postoperative lower extremity rehabilitation following primary	No	Other populations/diseases	Other populations/diseases	-
1048	2018	Comparative evaluation of periarticular infiltration of two cocktail regimens for analgesia in pos	No	Other populations/diseases	Other populations/diseases	-
1049	2017	HPR self-management exercise program associated to spa therapy increased the physical activity	No	Without intervention/comparator	Without intervention/comparator	-
1050	2017	Self-management exercise program associated to spa therapy increased the physical activity leve	No	Without intervention/comparator	Without intervention/comparator	-
1051	2018	Efficacy of self-management exercise program with spa therapy for behavioral management of k	No	Without intervention/comparator	Without intervention/comparator	-
1052	2019	Limited effect of a self-management exercise program added to spa therapy for increasing physic	No	Without intervention/comparator	Without intervention/comparator	-
1053	2019	An investigation of efficacy of manual therapy and exercise in patients with glenohumeral arthri	No	Without intervention/comparator	Without intervention/comparator	-

1054	2012	The effect of mud pack therapy on serum YKL-40 and hsCRP levels in patients with knee osteoa	No	Without intervention/comparator	Without intervention/comparator	-
1055	2019	Modulation of Adaptive Cognitive Control by Prefrontal High-Definition Transcranial Direct Cu	No	Without intervention/comparator	Without intervention/comparator	-
1056	2019	Dose-response relationship between physical activity and mortality in people with non-communi	No	Without intervention/comparator	Without intervention/comparator	-
1057	2020	German recommendations for physical activity and physical activity promotion in adults with nc	No	Without intervention/comparator	Without intervention/comparator	-
1058	2009	Effect of neuromuscular electrical stimulation on pain and functional parameters in knee osteoar	No	Without intervention/comparator	Without intervention/comparator	-
1059	2019	Long-term safety and efficacy of sarilumab plus methotrexate on disease activity, physical functi	No	Without intervention/comparator	Without intervention/comparator	-
1060	2015	Effects of a multi-modal exercise program on BMD, muscle function, knee cartilage structure, d	No	Without intervention/comparator	Without intervention/comparator	-
1061	2010	Hydrotherapy after total hip arthroplasty: a follow-up study	No	Other populations/diseases	Other populations/diseases	-
1062	1998	The treatment of arthritis with a lipid extract of Perna canaliculus: a randomized trial	No	Without intervention/comparator	Without intervention/comparator	-
1063	2014	Femoral fixation of hamstring tendon grafts in ACL reconstructions: the 2-year follow-up results	No	Other populations/diseases	Other populations/diseases	-
1064	2018	A randomized trial of a motivational interviewing intervention to increase lifestyle physical activ	No	Without intervention/comparator	Without intervention/comparator	-
1065	2017	Quadriceps strengthening with and without blood flow restriction in the treatment of patellofemo	No	Without intervention/comparator	Discussion	Without intervention/c
1066	2018	Generic versus neuromuscular strengthening: symptomatic relief and improved physical functior	No	Without intervention/comparator	Without intervention/comparator	-
1067	2016	Reliability and responsiveness of an omeract tenosynovitis magnetic resonance imaging scoring	No	Without intervention/comparator	Without intervention/comparator	-
1068	2011	Non-cardiopulmonary factors affecting the six-minute walk distance in patients with sickle cell c	No	Other populations/diseases	Other populations/diseases	-
1069	2019	Adverse events associated with encorafenib plus binimetinib in the COLUMBUS study: incidenc	No	Other populations/diseases	Other populations/diseases	-
1070	2019	Evaluating the efficacy of Internet-Based Exercise programme Aimed at Treating knee Osteoartl	No	Without intervention/comparator	Without intervention/comparator	-
1071	2019	Clinical and Radiological Comparison of Effects of Platelet-Rich Plasma, Hyaluronic Acid, and	No	Without intervention/comparator	Without intervention/comparator	-
1072	2010	Assessment of the effectiveness of a functional splint for osteoarthritis of the trapeziometacarpal	No	Without intervention/comparator	Without intervention/comparator	-
1073	2011	Sensorimotor training versus resistance training in patients with knee osteoarthritis	No	Without intervention/comparator	Without intervention/comparator	-
1074	2018	Sensory-motor training versus resistance training among patients with knee osteoarthritis: rando	No	Without intervention/comparator	Without intervention/comparator	-
1075	2013	Retrowalking as an adjunct to conventional treatment versus conventional treatment alone on pa	No	Without intervention/comparator	Without intervention/comparator	-
1076	2016	Interventions for the management of long-term post-surgical pain after total knee replacement: a	No	Review/Systematic Review/Meta-Analys	Review/Systematic Review/Meta-Analys	-
1077	2017	2017 American College of Rheumatology/American Association of Hip and Knee Surgeons Guic	No	Without intervention/comparator	Without intervention/comparator	-
1078	2015	The cost-effectiveness of aquatic physiotherapy for patients with rheumatoid arthritis	No	Without intervention/comparator	Without intervention/comparator	-
1079	2015	The effectiveness of aquatic physiotherapy in patients with rheumatoid arthritis: a randomised co	No	Without intervention/comparator	Without intervention/comparator	-
1080	2020	Wireless Motion Sensors-Useful in Assessing the Effectiveness of Physiotherapeutic Methods U	No	Without intervention/comparator	Without intervention/comparator	-
1081	2011	Conservative treatment of fresh scaphoid fractures supplemented with pulsed electromagnetic fie	No	Other populations/diseases	Other populations/diseases	-
1082	2019	Does occupational therapy delay or reduce the proportion of patients that receives thumb carpom	No	Other populations/diseases	Other populations/diseases	-
1083	2010	Low-frequency electric muscle stimulation combined with physical therapy after total hip osteoa	No	Without intervention/comparator	Without intervention/comparator	-
1084	2018	Hip arthroscopy compared to best conservative care for the treatment of femoroacetabular impin	No	Other populations/diseases	Other populations/diseases	-
1085	1995	Practical management of osteoarthritis. Integration of pharmacologic and nonpharmacologic mea	No	Without intervention/comparator	Without intervention/comparator	-
1086	2014	Manual therapy directed at the knee or lumbopelvic region does not influence quadriceps spinal	No	Other populations/diseases	Other populations/diseases	-
1087	2019	The effects of high- versus moderate-intensity exercise on fatigue in sarcoidosis	No	Other populations/diseases	Other populations/diseases	-
1088	2019	EFFECT OF ADDING LAY-TUTORS TO A BACK SCHOOL PROGRAMME FOR PATIENT	No	Other populations/diseases	Other populations/diseases	-
1089	2015	Train High Eat Low for Osteoarthritis study (THE LO study): protocol for a randomized controll	No	Without intervention/comparator	Without intervention/comparator	-
1090	2018	The knitting community-based trial for older women with osteoarthritis of the hands: design and	No	Without intervention/comparator	Without intervention/comparator	-
1091	2016	The frequency of and patient characteristics associated with fear of movement in adults with syn	No	Without intervention/comparator	Without intervention/comparator	-
1092	2017	Fear of Movement and Associated Factors Among Adults With Symptomatic Knee Osteoarthriti	No	Without intervention/comparator	Without intervention/comparator	-
1093	2016	Comparative study of the Shiva Guggulu and Yeshanada Guggulu in the management of Amava	No	Without intervention/comparator	Without intervention/comparator	-
1094	2017	Evaluation of the effect of HÃ©vÃ-z mud in patients with hand osteoarthritis: a randomized, co	No	Without intervention/comparator	Without intervention/comparator	-
1095	2019	Balance training in virtual reality improves temporal gait parameters in patients after total hip re	No	Without intervention/comparator	Without intervention/comparator	-
1096	2013	Effects of a 6-month physical training program on body composition, BMD and markers of bone	No	Other populations/diseases	Other populations/diseases	-
1097	1999	Dynamic strength training in patients with early rheumatoid arthritis increases muscle strength b	No	Without intervention/comparator	Without intervention/comparator	-

1098	2012	A randomized controlled trial to investigate the effects of water-based exercise to improve falls r	No	Without intervention/comparator	Without intervention/comparator	-
1099	2017	Mediating Effect of Changes in Hand Impairments on Hand Function in Patients With Rheumato	No	Without intervention/comparator	Without intervention/comparator	-
1100	2012	The effects of neuromuscular exercise on medial knee joint load post-arthroscopic partial medial	No	Without intervention/comparator	Without intervention/comparator	-
1101	2015	Neuromuscular Exercise post Partial Medial Meniscectomy: randomized Controlled Trial	No	Without intervention/comparator	Without intervention/comparator	-
1102	2017	Does the association between increased knee muscles strength and improved physical function d	No	Without intervention/comparator	Without intervention/comparator	-
1103	2017	Is the relationship between increased knee muscle strength and improved physical function follo	No	Without intervention/comparator	Without intervention/comparator	-
1104	2018	Does frontal knee kinematics predict treatment outcomes? Exploratory analyses from the Intensiv	No	Without intervention/comparator	Without intervention/comparator	-
1105	2018	Knee extensor strength gains mediate symptom improvement in knee osteoarthritis: secondary an	No	Without intervention/comparator	Without intervention/comparator	-
1106	2018	Moderators of strengthening exercise with pain coping skills training for people with knee osteoa	No	Without intervention/comparator	Without intervention/comparator	-
1107	2016	Contribution of therapeutic education in the treatment of knee osteoarthritis: preliminary results	No	Without intervention/comparator	Without intervention/comparator	-
1108	2016	The impact of self-efficacy on physical activity maintenance in patients with hip osteoarthritis -	No	Without intervention/comparator	Without intervention/comparator	-
1109	2015	Early rehabilitation after total knee replacement surgery: a multicenter, noninferiority, randomize	No	Without intervention/comparator	Without intervention/comparator	-
1110	2020	Anterior-stabilized TKA is inferior to posterior-stabilized TKA in terms of postoperative posterio	No	Without intervention/comparator	Without intervention/comparator	-
1111	2018	Immediate effects of leg-press exercises with tibial internal rotation on individuals with medial k	No	Without intervention/comparator	Without intervention/comparator	-
1112	2018	Immediate effects of legâpress exercises with tibial internal rotation on individuals with medial k	No	Without intervention/comparator	Without intervention/comparator	-
1113	2014	Quadriceps strength, knee pain and functional performance with patellar taping in knee osteoarth	No	Without intervention/comparator	Without intervention/comparator	-
1114	2018	Hangaard, S.	No	Without intervention/comparator	Without intervention/comparator	-
1115	2009	Hans, G.	No	Other topics	Other topics	-
1116	2013	Hansen, T. B.	No	Other topics	Other topics	-
1117	2013	Hansen, T. B.	No	Without intervention/comparator	Without intervention/comparator	-
1118	2010	Effect of an education programme for patients with osteoarthritis in primary care--a randomized	No	Without intervention/comparator	Without intervention/comparator	-
1119	2014	Evaluation of two cane instruments in older adults with knee osteoarthritis	No	Without intervention/comparator	Without intervention/comparator	-
1120	2011	A single bisphosphonate infusion does not accelerate fracture healing in high tibial osteotomies	No	Without intervention/comparator	Without intervention/comparator	-
1121	2018	Improved early outcome after TKA through an app-based active muscle training programme-a ra	No	Without intervention/comparator	Without intervention/comparator	-
1122	2012	The challenges of recruiting patients into a sham surgery trial	No	Without intervention/comparator	Without intervention/comparator	-
1123	2013	Arthroscopic partial meniscectomy in middle-aged patients with mild or no knee osteoarthritis: a	No	Without intervention/comparator	Without intervention/comparator	-
1124	2017	The effectiveness of the use of a digital activity coaching system in addition to a two-week home	No	Without intervention/comparator	Without intervention/comparator	-
1125	2017	The effectiveness of the use of a digital activity coaching system in addition to a two-week home	No	Other populations/diseases	Other populations/diseases	-
**1126**	**2019**	**Blood-flow restriction resistance exercise for older adults with knee osteoarthritis: A pilot**	**Yes**	**Full**	**Full**	**-**
1127	2016	Movement-Pattern Training to Improve Function in People With Chronic Hip Joint Pain: a Feasi	No	Without intervention/comparator	Without intervention/comparator	-
1128	2018	Reduced Hip Adduction Is Associated With Improved Function After Movement-Pattern Trainin	No	Without intervention/comparator	Without intervention/comparator	-
1129	2019	Movement pattern training compared to standard strengthening and flexibility among patients w	No	Without intervention/comparator	Without intervention/comparator	-
1130	2020	Sustained outcomes following movement pattern training or strengthening/flexibility among pati	No	Without intervention/comparator	Without intervention/comparator	-
1131	2015	Tourniquet versus no tourniquet on knee-extension strength early after fast-track total knee arthr	No	Other populations/diseases	Other populations/diseases	-
1132	2018	Prospective, randomized, double blind evaluation of the efficacy of a single dose hyaluronic acid	No	Other populations/diseases	Other populations/diseases	-
1133	2019	Prospective, Randomized, Double-Blind Evaluation of the Efficacy of a Single-Dose Hyaluronic	No	Other populations/diseases	Other populations/diseases	-
1134	2014	The contributions of diet and exercise to improving knee osteoarthritis in overweight adults	No	Without intervention/comparator	Without intervention/comparator	-
1135	2018	Effect of high impact exercise on femoral neck bone mineral density and T2 relaxation times of a	No	Without intervention/comparator	Without intervention/comparator	-
1136	2019	High impact exercise increased femoral neck bone density with no adverse effects on imaging m	No	Without intervention/comparator	Without intervention/comparator	-
1137	2019	High impact exercise increased femoral neck bone density with no adverse effects on imaging m	No	Without intervention/comparator	Without intervention/comparator	-
1138	2013	Community-based exercise program reduces chronic knee pain in elderly Japanese women at hig	No	Without intervention/comparator	Without intervention/comparator	-
1139	1998	The long-term efficacy and tolerability of the new anti-inflammatory agent Zaltoprofen in rheum	No	Without intervention/comparator	Without intervention/comparator	-
1140	2012	The addition of a supervised exercise class to a home exercise programme in the treatment of pat	No	Without intervention/comparator	Without intervention/comparator	-
1141	2013	Does bony hip morphology affect the outcome of treatment for patients with adductor-related gro	No	Without intervention/comparator	Without intervention/comparator	-

1142	2014	Does bony hip morphology affect the outcome of treatment for patients with adductor-related gro	No	Without intervention/comparator	Without intervention/comparator	-
1143	2016	Efficacy of glucosamine compounds phonophoresis in knee osteoarthritis	No	Without intervention/comparator	Without intervention/comparator	-
1144	2009	Eight-year safety follow-up of coronary artery disease patients after local intracoronary VEGF ge	No	Other populations/diseases	Other populations/diseases	-
1145	2014	Mini-midvastus versus mini-medial parapatellar approach for minimally invasive total knee arth	No	Other populations/diseases	Other populations/diseases	-
1146	2015	Quantitative effects of proprioceptive exercises and mulligan's MWM in subjects with osteoarthr	No	Without intervention/comparator	Without intervention/comparator	-
1147	2012	Effect of a walking skill training program in patients who have undergone total hip arthroplasty:	No	Other populations/diseases	Other populations/diseases	-
1148	2015	Exercise, recovery of physical functioning, and prediction of physical activity after total hip arth	No	Other populations/diseases	Other populations/diseases	-
1149	2016	Physical Functioning and Prediction of Physical Activity After Total Hip Arthroplasty: five-Year	No	Other populations/diseases	Other populations/diseases	-
1150	2017	Effect of total knee replacement surgery and postoperative 12 month home exercise program on	No	Other populations/diseases	Other populations/diseases	-
1151	2013	Sarah: strengthening and stretching for people with rheumatoid arthritis of the hands: a randomis	No	Without intervention/comparator	Without intervention/comparator	-
1152	2012	Development and delivery of an exercise intervention for rheumatoid arthritis: strengthening and	No	Without intervention/comparator	Without intervention/comparator	-
1153	2011	High-impact training to improve physical performance in postmenopausal women with early oste	No	Without intervention/comparator	Without intervention/comparator	-
1154	2017	Effects of a 12-week cardiovascular rehabilitation programme on systemic inflammation and tra	No	Without intervention/comparator	Without intervention/comparator	-
1155	2017	The impact of cardiac rehabilitation on cardiovascular risk in individuals with rheumatoid arthri	No	Without intervention/comparator	Without intervention/comparator	-
1156	2010	Exercise increases interleukin-10 levels both intraarticularly and peri-synovially in patients with	No	Without intervention/comparator	Without intervention/comparator	-
1157	2016	The effect of a contextually relevant non-pharmacological intervention on the functioning of Sou	No	Without intervention/comparator	Without intervention/comparator	-
1158	2013	Hand exercises significantly improved activity performance, grip strength and pain in women wi	No	Without intervention/comparator	Without intervention/comparator	-
1159	2014	Effect of home-based hand exercises in women with hand osteoarthritis: a randomised controlled	No	Without intervention/comparator	Without intervention/comparator	-
1160	2015	Effect of home-based hand exercises in women with hand osteoarthritis: a randomised controlled	No	Without intervention/comparator	Without intervention/comparator	-
1161	2016	Changes in ultrasound assessed markers of inflammation following intra-articular steroid injecti	No	Without intervention/comparator	Without intervention/comparator	-
1162	2011	Changes in knee muscle strength and muscle mass after weight loss in obese patients with knee	No	Without intervention/comparator	Without intervention/comparator	-
1163	2012	Cartilage loss during symptomatic maintenance after a clinically significant weight loss in obese	No	Without intervention/comparator	Without intervention/comparator	-
1164	2013	Exercise therapy reduces pain sensitivity in patients with knee osteoarthritis: a randomized contr	No	Without intervention/comparator	Without intervention/comparator	-
1165	2014	Association of exercise therapy and reduction of pain sensitivity in patients with knee osteoarthr	No	Without intervention/comparator	Without intervention/comparator	-
1166	2014	Structural changes in the knee during weight loss maintenance after a significant weight loss in	No	Without intervention/comparator	Without intervention/comparator	-
1167	2014	The effects of exercise therapy on knee joint biomechanics during walking in patients with knee	No	Without intervention/comparator	Without intervention/comparator	-
1168	2015	Evaluation of the benefit of corticosteroid injection before exercise therapy in patients with oste	No	Without intervention/comparator	Without intervention/comparator	-
1169	2015	Exercise versus analgesics for knee osteoarthritis pain: a meta-epidemiological study of cochrane	No	Review/Systematic Review/Meta-Analys	Review/Systematic Review/Meta-Analys	-
1170	2016	Intra-articular corticosteroids in addition to exercise for reducing pain sensitivity in knee osteoar	No	Without intervention/comparator	Without intervention/comparator	-
1171	2020	Analytical approach for determining the suitability of nine cadidate blood markers for investigat	No	Without intervention/comparator	Without intervention/comparator	-
1172	2019	MRI Brain Changes After Marathon Running: Results of the Berlin Beat of Running Study	No	Other populations/diseases	Other populations/diseases	-
1173	2013	Preoperative resistance training increases muscle function in patients diagnosed with hip osteoar	No	Without intervention/comparator	Without intervention/comparator	-
1174	2014	Preoperative effects of progressive explosive-type resistance training in patients with osteoarthrit	No	Without intervention/comparator	Without intervention/comparator	-
1175	2016	Preoperative progressive explosive-type resistance training is feasible and effective in patients w	No	Without intervention/comparator	Without intervention/comparator	-
1176	2012	Effects of a soft thumb base splint in persons with carpometacarpal osteoarthritis: a randomised	No	Without intervention/comparator	Without intervention/comparator	-
1177	2014	Effects of a soft prefabricated thumb orthosis in carpometacarpal osteoarthritis	No	Without intervention/comparator	Without intervention/comparator	-
1178	2019	Efficacy of core exercises in patients with osteoarthritis of the knee: a randomized controlled clin	No	Without intervention/comparator	Without intervention/comparator	-
1179	2018	Influence of disease activity in the physical activity of psoriatic arthritis patients	No	Other populations/diseases	Other populations/diseases	-
1180	2008	Effect of therapeutic exercise for hip osteoarthritis pain: results of a meta-analysis	No	Review/Systematic Review/Meta-Analys	Review/Systematic Review/Meta-Analys	-
1181	2011	A randomised controlled trial investigating the effectiveness of three different modes of exercise	No	Other populations/diseases	Other populations/diseases	-
1182	2013	Is arthroscopic surgery beneficial in treating non-traumatic, degenerative medial meniscal tears?	No	Other populations/diseases	Other populations/diseases	-
1183	2019	The efficacy of 'static' training interventions for improving indices of cardiorespiratory fitness in	No	Other populations/diseases	Other populations/diseases	-
1184	2018	Randomized blinded sham- and waitlist-controlled trial of acupuncture for joint symptoms relate	No	Other populations/diseases	Other populations/diseases	-
1185	2017	Developmentand validation ofanthropo-metric prediction model for estimation of muscle mass in	No	Without intervention/comparator	Without intervention/comparator	-

1186	2012	Postoperative pain and function in patients having a knee arthroscopy with viscosupplementatio	No	Other populations/diseases	Other populations/diseases	-
1187	2009	Balance exercises in arthritis need to be targeted to the individual	No	Without intervention/comparator	Without intervention/comparator	-
1188	2009	Laterally wedged insoles in knee osteoarthritis: do biomechanical effects decline after one month	No	Without intervention/comparator	Without intervention/comparator	-
1189	2012	Lateral wedge insoles for medial knee osteoarthritis: effects on lower limb frontal plane biomech	No	Without intervention/comparator	Without intervention/comparator	-
1190	2014	Unloading shoes for osteoarthritis of the knee: protocol for the SHARK randomised controlled tr	No	Without intervention/comparator	Without intervention/comparator	-
1191	2015	Use of nondrug, nonoperative interventions by community-dwelling people with hip and knee os	No	Without intervention/comparator	Without intervention/comparator	-
1192	2017	"Sounds a Bit Crazy, But It Was Almost More Personal: " A Qualitative Study of Patient and Cli	No	Without intervention/comparator	Without intervention/comparator	-
1193	2017	Telephone-Delivered Exercise Advice and Behavior Change Support by Physical Therapists for	No	Without intervention/comparator	Without intervention/comparator	-
1194	2019	Does telephone-delivered exercise advice and support by physiotherapists improve pain and/or fu	No	Without intervention/comparator	Without intervention/comparator	-
1195	2015	Adverse events in mobility-limited and chronically ill elderly adults participating in an exercise i	No	Without intervention/comparator	Without intervention/comparator	-
1196	2016	Home-Based Exercise Supported by General Practitioner Practices: ineffective in a Sample of Cl	No	Without intervention/comparator	Without intervention/comparator	-
1197	2013	Patient and public involvement (PPI) in informing the osteoarthritis of the thumb therapy (OTTE	No	Without intervention/comparator	Without intervention/comparator	-
1198	2012	A four-week walking exercise programme in patients with knee osteoarthritis improves the abilit	No	Without intervention/comparator	Without intervention/comparator	-
1199	2019	Effects of an Intervention to Improve Life-Space Mobility and Self-Efficacy in Patients following	No	Without intervention/comparator	Without intervention/comparator	-
1200	2016	Prevalence and Determinants of Fatigue Following Total Knee Replacement: a Longitudinal Coh	No	Without intervention/comparator	Without intervention/comparator	-
1201	2001	Physiotherapy in osteoarthritis--a review of literature on conservative therapy of knee and hip os	No	Without intervention/comparator	Without intervention/comparator	-
1202	2019	Quality of surgery and surgical reporting for patients with primary gastrointestinal stromal tumou	No	Other populations/diseases	Other populations/diseases	-
1203	2020	The hip injection trial (HIT) nested qualitative study: experiences of living with hip Osteoarthriti	No	Without intervention/comparator	Without intervention/comparator	-
1204	2010	No difference in knee function or prevalence of osteoarthritis after reconstruction of the anterior	No	Without intervention/comparator	Without intervention/comparator	-
1205	2012	No differences in prevalence of osteoarthritis or function after open versus endoscopic technique	No	Without intervention/comparator	Without intervention/comparator	-
1206	2017	High-intensity strength training in addition to standard non-surgical treatment in patients with kn	No	Without intervention/comparator	Without intervention/comparator	-
1207	2020	Low-dose strength training in addition to neuromuscular exercise and education in patients with	No	Without intervention/comparator	Without intervention/comparator	-
1208	2016	The effect of instruction in analgesic use compared with neuromuscular exercise on knee-joint lo	No	Without intervention/comparator	Without intervention/comparator	-
1209	2016	The effect on knee-joint load of instruction in analgesic use compared with neuromuscular exerc	No	Without intervention/comparator	Without intervention/comparator	-
1210	2017	One year effectiveness of neuromuscular exercise compared with instruction in analgesic use on	No	Without intervention/comparator	Without intervention/comparator	-
1211	2017	The effect of instruction in analgesic use compared with neuromuscular exercise on knee-joint lo	No	Without intervention/comparator	Without intervention/comparator	-
1212	2017	The effect of neuromuscular exercise compared with instruction in analgesic use on knee functio	No	Without intervention/comparator	Without intervention/comparator	-
1213	2018	One year effectiveness of neuromuscular exercise compared with instruction in analgesic use on	No	Without intervention/comparator	Without intervention/comparator	-
1214	2018	Postoperative effects of progressive resistance training prior to total hip arthroplasty e one year o	No	Without intervention/comparator	Without intervention/comparator	-
1215	2010	Bioreconstructive poly-L/D-lactide implant compared with Swanson prosthesis in metacarpopha	No	Without intervention/comparator	Without intervention/comparator	-
1216	2010	Preoperative therapeutic exercise in frail elderly scheduled for total hip replacement: a randomiz	No	Other populations/diseases	Other populations/diseases	-
1217	2019	Acute Anterior Cruciate Ligament Rupture: repair or Reconstruction? Two-Year Results of a Ra	No	Other populations/diseases	Other populations/diseases	-
1218	2015	The effects of a strength and neuromuscular exercise programme for the lower extremity on knee	No	Other populations/diseases	Other populations/diseases	-
1219	2012	Evaluation of the effect of balneotherapy in patients with osteoarthritis of the hands: a randomiz	No	Without intervention/comparator	Without intervention/comparator	-
1220	2016	Relationship between hsTnI and coronary stenosis in asymptomatic women with rheumatoid arth	No	Without intervention/comparator	Without intervention/comparator	-
1221	2013	Follow-up project : study on the impact of physical therapy in etanercept-treated patients	No	Without intervention/comparator	Without intervention/comparator	-
1222	2019	Early post-operative intervention of whole-body vibration in patients after total knee arthroplasty	No	Other populations/diseases	Other populations/diseases	-
1223	2016	Link Between Positive Clinician-Conveyed Expectations of Treatment Effect and Pain Reductio	No	Without intervention/comparator	Without intervention/comparator	-
1224	2012	Short-term effects of 890-nanometer radiation on pain, physical activity, and postural stability in	No	Without intervention/comparator	Without intervention/comparator	-
1225	2020	Effects of a dynamic combined training on impulse response for middle-aged and elderly patient	No	Without intervention/comparator	Without intervention/comparator	-
1226	2010	The frequency and significance of Iron-Deficiency anemia in patients with selected concurrent il	No	Other populations/diseases	Other populations/diseases	-
1227	2016	Recruitment of older adults into randomized controlled trials: issues and lessons learned from tw	No	Other populations/diseases	Other populations/diseases	-
1228	2017	Effects of quadriceps functional exercise with isometric contraction in the treatment of knee oste	No	Without intervention/comparator	Without intervention/comparator	-
1229	2017	The mobile application of patient management in education and follow-up for patients following	No	Without intervention/comparator	Without intervention/comparator	-

1230	2011	Intra-articular injections of sodium hyaluronate (HyalganÂ®) in osteoarthritis of the knee. a ranc	No	Without intervention/comparator	Without intervention/comparator	-
1231	2013	Effect of pre-operative neuromuscular training on functional outcome after total knee replacemer	No	Other populations/diseases	Other populations/diseases	-
1232	2015	Effect of preoperative neuromuscular training (NEMEX-TJR) on functional outcome after total l	No	Other populations/diseases	Other populations/diseases	-
1233	2011	Safety and effects of two mistletoe preparations on production of Interleukin-6 and other immun	No	Other populations/diseases	Other populations/diseases	-
1234	2018	Innovative treatment of clinically diagnosed meniscal tears: a randomized sham-controlled trial c	No	Other populations/diseases	Without intervention/comparator	Other populations/dis
1235	2017	Molecular alterations in skeletal muscle in rheumatoid arthritis are related to disease activity, phy	No	Without intervention/comparator	Without intervention/comparator	-
1236	2019	Low intensity blood flow restriction exercise: Rationale for a hypoalgesia effect	No	Other populations/diseases	Other populations/diseases	-
1237	2017	Blood flow restriction training in clinical musculoskeletal rehabilitation: a systematic review and	No	Review/Systematic Review/Meta-Analys	Review/Systematic Review/Meta-Analys	-
1238	2010	Fit and Strong!: bolstering maintenance of physical activity among older adults with lower-extrer	No	Without intervention/comparator	Without intervention/comparator	-
1239	2012	Efficacy and safety of Antarth for osteoarthritis	No	Without intervention/comparator	Without intervention/comparator	-
1240	2012	Biomarkers of cartilage degradation and synthesis relevant to knee osteoarthritis: relationships w	No	Without intervention/comparator	Without intervention/comparator	-
1241	2013	A physiotherapist-delivered, combined exercise and pain coping skills training intervention for i	No	Without intervention/comparator	Without intervention/comparator	-
1242	2013	Relationships amongst osteoarthritis biomarkers, dynamic knee joint load, and exercise: results f	No	Without intervention/comparator	Without intervention/comparator	-
1243	2018	Clinical and biomechanical changes following a 4-month toe-out gait modification program for p	No	Without intervention/comparator	Without intervention/comparator	-
1244	2008	The Management of Osteoarthritis: an Overview and Call to Appropriate Conservative Treatmen	No	Without intervention/comparator	Without intervention/comparator	-
1245	2012	The intensive diet and exercise for arthritis trial (IDEA): 18-month radiographic and MRI outcor	No	Without intervention/comparator	Without intervention/comparator	-
1246	2018	Effectiveness of a new model of primary care management on knee pain and function in patients	No	Without intervention/comparator	Without intervention/comparator	-
1247	2018	Optimising primary care management of knee osteoarthritis (partner): protocol for a randomised	No	Without intervention/comparator	Without intervention/comparator	-
1248	2010	Maintenance of physical activity after Internet-based physical activity interventions in patients w	No	Without intervention/comparator	Without intervention/comparator	-
1249	2017	Evaluation of recruitment procedures to the self-management of osteoarthritis and low back pain	No	Without intervention/comparator	Without intervention/comparator	-
1250	2017	Physiotherapists' views of the acceptability and feasibility of the self-management of osteoarthrit	No	Without intervention/comparator	Without intervention/comparator	-
1251	2016	Theory-driven group-based complex intervention to support self-management of osteoarthritis ar	No	Without intervention/comparator	Without intervention/comparator	-
1252	2018	Evaluation of the behavioural process and secondary outcomes of the self-management of osteoa	No	Without intervention/comparator	Without intervention/comparator	-
1253	2009	A community-based integrated rehabilitation programme for chronic knee pain	No	Without intervention/comparator	Without intervention/comparator	-
1254	2011	Sustained improvement in physical functioning following an integrated rehabilitation programm	No	Without intervention/comparator	Without intervention/comparator	-
1255	2012	Clinical effectiveness and costs of an integrated rehabilitation programme compared with outpat	No	Without intervention/comparator	Without intervention/comparator	-
1256	2012	Long term costs and cost-effectiveness of an integrated rehabilitation programme for chronic kne	No	Without intervention/comparator	Without intervention/comparator	-
1257	2012	Long-term outcomes and costs of an integrated rehabilitation program for chronic knee pain: a p	No	Without intervention/comparator	Without intervention/comparator	-
1258	2009	Early maximal strength training is an efficient treatment for patients operated with total hip arthr	No	Other populations/diseases	Other populations/diseases	-
1259	2010	Early postoperative maximal strength training improves work efficiency 6-12 months after osteo	No	Without intervention/comparator	Other populations/diseases	Without intervention/c
1260	2018	Randomized controlled trial of maximal strength training vs. standard rehabilitation following to	No	Other populations/diseases	Other populations/diseases	-
1261	2018	Efficacy of pre-operative quadriceps strength training on knee-extensor strength before and short	No	Other populations/diseases	Other populations/diseases	-
1262	2013	The impact of supplemental n-3 long chain polyunsaturated fatty acids and dietary antioxidants c	No	Other populations/diseases	Other populations/diseases	-
1263	2017	The presence of rheumatoid factor is associated with lower bone mass in Korean health screening	No	Without intervention/comparator	Without intervention/comparator	-
1264	2012	Heat stress and cardiovascular, hormonal, and heat shock proteins in humans	No	Other populations/diseases	Other populations/diseases	-
1265	2020	Transcutaneous Electrical Nerve Stimulation Improves Stair Climbing Capacity in People with K	No	Without intervention/comparator	Without intervention/comparator	-
1266	2018	Effect of exercise therapy combined with branched-chain amino acid supplementation on muscle	No	Without intervention/comparator	Without intervention/comparator	-
1267	2012	Inhibition of interleukin-1 activity by anakinra improves endothelial, coronary and aortic functic	No	Without intervention/comparator	Without intervention/comparator	-
1268	2019	Effect of physical therapy on early knee osteoarthritis with medial meniscal posterior tear assess	No	Without intervention/comparator	Without intervention/comparator	-
1269	2014	New field of application of radial shock wave therapy-osteoarthritis	No	Without intervention/comparator	Without intervention/comparator	-
1270	2013	Is neuromuscular electrical stimulation effective for improving pain, function and activities of da	No	Without intervention/comparator	Without intervention/comparator	-
1271	2011	Little clinical advantage of modified Watson-Jones approach over modified mini-incision direct	No	Other populations/diseases	Other populations/diseases	-
1272	2016	Which is the appropriate frequency of TENS in managing knee osteoarthritis: high or low freque	No	Without intervention/comparator	Without intervention/comparator	-
1273	2018	Multicentred randomised controlled trial of an augmented exercise referral scheme using web-ba	No	Without intervention/comparator	Without intervention/comparator	-

1274	2009	Effect of leg muscles exercise on clinical improvement of knee osteoarthritis	No	Without intervention/comparator	Without intervention/comparator	-
1275	2009	Effects of acupuncture, physiotherapy and exercise training on knee arthrosis	No	Without intervention/comparator	Without intervention/comparator	-
1276	2010	Outcome evaluation of total knee arthroplasty among patients with and without previous high tib	No	Without intervention/comparator	Without intervention/comparator	-
1277	2010	The effect of exercise on knee osteoarthritis	No	Without intervention/comparator	Without intervention/comparator	-
1278	2018	The effects of exercise therapy on functional status in people with Carpal Tunnel Syndrome	No	Other populations/diseases	Other populations/diseases	-
1279	2018	The effect of exercise on the balance of patients with knee osteoarthritis	No	Without intervention/comparator	Without intervention/comparator	-
1280	2010	The effect of Swedish massage on knee osteoarthritis	No	Without intervention/comparator	Without intervention/comparator	-
1281	2019	The effects of MWM techniques on the center of pressure sway changes in knee osteoarthritis	No	Without intervention/comparator	Without intervention/comparator	-
1282	2020	Effect of exercise therapy with and without intra-articular hyaluronic acid in knee osteoarthritis	No	Without intervention/comparator	Without intervention/comparator	-
1283	2011	Treatment of anterior knee pain according to risk factor correction	No	Without intervention/comparator	Without intervention/comparator	-
1284	2011	The effect of hippotherapy Yesulator in mechanical low back pain	No	Without intervention/comparator	Without intervention/comparator	-
1285	2019	Therapeutic exercise in knee osteoarthritis	No	Without intervention/comparator	Without intervention/comparator	-
1286	2011	The comparison between Aerobic exercise and progressive resistance exercise effect on Knee ost	No	Without intervention/comparator	Without intervention/comparator	-
1287	2011	Effect of physical activity on weight loss after delivery	No	Without intervention/comparator	Without intervention/comparator	-
1288	2019	Effects of mindfulness-based stress reduction and cognitive behavioral therapy on patients with	No	Without intervention/comparator	Without intervention/comparator	-
1289	2013	Comparison results of two methods of anterior cruciate ligament reconstruction surgery	No	Without intervention/comparator	Without intervention/comparator	-
1290	2012	Effectiveness of Self Care Education on Helplessness	No	Without intervention/comparator	Without intervention/comparator	-
1291	2011	The effect of Action Potential Yesulation and Low Level LASER in Reducing Pain and Improvir	No	Without intervention/comparator	Without intervention/comparator	-
1292	2013	Effectiveness of PRP in treatment of Knee degenerative disorder	No	Without intervention/comparator	Without intervention/comparator	-
1293	2011	Efficacy of Glucosamine in knee osteoarthrosis	No	Without intervention/comparator	Without intervention/comparator	-
1294	2011	the effect of distraction in the treatment of knee arthrosis	No	Without intervention/comparator	Without intervention/comparator	-
1295	2019	The effect of massage in rheumatoid arthritis	No	Without intervention/comparator	Without intervention/comparator	-
1296	2012	Investigation the effect of combination of whole body vibration training and strength exercise tra	No	Without intervention/comparator	Without intervention/comparator	-
1297	2012	Effect of platelet rich plasma on treatment of knee osteoarthritis	No	Without intervention/comparator	Without intervention/comparator	-
1298	2013	Study of the effects of local platelet-rich plasma (PRP) injection versus autologous blood injectio	No	Other populations/diseases	Other populations/diseases	-
1299	2014	Effects of Core Stability Training on performance,Pain and Balance in athletic patients with Chr	No	Without intervention/comparator	Without intervention/comparator	-
1300	2013	Evaluation of the effect of green tea extracts in osteoarthritis	No	Without intervention/comparator	Without intervention/comparator	-
1301	2013	Exercise therapy and Platelet Rich Plasma injection in shoulder pain	No	Other populations/diseases	Other populations/diseases	-
1302	2013	The effect of group education on self-management in patient with rheumatoid arthritis	No	Without intervention/comparator	Without intervention/comparator	-
1303	2014	Tendon/Nerve gliding exercises in carpal tunnel syndrome	No	Other populations/diseases	Other populations/diseases	-
1304	2013	Study of the long term effects of local Platelet-rich plasma (PRP) injection versus Autologous bl	No	Other populations/diseases	Other populations/diseases	-
1305	2018	The effect of dry needling of trigger point in patient with knee OA	No	Without intervention/comparator	Without intervention/comparator	-
1306	2019	Investigation the effect of valgus unloading knee brace on gait harmonic ratio in patients with m	No	Without intervention/comparator	Without intervention/comparator	-
1307	2014	Comparing the effect of Taichi and close kinetic chain exercise on the knee osteoarthritis	No	Without intervention/comparator	Without intervention/comparator	-
1308	2014	Assessment of platelet rich plasma injection in clinical outcome and MRI findings in patients wi	No	Without intervention/comparator	Without intervention/comparator	-
1309	2014	The effects of trunk stabilization exercise on anterior knee pain	No	Without intervention/comparator	Without intervention/comparator	-
1310	2014	A comparison of shock wave versus corticosteroid for pain reduction in plantar fasciitis: random	No	Other populations/diseases	Other populations/diseases	-
1311	2015	Effect of Exercise on gestational diabetes	No	Other populations/diseases	Other populations/diseases	-
1312	2014	Comparison of the effect of two types of exercise therapy in the treatment of shortening of poster	No	Without intervention/comparator	Without intervention/comparator	-
1313	2014	The effect of the shoulder muscles strengthening in treatment of patients with tennis elbow	No	Other populations/diseases	Other populations/diseases	-
1314	2018	The effects of high intensity laser therapy on knee ostoarthrosis	No	Without intervention/comparator	Without intervention/comparator	-
1315	2014	The effect of traction on cervical osteoarthritis symptoms	No	Without intervention/comparator	Without intervention/comparator	-
1316	2016	comparison of two methods of exercise therapy in patellofemoral pain syndrome	No	Other populations/diseases	Other populations/diseases	-
1317	2014	Effect of quadriceps muscle strengthening exercises on the fiber angle ratio of vastus medialis ol	No	Other populations/diseases	Other populations/diseases	-

1318	2015	Effect of Galbanum oil on pain of the knee arthrosis	No	Without intervention/comparator	Without intervention/comparator	-
1319	2015	The comparison of exercise therapy and exercise therapy with posture education effect on neck p	No	Other populations/diseases	Other populations/diseases	-
1320	2015	Self-management program for patients with osteoarthritis	No	Without intervention/comparator	Without intervention/comparator	-
1321	2015	The Healthy Worker Programme	No	Without intervention/comparator	Without intervention/comparator	-
1322	2018	The effect of treadmill exercise in subjects with bilateral knee osteoarthritis	No	Without intervention/comparator	Without intervention/comparator	-
1323	2015	Comparison of the Immediate Effect of Supplementary Cycle Ergometery and Treadmill Walkin	No	Without intervention/comparator	Without intervention/comparator	-
1324	2015	effectiveness of taping in knee osteoarthritis	No	Without intervention/comparator	Without intervention/comparator	-
1325	2015	The self-care education and stabilizing exercise in pain, disability and muscle activities in mothe	No	Other populations/diseases	Other populations/diseases	-
1326	2015	Effectiveness of group exercise therapy with the instructional pack in improvement of patients w	No	Other populations/diseases	Other populations/diseases	-
1327	2018	Examining the topical effectiveness of Jointâs cream on patients with Osteoarthritis of the knee	No	Without intervention/comparator	Without intervention/comparator	-
1328	2015	Comparison of three different types of aerobic exercise program (lower body with and without v	No	Without intervention/comparator	Without intervention/comparator	-
1329	2016	The effectiveness of intermittent vacuum in knee osteoarthritis	No	Without intervention/comparator	Without intervention/comparator	-
1330	2015	Effects of Aerobic Exercise on Hematologic Indices of Women with Rheumatoid Arthritis	No	Without intervention/comparator	Without intervention/comparator	-
1331	2015	The effect of patellar taping and patellar bracing on pain and fuction in females with knee pain	No	Other populations/diseases	Other populations/diseases	-
1332	2015	Effect of orthosis on low back pain	No	Without intervention/comparator	Without intervention/comparator	-
1333	2016	Pilates exercise on symptoms of rheumatoid arthritis patients and lower extremity function	No	Without intervention/comparator	Without intervention/comparator	-
1334	2015	Study of adding aerobic training to acupuncture in treatment of neck myofascial pain syndrome	No	Other populations/diseases	Other populations/diseases	-
1335	2015	Comparison between EMG-Biofeedback controlled exercises and conventional exercises on knee	No	Without intervention/comparator	Without intervention/comparator	-
1336	2015	Effect of orthosis on low back pain	No	Other populations/diseases	Other populations/diseases	-
1337	2016	comparison the effect of laser and exercise on the function and the pain of knee osteoarthritis	No	Without intervention/comparator	Without intervention/comparator	-
1338	2016	The study of the whole body vibration therapy effects on the patients with knee osteoarthritis	No	Without intervention/comparator	Without intervention/comparator	-
1339	2016	The Immediate Effect of Physical Therapy on the Knee Joint Load in Subjects with Knee ?Arthro	No	Without intervention/comparator	Without intervention/comparator	-
1340	2016	THE THERAPEUTIC EFFECT OF INTERFERENTIAL CURRENT AND EXERCISE-THERA	No	Without intervention/comparator	Without intervention/comparator	-
1341	2016	Vibration effect on delayed -onset muscle soreness following exercises	No	Other populations/diseases	Other populations/diseases	-
1342	2016	Comparison of the effects of routine physiotherapy and Pilates exercise on pain, function and qu	No	Without intervention/comparator	Without intervention/comparator	-
1343	2016	Comparing the effects of traditional and sensory-motor training exercises on joint position sense	No	Other populations/diseases	Other populations/diseases	-
1344	2016	The Effectiveness Of Kinesio taping On The Electrodiagnostic Parameters Of Median Nerve An	No	Other populations/diseases	Other populations/diseases	-
1345	2016	comparison of two taping methods in patellofemoral pain syndrome	No	Other populations/diseases	Other populations/diseases	-
1346	2019	The Effect of Education on Self-Care on Quality of Life in Women with Osteoarthritis in Ahvaz	No	Without intervention/comparator	Without intervention/comparator	-
1347	2016	Kinesiotape and knee osteoarthritis	No	Without intervention/comparator	Without intervention/comparator	-
1348	2019	The effect of hydrotherapy on balance in patients with grade 2 and 3 knee osteoarthritis: a rando	No	Without intervention/comparator	Without intervention/comparator	-
1349	2016	Effectiveness of proprioceptive neuromuscular facilitation and stabilization exercises on cross se	No	Other populations/diseases	Other populations/diseases	-
1350	2016	Effects of Resistance Exercise Training on Postmenopausal Women with Knee Osteoarthritis	No	Without intervention/comparator	Without intervention/comparator	-
1351	2016	The effect of ulnar styloid fracture fixation in treatment of radius fracture	No	Other populations/diseases	Other populations/diseases	-
1352	2016	The effect of education on quality of life and stress in patients with intestinal stoma	No	Other populations/diseases	Other populations/diseases	-
1353	2017	The effect of isokinetic exercise in patieuts with knee osteoarthritis	No	Without intervention/comparator	Without intervention/comparator	-
1354	2018	The Effectiveness of self-care skills training on self-esteem and quality of life in children with st	No	Other populations/diseases	Other populations/diseases	-
1355	2017	Effectiveness of Extra corporal Shock Wave therapy in the treatment of Knee Arthritis	No	Without intervention/comparator	Without intervention/comparator	-
1356	2016	The effect of exercise on lumbar and hip joints coordination in patients with chronic low back pa	No	Other populations/diseases	Other populations/diseases	-
1357	2016	Comparison of the effects of ShockWave Therapy and physiothreapy in teratment of muscular pa	No	Other populations/diseases	Other populations/diseases	-
1358	2016	Interval exercise training and the mechanism of reduced blood pressure	No	Other populations/diseases	Other populations/diseases	-
1359	2016	Effect of laser therapy in patients with osteoarthritis of the knee	No	Without intervention/comparator	Without intervention/comparator	-
1360	2017	The effect of foot reflexology massage and stretching exercises in patients with rheumatoid arthr	No	Without intervention/comparator	Without intervention/comparator	-
1361	2017	The effect of core training using unstable sitting in patients with patrllofemoral pain syndrome	No	Other populations/diseases	Other populations/diseases	-

1362	2018	The effect of resistance training and nano curcumin supplementation on synovial fluid biomarke	No	Other populations/diseases	Other populations/diseases	-
1363	2019	Reviews of the effectiveness of the learning-based mobile application (mobile app) to improve p	No	Without intervention/comparator	Without intervention/comparator	-
1364	2017	The Effect of High Power Laser Therapy on Knee Osteoarthritis	No	Without intervention/comparator	Without intervention/comparator	-
1365	2017	Effect of Group Psychotherapy in Patients With Rheumatoid Arthritis	No	Without intervention/comparator	Without intervention/comparator	-
1366	2017	Knee osteoarthritis rehabilitation	No	Without intervention/comparator	Without intervention/comparator	-
1367	2018	Effect of High power Laser in Knee Osteoarthritis	No	Without intervention/comparator	Without intervention/comparator	-
1368	2017	Comparison of an etiologic-based exercise and conventional strengthening exercises on knee loa	No	Without intervention/comparator	Without intervention/comparator	-
1369	2017	evaluating the effects of aerobic exercise on severity of symptoms and quality of life on female v	No	Other populations/diseases	Other populations/diseases	-
1370	2017	Effects of Exercise on Hemodialysis Patients	No	Other populations/diseases	Other populations/diseases	-
1371	2017	The effect of rehabilitation on patients with carpal tunnel syndrome after surgery	No	Other populations/diseases	Other populations/diseases	-
1372	2017	Effect of Biofeedback on Knee Osteoarthritis	No	Without intervention/comparator	Without intervention/comparator	-
1373	2018	The effects of high voltage pulse current on pain, effusion and range oh motion in subjects with	No	Other populations/diseases	Other populations/diseases	-
1374	2019	The effects of laser therapy on symptoms of patients with knee osteoarthritis	No	Without intervention/comparator	Without intervention/comparator	-
1375	2017	The Effect of Exercise on the Knee Injury and Osteoarthritis Outcomes of Elders	No	Without intervention/comparator	Without intervention/comparator	-
1376	2017	effectiveness of subcotaneous injection of piroxicam compared with oral use of piroxicam in pai	No	Without intervention/comparator	Without intervention/comparator	-
1377	2017	The Effects of Core Stabilization on swissball and Suspended Exercises on Chronic Low Back P	No	Other populations/diseases	Other populations/diseases	-
1378	2019	Effect of Backward Gait on Biomechanical Walking Parameters in Patients with Knee Osteoarth	No	Without intervention/comparator	Without intervention/comparator	-
1379	2017	the effect of walking with reverse heel shoes on improving knee osteoarthritis symptoms	No	Without intervention/comparator	Without intervention/comparator	-
1380	2017	spinal orthotic management of the elderly people with hyperkyphosis	No	Other populations/diseases	Other populations/diseases	-
1381	2017	The effects of aloe vera gel on pain and function among patients with knee osteoarthritis	No	Without intervention/comparator	Without intervention/comparator	-
1382	2018	The Effect of âChair Yogaâ on knee Joints pain &physical Function, Balance and Fear of Falling	No	Without intervention/comparator	Without intervention/comparator	-
1383	2017	The effect of vestibular exercises on fatigue in patients with parkinsonâs disease	No	Other populations/diseases	Other populations/diseases	-
1384	2018	Effect of Joint protection Treatment on the Elderly's OsteoArthritis	No	Without intervention/comparator	Without intervention/comparator	-
1385	2017	The Effect of Home-based Rehabilitation on the Treatment of Knee Qsteoarthritis	No	Without intervention/comparator	Without intervention/comparator	-
1386	2017	Efficacy of high power laser in treatment of patients with patellar chondromalacia	No	Other populations/diseases	Other populations/diseases	-
1387	2019	effect of taping on medial meniscal injury	No	Other populations/diseases	Other populations/diseases	-
1388	2018	"The effect of walking on sexual function in women with rheumatoid arthritis" ; "Evaluation of s	No	Without intervention/comparator	Without intervention/comparator	-
1389	2018	effectiveness of prolotherapy in reduce pain and functional improvements of osteoarthritis patier	No	Without intervention/comparator	Without intervention/comparator	-
1390	2018	The effect of Tai Chi exercise and Glucosamine supplementation on prevention of fall in the eld	No	Without intervention/comparator	Without intervention/comparator	-
1391	2018	The effect of kinesio tape on knee proprioception, pain , balance and quality of life in patients w	No	Without intervention/comparator	Without intervention/comparator	-
1392	2018	Effect of Mulligan Mobilization Technique on Balance in Knee Osteoarthritis	No	Without intervention/comparator	Without intervention/comparator	-
1393	2019	Magnet therapy ,acupuncture and isometric exercise in treatment knee osteoarthritis	No	Without intervention/comparator	Without intervention/comparator	-
1394	2019	effect of high intensity laser therapy on knee osteoarthritis	No	Without intervention/comparator	Without intervention/comparator	-
1395	2018	The Effect of transcranial direct stimulationof different brain cortex areas plus the traditional ph	No	Without intervention/comparator	Without intervention/comparator	-
1396	2019	TRX training vs. aquatic therapy for patients with kneeosteoarthritis	No	Without intervention/comparator	Without intervention/comparator	-
1397	2019	The Effect of High TENS Intensity on Knee Osteoarthritis	No	Without intervention/comparator	Without intervention/comparator	-
1398	2019	Effectiveness of a home-based exercise program via the mobile application (Varzeshyar) on pain	No	Without intervention/comparator	Without intervention/comparator	-
1399	2019	Comparison of the effect of intra-articular hypertonic saline, intra-articular HIALGAN and physi	No	Without intervention/comparator	Without intervention/comparator	-
1400	2019	The effect of exercise on osteoarthritis	No	Without intervention/comparator	Without intervention/comparator	-
1401	2019	MUSCLE ENERGY TECHNIQUE AND MAITLAND MOBILIZATIONS IN TREATING CHR	No	Other populations/diseases	Other populations/diseases	-
1402	2019	Compare the effect of TDCS with TENS on osteoarthritis of the knee	No	Without intervention/comparator	Without intervention/comparator	-
1403	2019	Effect of Hip Muscles Strengthening Exercises on Anterior Knee Pain	No	Other populations/diseases	Other populations/diseases	-
1404	2019	Extra corporeal shock wave therapy for chronic Low back pain	No	Without intervention/comparator	Without intervention/comparator	-
1405	2015	Randomized exercise trial of aromatase inhibitor-induced arthralgia in breast cancer survivors	No	Other populations/diseases	Other populations/diseases	-

1406	2018	Integrating participatory ergonomic management in non-weight-bearing exercise and progressive	No	Without intervention/comparator	Without intervention/comparator	-
1407	2018	Effects of supervised exercise in older population having osteoarthritis of knee: a randomised co	No	Without intervention/comparator	Without intervention/comparator	-
1408	2019	Effects of activity modification in older population having osteoarthritis of knee: a randomized c	No	Without intervention/comparator	Without intervention/comparator	-
1409	2002	A prospective randomised trial to examine the therapeutic effects of hydrotherapy in chronic rhe	No	Without intervention/comparator	Without intervention/comparator	-
1410	2002	Effectiveness of occupational therapy (OT) intervention with patients with early stage rheumatoi	No	Without intervention/comparator	Without intervention/comparator	-
1411	2002	Four year follow-up of a randomised controlled trial of joint protection for people with rheumato	No	Without intervention/comparator	Without intervention/comparator	-
1412	2003	A prospective, randomised, clinical trial comparing trapezial denervation with trapeziectomy for	No	Without intervention/comparator	Without intervention/comparator	-
1413	2003	A randomised controlled trial of intensive physiotherapy vs a home-based exercise treatment pro	No	Without intervention/comparator	Without intervention/comparator	-
1414	2003	Education programmes for people with arthritis: a comparative study	No	Without intervention/comparator	Without intervention/comparator	-
1415	2003	The effect of joint mobilisations on physiotherapy outcomes in patients with osteoarthritis (OA)	No	Without intervention/comparator	Without intervention/comparator	-
1416	2004	A comparison of general and specific dynamic stability exercises in osteoarthritis of the carpome	No	Without intervention/comparator	Without intervention/comparator	-
1417	2004	A controlled trial of shared care for chronic rheumatoid arthritis	No	Without intervention/comparator	Without intervention/comparator	-
1418	2004	A pragmatic randomised controlled trial of hydrotherapy and land exercises on global well-being	No	Without intervention/comparator	Without intervention/comparator	-
1419	2004	Application of static magnetic fields versus copper for the relief of pain in osteoarthritis: a rando	No	Without intervention/comparator	Without intervention/comparator	-
1420	2004	Comparison of post-discharge physiotherapy versus usual care following total knee replacement:	No	Other populations/diseases	Other populations/diseases	-
1421	2004	Development and evaluation of an education programme in rheumatoid arthritis: impact on comp	No	Without intervention/comparator	Without intervention/comparator	-
1422	2004	Pilot randomised controlled trial of an early rehabilitation class versus outpatient physiotherapy	No	Other populations/diseases	Other populations/diseases	-
1423	2004	Rehabilitation of Patients with Rheumatoid Arthritis (RA): changes in Muscle, Functional and B	No	Without intervention/comparator	Without intervention/comparator	-
1424	2005	A randomised controlled trial of acupunture, supervised exercise therapy and non intervention in	No	Without intervention/comparator	Without intervention/comparator	-
1425	2005	A randomized, controlled study of intra-articular injections of etanercept or glucocorticosteroids	No	Without intervention/comparator	Without intervention/comparator	-
1426	2005	Effective physiotherapy management of hip osteoarthritis: a preliminary trial	No	Without intervention/comparator	Without intervention/comparator	-
1427	2006	Physical Activity in Rheumatoid Arthritis: a randomised controlled multi-centre study	No	Without intervention/comparator	Without intervention/comparator	-
1428	2006	Randomised controlled trial for the treatment of hand arthrosis with prednisolone and naproxen	No	Without intervention/comparator	Without intervention/comparator	-
1429	2006	Randomised controlled trial to compare the functional outcome of the standard NexGen Legacy	No	Other populations/diseases	Other populations/diseases	-
1430	2006	Rheumatoid patients with wrist synovitis, if prescribed wrist splints do better in terms of grip str	No	Without intervention/comparator	Without intervention/comparator	-
1431	2006	The efficacy of wrist working splints in patients with non-destructive wrist arthritis	No	Without intervention/comparator	Other populations/diseases	Without intervention/c
1432	2006	Computer assisted surgery versus conventional arthroscopic anterior cruciate ligament reconstru	No	Other populations/diseases	Other populations/diseases	-
1433	2007	Functional and work outcomes improve in patients with Rheumatoid Arthritis (RA) who receive	No	Without intervention/comparator	Without intervention/comparator	-
1434	2007	Improving rehabilitation for patients with chronic knee pain	No	Without intervention/comparator	Other populations/diseases	Without intervention/c
1435	2007	In vivo longitudinal evaluation of vertebral bone strength in patients with rheumatoid arthritis tr	No	Without intervention/comparator	Without intervention/comparator	-
1436	2007	Oral cetylated fatty acid for the improvement of function, quality of life, and pain in patients wit	No	Without intervention/comparator	Without intervention/comparator	-
1437	2007	Total value of intensive exercise training (three weeks) immediately after hospital discharge for a	No	Without intervention/comparator	Without intervention/comparator	-
1438	2008	A dynamic exercise programme to improve patients' disability in rheumatoid arthritis: a prospect	No	Without intervention/comparator	Without intervention/comparator	-
1439	2008	Education and eXercise Training in early Rheumatoid Arthritis (EXTRA) study	No	Without intervention/comparator	Without intervention/comparator	-
1440	2008	Functional outcome in two different designs of knee replacements	No	Other populations/diseases	Other populations/diseases	-
1441	2008	High Intensity Training in patients with rheumatoid arthritis	No	Other populations/diseases	Other populations/diseases	-
1442	2009	Effectiveness of exercise therapy in hip osteoarthritis	No	Without intervention/comparator	Other populations/diseases	Without intervention/c
1443	2009	Feasibility of a therapeutic exercise before total hip replacement	No	Other populations/diseases	Other populations/diseases	-
1444	2009	Neuromuscular electrical stimulation of the quadriceps muscle: a novel alternative to total knee r	No	Other populations/diseases	Other populations/diseases	-
1445	2010	Neuromuscular electrical stimulation of the quadriceps muscle: a novel alternative to total knee r	No	Other populations/diseases	Other populations/diseases	-
1446	2010	Educational material on cardiovascular disease in rheumtoid arthritis (RA)	No	Without intervention/comparator	Without intervention/comparator	-
1447	2010	Individualising exercise for knee pain: developing an evidence-based impairment-targeted interv	No	Without intervention/comparator	Without intervention/comparator	-
1448	2011	Does thumb joint mobilization decrease hyperalgesia in elderly patients with secondary thumb ca	No	Without intervention/comparator	Without intervention/comparator	-
1449	2011	Facilitating Activity and Self management in Arthritis (FASA)	No	Without intervention/comparator	Without intervention/comparator	-

1450	2011	Hypoalgesic and motor effects of Kaltenborn mobilization on elderly patients with secondary thu	No	Without intervention/comparator	Without intervention/comparator	-
1451	2011	Optimising patient function following elective Total Hip Replacement (THR) surgery	No	Other populations/diseases	Other populations/diseases	-
1452	2012	Acupuncture in a group setting for chronic knee pain: scrutiKnee	No	Other populations/diseases	Other populations/diseases	-
1453	2012	Bilateral sensory and motor effects of unilateral combined treatment in patients with carpo-meta	No	Without intervention/comparator	Without intervention/comparator	-
1454	2012	Interdisciplinary Innovative model of care for people with Hand OsteoArthritis	No	Without intervention/comparator	Without intervention/comparator	-
1455	2012	The AGIR program ? A self-management program for patients with arthrosis including educatio	No	Without intervention/comparator	Without intervention/comparator	-
1456	2013	Can regenerative photonic therapy improve pain and mobility in knee osteoarthritis?	No	Without intervention/comparator	Without intervention/comparator	-
1457	2013	Creatine supplementation to treat "muscle wasting" in rheumatoid arthritis patients	No	Without intervention/comparator	Without intervention/comparator	-
1458	2013	Meniscal Transplantation and its Effect on Osteoarthritis Risk: a clinical trial of meniscal transp	No	Other populations/diseases	Other populations/diseases	-
1459	2013	Supervision preference for an effective walking program among older individuals with osteoarth	No	Without intervention/comparator	Without intervention/comparator	-
1460	2013	The efficacy of Low Level Laser Therapy (LLLT) in knee osteoarthritis	No	Without intervention/comparator	Without intervention/comparator	-
1461	2014	Efficacy of a topical cannabinoid preparation in decreasing symptoms of rheumatoid arthritis	No	Without intervention/comparator	Without intervention/comparator	-
1462	2014	Improving outcomes for patients with rheumatoid arthritis with intermediate disease - is intensiv	No	Without intervention/comparator	Without intervention/comparator	-
1463	2014	Total vs Robotic bi-UniCompartmental Knee (TRUCK)Trial	No	Other populations/diseases	Other populations/diseases	-
1464	2015	A multi-centred randomised trial to assess if adding web-based support to exercise referral schen	No	Without intervention/comparator	Without intervention/comparator	-
1465	2015	COmmunity-based Rehabilitation after Knee Arthroplasty (CORKA)	No	Other populations/diseases	Other populations/diseases	-
1466	2015	Osteoarthritis project	No	Without intervention/comparator	Without intervention/comparator	-
1467	2015	Prevention and rehabilitation of osteoporotic fractures In disadvantaged populations 2 â subproje	No	Other populations/diseases	Other populations/diseases	-
1468	2015	The effect of home electrical stimulation and intra-operative femoral nerve blockage on outcome	No	Other populations/diseases	Other populations/diseases	-
1469	2016	Activity orientated rehabilitation following knee arthroplasty: feasibility study	No	Other populations/diseases	Other populations/diseases	-
1470	2016	Chondroitin sulphate for hand osteoarthritis: a randomised, placebo-controlled trial in primary c	No	Without intervention/comparator	Without intervention/comparator	-
1471	2016	Efficacy of preoperative aquatic resistance training on muscle power and knee symptoms in pers	No	Without intervention/comparator	Without intervention/comparator	-
1472	2016	Osteoarthritis thumb therapy trial II	No	Without intervention/comparator	Without intervention/comparator	-
1473	2016	The effectiveness of adding arm exercise programme following hip replacement in the elderly	No	Other populations/diseases	Other populations/diseases	-
1474	2016	The REACT (retirement in action) study	No	Other populations/diseases	Other populations/diseases	-
1475	2017	A 12-week digital care program for chronic knee pain involving exercise, education, and psycho	No	Other populations/diseases	Other populations/diseases	-
1476	2017	A randomized controlled trial on effects of Cryo/Cuff in early rehabilitation of total knee arthrop	No	Other populations/diseases	Other populations/diseases	-
1477	2017	A study to evaluate the potential effectiveness of a dietary supplement for osteoarthritis	No	Without intervention/comparator	Without intervention/comparator	-
1478	2017	Balance exercises help rehabilitation after hip fracture	No	Other populations/diseases	Other populations/diseases	-
1479	2017	Comparison of the JOURNEY II Bi-Cruciate Stabilised and GENESIS II Total Knee Arthroplast	No	Other populations/diseases	Other populations/diseases	-
1480	2017	Peer mentorship in osteoarthritis	No	Without intervention/comparator	Without intervention/comparator	-
1481	2017	Pain and function of patients with knee osteoarthritis were improved after receiving therapeutic	No	Without intervention/comparator	Without intervention/comparator	-
1482	2017	Peer mentorship in osteoarthritis	No	Without intervention/comparator	Without intervention/comparator	-
1483	2017	Results after anterior cruciate ligament reconstruction using the quadriceps tendon with or witho	No	Other populations/diseases	Other populations/diseases	-
1484	2017	The Gait Rehabilitation in Early Arthritis Trial	No	Without intervention/comparator	Without intervention/comparator	-
1485	2017	What are the effects of balance exercises in patients' following total knee replacement ability to	No	Other populations/diseases	Other populations/diseases	-
1486	2018	Does exercise help greater trochanter pain syndrome?	No	Other populations/diseases	Other populations/diseases	-
1487	2018	Effects of modified drainage on total knee arthroplasty	No	Other populations/diseases	Other populations/diseases	-
1488	2018	IL-6 inhibition in patients with depression and low-grade inflammation: the Insight study	No	Other populations/diseases	Other populations/diseases	-
1489	2018	Is there a role for acupuncture in basal thumb arthritis? A randomised controlled trial of real vers	No	Without intervention/comparator	Without intervention/comparator	-
1490	2018	Osteoarthritis preoperative package for care of orthotics, rehabilitation, topical and oral agent us	No	Without intervention/comparator	Without intervention/comparator	-
1491	2018	Physical, physiological and biochemical effects of AmLexin following exercise in healthy runne	No	Other populations/diseases	Other populations/diseases	-
1492	2018	PROvision of braces for Patients with knee OsteoArthritis (PROP OA): a randomised controlled	No	Without intervention/comparator	Without intervention/comparator	-
1493	2018	The effect of the Mulligan mobilization with movement approach following knee replacement su	No	Other populations/diseases	Other populations/diseases	-

1494	2019	A cycling and education programme in the treatment of hip osteoarthritis	No	Without intervention/comparator	Without intervention/comparator	-
1495	2019	Can we use estrogen-containing therapy to improve pain in women after menopause with hand o	No	Without intervention/comparator	Without intervention/comparator	-
1496	2019	Effects of MaitlandÃ¢â¬â¢s Mobilization on modulating biomarkers in knee osteoarthritis	No	Without intervention/comparator	Without intervention/comparator	-
1497	2019	Electrical Stimulation treating knee osteoarthritis for pain, function and strength	No	Without intervention/comparator	Without intervention/comparator	-
1498	2019	Feasibility of TRAK to support physio in anterior cruciate ligament rehabilitation	No	Without intervention/comparator	Without intervention/comparator	-
1499	2015	Within-day variation and influence of physical exercise on circulating Galectin-3 in patients witl	No	Without intervention/comparator	Without intervention/comparator	-
1500	2000	Can a program of manual physical therapy and supervised exercise improve the symptoms of ost	No	Without intervention/comparator	Without intervention/comparator	-
1501	2014	Correlations between functional status and the quality of life in patients with knee osteoarthritis	No	Without intervention/comparator	Without intervention/comparator	-
1502	2016	Physical examination findings and their relationship with performance-based function in adults v	No	Without intervention/comparator	Without intervention/comparator	-
1503	2016	Sociodemographic and clinical correlates of physical therapy utilization in adults with symptoma	No	Without intervention/comparator	Without intervention/comparator	-
1504	2020	Initiating range of motion exercises within 24 hours following total knee arthroplasty affects the	No	Without intervention/comparator	Without intervention/comparator	-
1505	2009	Preventative effect of exercise against falls in the elderly: a randomized controlled trial	No	Without intervention/comparator	Without intervention/comparator	-
1506	2017	Patterns of routine primary care for osteoarthritis in the UK: a cross-sectional electronic health re	No	Other populations/diseases	Other populations/diseases	-
1507	2000	Use of glucocorticoids in rheumatoid arthritis	No	Without intervention/comparator	Without intervention/comparator	-
1508	2019	Effect of 16-week corrective training program on three dimensional joint moments of the domina	No	Other populations/diseases	Other populations/diseases	-
1509	2014	Hypertonic dextrose versus corticosteroid local injection for the treatment of osteoarthritis in the	No	Other populations/diseases	Other populations/diseases	-
1510	2019	Efficacy of balance training in combination with physical therapy in rehabilitation of knee osteo	No	Without intervention/comparator	Without intervention/comparator	-
1511	2018	The Effect of Prehabilitation on Postoperative Outcome in Patients Following Primary Total Kne	No	Without intervention/comparator	Without intervention/comparator	-
1512	?	10.1186/s13613-016-0114-z	No	Other topics	Other topics	-
1513	2014	Early progressive strength training to enhance recovery after fast-track total knee arthroplasty: a	No	Other populations/diseases	Other populations/diseases	-
1514	2011	Nitroglycerin improves bone mineral density, bone geometry and bone strength: results from a tv	No	Other populations/diseases	Other populations/diseases	-
1515	2016	The Effectiveness of Occupational Therapy Supervised Usage of Adaptive Devices on Functiona	No	Other populations/diseases	Other populations/diseases	-
1516	2010	High and low-intensity resistance training for postmenopausal bone: an updated meta-analysis	No	Review/Systematic Review/Meta-Analys	Review/Systematic Review/Meta-Analys	-
1517	2010	Efficacy ofanexercise programon the functional capacity and disease activity in females with rhe	No	Without intervention/comparator	Without intervention/comparator	-
1518	2012	Effect of exercise on cardiac autonomic function in females with rheumatoid arthritis	No	Without intervention/comparator	Without intervention/comparator	-
1519	2018	Exercise may decrease syncope secondary to postural change in females with rheumatoid arthriti	No	Without intervention/comparator	Without intervention/comparator	-
1520	2020	Changes in inflammation and musculoskeletal tissue-derived biomarker serum levels in response	No	Without intervention/comparator	Without intervention/comparator	-
1521	2014	Arthroscopic partial meniscectomy vs sham surgery for degenerative meniscus tear	No	Other populations/diseases	Other populations/diseases	-
1522	2020	The therapeutic effects of anti-gravity treadmill walking in individuals with knee osteoarthritis	No	Without intervention/comparator	Without intervention/comparator	-
1523	2009	Short-term outcomes of trapeziometacarpal artelon implant compared with tendon suspension in	No	Without intervention/comparator	Without intervention/comparator	-
1524	2017	The efficacy of early initiated, supervised, progressive resistance training compared to unsupervi	No	Other populations/diseases	Other populations/diseases	-
1525	2019	Equivalent hip stem fixation by Hi-Fatigue G and Palacos Râ+âG bone cement: a randomized ra	No	Other populations/diseases	Other populations/diseases	-
1526	2014	Effect of eccentric isokinetic strengthening in the rehabilitation of patients with knee osteoarthri	No	Without intervention/comparator	Without intervention/comparator	-
1527	2014	A randomized, controlled, prospective study evaluating the effect of patellar eversion on functio	No	Other populations/diseases	Other populations/diseases	-
1528	2009	Effects of dietary intervention and quadriceps strengthening exercises on pain and function in ov	No	Without intervention/comparator	Without intervention/comparator	-
1529	2011	Recovery in mechanical muscle strength following resurfacing vs standard total hip arthroplasty	No	Other populations/diseases	Other populations/diseases	-
1530	2012	Recovery in horizontal gait after hip resurfacing vs. total hip arthroplasty at 6-month follow-up -	No	Other populations/diseases	Other populations/diseases	-
1531	2013	The effect of education and supervised exercise vs. education alone on the time to total hip repla	No	Without intervention/comparator	Without intervention/comparator	-
1532	2014	Therapeutic effect viscosuplementa and local infiltration of corticosteroid in patients with knee c	No	Without intervention/comparator	Without intervention/comparator	-
1533	2009	Long-term clinical benefits and costs of an integrated rehabilitation programme compared with c	No	Without intervention/comparator	Without intervention/comparator	-
1534	2017	A comparative study on the efficacy of Maitland's mobilisation and Mulligan's mobilisation in su	No	Without intervention/comparator	Without intervention/comparator	-
1535	2019	Cobalt-chromium particles inducing preosteoblasts may aggravate periprosthetic inflammation	No	Other populations/diseases	Other populations/diseases	-
1536	2016	Effect of External Applying Compound Tripterygium wilfordii Hook F. on Joint Pain of Rheuma	No	Without intervention/comparator	Without intervention/comparator	-
1537	2019	Visual Arts Education improves self-esteem for persons with dementia and reduces caregiver bu	No	Other populations/diseases	Other populations/diseases	-

1538	2012	Effects of weekly and fortnightly therapeutic exercise on physical function and health-related qu	No	Without intervention/comparator	Without intervention/comparator	-
1539	2013	Effects of Nordic walking in the community dwelling subjects with hip osteoarthritis	No	Without intervention/comparator	Without intervention/comparator	-
1540	2014	Effects of education and strength training on functional tests among older people with osteoarthr	No	Without intervention/comparator	Without intervention/comparator	-
1541	2018	Virtual reality intervention in postoperative rehabilitation after total knee arthroplasty: a prospec	No	Other populations/diseases	Other populations/diseases	-
1542	2017	A randomized clinical trial assessment of nonsteroidal anti-inflammatory drugs and Chinese bon	No	Without intervention/comparator	Without intervention/comparator	-
1543	2015	Effectiveness of balance exercises in the acute post-operative phase following total hip and knee	No	Without intervention/comparator	Without intervention/comparator	-
1544	2016	Force-plate analyses of balance following a balance exercise program during acute post-operativ	No	Other populations/diseases	Other populations/diseases	-
1545	2012	A randomized controlled trial of a cognitive behavioural patient education intervention versus a	No	Without intervention/comparator	Without intervention/comparator	-
1546	2011	Dynamic splinting for postoperative hallux limitus: a randomized, controlled trial	No	Other populations/diseases	Other populations/diseases	-
1547	2018	Bayesian synthesis using prior information on fracture risk from randomized trials to analyze po:	No	Other populations/diseases	Other populations/diseases	-
1548	2017	A randomized controlled effectiveness trial of the enhance-fitnessÂ® physical activity program i	No	Without intervention/comparator	Without intervention/comparator	-
1549	2019	Protein Supplementation Does Not Augment Adaptations to Endurance Exercise Training	No	Other populations/diseases	Other populations/diseases	-
1550	2017	Effect of a model consultation informed by guidelines on recorded quality of care of osteoarthriti	No	Other populations/diseases	Other populations/diseases	-
1551	2015	Progressive resistance exercise in women with osteoarthritis of the knee: a randomized controlle	No	Without intervention/comparator	Without intervention/comparator	-
1552	2017	The efficacy of early initiated, supervised, progressive resistance training compared to unsupervi	No	Other populations/diseases	Other populations/diseases	-
1553	2019	Effect of retrowalking, a non-pharmacological treatment on pain, disability, balance and gait in l	No	Without intervention/comparator	Without intervention/comparator	-
1554	2013	Effectiveness of exercise with or without thermal therapy for community-dwelling elderly Japane	No	Other populations/diseases	Other populations/diseases	-
1555	2017	Development of evaluation scale and exercise program reflecting the clinical symptoms of knee	No	Without intervention/comparator	Without intervention/comparator	-
1556	2019	Total REview of Anti-Sclerostin antibody Use Related to Elderly people (TREASURE) study	No	Other populations/diseases	Other populations/diseases	-
1557	2019	Efficiency of GliSODin	No	Other populations/diseases	Other populations/diseases	-
1558	2019	Efficiency of V.D or V.E on osteoarthritis	No	Without intervention/comparator	Without intervention/comparator	-
1559	2005	Effect of Home Exercise of Quadriceps for Knee Osteoarthritis; A Randomized Con-trolled Trial	No	Without intervention/comparator	Without intervention/comparator	-
1560	2009	Multi-institutional prospective randomized controlled trial to compare the effects of home-based	No	Without intervention/comparator	Without intervention/comparator	-
1561	2009	Open labeled clinical research on utility of pentosan polysulfate(pentosan) to osteoarthritis of the	No	Without intervention/comparator	Without intervention/comparator	-
1562	2010	Effects of Water-based Exercise Program among the Community-Dwelling Elderly with Lower L	No	Other populations/diseases	Other populations/diseases	-
1563	2011	Effect of home-based quadriceps muscle strengthening and range of motion exercises for knee o:	No	Without intervention/comparator	Without intervention/comparator	-
1564	2011	Effects of COX-2 selective inhibitor and hyaluronan acid to prevent cartilage degeneration in pa	No	Without intervention/comparator	Without intervention/comparator	-
1565	2011	Influence of the home-based excecise of the patient with knee osteoarthritis by Cox2-Inhibitoror	No	Without intervention/comparator	Without intervention/comparator	-
1566	2011	The examination about the performance and safe operation of the Motivative exercise evaluation	No	Other populations/diseases	Other populations/diseases	-
1567	2012	Clinical study on the analgesic efficacy of a 4-week combination of NSAIDs patch and celecoxil	No	Without intervention/comparator	Without intervention/comparator	-
1568	2012	Effect of body weight support walking on exercise capacity and walking speed in patients with k	No	Without intervention/comparator	Without intervention/comparator	-
1569	2012	Postoperative quadriceps strength of continuous femoral ropivacaine or levobupivacaine: a prosp	No	Without intervention/comparator	Without intervention/comparator	-
1570	2012	Randomized controlled trial of integrative medicine program for self management on fibromyalg	No	Other populations/diseases	Other populations/diseases	-
1571	2012	The effect of microcurrent electrical neuromuscular stimulation after total knee arthroplasty	No	Other populations/diseases	Other populations/diseases	-
1572	2013	Additive effect of tidal knee irrigation and exercise for osteoarthritis of the knee	No	Without intervention/comparator	Without intervention/comparator	-
1573	2013	Evaluation of physical therapy on physical function and QOL in patients with RA	No	Without intervention/comparator	Without intervention/comparator	-
1574	2013	Postoperative quadriceps strength of continuous or patient-controlled femoral nerve block: a pro:	No	Without intervention/comparator	Without intervention/comparator	-
1575	2013	The effects of physical/occupational therapy after operations of the upper extremities on RA pati	No	Without intervention/comparator	Without intervention/comparator	-
1576	2013	The effects of sensory level neuromuscular electrical stimulation in patients after Total knee arth	No	Other populations/diseases	Other populations/diseases	-
1577	2013	The multicenter randomized controlled trial on the effectiveness of the medial side single hinge k	No	Without intervention/comparator	Without intervention/comparator	-
1578	2014	Clinical trial on the efficacy and safety of corrective osteotomy for the upper extremity using cus	No	Other populations/diseases	Other populations/diseases	-
1579	2014	Combined effects of nutritional supplement (agaro-oligosaccharides) intake and exercise training	No	Without intervention/comparator	Without intervention/comparator	-
1580	2014	Combined intervention of exercise program with pain coping skills training among the elderly w	No	Other populations/diseases	Other populations/diseases	-
1581	2015	Assessment of the effectiveness of a custom-made functional splint by three dimensionally cutin	No	Without intervention/comparator	Without intervention/comparator	-

1582	2015	Clinical application of robot suit HAL for joint disease	No	Other populations/diseases	Other populations/diseases	-
1583	2015	Clinical studies of the osteotomy for osteoarthritis of knee	No	Without intervention/comparator	Without intervention/comparator	-
1584	2015	Clinical trial of balance exercise "Assist"	No	Other populations/diseases	Other populations/diseases	-
1585	2015	Does the ROM-ex start timing effect the outcome and swelling after TKA ? -A Prospective Study	No	Other populations/diseases	Other populations/diseases	-
1586	2015	Effect of branched chain amino acid with muscle strength exercise on patients with hip osteoartl	No	Without intervention/comparator	Without intervention/comparator	-
1587	2015	Effect of instruction using measurement of physical activity on postoperative outcome in total jo	No	Other populations/diseases	Other populations/diseases	-
1588	2015	Effects of body weight supported walking exersise using lower body positive pressure device on	No	Other populations/diseases	Other populations/diseases	-
1589	2015	Effects of reaching-exercise system with motor-assisted unloading device for the upper paretic ar	No	Other populations/diseases	Other populations/diseases	-
1590	2015	Factors associated with perioperative physical activity in patients with hip osteoarthritis	No	Without intervention/comparator	Without intervention/comparator	-
1591	2015	Safety and efficacy of the single joint type of Hybrid Assistive Limb (sj-HAL), a device for any a	No	Without intervention/comparator	Without intervention/comparator	-
1592	2016	Analysis of association among quadriceps femoris muscle anatomy, knee extension strength and	No	Without intervention/comparator	Without intervention/comparator	-
1593	2016	Development of active exercise auxiliary equipment for robot suit HAL Single-Joint type	No	Without intervention/comparator	Without intervention/comparator	-
1594	2016	Effect of difference of decubitus position in abducting the hip on function after hip replacement	No	Other populations/diseases	Other populations/diseases	-
1595	2016	Effect of muscle strength training using electrical stimulation on postoperative patients	No	Other populations/diseases	Other populations/diseases	-
1596	2016	Effects of combined intervention of exercise and nutrient on physical function in knee osteoarthi	No	Without intervention/comparator	Without intervention/comparator	-
1597	2016	Effects of High- and Low-velocity Resistance Training on Gait Kinematics and Kinetics in Indiv	No	Without intervention/comparator	Without intervention/comparator	-
1598	2016	Effects of insoles is given to the mechanical stress on the knee joint during walking and running	No	Other populations/diseases	Other populations/diseases	-
1599	2016	Efficacy of ultrasound-guided pulsed radiofrequency treatment on the saphenous nerve in knee c	No	Other populations/diseases	Other populations/diseases	-
1600	2016	Immediate effects of leg press exercises with tibial internal rotation on symptoms and function o	No	Without intervention/comparator	Without intervention/comparator	-
1601	2016	Prospective comparison study on efficacy between physiotherapy and kinesitherapy for knee ost	No	Without intervention/comparator	Without intervention/comparator	-
1602	2016	Randomized controlled trial about the lymph drainage intervention in the physiotherapy after the	No	Without intervention/comparator	Without intervention/comparator	-
1603	2016	The effect of timing of exercise with olive fruit extract intake on cognitive performance and mot	No	Other populations/diseases	Other populations/diseases	-
1604	2016	The qualitative evaluation of the gluteus minimus muscle by the electromyography and examinat	No	Other populations/diseases	Other populations/diseases	-
1605	2016	Verification of the efficacy of collagen peptide for the purpose of flail-Locomotive Syndrome Pr	No	Other populations/diseases	Other populations/diseases	-
1606	2017	A study on a new rehabilitation program after total hip arthroplasty	No	Other populations/diseases	Other populations/diseases	-
1607	2017	Development of gene-based exercise and nutrition program for preventing locomotive syndrome	No	Other populations/diseases	Other populations/diseases	-
1608	2017	Effect of exercise therapy with tibial internal-rotation on clinical outcomes and knee kinematics	No	Without intervention/comparator	Without intervention/comparator	-
1609	2017	Effect of High-intensity and Short Term Preoperative Rehabilitation in TKA Patients	No	Other populations/diseases	Other populations/diseases	-
1610	2017	Effect of tibial internal-rotation exercise and insole on osteoarthiritis of the knee	No	Without intervention/comparator	Without intervention/comparator	-
1611	2017	Effectiveness of High Intensity Exercises for improvement of Stair Climbing in Patients Underg	No	Other populations/diseases	Other populations/diseases	-
1612	2017	Effectiveness of pain coping skills training in inpatients Undergone Total Knee Arthroplasty : ra	No	Other populations/diseases	Other populations/diseases	-
1613	2017	Effectiveness of Virtual Reality Balance Exercises in Patients Undergone Knee Joint Replaceme	No	Other populations/diseases	Other populations/diseases	-
1614	2017	Intraarticular injections of synovial stem cells for osteoarthritis of the knee	No	Without intervention/comparator	Without intervention/comparator	-
1615	2017	Investigation regarding effects of PSTR(Pericapsular soft tissue and realignment) exercises to im	No	Without intervention/comparator	Without intervention/comparator	-
1616	2017	Postoperative Evaluation Using Different Tourniquet Cuff Pressure in Yesultaneous Bilateral To	No	Other populations/diseases	Other populations/diseases	-
1617	2017	The effect of exercise preconditioning with blood flow restriction on ischemia reperfusion injury	No	Other populations/diseases	Other populations/diseases	-
1618	2017	The effect of high velocity trunk exercise on gait ability of patients after total hip arthroplasty	No	Other populations/diseases	Other populations/diseases	-
1619	2017	The effect of n-3 unsaturated fatty acids supplementation on quadriceps muscle weakness after t	No	Other populations/diseases	Other populations/diseases	-
1620	2017	The effect of toe grip strenth on the prognosis of patients with total knee arthroplasty	No	Other populations/diseases	Other populations/diseases	-
1621	2017	The effects of electrical muscle stimulation during walking on kinematics of knee and physical fu	No	Other populations/diseases	Other populations/diseases	-
1622	2017	The efficacy of Quadratus Lumborum Block as a postoperative analgesia for Total Hip Arthropla	No	Other populations/diseases	Other populations/diseases	-
1623	2017	The related factors of independent cane gait after total hip arthroplasty(THA)-Initiating cane gait	No	Other populations/diseases	Other populations/diseases	-
1624	2017	The study of effective training method in early rehabilitation after knee arthroplasty: effects of qu	No	Other populations/diseases	Other populations/diseases	-
1625	2018	Comparative study of physiotherapy program after total knee arthroplasty	No	Other populations/diseases	Other populations/diseases	-

1626	2018	Effect of early neuromuscular electrical stimulation on quadriceps muscle after total knee Arthro	No	Other populations/diseases	Other populations/diseases	-
1627	2018	Effect of exercise differences on hip abductor muscle strength during the early stage after THA	No	Other populations/diseases	Other populations/diseases	-
1628	2018	Effect of Preoperative Rehabilitation in TKA Patients	No	Other populations/diseases	Other populations/diseases	-
1629	2018	Effectiveness of Supervised Exercise Therapy for Community-dwelling People with Chronic Kn	No	Without intervention/comparator	Other populations/diseases	Other populations/dis
1630	2018	Is there a difference in recurrence of knee pain between the Mechanical Diagnosis and Therapy a	No	Without intervention/comparator	Without intervention/comparator	-
1631	2018	The comparison of analgesia and quadriceps strength between intermittent bolus and continuous	No	Other populations/diseases	Other populations/diseases	-
1632	2018	The effect of nutrition treatment for rectus femoris after total knee arthroplasty -randomized cont	No	Other populations/diseases	Other populations/diseases	-
1633	2018	The study of new bone substitute (AFFINOS) in bone osteoconductivity: bone microstructure an	No	Other populations/diseases	Other populations/diseases	-
1634	2019	Analysis of knee rotatory instability as a development factor of knee osteoarthritis using 3D ima	No	Without intervention/comparator	Without intervention/comparator	-
1635	2019	Combination effect of exercise instruction and biologics on rheumatoid arthritis patients. -a rand	No	Without intervention/comparator	Without intervention/comparator	-
1636	2019	Effects of pain neuroscience education in hospitalized patients with pain catastrophizing underg	No	Other populations/diseases	Other populations/diseases	-
1637	2019	Efficacy of 4 weeks preoperative exercise with blood flow restriction on ischemia reperfusion inj	No	Other populations/diseases	Other populations/diseases	-
1638	2019	The effects of Acceptance and Commitment Therapy among older outpatients with knee pain: a	No	Other populations/diseases	Other populations/diseases	-
1639	2019	Incorporating Specific Functional Strength Integration Techniques to Improve Functional Perfor	No	Other populations/diseases	Other populations/diseases	-
1640	2011	A pragmatic randomized controlled study of the effectiveness and cost consequences of exercise	No	Without intervention/comparator	Without intervention/comparator	-
1641	2019	Liuzijue Qigong: a Voice Training Method For Unilateral Vocal Fold Paralysis Patients	No	Other populations/diseases	Other populations/diseases	-
1642	2018	Comparison of three modes of aerobic exercise combined with resistance training on the pain an	No	Without intervention/comparator	Without intervention/comparator	-
1643	2016	Effect of treatment on back pain and back extensor strength with a spinal orthosis in elderly wo	No	Other populations/diseases	Other populations/diseases	-
1644	2017	Localized type Volkmann's contracture treated with tendon transfer and tension-reduced early m	No	Other populations/diseases	Other populations/diseases	-
1645	2018	Overcoming challenges for exercise in the clinic, participation and adherence by patients with os	No	Without intervention/comparator	Without intervention/comparator	-
1646	2014	Results from a single center, double-blind, randomized, placebo-controlled, parallel-group study	No	Other populations/diseases	Other populations/diseases	-
1647	2019	Efficacy of proprioception training for tibiofemoral arthritis in relation with pain and functional	No	Other populations/diseases	Other populations/diseases	-
1648	2010	Effectiveness of aquatic exercise and balneotherapy: a summary of systematic reviews based on	No	Review/Systematic Review/Meta-Analy	Review/Systematic Review/Meta-Analys	-
1649	2013	Magnetotherapy in hand osteoarthritis: a pilot trial	No	Without intervention/comparator	Without intervention/comparator	-
1650	2018	Study of effectiveness of lateral wedge insole in medial compartment of osteoarthritis of knee tre	No	Other populations/diseases	Other populations/diseases	-
1651	2018	Effects of a finger exercise program on hand function in automobile workers with hand osteoart	No	Without intervention/comparator	Without intervention/comparator	-
1652	2015	Glucosamine-containing supplement improves locomotor functions in subjects with knee pain: a	No	Other populations/diseases	Other populations/diseases	-
1653	2012	The effectiveness of a self-management program on quality of life for knee osteoarthritis (OA) p	No	Without intervention/comparator	Without intervention/comparator	-
1654	2016	Gait Using Pneumatic Brace for End-Stage Knee Osteoarthritis	No	Without intervention/comparator	Without intervention/comparator	-
1655	2015	The effectiveness of ultrasound treatment for the management of knee osteoarthritis: a randomiz	No	Without intervention/comparator	Without intervention/comparator	-
1656	2014	Efficacy of passive extension mobilization in addition to exercise in the osteoarthritic knee: an o	No	Without intervention/comparator	Without intervention/comparator	-
1657	2018	How does spa treatment affect cardiovascular function and vascular endothelium in patients wit	No	Without intervention/comparator	Other populations/diseases	Without intervention/c
1658	2019	Application of heat and a home exercise program for pain and function levels in patients with kn	No	Without intervention/comparator	Without intervention/comparator	-
1659	2017	The contribution of the palmaris longus muscle to the strength of wrist flexion and extension	No	Other populations/diseases	Other populations/diseases	-
1660	2019	Assesing the effects of pulsed ultrasound treatment on pain, functionality, synovial fluid and car	No	Without intervention/comparator	Without intervention/comparator	-
1661	2019	A mixedâmethods, randomized controlled feasibility trial of Eye Movement Desensitization and	No	Other populations/diseases	Other populations/diseases	-
1662	2015	Effects of preoperative group-based aquatic training on health related quality of life in persons w	No	Without intervention/comparator	Without intervention/comparator	-
1663	2015	Role of gracilis harvesting in four-strand hamstring tendon anterior cruciate ligament reconstruc	No	Other populations/diseases	Other populations/diseases	-
1664	2019	Aquatic Exercise Program for Individuals With Osteoarthritis: pain, Stiffness, Physical Function	No	Without intervention/comparator	Without intervention/comparator	-
1665	2017	The efficacy of peloid therapy in management of hand osteoarthritis: a pilot study	No	Without intervention/comparator	Without intervention/comparator	-
1666	2013	Arthroscopic partial meniscectomy was not more effective than physical therapy for meniscal tea	No	Without intervention/comparator	Without intervention/comparator	-
1667	2015	Predictors and outcomes of cross-over to surgery in a randomized trial of surgery vs. physical th	No	Without intervention/comparator	Without intervention/comparator	-
1668	2018	Physical Activity to Reduce Fatigue in Rheumatoid Arthritis: a Randomized Controlled Trial	No	Without intervention/comparator	Without intervention/comparator	-
1669	2020	CC4 COST-EFFECTIVENESS ANALYSIS OF THE STEPPED EXERCISE PROGRAM FOR I	No	Without intervention/comparator	Without intervention/comparator	-

1670	2010	Multidisciplinary rehabilitation after primary total knee arthroplasty: a randomized controlled stu	No	Other populations/diseases	Other populations/diseases	-
1671	2015	Comparison of two different mobilization techniques in the management of osteoarthritis of the l	No	Without intervention/comparator	Without intervention/comparator	-
1672	2017	Does Kinesio Taping of the Knee Improve Pain and Functionality in Patients with Knee Osteoart	No	Without intervention/comparator	Without intervention/comparator	-
1673	2018	A comparison of two manual physical therapy approaches and electrotherapy modalities for pati	No	Without intervention/comparator	Without intervention/comparator	-
1674	2017	HPR efficacy of different types of exercise programs in osteoporosis with high risk of falls	No	Other populations/diseases	Other populations/diseases	-
1675	2019	Moving through predeath grief: psychological support for family caregivers of people with deme	No	Other populations/diseases	Other populations/diseases	-
1676	2014	Effect of surgical closing in total knee arthroplasty at flexion or extension: a prospective, randon	No	Other populations/diseases	Other populations/diseases	-
1677	2010	The effect of additional therapeutic ultrasound in patients with primary hip osteoarthritis: a rand	No	Without intervention/comparator	Without intervention/comparator	-
1678	2018	Effects of isokinetic, isometric, and aerobic exercises on clinical variables and knee cartilage vol	No	Without intervention/comparator	Without intervention/comparator	-
1679	2009	Effect of neuromuscular electrical stimulation on pain and functional parameters in knee osteoar	No	Without intervention/comparator	Without intervention/comparator	-
1680	2017	Impact loading following quadriceps strength training in individuals with medial knee osteoarth	No	Without intervention/comparator	Without intervention/comparator	-
1681	2015	Individualized physiotherapy in the treatment of patellofemoral pain	No	Other populations/diseases	Other populations/diseases	-
1682	2015	A longitudinal assessment of the responsiveness of the ICECAP-A in a randomised controlled tri	No	Other populations/diseases	Other populations/diseases	-
1683	2007	Testing of the preliminary OMERACT validation criteria for a biomarker to be regarded as refle	No	Without intervention/comparator	Without intervention/comparator	-
1684	2016	Whole body electromyostimulation and protein to fight sarcopenic obesity in women 70 years an	No	Other populations/diseases	Other populations/diseases	-
1685	2019	Clinical validation of fully automated segmentation of thigh muscle and adipose tissue cross sect	No	Other populations/diseases	Other populations/diseases	-
1686	2018	A pilot randomised clinical trial of physiotherapy (manual therapy, exercise, and education) for e	No	Without intervention/comparator	Without intervention/comparator	-
1687	2015	A phase II trial for the efficacy of physiotherapy intervention for early-onset hip osteoarthritis: st	No	Without intervention/comparator	Without intervention/comparator	-
1688	2014	A pilot randomized control trial of aerobic cycling before total knee arthroplasty	No	Without intervention/comparator	Other populations/diseases	Without intervention/c
1689	2007	Inpatient and outpatient rehabilitation for patients with rheumatoid arthritis: a clinical and econo	No	Without intervention/comparator	Without intervention/comparator	-
1690	2019	EFFICACY OF BELT ELECTRODE SKELETAL MUSCLE ELECTRICAL STIMULATION O	No	Other populations/diseases	Other populations/diseases	-
1691	2017	Is home-based, high-intensity interval training cycling feasible and safe for patients with knee os	No	Without intervention/comparator	Without intervention/comparator	-
1692	2018	Willingness of older adults to participate in a randomized trial of conservative therapies for knee	No	Other populations/diseases	Other populations/diseases	-
1693	2006	Does psychiatric treatment help patients with intractable chronic pain?	No	Other populations/diseases	Other populations/diseases	-
1694	2018	The effectiveness of platelet rich plasma, hyaluronic acid and peptide injections in knee osteoart	No	Without intervention/comparator	Without intervention/comparator	-
1695	2015	Effect of Nigella sativa oil extract on selected immuno cell markers and oxidative stress in rheun	No	Without intervention/comparator	Without intervention/comparator	-
1696	2017	A pilot study of a minimally supervised home exercise and walking program for people with Par	No	Other populations/diseases	Other populations/diseases	-
1697	2018	Effect of diacerin vs glucosaminechondroitin on disease progression, and measures of function i	No	Without intervention/comparator	Without intervention/comparator	-
1698	2018	A comparative study of the effects of lateral wedge insole shoe modification on the patients with	No	Without intervention/comparator	Without intervention/comparator	-
1699	2019	Comparison of the effect of intraarticular hyaluronic acid and effect of naproxen sodium in patie	No	Without intervention/comparator	Without intervention/comparator	-
1700	2012	A comparison of a less invasive piriformis-sparing approach versus the standard posterior appro	No	Other populations/diseases	Other populations/diseases	-
1701	2016	A comparative study of the effects of lateral wedge insole shoe modification on the patients with	No	Without intervention/comparator	Without intervention/comparator	-
1702	2017	Use of art as therapeutic intervention for enhancement of hand function in patients with rheumat	No	Without intervention/comparator	Without intervention/comparator	-
1703	2016	Immunomodulatory Effect of Nigella sativa Oil on T Lymphocytes in Patients with Rheumatoid	No	Without intervention/comparator	Without intervention/comparator	-
1704	2014	High-intensity versus low-level laser therapy in the treatment of patients with knee osteoarthritis	No	Without intervention/comparator	Without intervention/comparator	-
1705	2017	Platelet autologous plasma in osteoarthritis treatment	No	Without intervention/comparator	Without intervention/comparator	-
1706	2016	The effectiveness of exercise therapy based on sahrmann approach in patients with patella-femor	No	Other populations/diseases	Other populations/diseases	-
1707	2015	Pelvic movements are restored to reference values during stair climbing but not during stepping	No	Other populations/diseases	Other populations/diseases	-
1708	2018	Cost-utility analysis of interventions to improve effectiveness of exercise therapy for adults with	No	Without intervention/comparator	Without intervention/comparator	-
1709	2019	Home-Based Exercise With Blood Flow Restriction to Improve Quadriceps Muscle and Physical	No	Other populations/diseases	Other populations/diseases	-
1710	2013	Comparison of the effectiveness of isometric and isokinetic exercise in patients with osteoarthrit	No	Without intervention/comparator	Without intervention/comparator	-
1711	2020	Comparison of the effectiveness of isokinetic exercise vs isometric exercise performed at differe	No	Without intervention/comparator	Without intervention/comparator	-
1712	2013	Effectiveness of exercise with or without thermal therapy for community-dwelling elderly Japane	No	Other populations/diseases	Other populations/diseases	-
1713	1995	A Comparision Between Bucillamine And D-Penicillamine In The Treatmnt Of Rheumatoid Art	No	Without intervention/comparator	Without intervention/comparator	-

1714	2012	The effectiveness of an aquarobic exercise program for patients with osteoarthritis	No	Without intervention/comparator	Without intervention/comparator	-
1715	2011	The effectiveness of minimally invasive total knee arthroplasty to preserve quadriceps strength: a	No	Other populations/diseases	Other populations/diseases	-
1716	2020	Effect of a Physical Activity Promoting Program Based on the IMB Model on Obese-Metabolic I	No	Without intervention/comparator	Without intervention/comparator	-
1717	2018	The association of microbial translocation and WOMAC function in patients with knee osteoartl	No	Without intervention/comparator	Without intervention/comparator	-
1718	2016	Ultrashort versus Conventional Anatomic Cementless Femoral Stems in the Same Patients Youn	No	Other populations/diseases	Other populations/diseases	-
1719	2017	Proteomic response following acute anterior cruciate ligament injury: implications for post-traum	No	Without intervention/comparator	Without intervention/comparator	-
1720	2016	Hydroxychloroquine is not effective in reducing symptoms of hand osteoarthritis: results from a	No	Without intervention/comparator	Without intervention/comparator	-
1721	2018	Hydroxychloroquine effectiveness in reducing symptoms of hand osteoarthritis a randomized tria	No	Without intervention/comparator	Without intervention/comparator	-
1722	2016	Exercise therapy versus arthroscopic partial meniscectomy for degenerative meniscal tear in mid	No	Other populations/diseases	Other populations/diseases	-
1723	2019	Complex Tears, Extrusion, and Larger Excision Are Prognostic Factors for Worse Outcomes 1 a	No	Other populations/diseases	Other populations/diseases	-
1724	2019	The 6-m timed hop test is a prognostic factor for outcomes in patients with meniscal tears treated	No	Other populations/diseases	Other populations/diseases	-
1725	2019	Five-year efficacy and safety of asfotase alfa therapy for adults and adolescents with hypophospl	No	Other populations/diseases	Other populations/diseases	-
1726	2016	Does occupational therapy reduce the need for surgery in carpometacarpal osteoarthritis? Protoc	No	Without intervention/comparator	Without intervention/comparator	-
1727	2019	Improving Physical Activity Through Adjunct Telerehabilitation Following Total Knee Arthropla	No	Other populations/diseases	Other populations/diseases	-
1728	2018	Cost-effectiveness of a blended physiotherapy intervention in patients with hip and/or knee osteo	No	Without intervention/comparator	Without intervention/comparator	-
1729	2014	Effectiveness and cost-effectiveness of a blended exercise intervention for patients with hip and/	No	Without intervention/comparator	Without intervention/comparator	-
1730	2016	Blended physical activity intervention with reduced face-to-face contact and usual physical thera	No	Without intervention/comparator	Without intervention/comparator	-
1731	2016	Blended intervention with reduced face-to-face contact and usual physiotherapy show Yesilar ef	No	Without intervention/comparator	Without intervention/comparator	-
1732	2018	Cost-effectiveness of a blended physiotherapy intervention compared to usual physiotherapy in p	No	Without intervention/comparator	Without intervention/comparator	-
1733	2018	Effectiveness of a Blended Physical Therapist Intervention in People With Hip Osteoarthritis, Kr	No	Without intervention/comparator	Without intervention/comparator	-
1734	2020	Physiotherapists' experiences with a blended osteoarthritis intervention: a mixed methods study	No	Without intervention/comparator	Without intervention/comparator	-
1735	2014	Pain in activity evaluation (PACE) in knee osteoarthritis-responsiveness and concurrent validity	No	Without intervention/comparator	Without intervention/comparator	-
1736	2016	Dynamic weight-bearing assessment of pain in knee osteoarthritis: construct validity, responsive	No	Without intervention/comparator	Without intervention/comparator	-
1737	2016	Randomized, placebo-controlled trial to evaluate clinical efficacy and structure modifying prope	No	Without intervention/comparator	Without intervention/comparator	-
1738	2015	Targeting motivation and self-regulation to increase physical activity among patients with rheum	No	Without intervention/comparator	Without intervention/comparator	-
1739	2016	Explaining Physical Activity Maintenance After a Theory-Based Intervention Among Patients W	No	Without intervention/comparator	Without intervention/comparator	-
1740	2018	Facilitating physical activity and reducing symptoms in patients with knee osteoarthritis: study p	No	Without intervention/comparator	Without intervention/comparator	-
1741	2012	Knee joint stabilization therapy in patients with osteoarthritis of the knee: a randomized, controll	No	Without intervention/comparator	Without intervention/comparator	-
1742	2013	Biomechanical mechanisms underlying treatment effects of exercise therapy in patients with kne	No	Without intervention/comparator	Without intervention/comparator	-
1743	2013	Knee joint stabilization therapy in patients with osteoarthritis of the knee: a randomized, controll	No	Without intervention/comparator	Without intervention/comparator	-
1744	2014	Importance of adherence in outcome of exercise therapy in patients with knee osteoarthritis	No	Without intervention/comparator	Without intervention/comparator	-
1745	2014	Is the severity of knee osteoarthritis on magnetic resonance imaging associated with outcome of	No	Without intervention/comparator	Without intervention/comparator	-
1746	2014	Knee joint stabilization therapy in patients with osteoarthritis of the knee and knee instability: su	No	Without intervention/comparator	Without intervention/comparator	-
1747	2015	Improvement in upper leg muscle strength underlies beneficial effects of exercise therapy in kne	No	Without intervention/comparator	Without intervention/comparator	-
1748	2019	Stratified exercise therapy compared with usual care by physical therapists in patients with knee	No	Without intervention/comparator	Without intervention/comparator	-
1749	2019	Social conversation skill improvements associated with the Social Tools And Rules for Teens pr	No	Other populations/diseases	Other populations/diseases	-
1750	2009	Effect of lateral-wedge shoe insoles on pain and function in patients with knee osteoarthritis	No	Without intervention/comparator	Without intervention/comparator	-
1751	2014	Physicaltherapy in treatmentof patients with glenohumeral osteoarthritis	No	Without intervention/comparator	Without intervention/comparator	-
1752	2013	The efficacy of tens in patients with lateral epicondylitis	No	Other populations/diseases	Other populations/diseases	-
1753	2016	The Patient's Perception Does Not Differ Following Subvastus and Medial Parapatellar Approacl	No	Without intervention/comparator	Without intervention/comparator	-
1754	2017	Effects of a brief action and coping planning intervention on completion of preventive exercises	No	Without intervention/comparator	Without intervention/comparator	-
1755	2015	Effects of Exercise on Patellar Cartilage in Women with Mild Knee Osteoarthritis	No	Without intervention/comparator	Without intervention/comparator	-
1756	2015	CLINICAL SCIENCES. Effects of Exercise on Patellar Cartilage in Women with Mild Knee Ost	No	Without intervention/comparator	Without intervention/comparator	-
1757	2016	Effective Treatment for Rapid Improvement of Both Disease Activity and Self-Reported Physica	No	Without intervention/comparator	Without intervention/comparator	-

1758	2013	Increased pain sensitivity but normal function of exercise induced analgesia in hip and knee oste	No	Without intervention/comparator	Without intervention/comparator	-
1759	2000	Comparative effects of aquatic recreational and aquatic exercise programs on mobility, pain perc	No	Without intervention/comparator	Without intervention/comparator	-
1760	2010	Efficacy of symptomatic control of knee osteoarthritis with 0.0125% of capsaicin versus placebo	No	Without intervention/comparator	Without intervention/comparator	-
1761	2018	Effectiveness of hip mobility exercise in patients with osteoarthritis knee	No	Without intervention/comparator	Without intervention/comparator	-
1762	2013	Long-term effects of video-based home exercise on clinical and radiographic outcomes in subjec	No	Without intervention/comparator	Without intervention/comparator	-
1763	2019	Superior knee flexor strength at 2 years with all-inside short-graft anterior cruciate ligament reco	No	Other populations/diseases	Other populations/diseases	-
1764	2012	The effect of sulphurous water in patients with osteoarthritis of hand. Double-blind, randomized	No	Without intervention/comparator	Without intervention/comparator	-
1765	2016	Effects of sulfur bath on hip osteoarthritis: a randomized, controlled, single-blind, follow-up tria	No	Without intervention/comparator	Without intervention/comparator	-
1766	2012	The effect of sulphurous water in patients with osteoarthritis of hand. Double-blind, randomized	No	Without intervention/comparator	Without intervention/comparator	-
1767	2016	Effects of sulfur bath on hip osteoarthritis: a randomized, controlled, single-blind, follow-up tria	No	Without intervention/comparator	Without intervention/comparator	-
1768	2014	Exercise therapy in hip osteoarthritis--a randomized controlled trial	No	Without intervention/comparator	Without intervention/comparator	-
1769	2011	Efficacy of conservative treatment regimes for hip osteoarthritis--evaluation of the therapeutic ex	No	Without intervention/comparator	Without intervention/comparator	-
1770	2014	Randomised controlled trial: sham treatment shows Yesilar effects on pain and function compar	No	Without intervention/comparator	Without intervention/comparator	-
1771	2016	Effectiveness and efficiency of an 11-week exercise intervention for patients with hip or knee os	No	Without intervention/comparator	Without intervention/comparator	-
1772	2017	International Society of Sports Nutrition position stand: safety and efficacy of creatine suppleme	No	Other populations/diseases	Other populations/diseases	-
1773	2018	Injection site reactions and injection site pain for the adalimumab bioYesilar ABP 501: results fr	No	Without intervention/comparator	Without intervention/comparator	-
1774	2019	Use of paraffin instead of lukewarm water prior to hand exercises had no additional effect on har	No	Other populations/diseases	Other populations/diseases	-
1775	2019	Low-dose prednisolone in patients with hand osteoarthritis (HOPE): results from a randomised d	No	Without intervention/comparator	Without intervention/comparator	-
1776	2010	No benefit of the two-incision THA over mini-posterior THA: a pilot study of strength and gait	No	Without intervention/comparator	Without intervention/comparator	-
1777	2011	No strength or gait benefit of two-incision THA: a brief followup at 1 year	No	Without intervention/comparator	Without intervention/comparator	-
1778	2019	Moderate-to-high intensity exercise with person-centered guidance influences fatigue in older ad	No	Without intervention/comparator	Without intervention/comparator	-
1779	2013	Analysis of effectiveness of therapeutic exercise for knee osteoarthritis and possible factors affec	No	Without intervention/comparator	Without intervention/comparator	-
1780	2016	Comparison of conventional physical therapy modalities and kinesiotaping therapies' effects on a	No	Without intervention/comparator	Without intervention/comparator	-
1781	2017	Effects of weight-loss on patellofemoral loading in overweight and obese adults with patellofem	No	Without intervention/comparator	Without intervention/comparator	-
1782	2018	Increase in patellofemoral loading is not associated with worsening symptoms or structure: secor	No	Without intervention/comparator	Without intervention/comparator	-
1783	2018	Efficacy of a biomechanically-based yoga exercise program in knee osteoarthritis: a randomized	No	Without intervention/comparator	Without intervention/comparator	-
1784	2019	Is four-week underwater treadmill exercise regimen compared to home exercise efficacious for p	No	Without intervention/comparator	Without intervention/comparator	-
1785	2017	The effects of therapeutic exercises on pain, muscle strength, functional capacity, balance and h	No	Without intervention/comparator	Without intervention/comparator	-
1786	2011	Benefits of starting rehabilitation within 24 hours of primary total knee arthroplasty: randomized	No	Without intervention/comparator	Without intervention/comparator	-
1787	2015	Proof of concept study of the arthritis health journal: an online tool to promote self-monitoring i	No	Without intervention/comparator	Without intervention/comparator	-
1788	2016	Proof of concept study of the arthritis health journal: an online tool to promote self-monitoring i	No	Without intervention/comparator	Without intervention/comparator	-
1789	2018	Does care by a multidisciplinary team improve outcomes in rheumatoid arthritis? a randomized	No	Without intervention/comparator	Without intervention/comparator	-
1790	2017	Effects of whole body vibration exercise on neuromuscular function for individuals with knee os	No	Without intervention/comparator	Without intervention/comparator	-
1791	2018	Effects of strength exercise on the knee and ankle proprioception of individuals with knee osteo	No	Without intervention/comparator	Without intervention/comparator	-
1792	2019	Effect of adding whole-body vibration training to squat training on physical function and muscl	No	Without intervention/comparator	Without intervention/comparator	-
1793	2008	COX-2 Selective Inhibitors in the Treatment of Osteoarthritis	No	Without intervention/comparator	Without intervention/comparator	-
1794	2015	Exercises to improve function of the rheumatoid hand (SARAH): a randomised controlled trial	No	Without intervention/comparator	Without intervention/comparator	-
1795	2015	Evaluation of single-dose and steady-state pharmacokinetics, bioavailability and tolerability of t	No	Without intervention/comparator	Without intervention/comparator	-
1796	2014	COLL2-1NO2: a biomarker for early knee osteoarthritis?	No	Without intervention/comparator	Without intervention/comparator	-
1797	2016	Reducing progression of knee OA features assessed by MRI in overweight and obese women: se	No	Without intervention/comparator	Without intervention/comparator	-
1798	2015	Association of urinary biomarker COLL2-1NO2 with incident clinical and radiographic knee O	No	Without intervention/comparator	Without intervention/comparator	-
1799	2012	Novel and targeted therapies for OA	No	Without intervention/comparator	Without intervention/comparator	-
1800	2007	Degenerative meniscus tears and mobility impairment in women with knee osteoarthritis	No	Without intervention/comparator	Without intervention/comparator	-
1801	2008	Strength training for treatment of osteoarthritis of the knee: a systematic review	No	Review/Systematic Review/Meta-Analy	Review/Systematic Review/Meta-Analys	-

1802	2009	Resistive Exercise for Arthritic Cartilage Health (REACH): a randomized double-blind, sham-ex	No	Without intervention/comparator	Other populations/diseases	Without intervention/c
1803	2019	Effects of Aerobic and Resistance Exercise in Older Adults With Rheumatoid Arthritis: a Rando	No	Without intervention/comparator	Without intervention/comparator	-
1804	2015	Sustained acoustic medicine provides pain relief for osteoarthritis of the knee	No	Without intervention/comparator	Without intervention/comparator	-
1805	2017	Inflammation and post-operative recovery in patients undergoing total knee arthroplasty-seconda	No	Without intervention/comparator	Without intervention/comparator	-
1806	2019	A randomized controlled trial comparing subscapularis tenotomy with peel in anatomic shoulder	No	Without intervention/comparator	Without intervention/comparator	-
1807	2012	Comparison of lesser tuberosity osteotomy to subscapularis peel in shoulder arthroplasty: a rand	No	Without intervention/comparator	Without intervention/comparator	-
1808	2013	Healing rates and subscapularis fatty infiltration after lesser tuberosity osteotomy versus subscap	No	Without intervention/comparator	Without intervention/comparator	-
1809	2017	Community-based intervention to promote physical activity in rheumatoid arthritis (CIPPA-RA)	No	Without intervention/comparator	Without intervention/comparator	-
1810	2017	Osteoporosis and ischemic cardiovascular disease	No	Other populations/diseases	Other populations/diseases	-
1811	2019	Multimodal rehabilitation in patients with persistent pain and functional disability after primary	No	Without intervention/comparator	Without intervention/comparator	-
1812	2020	Neuromuscular exercise and pain neuroscience education compared with pain neuroscience educ	No	Without intervention/comparator	Without intervention/comparator	-
1813	2020	Study protocol for a randomized controlled trial of neuromuscular exercise and pain neuroscienc	No	Without intervention/comparator	Without intervention/comparator	-
1814	2012	A prospective multicenter study of Legg-Calve-Perthes disease: functional and radiographic outc	No	Without intervention/comparator	Without intervention/comparator	-
1815	2012	Treatment with 4jointz reduces knee pain over twelve weeks of treatment in patients with clinica	No	Without intervention/comparator	Without intervention/comparator	-
1816	2014	Capsaicin treatment for osteoarthritis pain: a meta-analysis	No	Review/Systematic Review/Meta-Analys	Review/Systematic Review/Meta-Analys	-
1817	2014	Capsaicin treatment for osteoarthritis pain: a meta-analysis	No	Review/Systematic Review/Meta-Analys	Review/Systematic Review/Meta-Analys	-
1818	2013	Treatment with 4jointz reduces knee pain over twelve weeks of treatment in patients with clinica	No	Without intervention/comparator	Without intervention/comparator	-
1819	2018	A brief report on the clinical trial on neural mobilization exercise for joint pain in patients with r	No	Without intervention/comparator	Without intervention/comparator	-
1820	2014	The effects of exercise and neuromuscular electrical stimulation in subjects with knee osteoarthr	No	Without intervention/comparator	Without intervention/comparator	-
1821	2018	Combined Effect of Sauna Bathing and Cardiorespiratory Fitness on the Risk of Sudden Cardiac	No	Other populations/diseases	Other populations/diseases	-
1822	2019	Impacts of a multicomponent intervention programme on neuropsychiatric symptoms in people v	No	Other populations/diseases	Other populations/diseases	-
1823	2018	"I was really sceptical...But it worked really well": a qualitative study of patient perceptions of te	No	Without intervention/comparator	Without intervention/comparator	-
1824	2018	âI was really scepticalâ¦But it worked really wellâ: a qualitative study of patient perceptions of te	No	Without intervention/comparator	Without intervention/comparator	-
1825	2018	Moderators of Effects of Internet-Delivered Exercise and Pain Coping Skills Training for People	No	Without intervention/comparator	Without intervention/comparator	-
1826	2018	Training Physical Therapists in Person-Centered Practice for People With Osteoarthritis: a Quali	No	Without intervention/comparator	Without intervention/comparator	-
1827	2019	"I Was Really Pleasantly Surprised": firsthand Experience and Shifts in Physical Therapist Perce	No	Without intervention/comparator	Without intervention/comparator	-
1828	2019	Therapeutic alliance between physiotherapists and patients with knee osteoarthritis consulting vi	No	Without intervention/comparator	Without intervention/comparator	-
1829	2016	Effects of Making Art and Listening to Music on Symptoms Related to Blood and Marrow Trans	No	Other populations/diseases	Other populations/diseases	-
1830	2016	Functional Comparison of the Outcome after Midvastus and Medial Parapatellar Surgical Appro	No	Without intervention/comparator	Without intervention/comparator	-
1831	2015	Quality Markers in Cardiology. Main Markers to Measure Quality of Results (Outcomes) and Qu	No	Other populations/diseases	Other populations/diseases	-
1832	2018	Long-term, health-enhancing physical activity is associated with reduction of pain but not pain s	No	Without intervention/comparator	Without intervention/comparator	-
1833	2014	Effect of intermittent aerobic exercise on sleep quality and sleep disturbances in patients with rh	No	Without intervention/comparator	Without intervention/comparator	-
1834	2017	Effect of Tai Ji Quan training on self-reported sleep quality in elderly Chinese women with knee	No	Without intervention/comparator	Without intervention/comparator	-
1835	2015	Compliance of hand orthosis in rheumatoid arthritis : a randomised, comparative, controlled stud	No	Without intervention/comparator	Without intervention/comparator	-
1836	2017	Exercise and adherence over two years: beliefs of adults with knee osteoarthritis	No	Without intervention/comparator	Without intervention/comparator	-
1837	2017	Promoting Exercise Adherence Among Adults with Knee Osteoarthritis: a New Look	No	Without intervention/comparator	Without intervention/comparator	-
1838	2018	Kinesiophobia and physical function among adults with knee osteoarthritis: before and after stre	No	Without intervention/comparator	Without intervention/comparator	-
1839	2016	Mindfulness predicts treatment response from non-pharmacological therapy in knee osteoarthriti	No	Without intervention/comparator	Without intervention/comparator	-
1840	2017	Dose-response effects of tai chi and physical therapy exercise interventions in symptomatic knee	No	Without intervention/comparator	Without intervention/comparator	-
1841	2017	Pain and functional trajectories in symptomatic knee osteoarthritis over a 12- week period of nor	No	Without intervention/comparator	Without intervention/comparator	-
1842	2016	Longitudinal construct validity for four patient-reported outcomes measurement information syst	No	Without intervention/comparator	Without intervention/comparator	-
1843	2017	Mindfulness Is Associated With Treatment Response From Nonpharmacologic Exercise Interven	No	Without intervention/comparator	Without intervention/comparator	-
1844	2018	Dose-Response Effects of Tai Chi and Physical Therapy Exercise Interventions in Symptomatic	No	Without intervention/comparator	Without intervention/comparator	-
1845	2018	Pain and functional trajectories in symptomatic knee osteoarthritis over up to 12 weeks of exerci	No	Without intervention/comparator	Without intervention/comparator	-

1846	2011	Complementary and alternative medicine in chronic pain	No	Without intervention/comparator	Without intervention/comparator	-
1847	2017	Mechanism evaluation of a lifestyle intervention for patients with musculoskeletal pain who are	No	Other populations/diseases	Other populations/diseases	-
1848	2012	Tai Chi exercise and auricular acupressure for people with rheumatoid arthritis: an evaluation stu	No	Without intervention/comparator	Without intervention/comparator	-
1849	2017	Aqua walking as an alternative exercise modality during cardiac rehabilitation for coronary arter	No	Without intervention/comparator	Without intervention/comparator	-
1850	2013	Moxibustion for treating knee osteoarthritis: study protocol of a multicentre randomised controll	No	Without intervention/comparator	Without intervention/comparator	-
1851	2013	Is multimodal analgesia as effective as postoperative patient-controlled analgesia following uppe	No	Without intervention/comparator	Without intervention/comparator	-
1852	2012	Immediate effects of motion style acupuncture treatment (MSAT) in acute low back pain with se	No	Without intervention/comparator	Without intervention/comparator	-
1853	2016	Phase i clinical trial of intra-articular injection of autologous mesenchymal stem cells for the trea	No	Other populations/diseases	Other populations/diseases	-
1854	2016	Effects of an early eccentrically based rehabilitation after total knee arth-roplasty	No	Without intervention/comparator	Without intervention/comparator	-
1855	2011	Promoting physical activity in children with juvenile idiopathic arthritis through an internet-base	No	Other populations/diseases	Other populations/diseases	-
1856	2009	Effects of high-intensity resistance training in patients with rheumatoid arthritis: a randomized c	No	Without intervention/comparator	Without intervention/comparator	-
1857	2012	Are the benefits of a high-intensity progressive resistance training program sustained in rheumat	No	Without intervention/comparator	Without intervention/comparator	-
1858	2020	Kinesiotape and quadriceps strengthening with elastic band in women with knee osteoarthritis ar	No	Without intervention/comparator	Without intervention/comparator	-
1859	2019	Live Music Therapy During Rehabilitation After Total Knee Arthroplasty: a Randomized Contro	No	Without intervention/comparator	Without intervention/comparator	-
1860	2012	Osteoarthritis of the trapeziometacarpal joint: conservative treatment according to guidelines of l	No	Without intervention/comparator	Without intervention/comparator	-
1861	2013	Comparing conventional physical therapy rehabilitation with neuromuscular electrical stimulatic	No	Without intervention/comparator	Without intervention/comparator	-
1862	2017	High-speed resistance training and balance training for people with knee osteoarthritis to reduce	No	Without intervention/comparator	Without intervention/comparator	-
1863	2016	Reduced knee adduction moments for management of knee osteoarthritis:: a three month phase I	No	Without intervention/comparator	Without intervention/comparator	-
1864	2009	The effect of tourniquet use on hidden blood loss in total knee arthroplasty	No	Other populations/diseases	Other populations/diseases	-
1865	2017	Effect of continuous and single shot adductor canal blocks for postoperative analgesia and early	No	Without intervention/comparator	Without intervention/comparator	-
1866	2017	Effects of multi-site infiltration analgesia on pain management and early rehabilitation comparec	No	Without intervention/comparator	Without intervention/comparator	-
1867	2016	Clinical observation of Taoren Xikang Pills combined with Chinese medicine impregnation metl	No	Without intervention/comparator	Without intervention/comparator	-
1868	2017	Efficacy of a wearable-enabled physical activity counselling program for people with knee osteo	No	Without intervention/comparator	Without intervention/comparator	-
1869	2019	Tai chi chuan exercises improve functional outcomes and quality of life in patients with primary	No	Without intervention/comparator	Without intervention/comparator	-
1870	2018	Efficacy of a Community-Based Technology-Enabled Physical Activity Counseling Program for	No	Without intervention/comparator	Without intervention/comparator	-
1871	2015	Case control study of risk factors for frozen shoulder in China	No	Other populations/diseases	Other populations/diseases	-
1872	2019	Blood flow restriction training: A new method for accelerating musculoskeletal rehabilitation	No	Other populations/diseases	Other populations/diseases	-
1873	2018	Mini-subvastus versus medial parapatellar approach for total knee arthroplasty: a prospective rar	No	Without intervention/comparator	Without intervention/comparator	-
1874	2019	Comparison of therapeutic effect of soft-tissue relaxing needling and electroacupuncture for knee	No	Without intervention/comparator	Without intervention/comparator	-
1875	2013	Effects of balance training on functional outcome after total knee replacement in patients with kı	No	Without intervention/comparator	Without intervention/comparator	-
1876	2015	Functional outcomes of outpatient balance training following total knee replacement in patients v	No	Without intervention/comparator	Without intervention/comparator	-
1877	2020	Effects of Elastic Resistance Exercise After Total Knee Replacement on Muscle Mass and Physi	No	Without intervention/comparator	Without intervention/comparator	-
1878	2017	Dose-response relationships following 3 months resistance training in older adults on circulating	No	Other populations/diseases	Other populations/diseases	-
1879	2010	Ergometer cycling after hip or knee replacement surgery: a randomized controlled trial	No	Without intervention/comparator	Without intervention/comparator	-
1880	2012	Multicenter randomized controlled trial comparing early versus late aquatic therapy after total hi	No	Without intervention/comparator	Without intervention/comparator	-
1881	2010	Effectiveness of aquatic exercise for obese patients with knee osteoarthritis: a randomized contro	No	Without intervention/comparator	Without intervention/comparator	-
1882	2016	Effects of joint effusion on quadriceps muscles in patients with knee osteoarthritis	No	Without intervention/comparator	Without intervention/comparator	-
1883	2019	Blood flow restriction and blood flow restriction resistance training improves muscle mass, musc	No	Case report	Case report	-
1884	2018	Incidence and related risk factors of radiographic knee osteoarthritis in a Chinese suburban area:	No	Without intervention/comparator	Without intervention/comparator	-
1885	2011	Multi-center clinical study on therapeutic effect of kunxian capsule on rheumatoid arthritis	No	Without intervention/comparator	Without intervention/comparator	-
1886	2016	The clinical observation of rheumatoid arthritis treated with traditional Chinese and Western me	No	Without intervention/comparator	Without intervention/comparator	-
1887	2009	Quadriceps-sparing, minimal-incision total knee arthroplasty: a comparative study	No	Other populations/diseases	Other populations/diseases	-
1888	2015	Frontal plane kinematics in walking with moderate hip osteoarthritis: stability and fall risk	No	Without intervention/comparator	Without intervention/comparator	-
1889	2020	Active video games for knee osteoarthritis improve mobility but not WOMAC score: a randomiz	No	Without intervention/comparator	Without intervention/comparator	-

1890	2020	Quadriceps tendon grafts does not cause patients to have inferior subjective outcome after anteri	No	Without intervention/comparator	Without intervention/comparator	-
1891	2019	Acute highâintensity football games can improve children's inhibitory control and neurophysiolo	No	Without intervention/comparator	Without intervention/comparator	-
1892	2011	Cemented versus uncemented fixation of humeral components in total shoulder arthroplasty for c	No	Without intervention/comparator	Without intervention/comparator	-
1893	2014	[The contributing risk factors, prevention and treatment of functional dependence among the old	No	Without intervention/comparator	Without intervention/comparator	-
1894	2019	Glucosamine, chondroitin sulfate combined with bone health exercise can promote bone mineral	No	Other populations/diseases	Other populations/diseases	-
1895	2019	Different exercise modalities relieve pain syndrome in patients with knee osteoarthritis and modu	No	Without intervention/comparator	Without intervention/comparator	-
1896	2019	Modulatory effects of different exercise modalities on the functional connectivity of the periaque	No	Without intervention/comparator	Without intervention/comparator	-
1897	2014	A multicenter, randomized, double-blinded, placebo-controlled trial evaluating the efficacy and c	No	Without intervention/comparator	Without intervention/comparator	-
1898	2016	A multicenter, randomized, double-blinded, placebo-controlled trial evaluating the efficacy and c	No	Without intervention/comparator	Without intervention/comparator	-
1899	2009	Effects of wenhua juanbi recipe on TNF-alpha and IL-1beta in peripheral blood of rheumatoid ar	No	Without intervention/comparator	Without intervention/comparator	-
1900	2012	[Closed treatment for type III humeral supracondylar fractures and prevention of ischemic contra	No	Other populations/diseases	Other populations/diseases	-
1901	2000	Clinical observation on the effect of Chinese medicine, physical therapy and exercises for rheum	No	Without intervention/comparator	Without intervention/comparator	-
1902	2017	The effect of nerve mobilization exercise in patients with rheumatoid arthritis: a pilot study	No	Without intervention/comparator	Without intervention/comparator	-
1903	2020	Walking for exercise may be symptom and structure modifying for those with established osteoa	No	Without intervention/comparator	Without intervention/comparator	-
1904	2016	Effects of exercise and weight loss on interstitial matrix turnover and tissue inflammation bioma	No	Without intervention/comparator	Without intervention/comparator	-
1905	2017	Effects of dietary weight loss with and without exercise on interstitial matrix turnover and tissue	No	Without intervention/comparator	Without intervention/comparator	-
1906	2014	The implementation of Ottawa panel evidence-based clinical practice guidelines for aerobic walk	No	Without intervention/comparator	Without intervention/comparator	-
1907	2017	An evidence-based walking program among older people with knee osteoarthritis: the PEP (parti	No	Without intervention/comparator	Without intervention/comparator	-
1908	2007	Clinical update: treating osteoarthritis	No	Without intervention/comparator	Without intervention/comparator	-
1909	2015	Cognitive behavioral group intervention for pain and well-being in children with juvenile idiopat	No	Other populations/diseases	Other populations/diseases	-
1910	2017	Effects of dietary supplementation with a standardized aqueous extract of Terminalia chebula fru	No	Without intervention/comparator	Without intervention/comparator	-
1911	2014	Effect of intermittent aerobic exercise on sleep quality and sleep disturbances in patients with rh	No	Without intervention/comparator	Without intervention/comparator	-
1912	2019	Efficacy of intermittent aerobic exercise on sleep efficiency in patients with rheumatoid arthritis.	No	Without intervention/comparator	Without intervention/comparator	-
1913	2019	Parentâdirected intervention in promoting knowledge of pediatric nutrition and healthy lifestyle a	No	Other populations/diseases	Other populations/diseases	-
1914	2017	Randomized Controlled Trial of an Educational Intervention Using an Online Risk Calculator fo	No	Without intervention/comparator	Without intervention/comparator	-
1915	2018	Financial Incentives and Health Coaching to Improve Physical Activity Following Total Knee Re	No	Without intervention/comparator	Without intervention/comparator	-
1916	2017	Effectiveness of an overall progressive resistance strength program for improving the functional	No	Without intervention/comparator	Without intervention/comparator	-
1917	2017	Influence of Hip Abductor Strength on Functional Outcomes Before and After Total Knee Arthro	No	Without intervention/comparator	Without intervention/comparator	-
1918	2019	Comparison of the functional impact of verum and placebo thumb base orthoses: a proof of conc	No	Without intervention/comparator	Without intervention/comparator	-
1919	2014	Differences between women and men with intermittent claudication: a cross-sectional study	No	Without intervention/comparator	Without intervention/comparator	-
1920	2020	Effect of ultrasound combined with nerve stimulator in guiding saphenous nerve block on patien	No	Without intervention/comparator	Without intervention/comparator	-
1921	2014	Effects of exercises on knee cartilage volume in young healthy adults: a randomized controlled tr	No	Other populations/diseases	Other populations/diseases	-
1922	2018	Associations among knee muscle strength, structural damage, and pain and mobility in individua	No	Without intervention/comparator	Without intervention/comparator	-
1923	2018	Associations among quadriceps strength, joint structure, and pain and ambulation in individuals	No	Without intervention/comparator	Without intervention/comparator	-
1924	2013	Strontium ranelate in bone healing: the results of a randomized clinical trial	No	Other populations/diseases	Other populations/diseases	-
1925	2011	Does emotional disclosure about stress improve health in rheumatoid arthritis? Randomized, con	No	Without intervention/comparator	Without intervention/comparator	-
1926	2015	Efficacy of hip strengthening exercises compared with leg strengthening exercises on knee pain,	No	Without intervention/comparator	Without intervention/comparator	-
1927	2009	Can stimulating massage improve joint repositioning error in patients with knee osteoarthritis?	No	Without intervention/comparator	Without intervention/comparator	-
1928	2017	Feasibility of two exercise programs in patients with knee osteoarthritis	No	Without intervention/comparator	Without intervention/comparator	-
1929	2001	Exercise prescription for older adults with osteoarthritis pain: consensus practice recommendatio	No	Without intervention/comparator	Without intervention/comparator	-
1930	2020	Effectiveness and cost-effectiveness of guided Internet- And mobile-based CBT for adolescents a	No	Other populations/diseases	Other populations/diseases	-
1931	2019	Randomized controlled clinical trial of acupuncture treatment for knee osteoarthritis in the early	No	Without intervention/comparator	Without intervention/comparator	-
1932	2017	Additive effect of tDCS combined with Peripheral Electrical Stimulation to an exercise program	No	Without intervention/comparator	Without intervention/comparator	-
1933	2018	The effects of exercise rehabilitation on physiological function and quality of life in maintain he	No	Other populations/diseases	Other populations/diseases	-

1934	2008	Aquatic exercise and quality of life in persons with osteoarthritis	No	Without intervention/comparator	Without intervention/comparator	-
1935	2019	The effect of extension assist orthosis with pneumatic bladders on pain and function for patients	No	Without intervention/comparator	Without intervention/comparator	-
1936	2013	Late group-based rehabilitation has no advantages compared with supervised home-exercises aft	No	Without intervention/comparator	Without intervention/comparator	-
1937	2013	Bovine xenograft locking Puddu plate versus tricalcium phosphate spacer non-locking Puddu pla	No	Without intervention/comparator	Without intervention/comparator	-
1938	2016	Effects of cyclic yoga on bone health in postmenopausal women	No	Other populations/diseases	Other populations/diseases	-
1939	2014	The Effect of Femoral Nerve Block on Strength and Patient-reported Outcomes Following ACL	No	Other populations/diseases	Other populations/diseases	-
1940	2017	Femoral Nerve Block after Anterior Cruciate Ligament Reconstruction	No	Other populations/diseases	Other populations/diseases	-
1941	2013	Effects of aquatic therapy versus conventional land-based exercise on pain relief in rheumatoid a	No	Without intervention/comparator	Without intervention/comparator	-
1942	2019	Effectiveness of low-dose radiation therapy on symptoms in patients with knee osteoarthritis: a r	No	Without intervention/comparator	Without intervention/comparator	-
1943	2020	Effects of mulligan's mobilization with movements versus myofascial release in addition to usua	No	Without intervention/comparator	Without intervention/comparator	-
1944	2016	Effect of Electromyographic Biofeedback Training on Pain, Quadriceps Muscle Strength, and Fu	No	Other populations/diseases	Other populations/diseases	-
1945	2019	Effectiveness of home based intervention program in reducing mortality of hip fracture patients:	No	Other populations/diseases	Other populations/diseases	-
1946	2016	Dexamethasone and viscossuplementation in the treatment of patients with knee osteoarthritis: c	No	Without intervention/comparator	Without intervention/comparator	-
1947	2019	Viscosupplementation improves pain, function and muscle strength, but not proprioception, in p	No	Without intervention/comparator	Without intervention/comparator	-
1948	2017	PNF and manual therapy treatment results of patients with cervical spine osteoarthritis	No	Without intervention/comparator	Without intervention/comparator	-
1949	2020	Effects of a multicomponent exercise program in physical function and muscle mass in sarcopen	No	Other populations/diseases	Other populations/diseases	-
1950	2013	Effects of different strength training on muscle architecture: clinical and ultrasonographic evalua	No	Without intervention/comparator	Without intervention/comparator	-
1951	2016	Superior gains in bone mineral density and estimated strength at the hip for romosozumab comp	No	Other populations/diseases	Other populations/diseases	-
1952	2014	Osteoarthritis year in review: rehabilitation and outcomes	No	Review/Systematic Review/Meta-Analys	Review/Systematic Review/Meta-Analys	-
1953	2019	Protocol for a randomised trial evaluating the comparative effectiveness of strategies to promote	No	Without intervention/comparator	Without intervention/comparator	-
1954	2020	Effectiveness of exergaming, Tai Chi, and physical therapy in improving dynamic balance and e	No	Without intervention/comparator	Without intervention/comparator	-
1955	2013	Upper limb exercise, education and self management in early rheumatoid arthritis (the extra prog	No	Without intervention/comparator	Without intervention/comparator	-
1956	2013	A brief exercise and self-management programme improves upper limb disability in people with	No	Without intervention/comparator	Without intervention/comparator	-
1957	2014	Education, self-management, and upper extremity exercise training in people with rheumatoid ar	No	Without intervention/comparator	Without intervention/comparator	-
1958	2015	Economic evaluation of a brief Education, Self-management and Upper Limb Exercise Training	No	Without intervention/comparator	Without intervention/comparator	-
1959	2016	The PLE(2)NO self-management and exercise program for knee osteoarthritis: study Protocol for	No	Without intervention/comparator	Without intervention/comparator	-
1960	2018	A randomized controlled trial of a combined self-management and exercise intervention for elder	No	Without intervention/comparator	Without intervention/comparator	-
1961	2014	Effect of hydrotherapy vs. Conventional land-based exercise in patients with hiparthroplasty for	No	Without intervention/comparator	Without intervention/comparator	-
1962	2015	Correlations between the functional status and the quality of life in patients with hip osteoarthrit	No	Without intervention/comparator	Without intervention/comparator	-
1963	2015	Efficacy of physical exercise in patients with fibromyalgia	No	Other populations/diseases	Other populations/diseases	-
1964	2016	Correlations between functional status and quality of life in patients with hip osteoarthritis	No	Without intervention/comparator	Without intervention/comparator	-
1965	2016	Role of physical exercise in patients with knee osteoarthritis	No	Without intervention/comparator	Without intervention/comparator	-
1966	2017	Physical exercise program in patients with scapulohumeral periarthritis	No	Other populations/diseases	Other populations/diseases	-
1967	2017	Role of physical exercise in patients with knee arthroplasty for osteoarthritis	No	Without intervention/comparator	Without intervention/comparator	-
1968	2017	The impact ofrehabilitation program on increasing quality of life in patients with low back pain	No	Other populations/diseases	Other populations/diseases	-
1969	2018	Results of rehabilitation treatment on functional status and quality of life in patients with hip ost	No	Without intervention/comparator	Without intervention/comparator	-
1970	2013	Role of physical exercise in patients with hip osteoarthritis	No	Without intervention/comparator	Without intervention/comparator	-
1971	2011	Low-level laser therapy and exercise in patients with knee osteoarthritis	No	Without intervention/comparator	Without intervention/comparator	-
1972	2010	Pharmacist-initiated Intervention Trial in Osteoarthritis (PhIT-OA)	No	Without intervention/comparator	Without intervention/comparator	-
1973	2012	Pharmacist-initiated intervention trial in osteoarthritis: a multidisciplinary intervention for knee	No	Without intervention/comparator	Without intervention/comparator	-
1974	2014	Cost-utility analysis of a multidisciplinary strategy to manage osteoarthritis of the knee: econom	No	Without intervention/comparator	Without intervention/comparator	-
1975	2019	A year in review: rehabilitation & outcomes	No	Review/Systematic Review/Meta-Analys	Review/Systematic Review/Meta-Analys	-
1976	2016	Outcome expectations for exercise are associated with self-efficacy and depression in patients w	No	Without intervention/comparator	Without intervention/comparator	-
1977	2019	Effects of 24âweek exergame intervention on physical function under singleâ and dualâtask conc	No	Other populations/diseases	Other populations/diseases	-

1978	2018	Effects of the insulin-like growth factor axis and its relationship in nonsurgical treatments in pat	No	Other populations/diseases	Other populations/diseases	-
1979	2017	Trends in activity levels a year after hip resurfacing arthroplasty	No	Without intervention/comparator	Without intervention/comparator	-
1980	2019	Is there scope to implement the evidencebased walk with ease programme more widely in the Ur	No	Without intervention/comparator	Without intervention/comparator	-
1981	2019	Protocol for a multicentre randomised controlled parallel-group trial to compare the effectiveness	No	Without intervention/comparator	Without intervention/comparator	-
1982	2017	Effectiveness of an overall progressive resistance strength program for improving the functional	No	Without intervention/comparator	Without intervention/comparator	-
1983	2014	Factors influencing further acupuncture usage and a more positive outcome in patients with oste	No	Without intervention/comparator	Without intervention/comparator	-
1984	2012	Effects of kinesiotherapy, ultrasound and electrotherapy in management of bilateral knee osteoar	No	Without intervention/comparator	Without intervention/comparator	-
1985	2018	Capsulodesis Versus Bone Trough Technique in Lateral Meniscal Allograft Transplantation: gra	No	Without intervention/comparator	Without intervention/comparator	-
1986	2020	Does aquatic thermal therapy improve quality of life after total hip replacement? A retrospective	No	Without intervention/comparator	Without intervention/comparator	-
1987	2010	Methodological issues arising from a pilot RCT investigating the effectiveness of joint protectio	No	Without intervention/comparator	Without intervention/comparator	-
1988	2016	Can exercise interventions designed to reduce falls improve bone quality	No	Other populations/diseases	Other populations/diseases	-
1989	2016	Preoperative physiotherapy and short-term functional outcomes of primary total knee arthroplast	No	Without intervention/comparator	Without intervention/comparator	-
1990	2016	Effect of the Otago exercises on postural balance and fear of falling among older fallers with kne	No	Without intervention/comparator	Without intervention/comparator	-
1991	2018	Effect of Modified Otago Exercises on Postural Balance, Fear of Falling, and Fall Risk in Older	No	Without intervention/comparator	Without intervention/comparator	-
1992	2014	Range of motion after total knee arthroplasty: the effect of a preoperative home exercise progran	No	Without intervention/comparator	Without intervention/comparator	-
1993	2014	Effectiveness of rehabilitation program on lower limb functional status after knee arthroplasty	No	Without intervention/comparator	Without intervention/comparator	-
1994	2018	Strength and mobilization training within the first week following total hip arthroplasty	No	Without intervention/comparator	Without intervention/comparator	-
1995	2017	Combined application of electrical stimulation and volitional contraction prevents muscle weakr	No	Without intervention/comparator	Without intervention/comparator	-
1996	2011	The role of exercise in combination with NSAID or glucosamine on rage immunoreactivity in th	No	Without intervention/comparator	Without intervention/comparator	-
1997	2013	Morphological adaptation of muscle collagen and receptor of advanced glycation end product (R	No	Without intervention/comparator	Without intervention/comparator	-
1998	2018	The Application of Blood Flow Restriction: Lessons From the Laboratory	No	Without intervention/comparator	Without intervention/comparator	-
1999	2014	Cardiac tissue characterization and the diagnostic value of cardiovascular magnetic resonance in	No	Without intervention/comparator	Without intervention/comparator	-
2000	2017	Myocardial perfusion in peripheral Raynaud's phenomenon. Evaluation using stress cardiovascu	No	Other populations/diseases	Other populations/diseases	-
2001	2013	Implementation of the osteoarthritis clinical guideline: results of a cluster randomized trial in pri	No	Without intervention/comparator	Without intervention/comparator	-
2002	2017	Neuromuscular electrostimulation (nmes) used for increasing quadriceps strength may also provi	No	Other populations/diseases	Other populations/diseases	-
2003	2014	Comparison of the effect of aquatic exercise therapy and land-based therapeutic exercise on kne	No	Without intervention/comparator	Without intervention/comparator	-
2004	2018	The comparison of the effectiveness of conventional therapeutic exercises and Pilates on pain an	No	Without intervention/comparator	Without intervention/comparator	-
2005	2019	Gait retraining as a conservative treatment for medial knee osteoarthritis	No	Without intervention/comparator	Without intervention/comparator	-
2006	2018	Foot exercises and foot orthoses are more effective than knee focused exercises in individuals wi	No	Without intervention/comparator	Without intervention/comparator	-
2007	2015	Impact of a magnetic resonance imaging-guided treat-to-target strategy on disease activity and p	No	Without intervention/comparator	Without intervention/comparator	-
2008	2019	A Pilot Study of the Effects of Chair Yoga and Chair-Based Exercise on Biopsychosocial Outco	No	Without intervention/comparator	Without intervention/comparator	-
2009	2001	The effectiveness of supplementing a home exercise programme with a class based exercise prog	No	Without intervention/comparator	Without intervention/comparator	-
2010	2016	Impact of falls on fractures and mortality-an opportunity for intervention and enhancement of fra	No	Other populations/diseases	Other populations/diseases	-
2011	2019	The Impact of Morning versus Afternoon Exercise on Iron Absorption in Athletes	No	Other populations/diseases	Other populations/diseases	-
2012	2003	Rehabilitation effects on compensatory gait mechanics in people with arthritis and strength impa	No	Without intervention/comparator	Without intervention/comparator	-
2013	2016	Aquatic exercise for women with persistent knee pain. a pilot randomised controlled trial	No	Without intervention/comparator	Without intervention/comparator	-
2014	2017	Aquatic therapy for people with persistent knee pain: a feasibility study	No	Without intervention/comparator	Without intervention/comparator	-
2015	2012	The effect of a prehabilitation exercise program on quadriceps strength for patients undergoing t	No	Without intervention/comparator	Without intervention/comparator	-
2016	2019	Rehabilitation following regenerative medicine treatment for knee osteoarthritis-current concept	No	Without intervention/comparator	Without intervention/comparator	-
2017	2018	The impact of exercise on sleep (time, quality, and disturbance) in patients with rheumatoid arth	No	Without intervention/comparator	Without intervention/comparator	-
2018	2018	The impact of exercise on sleep in people with rheumatoid arthritis: a pilot randomised controlle	No	Without intervention/comparator	Without intervention/comparator	-
2019	2019	The association between comorbidity and physical activity levels in people with osteoarthritis: s	No	Without intervention/comparator	Without intervention/comparator	-
2020	2010	A comparison of strength training, self-management, and the combination for early osteoarthritis	No	Without intervention/comparator	Without intervention/comparator	-
2021	2010	Coping self-efficacy as a mediator between catastrophizing and physical functioning: treatment t	No	Without intervention/comparator	Without intervention/comparator	-

2022	2017	Baseline Characteristics of Participants in the ASPREE (ASPirin in Reducing Events in the Elde	No	Other populations/diseases	Other populations/diseases	-
2023	2015	Osteoarthritis of the knee	No	Without intervention/comparator	Without intervention/comparator	-
2024	2018	Effects of a 12-Week Digital Care Program for Chronic Knee Pain on Pain, Mobility, and Surge	No	Without intervention/comparator	Without intervention/comparator	-
2025	2018	Class IV laser therapy for trapeziometacarpal joint osteoarthritis: study protocol for a randomize	No	Without intervention/comparator	Without intervention/comparator	-
2026	2011	Serotonergic descending inhibition in chronic pain: design, preliminary results and early cessatic	No	Without intervention/comparator	Without intervention/comparator	-
2027	2015	Endogenous pain modulation in response to exercise in patients with rheumatoid arthritis, patien	No	Without intervention/comparator	Without intervention/comparator	-
2028	2010	Assessment of the effectiveness of low-level laser therapy on the hands of patients with rheumat	No	Without intervention/comparator	Without intervention/comparator	-
2029	2007	Evidence for placebo effects on physical but not on biochemical outcome parameters: a review o	No	Review/Systematic Review/Meta-Analy	Review/Systematic Review/Meta-Analys	-
2030	2017	Increased risk for incident hip fracture in men with type 2 diabetes	No	Other populations/diseases	Other populations/diseases	-
2031	2015	Effects of neuromuscular electrical stimulation and low-level laser therapy on the muscle archite	No	Without intervention/comparator	Without intervention/comparator	-
2032	2017	Stepped care approach for medial tibiofemoral osteoarthritis	No	Without intervention/comparator	Without intervention/comparator	-
2033	2018	Stepped care approach for medial tibiofemoral osteoarthritis (streamline)	No	Without intervention/comparator	Without intervention/comparator	-
2034	2019	Comparison between intra-articular Botulinum toxin type A, corticosteroid, and saline in knee o	No	Without intervention/comparator	Without intervention/comparator	-
2035	2015	Effect of low-level laser therapy (904nm) and static stretching exercises in patients with knee os	No	Without intervention/comparator	Without intervention/comparator	-
2036	2017	Predictors of response to prefabricated foot orthoses or rocker-sole footwear in individuals with	No	Without intervention/comparator	Without intervention/comparator	-
2037	2009	Use of recommended osteoarthritis pain treatment by older adults	No	Without intervention/comparator	Without intervention/comparator	-
2038	2009	The Intensive Diet and Exercise for Arthritis (IDEA) trial: design and rationale	No	Without intervention/comparator	Without intervention/comparator	-
2039	2011	Does high weight loss in older adults with knee osteoarthritis affect bone-on-bone joint loads an	No	Without intervention/comparator	Without intervention/comparator	-
2040	2011	The intensive diet and exercise for arthritis trial: 18-month clinical outcomes	No	Without intervention/comparator	Without intervention/comparator	-
2041	2012	The intensive diet and exercise for arthritis trial (IDEA): effects on knee joint loading and inflam	No	Without intervention/comparator	Without intervention/comparator	-
2042	2013	Effects of intensive diet and exercise on knee joint loads, inflammation, and clinical outcomes a	No	Without intervention/comparator	Without intervention/comparator	-
2043	2013	Strength Training for Arthritis Trial (START): design and rationale	No	Without intervention/comparator	Without intervention/comparator	-
2044	2014	Changes in joint loads, leptin, and mmp-3 subsequent to long-term intensive weight loss and exe	No	Without intervention/comparator	Without intervention/comparator	-
2045	2014	Knee joint loading in knee osteoarthritis: influence of abdominal and thigh fat	No	Without intervention/comparator	Without intervention/comparator	-
2046	2016	Does long-term intensive diet and exercise reduce the biomechanical burden in overweight and c	No	Without intervention/comparator	Without intervention/comparator	-
2047	2017	Weight-loss and exercise for communities with arthritis in North Carolina (we-can): design and	No	Without intervention/comparator	Without intervention/comparator	-
2048	2018	Intentional Weight Loss in Overweight and Obese Patients With Knee Osteoarthritis: is More Be	No	Without intervention/comparator	Without intervention/comparator	-
2049	2019	The effects of intensive dietary weight loss and exercise on gait in overweight and obese adults v	No	Without intervention/comparator	Without intervention/comparator	-
2050	2020	Is long-term strength training more effective for knee osteoarthritis patients with low- versus hig	No	Without intervention/comparator	Without intervention/comparator	-
2051	2018	Effectiveness of Superficial Radial Nerve Block on Pain, Function and Quality of Life in Patient	No	Without intervention/comparator	Without intervention/comparator	-
2052	2010	The success of a general school-based physical activity intervention on bone mineral content dep	No	Other populations/diseases	Other populations/diseases	-
2053	2013	A pilot study of yoga as self-care for arthritis in minority communities	No	Without intervention/comparator	Without intervention/comparator	-
2054	2019	Effect of intensive diet and exercise on self-efficacy in overweight and obese adults with knee os	No	Without intervention/comparator	Without intervention/comparator	-
2055	2010	Effect of intensified home-based exercise after Total Hip Replacement - A clinical randomised c	No	Without intervention/comparator	Without intervention/comparator	-
2056	2011	Feasability of early progressive resistance training after total hip replacement	No	Without intervention/comparator	Without intervention/comparator	-
2057	2012	Early, intensified home-based exercise after total hip replacement--a pilot study	No	Without intervention/comparator	Without intervention/comparator	-
2058	2014	Effect of early supervised progressive resistance training compared to unsupervised home-based	No	Without intervention/comparator	Without intervention/comparator	-
2059	2017	Description of load progression and pain response during progressive resistance training early af	No	Without intervention/comparator	Without intervention/comparator	-
2060	2018	A randomized trial of class 2 and class 3 elastic compression in the prevention of recurrence of v	No	Without intervention/comparator	Without intervention/comparator	-
2061	2019	A Randomized Trial of Moderate (Class 2), High (Class 3), and Very High (Class 4) Elastic Con	No	Without intervention/comparator	Without intervention/comparator	-
2062	2015	A randomized trial of elastic compression systems with high and very high sub-bandage pressure	No	Without intervention/comparator	Without intervention/comparator	-
2063	2012	Basal growth hormone concentration increased following a weight loss focused dietary intervent	No	Without intervention/comparator	Without intervention/comparator	-
2064	2012	Influence of weight loss, body composition, and lifestyle behaviors on plasma adipokines: a rand	No	Without intervention/comparator	Without intervention/comparator	-
2065	2001	Group comparisons involving missing data in clinical trials: a comparison of estimates and powe	No	Other topics	Other populations/diseases	Other topics

2066	2007	Effectiveness of physiotherapy exercise after knee arthroplasty for osteoarthritis: systematic revi	No	Review/Systematic Review/Meta-Analys	Review/Systematic Review/Meta-Analys	-
2067	2012	Comparison of postdischarge physiotherapy versus usual care following primary total knee arthr	No	Without intervention/comparator	Without intervention/comparator	-
2068	2016	Effectiveness of radiation therapy in osteoarthritis of the hand and knee: design of two parallel, t	No	Without intervention/comparator	Without intervention/comparator	-
2069	2019	The associated factors for falls and fear of falls in indian patients with rheumatoid arthritis	No	Without intervention/comparator	Without intervention/comparator	-
2070	2019	Bisphosphonate use in the horse: what is good and what is not?	No	Other populations/diseases	Other populations/diseases	-
2071	2017	The effectiveness of supplementary arm and upper body exercises following total hip arthroplast	No	Without intervention/comparator	Without intervention/comparator	-
2072	2013	Effects of neuromuscular electrical stimulation combined with exercises versus an exercise progr	No	Without intervention/comparator	Without intervention/comparator	-
2073	2019	Do Postoperative Results Differ in a Randomized Trial Between a Direct Anterior and a Direct L	No	Other populations/diseases	Other populations/diseases	-
2074	2011	Evaluating the quality of an on-going clinical trial on the effectiveness of telerehabilitation servi	No	Without intervention/comparator	Without intervention/comparator	-
2075	2015	In-Home Telerehabilitation Compared with Face-to-Face Rehabilitation After Total Knee Arthro	No	Without intervention/comparator	Without intervention/comparator	-
2076	2019	A self-help diet and physical activity intervention with dietetic support for weight management i	No	Other populations/diseases	Other populations/diseases	-
2077	2018	Comparing the Effect of Proprioceptive Retraining Technique against Home Exercise Programm	No	Without intervention/comparator	Without intervention/comparator	-
2078	2000	Manual physical therapy and exercise improved function in osteoarthritis of the knee	No	Without intervention/comparator	Without intervention/comparator	-
2079	2017	Foot exercises and foot orthoses are more effective than knee focused exercises in individuals wi	No	Without intervention/comparator	Without intervention/comparator	-
2080	2015	Impact of a magnetic resonance imaging-guided treat-to-target strategy on disease activity and p	No	Without intervention/comparator	Without intervention/comparator	-
2081	2014	Yesilar early migration when comparing CR and PS in Triathlon TKA: a prospective randomised	No	Without intervention/comparator	Without intervention/comparator	-
2082	2014	The effect of tourniquet use on fixation quality in cemented total knee arthroplasty a prospective	No	Without intervention/comparator	Without intervention/comparator	-
2083	2012	Functional exercise after total hip replacement (FEATHER): a randomised control trial	No	Without intervention/comparator	Without intervention/comparator	-
2084	2015	Functional exercise after total hip replacement (feather)	No	Without intervention/comparator	Without intervention/comparator	-
2085	2017	Randomised controlled trial to evaluate a physiotherapy-led functional exercise programme after	No	Without intervention/comparator	Without intervention/comparator	-
2086	2015	Unloader Bracing for Knee Osteoarthritis: a Pilot Study of Gait and Function	No	Without intervention/comparator	Without intervention/comparator	-
2087	2014	Task-oriented exercises and early full weight-bearing contribute to improving disability after tota	No	Without intervention/comparator	Without intervention/comparator	-
2088	2018	How balance task-specific training contributes to improving physical function in older subjects u	No	Without intervention/comparator	Without intervention/comparator	-
2089	2015	Yoga in Sedentary Adults with Arthritis: effects of a Randomized Controlled Pragmatic Trial	No	Without intervention/comparator	Without intervention/comparator	-
2090	2019	Therapeutic alliance facilitates adherence to physiotherapy-led exercise and physical activity for	No	Without intervention/comparator	Without intervention/comparator	-
2091	2015	A feasibility trial for the efficacy of physiotherapy intervention for early-onset hip osteoarthritis	No	Without intervention/comparator	Without intervention/comparator	-
2092	2019	Efficacy of Blood Flow Restriction Training as Part of Knee Rehabilitation	No	Other topics	Other topics	-
2093	2019	A comparison of the efficacy of nonweight-bearing and weight-bearing exercise programmes on	No	Without intervention/comparator	Without intervention/comparator	-
2094	2014	Intraoperative platelet-rich plasma does not improve outcomes of total knee arthroplasty	No	Without intervention/comparator	Without intervention/comparator	-
2095	2013	Effect of alfacalcidol on muscle strength and performance	No	Without intervention/comparator	Without intervention/comparator	-
2096	2015	Exercise-related transient abdominal pain (ETAP)	No	Other populations/diseases	Other populations/diseases	-
2097	2009	Arthroscopic surgery did not provide additional benefit to physical and medical therapy for osteo	No	Without intervention/comparator	Without intervention/comparator	-
2098	2018	Implementing international osteoarthritis guidelines in primary health care-evaluating fidelity an	No	Without intervention/comparator	Without intervention/comparator	-
2099	2019	Implementing international osteoarthritis guidelines in primary care: uptake and fidelity among h	No	Without intervention/comparator	Without intervention/comparator	-
2100	2016	Subjects with knee osteoarthritis exhibit widespread hyperalgesia to pressure and cold	No	Without intervention/comparator	Without intervention/comparator	-
2101	2017	Fourteen days of etoricoxib 60 mg improves pain, hyperalgesia and physical function in individu	No	Without intervention/comparator	Without intervention/comparator	-
2102	2019	The therapeutic role of motor imagery during the chronic phase after total knee arthroplasty: a pi	No	Without intervention/comparator	Without intervention/comparator	-
2103	2019	Early initiation of home-based sensori-motor training improves muscle strength, activation and s	No	Without intervention/comparator	Without intervention/comparator	-
2104	2018	The Effects of Lower Extremity Strengthening Delivered in the Workplace on Physical Function	No	Without intervention/comparator	Without intervention/comparator	-
2105	2011	The effects of bone exercise on knee pain, stiffness and function among women with mild knee o	No	Without intervention/comparator	Without intervention/comparator	-
2106	2012	High-impact bone exercise does not have controversial effects on articular cartilage: a randomize	No	Without intervention/comparator	Without intervention/comparator	-
2107	2014	Effects of high-impact training on bone and articular cartilage: 12-month randomized controlled	No	Without intervention/comparator	Without intervention/comparator	-
2108	2017	Effect of progressive high-impact exercise on femoral neck structural strength in postmenopausa	No	Without intervention/comparator	Without intervention/comparator	-
2109	2001	The value of a continuous ambulatory activity monitor to quantify the amount and intensity of da	No	Without intervention/comparator	Without intervention/comparator	-

2110	2011	Effectiveness of intra-articular hyaluronan (Synvisc, hylan G-F 20) for the treatment of first meta	No	Without intervention/comparator	Without intervention/comparator	-
2111	2019	The effect of weight loss on the progression of meniscal extrusion and size in knee osteoarthritis	No	Without intervention/comparator	Without intervention/comparator	-
2112	2020	The effect of weight loss on the progression of meniscal extrusion and size in knee osteoarthritis	No	Without intervention/comparator	Without intervention/comparator	-
2113	2016	Association between leisure time physical activity level and articular cartilage in postmenopausa	No	Without intervention/comparator	Without intervention/comparator	-
2114	2016	Efficacy of progressive aquatic resistance training for tibiofemoral cartilage in postmenopausal v	No	Without intervention/comparator	Without intervention/comparator	-
2115	2020	Effects of progressive aquatic resistance training on symptoms and quality of life in women with	No	Without intervention/comparator	Without intervention/comparator	-
2116	2017	Physical Activity Is Related with Cartilage Quality in Women with Knee Osteoarthritis	No	Without intervention/comparator	Without intervention/comparator	-
2117	2016	The effect of exercises on serum Bmp-6 levels of knee osteoarthritis	No	Without intervention/comparator	Without intervention/comparator	-
2118	2014	Does flexion of the femoral implant in total knee arthroplasty increase knee flexion: a randomise	No	Without intervention/comparator	Without intervention/comparator	-
2119	2011	Activity pacing for osteoarthritis symptom management: study design and methodology of a ran	No	Without intervention/comparator	Without intervention/comparator	-
2120	2016	Brief time-based activity pacing instruction as a singular behavioral intervention was not effectiv	No	Without intervention/comparator	Without intervention/comparator	-
2121	2015	Effects of multi-joint kinetics-chain exercise versus conventional exercise for patients with TKA	No	Without intervention/comparator	Without intervention/comparator	-
2122	2010	Gait variability patterns are altered in healthy young individuals during the acute reperfusion pha	No	Other populations/diseases	Other populations/diseases	-
2123	2011	Topical analgesic added to paraffin enhances paraffin bath treatment of individuals with hand os	No	Without intervention/comparator	Without intervention/comparator	-
2124	2000	Effects of Therapeutic Exercise on Patients with Osteoarthritis of Knee	No	Without intervention/comparator	Without intervention/comparator	-
2125	2017	The Effectiveness of Hand Exercise and Thermal Modalities Agents in Managing Osteoarthritis	No	Without intervention/comparator	Without intervention/comparator	-
2126	2019	Maslinic acid derived from olive fruit in combination with resistance training improves muscle r	No	Without intervention/comparator	Without intervention/comparator	-
2127	2010	Evaluation of the effects of a supplementary diet containing chicken comb extract on symptoms	No	Without intervention/comparator	Without intervention/comparator	-
2128	2019	Effectiveness of a low-value financial-incentive program for increasing vegetable-rich restaurant	No	Other populations/diseases	Other populations/diseases	-
2129	2018	The effects of routine physiotherapy alone and in combination with either Tai Chi or closed kine	No	Without intervention/comparator	Without intervention/comparator	-
2130	2009	A comparative study of the effects of bucillamine and salazosulfapyridine in the treatment of rhe	No	Without intervention/comparator	Without intervention/comparator	-
2131	2015	Specific exercises and heel lift improve functional and patient-perceived leg length discrepancie	No	Without intervention/comparator	Without intervention/comparator	-
2132	2016	Effect of virgin olive oil versus piroxicam phonophoresis on exercise-induced anterior knee pain	No	Without intervention/comparator	Without intervention/comparator	-
2133	2009	Comparison of 1.0-T extremity MR and 1.5-T conventional high-field-strength MR in patients w	No	Without intervention/comparator	Without intervention/comparator	-
2134	2017	The connection between diastasis recti abdominis evolution and low-back pain from childbirth t	No	Other populations/diseases	Other populations/diseases	-
2135	2020	Human adipose-derived Mesenchymal stem cells, low-intensity pulsed ultrasound, or their comb	No	Without intervention/comparator	Without intervention/comparator	-
2136	2020	Comparative study of ropivacaine vs bupivacaine for pain relief in postoperative total knee arthr	No	Without intervention/comparator	Without intervention/comparator	-
2137	2015	Is there a role for rehabilitation streaming following total knee arthroplasty? Preliminary insights	No	Without intervention/comparator	Without intervention/comparator	-
2138	2016	Minimal important improvement thresholds for the six-minute walk test in a knee arthroplasty co	No	Without intervention/comparator	Without intervention/comparator	-
2139	2019	Efficacy of high-intensity laser therapy in comparison with conventional physiotherapy and exer	No	Without intervention/comparator	Without intervention/comparator	-
2140	1999	Effects of Comprehensive Care for Knee OA	No	Without intervention/comparator	Without intervention/comparator	-
2141	1999	Effects of Strength Training on Knee Osteoarthritis	No	Without intervention/comparator	Without intervention/comparator	-
2142	2000	Impact of Exercise on Older Persons With Osteoarthritis	No	Without intervention/comparator	Without intervention/comparator	-
2143	2000	Muscle Strengthening Device for Knee Osteoarthritis	No	Without intervention/comparator	Without intervention/comparator	-
2144	2002	Aerobic Exercise Intervention for Knee Osteoarthritis	No	Without intervention/comparator	Without intervention/comparator	-
2145	2002	Efficacy of Acupuncture With Physical Therapy for Knee Osteo-Arthritis	No	Without intervention/comparator	Without intervention/comparator	-
2146	2002	Safety Study of SCIO-469 to Treat Patients With Active Rheumatoid Arthritis Receiving Metho	No	Without intervention/comparator	Without intervention/comparator	-
2147	2003	Coping Skills Training for Early Rheumatoid Arthritis	No	Without intervention/comparator	Without intervention/comparator	-
2148	2003	Relaxation Response Training for the Treatment of Rheumatoid Arthritis	No	Without intervention/comparator	Without intervention/comparator	-
2149	2003	The Effect of Weight Loss and Exercise on Knee Osteoarthritis	No	Without intervention/comparator	Without intervention/comparator	-
2150	2004	Knee Stability Training for Knee Osteoarthritis (OA)	No	Without intervention/comparator	Without intervention/comparator	-
2151	2005	A Comparison of Fixation Method in Total Knee Arthroplasty - Low Viscosity Versus High Visc	No	Other populations/diseases	Other populations/diseases	-
2152	2005	A Multicentre Trial to Determine the Efficacy of AD 452 in RA Subjects	No	Without intervention/comparator	Without intervention/comparator	-
2153	2005	A Targeted Strengthening Program Following Total Hip Replacement	No	Other populations/diseases	Other populations/diseases	-

2154	2005	An RSA and DEXA Study on Migration of Proximal Interphalangeal (PIP) Joint Prostheses of th	No	Other populations/diseases	Other populations/diseases	-
2155	2005	Cost Efficacy of a Clinical Pathway to Patients Undergoing Hip and Knee Replacement Surgery	No	Other populations/diseases	Other populations/diseases	-
2156	2005	Couples-Oriented Psychosocial Intervention for Osteoarthritis	No	Without intervention/comparator	Without intervention/comparator	-
2157	2005	Effect of Sling Suspension Exercises in Proprioception of Patients With Knee Osteoarthritis	No	Without intervention/comparator	Without intervention/comparator	-
2158	2005	Effects of Pulsed Magnetic Pads on Rheumatoid Arthritis Symptoms in Postmenopausal Womer	No	Without intervention/comparator	Without intervention/comparator	-
2159	2005	Electrical Stimulation After Total Knee Arthroplasty	No	Other populations/diseases	Other populations/diseases	-
2160	2005	Exercise and Physical Fitness for Persons With Knee Osteoarthritis	No	Without intervention/comparator	Without intervention/comparator	-
2161	2005	Health Benefits of an Exercise Program for Adults With Arthritis	No	Without intervention/comparator	Without intervention/comparator	-
2162	2005	Home-Based Exercise and Weight Control Program for Pain Control in Overweight Elderly Witl	No	Without intervention/comparator	Without intervention/comparator	-
2163	2005	Program Evaluation of People With Arthritis Can Exercise	No	Without intervention/comparator	Without intervention/comparator	-
2164	2005	Qigong Therapy for Individuals With Knee Osteoarthritis	No	Without intervention/comparator	Without intervention/comparator	-
2165	2005	Rituximab for the Treatment of Refractory Adult and Juvenile Dermatomyositis (DM) and Adult	No	Other populations/diseases	Other populations/diseases	-
2166	2005	Walking Aids in the Management of Knee Osteoarthritis	No	Without intervention/comparator	Without intervention/comparator	-
2167	2006	A Study Comparing the Effectiveness and Safety of Varying Dose Strengths (100, 200, 300 and	No	Without intervention/comparator	Without intervention/comparator	-
2168	2006	Aquatic Exercise and Efficacy Enhancement to Decrease Fall Risk in Older Adults With Hip Os	No	Without intervention/comparator	Without intervention/comparator	-
2169	2006	Comparison of Two Resurfacing Prostheses in the Treatment of Osteoarthritis of the Shoulder	No	Without intervention/comparator	Without intervention/comparator	-
2170	2006	Effectiveness of Two Types of Treatment in Restoring Muscle After Hip or Knee Surgery	No	Other populations/diseases	Other populations/diseases	-
2171	2006	Efficacy Study of Condrosulf in the Treatment of Symptomatic OA of the Hand	No	Without intervention/comparator	Without intervention/comparator	-
2172	2006	Exercise Therapy and Patient Education for Individuals With Hip Osteoarthritis. a RCT	No	Without intervention/comparator	Without intervention/comparator	-
2173	2006	Intensive Diet and Exercise for Improving Knee Osteoarthritis in Obese and Overweight Older A	No	Without intervention/comparator	Without intervention/comparator	-
2174	2006	Is Botox Effective in Relieving Pain From Knee Osteoarthritis?	No	Without intervention/comparator	Without intervention/comparator	-
2175	2006	Knee Malalignment and Thigh Muscle Strengthening in Individuals With Medial Knee Arthritis	No	Without intervention/comparator	Without intervention/comparator	-
2176	2006	Massage Therapy for Osteoarthritis of the Knee	No	Without intervention/comparator	Without intervention/comparator	-
2177	2006	Pharmacist-initiated Intervention Trial in OsteoArthritis (PhIT-OA)	No	Without intervention/comparator	Without intervention/comparator	-
2178	2006	Preheating of Femur Component in Hybrid Total Hip Arthroplasty	No	Other populations/diseases	Other populations/diseases	-
2179	2006	Proximal Tibial Open Wedge Osteotomy. A Clinical Prospective, Randomized RSA-trial	No	Other populations/diseases	Other populations/diseases	-
2180	2006	Study of Effectiveness of Hand Exercises to Treat Symptoms of Hand Osteoarthritis	No	Without intervention/comparator	Without intervention/comparator	-
2181	2006	Tai Chi Mind-Body Therapy for Knee Osteoarthritis	No	Without intervention/comparator	Without intervention/comparator	-
2182	2006	The Effects of a 16-Week Individualized, Intensive Strength Training Program in Patients With I	No	Without intervention/comparator	Without intervention/comparator	-
2183	2006	Weight Management and Coping Skills Training For Patients With Knee Osteoarthritis	No	Without intervention/comparator	Without intervention/comparator	-
2184	2006	Yoga for Rheumatoid Arthritis	No	Without intervention/comparator	Without intervention/comparator	-
2185	2007	A Study of the Effectiveness of Different Types of Exercise for People With Knee Osteoarthritis	No	Without intervention/comparator	Without intervention/comparator	-
2186	2007	An Intervention of Electrical Stimulation in Osteoarthritis	No	Without intervention/comparator	Without intervention/comparator	-
2187	2007	ARTIST: aRThrose Intervention STandardisÃ©e	No	Without intervention/comparator	Without intervention/comparator	-
2188	2007	Chloroquine and Post Malaria Anaemia Study	No	Other populations/diseases	Other populations/diseases	-
2189	2007	Clinical Assessment of Two Manipulative Protocols in Treatment of Hip Osteoarthritis	No	Without intervention/comparator	Without intervention/comparator	-
2190	2007	Community-Based Programs for Improving Physical Function in People With Early Knee Osteo	No	Without intervention/comparator	Without intervention/comparator	-
2191	2007	Comparison of The Clinical Effects Between Different Physical Therapy Tools in Patellofemoral	No	Other populations/diseases	Other populations/diseases	-
2192	2007	Correlation of 6-Minute Walk Test and Neuromuscular Parameters in the Elderly	No	Other populations/diseases	Other populations/diseases	-
2193	2007	Effects of Aquamin F on Osteoarthritis of the Knee	No	Without intervention/comparator	Without intervention/comparator	-
2194	2007	Efficacy Study on Symptomatic Control of Patient With Knee Osteoarthritis Between 0.0125% c	No	Without intervention/comparator	Without intervention/comparator	-
2195	2007	Exercise Adherence Among Older Adults With Osteoarthritis	No	Without intervention/comparator	Without intervention/comparator	-
2196	2007	Exercise and Respiratory Therapy in Patients With Rheumatoid Arthritis / Collagenosis and Pulr	No	Without intervention/comparator	Without intervention/comparator	-
2197	2007	Initial Graft Tension and ACL Surgery	No	Other populations/diseases	Other populations/diseases	-

2198	2007	Muscle Function in Elderly Postoperative Patients	No	Other populations/diseases	Other populations/diseases	-
2199	2007	Operative or Conservative Treatment for Subacromial Impingement Syndrome?	No	Other populations/diseases	Other populations/diseases	-
2200	2007	Perioperative Intervention to Improve Post-TKR Support and Function	No	Other populations/diseases	Other populations/diseases	-
2201	2007	Pre-operative Rehabilitation Exercise Program for Total Knee Arthroplasty	No	Other populations/diseases	Other populations/diseases	-
2202	2007	Resistive Exercise for Arthritic Cartilage Health (REACH)	No	Without intervention/comparator	Without intervention/comparator	-
2203	2007	Rheumatoid Arthritis Patients in Training	No	Without intervention/comparator	Without intervention/comparator	-
2204	2007	The Clinical Effectiveness of Static Resting Splints in Early Rheumatoid Arthritis	No	Without intervention/comparator	Without intervention/comparator	-
2205	2007	The Effectiveness of Behavioral Graded Activity in Patients With Osteoarthritis of the Hip and/c	No	Without intervention/comparator	Without intervention/comparator	-
2206	2008	A Migration and Bone Density Study Comparing 2 Types of Bone Cement in the OptiPac Bone	No	Other populations/diseases	Other populations/diseases	-
2207	2008	An Examination of the Value of Shortwave Diathermy and Hydrotherapy for Patients With Oste	No	Without intervention/comparator	Without intervention/comparator	-
2208	2008	BION Treatment of Knee Osteoarthritis	No	Without intervention/comparator	Without intervention/comparator	-
2209	2008	Chromium Piccolinate in the Prevention of Weight Gain Induced by Serotonergic Medications Ir	No	Other populations/diseases	Other populations/diseases	-
2210	2008	CMC (Carpometacarpal) OA (Osteoarthritis) Thumb Splint Study	No	Without intervention/comparator	Without intervention/comparator	-
2211	2008	Comparing Knee Cartilage Surgery Versus Standard Physical Therapy in Treating People With a	No	Without intervention/comparator	Without intervention/comparator	-
2212	2008	Early Neuromuscular Electrical Stimulation For Quadriceps Muscle Activation Deficits Followin	No	Other populations/diseases	Other populations/diseases	-
2213	2008	Effect of Physiotherapy After Total Knee Replacement	No	Other populations/diseases	Other populations/diseases	-
2214	2008	Effectiveness and Cost-Efficiency of Aquatic Exercise for Management of Osteoarthritis	No	Without intervention/comparator	Without intervention/comparator	-
2215	2008	Effectiveness of Minimally Invasive Total Knee Replacement in Improving Rehabilitation and F	No	Other populations/diseases	Other populations/diseases	-
2216	2008	Effects of an Herbal Topical Cream on Osteoarthritis Symptoms, Biomarkers, and Disease Progr	No	Without intervention/comparator	Without intervention/comparator	-
2217	2008	Effects of Creatine Supplementation in Women With Knee Osteoarthritis and Fibromyalgia	No	Without intervention/comparator	Without intervention/comparator	-
2218	2008	Effects of Proximal and Distal Tibiofibular Joint Manipulation on Lower Extremity Muscle Acti	No	Other populations/diseases	Without intervention/comparator	Other populations/dis
2219	2008	Efficacy and Safety of Celecoxib Versus Placebo in the Treatment of Patients With Osteoarthriti	No	Without intervention/comparator	Without intervention/comparator	-
2220	2008	EMPART - Exercise and Manual Physiotherapy Arthritis Research Trial	No	Without intervention/comparator	Without intervention/comparator	-
2221	2008	Exercise Plus Activity Strategy Training for Osteoarthritis	No	Without intervention/comparator	Without intervention/comparator	-
2222	2008	Group Physical Therapy for Knee Osteoarthritis	No	Without intervention/comparator	Without intervention/comparator	-
2223	2008	Hip Osteoarthritis: effects of Exercise Programs on Pain and Disability	No	Without intervention/comparator	Without intervention/comparator	-
2224	2008	Influence of Weight Loss or Exercise on Cartilage in Obese Knee Osteoarthritis Patients	No	Without intervention/comparator	Without intervention/comparator	-
2225	2008	Muscle Regrowth During Physical Rehabilitation and Amino Acid Supplementation	No	Without intervention/comparator	Without intervention/comparator	-
2226	2008	Patterned Electrical Neuromuscular Stimulation and Therapeutic Exercise for Osteoarthritis of th	No	Without intervention/comparator	Without intervention/comparator	-
2227	2008	PNF Stretching for TKA on ROM	No	Without intervention/comparator	Without intervention/comparator	-
2228	2008	PRCT: ligament Reconstruction & Tendon Interposition With a Joint Spacer for Trapeziometaca	No	Other populations/diseases	Other populations/diseases	-
2229	2008	Progressive Exercise After Total Knee Arthroplasty (TKA)	No	Other populations/diseases	Other populations/diseases	-
2230	2008	Prolotherapy Versus Steroids for Thumb Carpo-metacarpal Joint Arthritis	No	Without intervention/comparator	Without intervention/comparator	-
2231	2008	Safety and Efficacy of Celecoxib Versus Placebo in the Treatment of Knee Osteoarthritis in Pati	No	Without intervention/comparator	Without intervention/comparator	-
2232	2008	Yesple Home-Based Exercise for Knee Osteoarthritis	No	Without intervention/comparator	Without intervention/comparator	-
2233	2008	Sleep in Osteoarthritis Project	No	Without intervention/comparator	Without intervention/comparator	-
2234	2008	Study of the Effect of Neck Treatment on Shoulder Impingement	No	Other populations/diseases	Other populations/diseases	-
2235	2008	The Effects of Home-Based Rehabilitation Treatments Among Persons With Symptomatic Knee	No	Without intervention/comparator	Without intervention/comparator	-
2236	2008	Zurich Multiple Endpoint Vitamin D Trial in Knee OA Patients	No	Without intervention/comparator	Without intervention/comparator	-
2237	2009	A Clinical Study to Investigate the Effect on Pain Relief of a Single Dose of JNJ-39439335 in Pa	No	Without intervention/comparator	Without intervention/comparator	-
2238	2009	Conservative Treatment for Hip Osteoarthritis	No	Without intervention/comparator	Without intervention/comparator	-
2239	2009	Effect of an Education Programme for Patients With Osteoarthritis in Primary Care - a Randomi	No	Without intervention/comparator	Without intervention/comparator	-
2240	2009	Effect of Glucosamine or Ibuprofen Combined With Physical Training in Patients With Knee-Os	No	Without intervention/comparator	Without intervention/comparator	-
2241	2009	Effect of Pre-surgery Neuromuscular Physiotherapy (PT)	No	Other populations/diseases	Other populations/diseases	-

2242	2009	Effectiveness of Acupuncture as an Adjunct to Rehabilitation After Knee Arthroplasty	No	Other populations/diseases	Other populations/diseases	-
2243	2009	Evaluate Safety & Efficacy of Condroflex in Subjects With OA	No	Without intervention/comparator	Without intervention/comparator	-
2244	2009	Influence of Whole Body Vibration Compared to Conventional Physiotherapy in Patients With (	No	Other populations/diseases	Other populations/diseases	-
2245	2009	Intradialytic Progressive Resistance Training for Maintenance Haemodialysis Patients	No	Other populations/diseases	Other populations/diseases	-
2246	2009	Manual Therapy Versus Exercise on Knee Osteoarthritis	No	Without intervention/comparator	Without intervention/comparator	-
2247	2009	Mobility Optimization Through Velocity Exercise	No	Other populations/diseases	Other populations/diseases	-
2248	2009	Perioperative Iron With Erythropoietin in Bilateral Total Knee Replacement Arthroplasty (TKRA	No	Other populations/diseases	Other populations/diseases	-
2249	2009	Placebo-controlled Trial With OROS Hydromorphone Hydrochloride to Treat Patients With Mo	No	Without intervention/comparator	Without intervention/comparator	-
2250	2009	Preoperative Exercise in Patients Undergoing Total Hip or Knee Replacement	No	Other populations/diseases	Other populations/diseases	-
2251	2009	Stick Versus Quadricep Exercise for Knee Osteoarthritis	No	Without intervention/comparator	Without intervention/comparator	-
2252	2009	Study Examining the Effect of Exercise in People With Rheumatoid Arthritis Taking Anti-TNFa	No	Without intervention/comparator	Without intervention/comparator	-
2253	2009	Surgical or Exercise Therapy on Patients With Degenerative Meniscus Tears	No	Other populations/diseases	Other populations/diseases	-
2254	2009	The Arthritis, Diet, and Activity Promotion Trial	No	Without intervention/comparator	Without intervention/comparator	-
2255	2009	The Use of Neuromuscular Electrical Stimulation to Reverse Muscle Atrophy in Patients With R	No	Without intervention/comparator	Without intervention/comparator	-
2256	2010	Biomarkers and Knee Osteoarthritis	No	Without intervention/comparator	Without intervention/comparator	-
2257	2010	Botulism Toxin Injection as a Treatment for Arthritis of the Basal Thumb Joint	No	Without intervention/comparator	Without intervention/comparator	-
2258	2010	Comparing Manipulation, Rehabilitation and Combination of the Two in the Treatment of Knee	No	Without intervention/comparator	Without intervention/comparator	-
2259	2010	Comparison of Quadriceps-sparing Minimally Invasive and Medial Parapatellar Total Knee Arth	No	Without intervention/comparator	Without intervention/comparator	-
2260	2010	Continuous Saphenous Block Versus Continuous Femoral Block for Total Knee Arthroplasty	No	Without intervention/comparator	Without intervention/comparator	-
2261	2010	Early Rehabilitation After Total Hip Replacement	No	Without intervention/comparator	Without intervention/comparator	-
2262	2010	Effectiveness of Community-Based Physiotherapy Versus General Practice	No	Without intervention/comparator	Without intervention/comparator	-
2263	2010	Effects of Kneehab 12-week Peri-operative Total Knee Arthroplasty	No	Without intervention/comparator	Without intervention/comparator	-
2264	2010	Effects of Transcutaneous Electrical Nerve Stimulation on Pain and Disability in Patients With (	No	Without intervention/comparator	Without intervention/comparator	-
2265	2010	Efficacy of Tart Cherry Juice to Reduce Pain and Inflammation Among Patients With Inflammat	No	Without intervention/comparator	Without intervention/comparator	-
2266	2010	Efficacy of Zoledronic Acid in Enhancement of Early Stability of Cementless Primary Hip Prost	No	Without intervention/comparator	Without intervention/comparator	-
2267	2010	Exercise Therapy and Patient Education for Individuals With Hip Osteoarthritis. Long-term Foll	No	Without intervention/comparator	Without intervention/comparator	-
2268	2010	Group Exercise After Hip Replacement Surgery	No	Without intervention/comparator	Without intervention/comparator	-
2269	2010	Group Physical Therapy for Knee Osteoarthritis	No	Without intervention/comparator	Without intervention/comparator	-
2270	2010	Pain Inhibition in Patients With Rheumatoid Arthritis and Central Sensitivity Syndromes	No	Without intervention/comparator	Without intervention/comparator	-
2271	2010	Patient and Provider Interventions for Managing Osteoarthritis in Primary Care	No	Without intervention/comparator	Without intervention/comparator	-
2272	2010	Patient Education Program for Osteoarthritis With Exercise Included	No	Without intervention/comparator	Without intervention/comparator	-
2273	2010	Platform Exercise Training	No	Without intervention/comparator	Without intervention/comparator	-
2274	2010	Preoperative Resistance Training in Patients Scheduled for Total Hip Arthroplasty	No	Without intervention/comparator	Without intervention/comparator	-
2275	2010	Regenerative Injection Therapy and Osteoarthritis	No	Without intervention/comparator	Without intervention/comparator	-
2276	2010	Regulation of Intraarticular and Synovium-related Biomarkers of Osteoarthritis. Effect of Acute	No	Without intervention/comparator	Without intervention/comparator	-
2277	2010	Resistance Exercise and Knee Osteoarthritis Pain, Functional Impairment and Cartilage Turnove	No	Without intervention/comparator	Without intervention/comparator	-
2278	2010	Resistance Training in Knee Osteoarthritis	No	Without intervention/comparator	Without intervention/comparator	-
2279	2010	Self-Directed Exercise Program for Adults With Arthritis	No	Without intervention/comparator	Without intervention/comparator	-
2280	2010	Tai Chi and Physical Therapy for Knee Osteoarthritis	No	Without intervention/comparator	Without intervention/comparator	-
2281	2010	The Benefit of Arthroscopic Partial Meniscectomy in Middle-Aged Patients	No	Without intervention/comparator	Without intervention/comparator	-
2282	2010	The Effects of the Electro, Heat and Cold -Therapy During Physiotherapy Treatment in Osteoartl	No	Without intervention/comparator	Without intervention/comparator	-
2283	2010	The Efficacy of Viscosupplementation for Early Knee Osteoarthritis	No	Without intervention/comparator	Without intervention/comparator	-
2284	2010	The Hand Osteoarthritis Exercise Trial	No	Without intervention/comparator	Without intervention/comparator	-
2285	2010	VItamin D Effect on Osteoarthritis Study	No	Without intervention/comparator	Without intervention/comparator	-

2286	2011	A Comparison of Manual Physical Therapy and Corticosteroid Injections for Knee Osteoarthritis	No	Without intervention/comparator	Without intervention/comparator	-
2287	2011	A Study To Compare The Amount Of CP-690,550 That Is Absorbed Into The Blood Of Healthy	No	Without intervention/comparator	Without intervention/comparator	-
2288	2011	A Study to Determine if Aquamin Modulates Inflammatory Biomarkers in the Blood of Osteoart	No	Without intervention/comparator	Without intervention/comparator	-
**2289**	**2011**	**Assessment of Efficacy of Low Intensity Resistance Training in Men at Risk for Symptoma**	**Yes**	**Full**	**Full**	**-**
**2290**	**2011**	**Assessment of Efficacy of Low Intensity Resistance Training in Women at Risk for Sympto**	**Yes**	**Full**	**Full**	**-**
2291	2011	Biomechanics of Gait Pattern Adaptation in Patients After Total Knee Arthroplasty	No	Without intervention/comparator	Without intervention/comparator	-
2292	2011	Bone Mineral Density (BMD) in Cemented Versus Cementless Hip Resurfacing	No	Without intervention/comparator	Without intervention/comparator	-
2293	2011	Can Computer-based Telephone Counseling Improve Long-term Adherence to Strength Training	No	Without intervention/comparator	Without intervention/comparator	-
2294	2011	Changes in Quadriceps Function Following Local or Distant Interventions in Individuals With Pa	No	Without intervention/comparator	Without intervention/comparator	-
2295	2011	Changes Of Sleep on the Sensoriomotor and Cytokine In Patients With Osteoarthritis	No	Without intervention/comparator	Without intervention/comparator	-
2296	2011	Development of a Translational Tool to Study Yoga Therapy	No	Without intervention/comparator	Without intervention/comparator	-
2297	2011	Early Progressive Strength Training to Patients With Unicompartmental Knee Replacement	No	Without intervention/comparator	Without intervention/comparator	-
2298	2011	Echogenic Versus Stimulating Needle and Catheter for Sciatic Blocks	No	Without intervention/comparator	Without intervention/comparator	-
2299	2011	Effect of Footwear on the Clinical, Functional, and Biomechanical Aspects in Elderly Women W	No	Without intervention/comparator	Without intervention/comparator	-
2300	2011	Effectiveness of Hemi- Versus Total Shoulder Arthroplasty Using Implants of the "Epoca" Syste	No	Without intervention/comparator	Without intervention/comparator	-
2301	2011	Effectiveness of Water Exercises on Isokinetic Muscle Strength	No	Without intervention/comparator	Without intervention/comparator	-
2302	2011	Effects of a Rehabilitation Program on Physical Performance and Disease Self-management in R	No	Without intervention/comparator	Without intervention/comparator	-
2303	2011	Effects of Glucosamine and Chondroitin Supplementation in Women With Knee Osteoarthritis F	No	Without intervention/comparator	Without intervention/comparator	-
2304	2011	Enhancing the Effectiveness of Physical Therapy for People With Knee Osteoarthritis	No	Without intervention/comparator	Without intervention/comparator	-
2305	2011	Exercise and Muscle Stimulation in Patients With Knee Osteoarthritis	No	Without intervention/comparator	Without intervention/comparator	-
2306	2011	Exercise and Pain Sensitivity	No	Without intervention/comparator	Without intervention/comparator	-
2307	2011	Exercise in Patients With Osteoarthritis of the Hip	No	Without intervention/comparator	Without intervention/comparator	-
2308	2011	Exercise-induced Improvements of Inflammatory Status in Patients With Rheumatoid Arthritis	No	Without intervention/comparator	Without intervention/comparator	-
2309	2011	Galileo-Hip Whole Body Vibration /Conventional Physiotherapy /Coxarthrosis	No	Without intervention/comparator	Without intervention/comparator	-
2310	2011	High-speed Power Training in Older Adults With Knee Osteoarthritis (OA)	No	Without intervention/comparator	Without intervention/comparator	-
2311	2011	Knee Osteoarthritis (OA) Project Treatment Versus Conventional Physical Therapy in the Treatm	No	Without intervention/comparator	Without intervention/comparator	-
2312	2011	Local Infiltration Versus Block Against Pains After High Tibial Osteotomy	No	Other populations/diseases	Other populations/diseases	-
2313	2011	Low Intensity Resistance Training With Partial Blood Flow Restriction for Quadriceps Strengthe	No	Other populations/diseases	Other populations/diseases	-
2314	2011	Low Power Laser and Exercise in Osteoarthritis of the Knee: a Randomized Clinical Trial	No	Without intervention/comparator	Without intervention/comparator	-
2315	2011	Minimally Invasive Surgical Approaches In Total Knee Arthroplasty	No	Without intervention/comparator	Without intervention/comparator	-
2316	2011	MOVE OUT: a Partnership With Veterans Groups to Enhance Weight Management in the Veter	No	Without intervention/comparator	Without intervention/comparator	-
2317	2011	Moxibustion for Knee Osteoarthritis	No	Without intervention/comparator	Without intervention/comparator	-
2318	2011	Neuromuscular Electrical Stimulation and Strength Training in Patients With Knee Osteoarthriti	No	Without intervention/comparator	Without intervention/comparator	-
2319	2011	Patient and Provider Interventions for Managing Osteoarthritis in Primary Care	No	Without intervention/comparator	Without intervention/comparator	-
2320	2011	Rapid Easy Strength Training (REST) to Improve Function in Late Stage Cancer	No	Other populations/diseases	Other populations/diseases	-
2321	2011	Single- vs. Double-Bundle ACL Reconstruction	No	Without intervention/comparator	Without intervention/comparator	-
2322	2011	Staying Active With Arthritis: RCT of Physical Activity for Older Adults With Osteoarthritis an	No	Without intervention/comparator	Without intervention/comparator	-
2323	2011	Strength Training for ARthritis Trial	No	Without intervention/comparator	Without intervention/comparator	-
2324	2011	Structured Treatment of Osteoarthritis of the Knee With or Without Total Knee Replacement	No	Without intervention/comparator	Without intervention/comparator	-
2325	2011	The Difference Between Rehabilitation With or Without Strength Training After Total Knee Rep	No	Without intervention/comparator	Without intervention/comparator	-
2326	2011	The Effect of 6 Months of Local Vibration Training in Institutionalized Elderly	No	Without intervention/comparator	Without intervention/comparator	-
2327	2011	The Effect of Early WBVT on Neuromuscular Control After ACLR	No	Without intervention/comparator	Without intervention/comparator	-
2328	2011	The Immune and Clinical Impacts of Vitamin D in Patients With Chronic Musculo-skeletal Pain	No	Without intervention/comparator	Without intervention/comparator	-
2329	2011	The Recovery Time of Quadriceps Strength After Total Knee Arthroplasty (TKA): the Effect of (	No	Without intervention/comparator	Without intervention/comparator	-

2330	2011	Time to Total Hip Replacement After Supervised Exercise and Patient Education	No	Without intervention/comparator	Without intervention/comparator	-
**2331**	**2011**	**Vascular Occlusion and Rheumatoid Arthritis**	**Yes**	**Full**	**Full**	**-**
**2332**	**2011**	**Vascular Occlusion in Patients With Osteoarthritis**	**Yes**	**Full**	**Full**	**-**
2333	2011	Web-based Support to Manage Arthritis Pain	No	Without intervention/comparator	Without intervention/comparator	-
2334	2012	A Comparative Analysis of Two Types of Exercise on Outcomes Following Total Knee Arthropl	No	Without intervention/comparator	Without intervention/comparator	-
2335	2012	A Study of the Effect of Supervised Exercise Programme in Patients With Rheumatoid Arthritis	No	Without intervention/comparator	Without intervention/comparator	-
2336	2012	Assessing the Impact of Isokinetic Muscular Strengthening in Eccentric Mode in the Medical Tr	No	Without intervention/comparator	Without intervention/comparator	-
2337	2012	Can Shoulder Arthroscopy Work	No	Without intervention/comparator	Without intervention/comparator	-
2338	2012	Comparing the Clinical Effects of Posterior Approach Versus Lateral Approach in Osteoarthritis	No	Without intervention/comparator	Without intervention/comparator	-
2339	2012	Dose-response: exercise Therapy on Hip Osteoarthritis	No	Without intervention/comparator	Without intervention/comparator	-
2340	2012	Effect of Cycle Ergometer in the Rehabilitation of Elderly Patients With Total Hip Arthroplasty	No	Without intervention/comparator	Without intervention/comparator	-
2341	2012	Effect of Exercise as Non-surgical Treatments on Time to Total Hip Replacement Surgery	No	Without intervention/comparator	Without intervention/comparator	-
2342	2012	Effect of Tai Chi on Osteoarthritic Knee Pain in Elders With Mild Dementia	No	Without intervention/comparator	Without intervention/comparator	-
2343	2012	Effectiveness of Exercise Programs Following Total Hip and Knee Joint Arthroplasty	No	Without intervention/comparator	Without intervention/comparator	-
2344	2012	Effects of Intraarticular Botulinum Toxin A in Ankle Osteoarthritis	No	Without intervention/comparator	Without intervention/comparator	-
2345	2012	Efficacy and Safety of Synera in Osteoarthritis Pain	No	Without intervention/comparator	Without intervention/comparator	-
2346	2012	Efficacy of Exercise on Quality of Life and Physical Function in Patients With Knee Osteoarthri	No	Without intervention/comparator	Without intervention/comparator	-
2347	2012	Evaluation of an Educational Program Associated With Exercises (EDEX) Before Total Knee Ar	No	Without intervention/comparator	Without intervention/comparator	-
2348	2012	Evaluation of Bisphosphonate Coated Pins for Extern Fixation in Tibia Osteotomy	No	Without intervention/comparator	Without intervention/comparator	-
2349	2012	Exercise and Pain Sensitivity in Knee Osteoarthritis	No	Without intervention/comparator	Without intervention/comparator	-
2350	2012	Home Based Computer Gaming Hand Exercise Regimen for People With Arthritis Affecting the	No	Without intervention/comparator	Without intervention/comparator	-
2351	2012	Impact of Self-care Education Program in Patients With Type 2 Diabetes in Primary Care in the	No	Other populations/diseases	Other populations/diseases	-
2352	2012	Influence of Manual Lymph Drainage During Hospitalization on Swelling, Function and Pain in	No	Without intervention/comparator	Without intervention/comparator	-
2353	2012	Interactive Virtual Telerehabilitation After Total Knee Arthroplasty	No	Without intervention/comparator	Without intervention/comparator	-
2354	2012	Internet Intervention to Improve Physical Activity in Early Knee Osteoarthritis	No	Without intervention/comparator	Without intervention/comparator	-
2355	2012	Low-level Laser Therapy and Static Stretching in Knee Osteoarthritis	No	Without intervention/comparator	Without intervention/comparator	-
2356	2012	Nuclear Magnetic Resonance Therapy in Knee Osteoarthrosis	No	Without intervention/comparator	Without intervention/comparator	-
2357	2012	Postoperative Therapy After Interposition Arthroplasty in CMC1	No	Without intervention/comparator	Without intervention/comparator	-
2358	2012	Preoperative Strength Training in Patients With Total Knee Arthroplasty	No	Without intervention/comparator	Without intervention/comparator	-
2359	2012	Progressive Rehabilitation Following Total Knee Arthroplasty	No	Without intervention/comparator	Without intervention/comparator	-
2360	2012	Project Osteoarthritis: recovering Quality of Life Through Education	No	Without intervention/comparator	Without intervention/comparator	-
2361	2012	Randomized Evaluation of the Efficacy of Synvisc-OneÂ® for the Treatment of Patellofemoral (	No	Other populations/diseases	Other populations/diseases	-
2362	2012	RAPID: reducing Pain; Preventing Depression	No	Other populations/diseases	Other populations/diseases	-
2363	2012	S1200: acupuncture, Sham Acupuncture, or Wait List for Joint Symptoms Related to Aromatase	No	Other populations/diseases	Other populations/diseases	-
2364	2012	Sensorimotor Training Versus Resistance Training in Patients With Knee Osteoarthritis	No	Without intervention/comparator	Without intervention/comparator	-
2365	2012	Serum Cartilage Oligomeric Matrix Protein Levels After 12 Weeks of Different Exercises	No	Without intervention/comparator	Without intervention/comparator	-
2366	2012	Spa Therapy in Knee Osteoarthritis (OA): nancy-thermal	No	Without intervention/comparator	Without intervention/comparator	-
2367	2012	Strengthening Exercise and Quadriceps Force During Walking	No	Without intervention/comparator	Without intervention/comparator	-
2368	2012	Structured Non-operative Treatment of Knee Osteoarthritis	No	Without intervention/comparator	Without intervention/comparator	-
2369	2012	Testosterone Administration and ACL Reconstruction in Men	No	Other populations/diseases	Other populations/diseases	-
2370	2012	The Effect on Knee Joint Loads of Analgesic Use Compared With Exercise in Patients With Kne	No	Without intervention/comparator	Without intervention/comparator	-
2371	2012	The Effects of Cardiac Rehabilitation on Cardiovascular Disease Risk in Rheumatoid Arthritis P	No	Without intervention/comparator	Without intervention/comparator	-
2372	2012	The McKenzie System With Arthritic Knees: do Some Knees Respond to Specific Exercise Mor	No	Without intervention/comparator	Without intervention/comparator	-
2373	2012	Yoga as Self-Care for Arthritis in Minority Communities	No	Without intervention/comparator	Without intervention/comparator	-

2374	2013	Adductor Canal Block With Posterior Capsular Injection for Total Knee Replacement	No	Without intervention/comparator	Without intervention/comparator	-
2375	2013	An Exploratory Study of PRX167700 in Patients With Knee Osteoarthritis	No	Without intervention/comparator	Without intervention/comparator	-
2376	2013	An Intelligent Instrument for Improved Leg Length and Hip Offset Accuracy in Total Hip Arthro	No	Without intervention/comparator	Without intervention/comparator	-
2377	2013	Aquatic Exercise Program for Knee Osteoarthritis	No	Without intervention/comparator	Without intervention/comparator	-
2378	2013	Can Creatine Supplementation Improve Body Composition and Physical Function in Rheumatoi	No	Without intervention/comparator	Without intervention/comparator	-
2379	2013	Comparison of Fascial Manipulation With Traditional Physiotherapy for the Treatment of Trigge	No	Without intervention/comparator	Without intervention/comparator	-
2380	2013	Conservative Management of Femoroacetabular Impingement	No	Without intervention/comparator	Without intervention/comparator	-
2381	2013	DEMAND - DEgenerative Meniscal Tears - Arthroscopy vs. Dedicated Exercise	No	Without intervention/comparator	Without intervention/comparator	-
2382	2013	Does Prescriptive Treatment of the Hips Improve Outcomes in Patients With Low Back Pain? A	No	Without intervention/comparator	Without intervention/comparator	-
2383	2013	Effect of Moderate to High Intensity Aerobic Interval Training on Polysomnographic Measured S	No	Without intervention/comparator	Without intervention/comparator	-
2384	2013	Effective Rehabilitation of Patients Operated With Total Knee Arthroplasty	No	Without intervention/comparator	Without intervention/comparator	-
2385	2013	Effectiveness and Safety of Physical Exercises in the Improvement of the Sleep Quality. Regardi	No	Without intervention/comparator	Without intervention/comparator	-
2386	2013	Effectiveness of Resistive Capacitive Diathermy in Patients Affected by Knee Osteoarthritis	No	Without intervention/comparator	Without intervention/comparator	-
2387	2013	Effects of DHA on Pro-resolving Anti-inflammatory Mediators in Obese Patients Undergoing W	No	Without intervention/comparator	Without intervention/comparator	-
2388	2013	Effects of RM-493 on Energy Expenditure in Obese Individuals	No	Without intervention/comparator	Without intervention/comparator	-
2389	2013	Exercise and Steroid in Knee Osteoarthritis	No	Without intervention/comparator	Without intervention/comparator	-
2390	2013	Exercise Therapy in Patients With Knee Osteoarthritis	No	Without intervention/comparator	Without intervention/comparator	-
2391	2013	Functional Movement Retraining After Hip Replacement	No	Without intervention/comparator	Without intervention/comparator	-
2392	2013	Independent Exercise Compared With Formal Rehabilitation Following Primary Total Knee Rep	No	Without intervention/comparator	Without intervention/comparator	-
2393	2013	Inflammation Inhibition in Prediabetic Humans	No	Without intervention/comparator	Without intervention/comparator	-
2394	2013	Obesity Surgery, Counseling, and Psychological Well-Being	No	Without intervention/comparator	Without intervention/comparator	-
2395	2013	Occupational Therapy and Surgery in Carpometacarpal Osteoarthritis	No	Without intervention/comparator	Without intervention/comparator	-
2396	2013	Outcome Comparison of Allograft and Synthetic Bone Substitute in High Tibial Osteotomy	No	Without intervention/comparator	Without intervention/comparator	-
2397	2013	Pedometer Based Intervention After Total Hip Replacement-A Pilot Study	No	Without intervention/comparator	Without intervention/comparator	-
2398	2013	Pilot Study of Therapy With Hylan G-F 20 Exercise Capacity	No	Without intervention/comparator	Without intervention/comparator	-
2399	2013	Progressive Collective-exercise Program on the Knee Osteoarthritis	No	Without intervention/comparator	Without intervention/comparator	-
2400	2013	Reduction of Daily Sitting Time in Patients With Rheumatoid Arthritis	No	Without intervention/comparator	Without intervention/comparator	-
2401	2013	Regular Swimming, Vascular Function, and Arthritis	No	Without intervention/comparator	Without intervention/comparator	-
2402	2013	Rotator Cuff Sparing Total Arthroplasty	No	Without intervention/comparator	Without intervention/comparator	-
2403	2013	Short-wave Diathermy in Patients With Osteoarthritis of the Hand	No	Without intervention/comparator	Without intervention/comparator	-
2404	2013	Study of Cartilage Relaxometry and Physical Activity in Osteoarthritis	No	Without intervention/comparator	Without intervention/comparator	-
2405	2013	Targeted Rehabilitation to Improve Outcome After Knee Replacement- A Physiotherapy Study	No	Without intervention/comparator	Without intervention/comparator	-
2406	2013	The Effect of Exercise Training in the Community-dwelling Adults With Chronic Disorders	No	Without intervention/comparator	Without intervention/comparator	-
2407	2013	The Impact of a Pre-Operative Exercise Program on Fitness Outcomes Following Bariatric Surge	No	Other populations/diseases	Other populations/diseases	-
2408	2013	Trial of a Physical Activity Intervention for RA Fatigue	No	Without intervention/comparator	Without intervention/comparator	-
2409	2013	Use of FlexHD as Post Trapeziectomy Spacer	No	Without intervention/comparator	Without intervention/comparator	-
2410	2013	Yoga for Managing Knee Osteoarthritis in Older Women: a Feasibility Study	No	Without intervention/comparator	Without intervention/comparator	-
2411	2014	A Randomised Controlled Trial Comparing Single Shot Adductor Canal Block With Local Infilt	No	Without intervention/comparator	Without intervention/comparator	-
2412	2014	A RCT of Supportive Finger Tape for PIPJ Osteoarthritis	No	Without intervention/comparator	Without intervention/comparator	-
2413	2014	Adductor Canal Versus Femoral Nerve Block for Analgesia Post Total Knee Arthroscopy	No	Without intervention/comparator	Without intervention/comparator	-
2414	2014	Alterations in Muscle After Total Knee Arthroplasty	No	Without intervention/comparator	Without intervention/comparator	-
2415	2014	Arthritis Pilot for Preserving Muscle While Losing Weight	No	Without intervention/comparator	Without intervention/comparator	-
2416	2014	Attune With TruMatch TM Personalized Solutions Instruments	No	Without intervention/comparator	Without intervention/comparator	-
2417	2014	Capsulectomy vs Capsulotomy With Repair in Direct Anterior Total Hip Arthroplasty	No	Without intervention/comparator	Without intervention/comparator	-

2418	2014	Close Kinect Chain Exercise With Kinesio Taping in the Management of Patellofemoral Pain Sy	No	Without intervention/comparator	Without intervention/comparator	-
2419	2014	Comparison Between Land-based and Water-based Exercises for Knee Osteoarthritis: randomize	No	Without intervention/comparator	Without intervention/comparator	-
2420	2014	Comparison of Treatments Following Total Knee Replacement	No	Without intervention/comparator	Without intervention/comparator	-
2421	2014	Context Effects in Exercise Therapy for Knee and/or Hip Pain	No	Without intervention/comparator	Without intervention/comparator	-
2422	2014	Corticosteroid Intra-articular Injection in Hands Osteoarthritis	No	Without intervention/comparator	Without intervention/comparator	-
2423	2014	Creatine, Exercise and Inflammatory Markers in Knee Osteoarthritis	No	Without intervention/comparator	Without intervention/comparator	-
2424	2014	Eccentric Exercise and Cachexia in Rheumatoid Arthritis	No	Without intervention/comparator	Without intervention/comparator	-
2425	2014	Effect of a Home-based Electrical Stimulation on Quadriceps Function After Knee Surgery	No	Without intervention/comparator	Without intervention/comparator	-
2426	2014	Effect of Adductor Canal Nerve Block Compared to a Yesulated Block on Quadriceps Strength I	No	Without intervention/comparator	Without intervention/comparator	-
2427	2014	Effect of Laser Acupuncture on Obesity	No	Other populations/diseases	Other populations/diseases	-
2428	2014	Effect of Methylprednisolone on Quadriceps Muscle Function in Patients Undergoing Total Kne	No	Without intervention/comparator	Without intervention/comparator	-
2429	2014	Effect of Phototherapy Incorporated Into an Exercise Program on Osteoarthritis of the Knee	No	Without intervention/comparator	Without intervention/comparator	-
2430	2014	Effectiveness of Aquatic Physical Therapy for Knee Osteoarthritis Patients	No	Without intervention/comparator	Without intervention/comparator	-
2431	2014	Effectiveness of Passive and Active ROM Exercises Following TKA	No	Without intervention/comparator	Without intervention/comparator	-
2432	2014	Effects of Elastic Therapeutic Tape on Biomechanical Changes of Knee Joint During Drop Verti	No	Other populations/diseases	Other populations/diseases	-
2433	2014	Efficacy and Safety of Postoperative Intravenous Parecoxib Sodium Followed by Oral Celecoxib	No	Without intervention/comparator	Without intervention/comparator	-
2434	2014	Evaluation of Motor Sparing Knee Block to Infiltration Analgesia for Pain Following Knee Arthi	No	Without intervention/comparator	Without intervention/comparator	-
2435	2014	Exercise and Cycle Ergometry Post TKA - A Randomized Controlled Trial	No	Without intervention/comparator	Without intervention/comparator	-
2436	2014	Fatigue in People With Rheumatoid Arthritis- Randomized Controlled Trial	No	Without intervention/comparator	Without intervention/comparator	-
2437	2014	Hand Exercises for Women With Rheumatoid Arthritis and Impaired ADL Ability: an Explorato	No	Without intervention/comparator	Without intervention/comparator	-
2438	2014	Health Benefits of a 6-month Brisk Walking Program in Sedentary Postmenopausal Women	No	Without intervention/comparator	Without intervention/comparator	-
2439	2014	Hypoxic Training in Obese Patients	No	Without intervention/comparator	Without intervention/comparator	-
2440	2014	MONITOR-OA: using Wearable Activity Trackers to Improve Physical Activity in Knee Osteoar	No	Without intervention/comparator	Without intervention/comparator	-
2441	2014	Negative Work Exercise for the Treatment of Knee Arthritis	No	Without intervention/comparator	Without intervention/comparator	-
2442	2014	Neurocognitive Rehabilitation After Hip Replacement	No	Without intervention/comparator	Without intervention/comparator	-
2443	2014	Neuroscience Education on Osteoarthritis	No	Without intervention/comparator	Without intervention/comparator	-
2444	2014	Outcomes Following Tourniquet and Non-Tourniquet Assisted Total Knee Arthroplasty	No	Without intervention/comparator	Without intervention/comparator	-
2445	2014	Physical Therapy Versus Internet-Based Exercise Training for Patients With Knee Osteoarthritis	No	Without intervention/comparator	Without intervention/comparator	-
2446	2014	PILOT STUDY: gait and Functional Improvement in Total Knee Arthroplasty With the Use of V	No	Without intervention/comparator	Without intervention/comparator	-
2447	2014	Progressive Resistance Exercise in Rheumatoid Arthritis	No	Without intervention/comparator	Without intervention/comparator	-
2448	2014	Project Arthritis Recovering Quality of Life by Means Education II (PARQVE II)	No	Without intervention/comparator	Without intervention/comparator	-
2449	2014	Regenexxâ¢ SD Versus Exercise Therapy for Treatment of Knee Osteoarthritis With Historical C	No	Without intervention/comparator	Without intervention/comparator	-
2450	2014	Repair of Medial Patellofemoral Ligament Compared to Conservative Treatment for First Time I	No	Without intervention/comparator	Without intervention/comparator	-
2451	2014	Synergic Effects of Ultrasound and Laser on the Pain Relief and Functionality	No	Without intervention/comparator	Without intervention/comparator	-
2452	2014	The Effect of Flexible Tape in Knee Osteoarthritis	No	Without intervention/comparator	Without intervention/comparator	-
2453	2014	The Effect of Neurodynamic Mobilization Exercise on Lower Limb Pain in Patients With Rheun	No	Without intervention/comparator	Without intervention/comparator	-
2454	2014	The Influence of Heart Rate Limitation on Exercise Tolerance in Pacemaker Patients	No	Without intervention/comparator	Without intervention/comparator	-
2455	2014	The PRESS RA: protein and Resistance Exercise Supplementation Study for Rheumatoid Arthri	No	Without intervention/comparator	Without intervention/comparator	-
2456	2014	Vitamin E-Diffused Highly Cross-Linked Polyethylene Liner	No	Without intervention/comparator	Without intervention/comparator	-
2457	2014	Wearable LITUS Device for Osteoarthritis of the Knee: a Randomized Double-Blind Placebo-Co	No	Without intervention/comparator	Without intervention/comparator	-
2458	2014	Yoga as an Intervention for Women With Knee Osteoarthritis	No	Without intervention/comparator	Without intervention/comparator	-
2459	2015	A Biomechanical Exercise Program for Knee OA	No	Without intervention/comparator	Without intervention/comparator	-
2460	2015	A Prospective Randomized Trial Comparing Standard Ligament Reconstruction Tendon Interpos	No	Without intervention/comparator	Without intervention/comparator	-
2461	2015	A Study Evaluating the Safety, Tolerability, and Efficacy of SM04690 Injected in the Target Kn	No	Without intervention/comparator	Without intervention/comparator	-

2462	2015	A Study of Siriraj Home Exercises Protocol for Primary Osteoarthritis of the Knee	No	Without intervention/comparator	Without intervention/comparator	-
2463	2015	A Study of the Safety and Effectiveness of Orally Administered CR845 in Patients With Osteoar	No	Without intervention/comparator	Without intervention/comparator	-
2464	2015	A Trial to Determine the Effects of Exercise in Inflammatory Bowel Disease and Rheumatoid Ar	No	Without intervention/comparator	Without intervention/comparator	-
2465	2015	Acupuncture in Distal Radius Fracture Patients	No	Other populations/diseases	Other populations/diseases	-
2466	2015	APOS Therapy for Osteoarthritis of the Knee: a Randomized Controlled Trial BIOTOK	No	Without intervention/comparator	Without intervention/comparator	-
2467	2015	Dry Needling Versus Conventional Physical Therapy in Patients With Knee Osteoarthritis	No	Without intervention/comparator	Without intervention/comparator	-
2468	2015	Effect of Periodontitis on Bone Mineral Density in Postmenopausal Women	No	Other populations/diseases	Other populations/diseases	-
2469	2015	Effectiveness of Manual Therapy and Exercise in Shoulder OA	No	Without intervention/comparator	Without intervention/comparator	-
2470	2015	Effectiveness of Pain Relief Between Adductor Canal Block and Femoral Nerve Block in Total K	No	Without intervention/comparator	Without intervention/comparator	-
2471	2015	Effectiveness of Trapeziometacarpal Splint	No	Without intervention/comparator	Without intervention/comparator	-
2472	2015	Effects of HMB Supplementation on Recovery Following ACL Surgery	No	Other populations/diseases	Other populations/diseases	-
2473	2015	Effects of Tele- or In-person Prehabilitation in Candidates Awaiting Total Hip or Knee Arthropla	No	Without intervention/comparator	Without intervention/comparator	-
2474	2015	Effects of Therapeutic Exercises in Elderly Women With Knee Osteoarthritis	No	Without intervention/comparator	Without intervention/comparator	-
2475	2015	Effects of Virtual Reality Rehabilitation in Patients With Total Knee Arthroplasty	No	Without intervention/comparator	Without intervention/comparator	-
2476	2015	Efficacy of Eccentric Resistance Training in Persons With Knee Osteoarthritis	No	Without intervention/comparator	Without intervention/comparator	-
2477	2015	Energy Dispersive Bracing for Conservative Treatment of Knee Osteoarthritis	No	Without intervention/comparator	Without intervention/comparator	-
2478	2015	Evaluation of rGH Therapy to Prevent Muscle Atrophy in Patients With ACL Tears	No	Other populations/diseases	Other populations/diseases	-
2479	2015	Evaluation of the Effect of Acupuncture on Hand Pain, Functional Deficits and Health Related Q	No	Without intervention/comparator	Without intervention/comparator	-
2480	2015	Evaluation of the Effectiveness of Self Management Program on Changes in Physical Activity Le	No	Without intervention/comparator	Without intervention/comparator	-
2481	2015	Exparel vs Exparel Plus ACB in TKAs	No	Without intervention/comparator	Without intervention/comparator	-
2482	2015	Exploring the Effectiveness of Sensor-based Balance Training on Patient Outcome Measures	No	Without intervention/comparator	Without intervention/comparator	-
2483	2015	Finnish Unicompartmental and Total Knee Arthroplasty Investigation	No	Without intervention/comparator	Without intervention/comparator	-
2484	2015	Home Rehabilitation in Patients After Primary Total Knee Arthroplasty	No	Without intervention/comparator	Without intervention/comparator	-
2485	2015	Hyaluronic Acid vs Platelet Rich Plasma: effects on Clinical Outcomes and Intra-articular Biolog	No	Without intervention/comparator	Without intervention/comparator	-
2486	2015	Improved Ability to Cope With Everyday Life Through a Person-centered Training Program in E	No	Without intervention/comparator	Without intervention/comparator	-
2487	2015	Influence of Using Physical Therapy Resources for Knee Osteoarthritis	No	Without intervention/comparator	Without intervention/comparator	-
2488	2015	Intra-Articular Injections of Platelet-Rich Plasma in Knee Osteoarthritis: unique Application Ver	No	Without intervention/comparator	Without intervention/comparator	-
2489	2015	Joint Loading vs Normal Physiotherapy Care in Degenerative Knees (V1)	No	Without intervention/comparator	Without intervention/comparator	-
2490	2015	Joint Replacement or Interpositional Arthroplasty for CMC1 Arthritis, a Prospective Trial	No	Without intervention/comparator	Without intervention/comparator	-
2491	2015	Manual Therapy and Dry Needling in Patellofemoral Syndrome	No	Without intervention/comparator	Without intervention/comparator	-
2492	2015	Microcurrent Stimulation Reduces Post-Operative Swelling and Healing Time Following Knee R	No	Without intervention/comparator	Without intervention/comparator	-
2493	2015	OPAM-IA: using Digital Activity Trackers to Improve Physical Activity in Inflammatory Arthrit	No	Without intervention/comparator	Without intervention/comparator	-
2494	2015	Overcoming TWEAK Signaling to Restore Muscle and Mobility After Joint Replacement	No	Without intervention/comparator	Without intervention/comparator	-
2495	2015	Pilot RCT Comparing Effectiveness of Two Decision Aids for Hip and Knee Osteoarthritis	No	Without intervention/comparator	Without intervention/comparator	-
2496	2015	Progressive Strength in Hand Osteoarthritis	No	Without intervention/comparator	Without intervention/comparator	-
2497	2015	PRP Therapy to m. Gluteus Medius During THA	No	Without intervention/comparator	Without intervention/comparator	-
2498	2015	Resilience for Older Workers With OA Through Exercise	No	Without intervention/comparator	Without intervention/comparator	-
2499	2015	Skeletal Muscle Dysfunction in Rheumatoid Arthritis (RA)	No	Without intervention/comparator	Without intervention/comparator	-
2500	2015	SuPRA: using Wearable Activity Trackers With a New Application to Improve Physical Activity	No	Without intervention/comparator	Without intervention/comparator	-
2501	2015	Swedish PAP in Osteoarthritis - a RCT Study	No	Without intervention/comparator	Without intervention/comparator	-
2502	2015	The Effect of a Telerehabilitaion Program on Gait and Balance in Patients After Hip Surgery	No	Without intervention/comparator	Without intervention/comparator	-
2503	2015	The Effect of AyuFlexÂ® Supplementation on Joint Health	No	Without intervention/comparator	Without intervention/comparator	-
2504	2015	the Evaluation of Four Non-operative Treatments for Degenerative Lumbar Spinal Stenosis	No	Without intervention/comparator	Without intervention/comparator	-
2505	2015	The KNEEhabilitation Study: improving Disability in Individuals With Knee Osteoarthritis	No	Without intervention/comparator	Without intervention/comparator	-

2506	2015	The PLEÂ²NO Self-management and Exercise Program for Knee Osteoarthritis	No	Without intervention/comparator	Without intervention/comparator	-
2507	2015	Using CERS to Optimize Quality of Life for Persons With Diabetes and Chronic Pain	No	Other populations/diseases	Other populations/diseases	-
2508	2015	Walk for Rheumatoid Arthritis (WARA Study)	No	Without intervention/comparator	Without intervention/comparator	-
2509	2015	Wearable Sensor-based Balance Training for Patients With Knee Osteoarthritis	No	Without intervention/comparator	Without intervention/comparator	-
2510	2015	Weight Loss and Exercise for Communities With Arthritis in North Carolina	No	Without intervention/comparator	Without intervention/comparator	-
2511	2015	Yoga Versus Exercises for Managing OA	No	Without intervention/comparator	Without intervention/comparator	-
2512	2016	Accute Effect of B-Turmactive Both on Mild and Moderate Knee Pain on Healthy Volunteers	No	Without intervention/comparator	Without intervention/comparator	-
2513	2016	Adapted Yoga for Inactive Older Adults	No	Without intervention/comparator	Without intervention/comparator	-
2514	2016	An Animated Home-based Physical Exercise Program as a Treatment Option for Patients With F	No	Without intervention/comparator	Without intervention/comparator	-
2515	2016	An Innovative Mind-motor Exercise Approach to Osteoarthritis Treatment	No	Without intervention/comparator	Without intervention/comparator	-
2516	2016	Blood Flow Restriction Exercise Study	No	Other populations/diseases	Other populations/diseases	-
2517	2016	Circuit Training on Knee Osteoarthritis Patients	No	Without intervention/comparator	Without intervention/comparator	-
2518	2016	Clinical Trial to Evaluate the Adjuvant Effect of Shock Wave Therapy in the Insertional Achilles	No	Without intervention/comparator	Without intervention/comparator	-
2519	2016	Collaborative Lifestyle Intervention Program in Knee Osteoarthritis Patients	No	Without intervention/comparator	Without intervention/comparator	-
2520	2016	Combined Application of Electrical Stimulated Antagonist Contraction During Walking (Walkin	No	Without intervention/comparator	Without intervention/comparator	-
2521	2016	Combined Application of Electrical Stimulation and Volitional Contractions for Muscle Strengtl	No	Without intervention/comparator	Without intervention/comparator	-
2522	2016	Comparative Study of Anterior Cruciate Ligament Reconstruction (Quadriceps Versus Hamstrin	No	Without intervention/comparator	Without intervention/comparator	-
2523	2016	Comparison Between Hemitrapeziectomy and Total Trapeziectomy With Ligament Reconstructi	No	Without intervention/comparator	Without intervention/comparator	-
2524	2016	Comparison Between Kinesiotaping and Cold Therapy After Total Knee Arthroplasty	No	Without intervention/comparator	Without intervention/comparator	-
2525	2016	Continuous Blockade of the Brachial Plexus	No	Without intervention/comparator	Without intervention/comparator	-
2526	2016	Continuous Versus Single-Shot Adductor Canal Block in Total Knee Arthroplasty	No	Without intervention/comparator	Without intervention/comparator	-
2527	2016	Danish Rct on Exercise Versus Arthroscopic Meniscal Surgery for Young Adults	No	Without intervention/comparator	Without intervention/comparator	-
2528	2016	Effect of High-intensity Interval Training on Metabolic Fitness in Overweight Males	No	Without intervention/comparator	Without intervention/comparator	-
2529	2016	Effect of Hip and Core Muscle Strengthening for Patellofemoral Osteoarthritis: a Feasibility Stuc	No	Without intervention/comparator	Without intervention/comparator	-
2530	2016	Effect of Immobilization of the Metacarpophalangeal Joint in Thumb Osteoarthritis	No	Without intervention/comparator	Without intervention/comparator	-
2531	2016	Effect of Strengthening the Hip Abductor in Patients With Knee Osteoarthritis: randomized Con	No	Without intervention/comparator	Without intervention/comparator	-
2532	2016	Effects of Specific Balance Training Prior TKR Surgery in the Early Postoperative Outcomes	No	Without intervention/comparator	Without intervention/comparator	-
2533	2016	Effects of Underwater Ultrasound Therapy on Hand Function and Quality of Life in Patients Wit	No	Without intervention/comparator	Without intervention/comparator	-
2534	2016	Efficacy of Proprioceptive Neuromuscular Facilitation in Older Women With Gonarthrosis	No	Without intervention/comparator	Without intervention/comparator	-
2535	2016	Evaluation of the Effectiveness of Night Orthosis in Treating Women With Symptomatic Osteoa	No	Without intervention/comparator	Without intervention/comparator	-
2536	2016	Evaluation of Two Types of PRP in Knee Osteoarthritis	No	Without intervention/comparator	Without intervention/comparator	-
2537	2016	Exercise and Mediterranean Diet on Body Composition, Disease Activity and Inflammatory Mar	No	Without intervention/comparator	Without intervention/comparator	-
2538	2016	Exercise Training in Patients With Rheumatoid Arthritis and Cryotherapy	No	Without intervention/comparator	Without intervention/comparator	-
2539	2016	Fit-Joint: getting Fit for Hip or Knee Replacement	No	Without intervention/comparator	Without intervention/comparator	-
2540	2016	Fixation of Patellar Tendon Grafts in Anterior Cruciate Ligament Reconstruction. Endobutton vs	No	Without intervention/comparator	Without intervention/comparator	-
2541	2016	Formal vs. Home-Based Physical Therapy After Unicompartmental Knee Arthroplasty	No	Without intervention/comparator	Without intervention/comparator	-
2542	2016	Functional Outcome and Analgesia in TKA: radiofrequency vs Continuous Adductor Canal Bloc	No	Without intervention/comparator	Without intervention/comparator	-
2543	2016	Home PT vs FORCE PT	No	Without intervention/comparator	Without intervention/comparator	-
2544	2016	Improvement in Pain and Function Following a Physiotherapy Program in Older Adults With Kn	No	Without intervention/comparator	Without intervention/comparator	-
2545	2016	Improving Rehabilitation Outcomes After Total Hip Arthroplasty	No	Without intervention/comparator	Without intervention/comparator	-
2546	2016	Influences of Balance Training With a Dynamometric Platform in Total Knee Arthroplasty	No	Without intervention/comparator	Without intervention/comparator	-
2547	2016	Interval Training Study in Psoriatic Arthritis	No	Other populations/diseases	Other populations/diseases	-
2548	2016	Kinematic Comparison of Vanguard XP and Vanguard CR Total Knee Arthroplasties	No	Without intervention/comparator	Without intervention/comparator	-
2549	2016	Knee Osteoarthritis Care: a Quality Improvement Intervention in General Practice	No	Without intervention/comparator	Without intervention/comparator	-

2550	2016	Manual Therapy for Hand Osteoarthritis	No	Without intervention/comparator	Without intervention/comparator	-
2551	2016	Outcomes Following Suction Drain and Non-suction Drain Assisted Total Knee Arthroplasty	No	Without intervention/comparator	Without intervention/comparator	-
2552	2016	P.A.R.Q.V.E III - Comparison of the Educational Program With and Without Multidisciplinary (	No	Without intervention/comparator	Without intervention/comparator	-
2553	2016	Patient Education and Basic Body Awareness Therapy in Hip Osteoarthritis: a Randomized Con	No	Without intervention/comparator	Without intervention/comparator	-
2554	2016	Patient-Centred Innovations for Persons With Multimorbidity - Ontario	No	Other populations/diseases	Other populations/diseases	-
2555	2016	Patient-Centred Innovations for Persons With Multimorbidity - Quebec	No	Other populations/diseases	Other populations/diseases	-
2556	2016	Prehabilitation Using Aquatic Exercise	No	Without intervention/comparator	Without intervention/comparator	-
2557	2016	Quadriceps Exercise Before Total Knee Arthroplasty (The QUADX-1 Trial)	No	Without intervention/comparator	Without intervention/comparator	-
2558	2016	STepped Exercise Program for Knee OsteoArthritis	No	Without intervention/comparator	Without intervention/comparator	-
2559	2016	The Effect of Functional Task Training Combined With Therapeutic Ultrasound on Adults With	No	Without intervention/comparator	Without intervention/comparator	-
2560	2016	The Effects of a Six-week Exercise Programme Undertaken by Women With Rheumatoid Arthri	No	Without intervention/comparator	Without intervention/comparator	-
2561	2016	The Impaction of Exercise Training on Bone Mineral Density in Patients After Total Knee Arthr	No	Without intervention/comparator	Without intervention/comparator	-
2562	2016	The Maintaining Musculoskeletal Health Study	No	Without intervention/comparator	Without intervention/comparator	-
2563	2016	Ultrasound-Guided Pulsed Radiofrequency In The Treatment Of Patients With Osteoarthritis Kn	No	Without intervention/comparator	Without intervention/comparator	-
2564	2016	WEB-Based Physiotherapy for People With Axial Spondyloarthritis	No	Without intervention/comparator	Without intervention/comparator	-
2565	2016	Whole Body Vibration on Running Biomechanics	No	Other populations/diseases	Other populations/diseases	-
2566	2017	2 Weekly Intra-articular Hyaluronan Knee Injections, Given 1 wk. Apart, of HYMOVIS Combin	No	Without intervention/comparator	Without intervention/comparator	-
2567	2017	3VM for Treatment of Chronic Osteoarthritis Knee Pain	No	Without intervention/comparator	Without intervention/comparator	-
2568	2017	A Physical Therapist Administered Physical Activity Intervention After Total Knee Replacement	No	Without intervention/comparator	Without intervention/comparator	-
2569	2017	Addition of Clonidine to Ropivacaine in Adductor Canal Block	No	Without intervention/comparator	Without intervention/comparator	-
2570	2017	Biomechanical Osteoarthritis Outcomes in Meniscectomy Patients	No	Without intervention/comparator	Without intervention/comparator	-
2571	2017	Blood Flow Restriction to Improve Muscle Strength After ACL Injury	No	Other populations/diseases	Other populations/diseases	-
2572	2017	Comparing Different Types of Physical Therapy for Treating People With a Meniscal Tear and (	No	Without intervention/comparator	Without intervention/comparator	-
2573	2017	Comparison Between Supervised Group Exercise and Home Exercise Program for Knee Osteoar	No	Without intervention/comparator	Without intervention/comparator	-
2574	2017	Cryotherapy Associated With Exercise in Pain Control and Physical Function in Individuals Wit	No	Without intervention/comparator	Without intervention/comparator	-
2575	2017	Digital Biofeedback System Versus Conventional Home-based Rehabilitation After Total Hip R	No	Without intervention/comparator	Without intervention/comparator	-
2576	2017	Digital Motivation to Decrease Inactive Behaviour in Patients With Knee Osteoarthritis	No	Without intervention/comparator	Without intervention/comparator	-
2577	2017	Economic and Functional Impact of Peri-Operative Bracing for Primary Total Knee Arthroplasty	No	Without intervention/comparator	Without intervention/comparator	-
2578	2017	Effect of Flamingo Exercises on Balance	No	Without intervention/comparator	Without intervention/comparator	-
2579	2017	Effectiveness of Reduced Frequency Physical Therapy in Total Knee Arthroplasty	No	Without intervention/comparator	Without intervention/comparator	-
2580	2017	Effects of Action Observation Therapy on Pain, Functional Level and Brain Hemodynamic in Pa	No	Without intervention/comparator	Without intervention/comparator	-
2581	2017	Effects of Dry Needling Technique in Hip Muscles in Subjects With Grade I-III Hip Osteoarthrit	No	Without intervention/comparator	Without intervention/comparator	-
2582	2017	Effects of Ginger on Muscle Soreness and Dysfunction Stemming From Downhill Running	No	Without intervention/comparator	Without intervention/comparator	-
2583	2017	Effects of Video Game on Patients With Knee Osteoarthritis	No	Without intervention/comparator	Without intervention/comparator	-
2584	2017	Efficacy of an Intra-articular Injection of Botulinum Toxin A Associated With Splinting for Bas	No	Without intervention/comparator	Without intervention/comparator	-
2585	2017	Epidural Analgesia vs Adductor Canal Block in Bilateral TKA	No	Without intervention/comparator	Without intervention/comparator	-
2586	2017	ESCAPE-pain Programme in Malaysia	No	Without intervention/comparator	Without intervention/comparator	-
2587	2017	Evaluation of a Web-based Platform for Osteoarthritis Treatment	No	Without intervention/comparator	Without intervention/comparator	-
2588	2017	Evaluation of Methods of Teaching Self-management Strategies to Patients With Symptomatic K	No	Without intervention/comparator	Without intervention/comparator	-
2589	2017	EXercise as TReatment for osteoArthritis	No	Without intervention/comparator	Without intervention/comparator	-
2590	2017	Exercises in the Post-operative Rehabilitation of THA	No	Without intervention/comparator	Without intervention/comparator	-
2591	2017	Extracorporeal Shockwave Therapy for Knee Osteoarthritis	No	Without intervention/comparator	Without intervention/comparator	-
2592	2017	Improving Resilience and Longevity for Workers Through Exercise	No	Without intervention/comparator	Without intervention/comparator	-
2593	2017	In-home Versus Hospital Preoperative Training for Patients Undergoing Total Knee Replacemen	No	Without intervention/comparator	Without intervention/comparator	-

2594	2017	Is Fluidotherapy Effective in Rheumatoid Hand?	No	Without intervention/comparator	Without intervention/comparator	-
2595	2017	Isometric Versus Isotonic Exercise for Greater Trochanteric Pain Syndrome	No	Without intervention/comparator	Without intervention/comparator	-
2596	2017	Laser Therapy, Pain and Carpometacarpal Joint Osteoarthritis Treatment	No	Without intervention/comparator	Without intervention/comparator	-
2597	2017	Lessening the Impact of Fatigue in Inflammatory Rheumatic Diseases	No	Without intervention/comparator	Without intervention/comparator	-
2598	2017	Microcurrent Dressing to Treat Infections, Before, During and After Surgery	No	Without intervention/comparator	Without intervention/comparator	-
2599	2017	Novel Pre-Surgery Exercise-Conditioning in Patients Waiting for Total Knee Arthroplasty (TKA	No	Without intervention/comparator	Without intervention/comparator	-
2600	2017	Post-operative Rehabilitation of Total Knee Arthroplasty With Applications on Smart Phone	No	Without intervention/comparator	Without intervention/comparator	-
2601	2017	Postoperative Intervention Program Effectiveness in Hip Fracture Patients: a Randomized Clinic	No	Without intervention/comparator	Without intervention/comparator	-
2602	2017	Saline Lavage X Saline Lavage and OsteonilÂ® Mini in Rizarthritis	No	Other populations/diseases	Other populations/diseases	-
2603	2017	Saphenous Nerve Block Versus Platelet Rich Plasma for Chronic Knee Osteoarthritis	No	Without intervention/comparator	Without intervention/comparator	-
2604	2017	Selecting the Right Hip Prosthesis for Young Patients	No	Without intervention/comparator	Without intervention/comparator	-
2605	2017	Skeletal Muscle Atrophy and Dysfunction Following Total Knee Arthroplasty	No	Without intervention/comparator	Without intervention/comparator	-
2606	2017	Sleep and Exercise in Rheumatoid Arthritis	No	Without intervention/comparator	Without intervention/comparator	-
2607	2017	Star Excursion Balance for Patellofemoral Pain Syndrome	No	Without intervention/comparator	Without intervention/comparator	-
2608	2017	Task Shifting in the Care for Patients With Hand Osteoarthritis	No	Without intervention/comparator	Without intervention/comparator	-
2609	2017	The Effect of AposTherapy on Knee Pain	No	Without intervention/comparator	Without intervention/comparator	-
2610	2017	The Effects of Two Different Orthosis on Pain, Hand Strength and Function in Patients With Th	No	Without intervention/comparator	Without intervention/comparator	-
2611	2017	The Marigot Osteoarthritis Nutritional Intervention (MOANi) Trial	No	Without intervention/comparator	Without intervention/comparator	-
2612	2017	The Study of Neuromuscular Exercise Therapy and Patients Self-management Program in Knee	No	Without intervention/comparator	Without intervention/comparator	-
2613	2017	Traditional Versus Alternative Alignment in TKR	No	Without intervention/comparator	Without intervention/comparator	-
2614	2017	Trial Evaluating multimOdal toPical Cream In CompArison to pLacebo (TOPICAL)	No	Without intervention/comparator	Without intervention/comparator	-
2615	2017	Using Mobilization Exercises on Total Knee Arthroplasty Rehabilitation	No	Without intervention/comparator	Without intervention/comparator	-
2616	2018	A Prospective Multicenter Longitudinal Cohort Study of the Mymobility Platform	No	Without intervention/comparator	Without intervention/comparator	-
2617	2018	A Randomized Control Trial: returning to Run After Injury	No	Without intervention/comparator	Without intervention/comparator	-
2618	2018	ACL Rehabilitation	No	Without intervention/comparator	Without intervention/comparator	-
2619	2018	An Intervention Study Evaluating the Effects of a Raspberry Leaf Extract in an Osteoarthritic Po	No	Without intervention/comparator	Without intervention/comparator	-
2620	2018	an On-demand Program to EmpoweR Active Self-management (OPERAS)	No	Without intervention/comparator	Without intervention/comparator	-
2621	2018	Are Women With Higher Professional Status More Sedentary Compared to Men?	No	Without intervention/comparator	Without intervention/comparator	-
2622	2018	Assessment of HydroxyColl Bone Graft Substitute in High Tibial Osteotomy Wedge Grafting	No	Without intervention/comparator	Without intervention/comparator	-
2623	2018	Better Before - Better After: prehabilitation Program for Older Patients Awaiting Total Hip Repl	No	Without intervention/comparator	Without intervention/comparator	-
2624	2018	Biofeedback With Cycling Exercise in OA Knee Patients	No	Without intervention/comparator	Without intervention/comparator	-
2625	2018	Cartilage Adaptation and Response to Interleukins and Exercise	No	Without intervention/comparator	Without intervention/comparator	-
2626	2018	Case Series Evaluation of Psychodynamic Interpersonal Therapy in Chronic Low Back Pain	No	Without intervention/comparator	Without intervention/comparator	-
2627	2018	CBS Treatment in Hand Osteoarthritis and Psoriatic Arthritis	No	Other populations/diseases	Other populations/diseases	-
2628	2018	Ceramic-on-Ceramic Versus Ceramic-on-HXLPE THA	No	Without intervention/comparator	Without intervention/comparator	-
2629	2018	Cognitive Behavioural Therapy for Insomnia on Sleep in Rheumatoid Arthritis	No	Without intervention/comparator	Without intervention/comparator	-
2630	2018	Comparison of an Old Versus a New Total Knee Replacement	No	Without intervention/comparator	Without intervention/comparator	-
2631	2018	Comparison of the Accuracy in Rehabilitation Exercise Between Mobile Application Actuated R	No	Without intervention/comparator	Without intervention/comparator	-
2632	2018	Cryoneurolysis for the Management of Chronic Pain in Patients With Knee Osteoarthritis	No	Without intervention/comparator	Without intervention/comparator	-
2633	2018	Desflurane-based Enhanced Recovery After Surgery (D-ERAS) Pathway for Primary Hip and Kı	No	Without intervention/comparator	Without intervention/comparator	-
2634	2018	Developing an Online Therapeutic Intervention for Chronic Pain in Veterans	No	Without intervention/comparator	Without intervention/comparator	-
2635	2018	Does Rehabilitation After Total Hip and Knee Arthroplasty Work	No	Without intervention/comparator	Without intervention/comparator	-
2636	2018	Educational Program Associated With a Conventional Spa Therapy of Knee Osteoarthritis Patier	No	Without intervention/comparator	Without intervention/comparator	-
2637	2018	Effect of Chosen Treatment Methods in Patients With Cervical Spine Osteoarthritis	No	Without intervention/comparator	Without intervention/comparator	-

2638	2018	Effect of Kinesio Taping in Women With Knee Osteoarthritis	No	Without intervention/comparator	Without intervention/comparator	-
2639	2018	Effect of Kinesotaping on Management of Supraspinatus Tendinitis	No	Without intervention/comparator	Without intervention/comparator	-
2640	2018	Effect of Tai Chi Exercise on Mechanical Joint Loading in Knee Osteoarthritis	No	Without intervention/comparator	Without intervention/comparator	-
2641	2018	Effect of Tai Chi on Functional Fitness of Elderly Patients With Degenerative Arthritis	No	Without intervention/comparator	Without intervention/comparator	-
2642	2018	Effects of Internet / Web-based Exercises on the Population With Knee Arthritis	No	Without intervention/comparator	Without intervention/comparator	-
2643	2018	Effects of Motor Imagery Intervention on Functional Recovery Following Total Knee Arthroplas	No	Without intervention/comparator	Without intervention/comparator	-
2644	2018	Effects of Web-based Exercises on the Population With Knee Arthritis	No	Without intervention/comparator	Without intervention/comparator	-
2645	2018	Enhanced Recovery After Surgery (ERAS) Pathway for Primary Hip and Knee Arthroplasty	No	Without intervention/comparator	Without intervention/comparator	-
2646	2018	Evaluation of the Effect of Rehabilitation Sport After Total Hip Arthroplasty (THA)	No	Without intervention/comparator	Without intervention/comparator	-
2647	2018	Exercise Therapy in Combination With Central Nervous System-targeted Treatment for Osteoart	No	Without intervention/comparator	Without intervention/comparator	-
2648	2018	Gamification in Knee Replacement Rehabilitation	No	Without intervention/comparator	Without intervention/comparator	-
2649	2018	Healing Osteoarthritic Joints in the Wrist With Adult ADRCs	No	Without intervention/comparator	Without intervention/comparator	-
2650	2018	Home Exercise vs PT for Reverse Total Shoulder Arthroplasty	No	Without intervention/comparator	Without intervention/comparator	-
2651	2018	Inflammatory Targeted Laser Treatment of Knee Osteoarthritis	No	Without intervention/comparator	Without intervention/comparator	-
2652	2018	Intraoperative Direct vs Postoperative Ultrasound Guided Adductor Canal Nerve Block After To	No	Without intervention/comparator	Without intervention/comparator	-
2653	2018	Investigating the Effect of Deep Sea Krill Oil Supplementation in Osteoarthritis of the Knee	No	Without intervention/comparator	Without intervention/comparator	-
2654	2018	Is Pulsed Ultrasound Treatment Effective in Knee Osteoarthritis	No	Without intervention/comparator	Without intervention/comparator	-
2655	2018	Knee Brace and Biomechanical Footwear in the Treatment of Knee Osteoarthritis	No	Without intervention/comparator	Without intervention/comparator	-
2656	2018	Load Modification Versus Standard Exercise for Greater Trochanteric Pain Syndrome	No	Without intervention/comparator	Without intervention/comparator	-
2657	2018	Lower Knee Joint Loading by Real-time Biofeedback Stair Walking Rehabilitation for Patients V	No	Without intervention/comparator	Without intervention/comparator	-
2658	2018	Mobile Technology to Support Physical Therapy Exercise	No	Without intervention/comparator	Without intervention/comparator	-
2659	2018	Motor Imagery to Facilitate Sensorimotor Relearning After ACL Injury	No	Other populations/diseases	Other populations/diseases	-
2660	2018	Neck Exercises in Patients With Temporomandibular Disorders	No	Other populations/diseases	Other populations/diseases	-
2661	2018	Non-invasive Vagus Nerve Stimulation (nVNS) in Pediatric Chronic Inflammatory Demyelinatii	No	Without intervention/comparator	Without intervention/comparator	-
2662	2018	Non-Surgical Management of Knee Osteoarthritis in the Military Health System (MHS)	No	Without intervention/comparator	Without intervention/comparator	-
2663	2018	Online Mindfulness Program for Stress Management	No	Other populations/diseases	Other populations/diseases	-
2664	2018	RCT of a Temporomandibular Joint Distraction Device for Patients With Articular Disorders Di	No	Without intervention/comparator	Without intervention/comparator	-
2665	2018	Reverse Shoulder Replacement: formal vs. Home Physiotherapy	No	Without intervention/comparator	Without intervention/comparator	-
2666	2018	RF TKA Prehabilitation	No	Without intervention/comparator	Without intervention/comparator	-
2667	2018	Shoulder Instability Trial Comparing Arthroscopic Stabilization Benefits Compared With Latarj	No	Without intervention/comparator	Without intervention/comparator	-
2668	2018	STaR Trial: multiple Ligament Knee Injuries	No	Without intervention/comparator	Without intervention/comparator	-
2669	2018	Tai Chi in Rheumatoid Arthritis (TaiChiRA)	No	Without intervention/comparator	Without intervention/comparator	-
2670	2018	The Benefit of Functional Movement Control for Patients With Knee Osteoarthritis	No	Without intervention/comparator	Without intervention/comparator	-
2671	2018	The Effects of a Tourniquet in Total Knee Arthroplasty	No	Without intervention/comparator	Without intervention/comparator	-
2672	2018	The Effects of Neuromuscular Exercises Training on Physical Activity, Functionality and Balanc	No	Without intervention/comparator	Without intervention/comparator	-
2673	2018	The Efficacy of Exercise and Alternative Applications of NMES on Pain and Function in Patient	No	Without intervention/comparator	Without intervention/comparator	-
2674	2018	The Efficacy of TGF for Treating Osteoarthritis of the Knee	No	Without intervention/comparator	Without intervention/comparator	-
2675	2018	Treatment of Knee Osteoarthrosis Using the Electromagnetic Fields	No	Without intervention/comparator	Without intervention/comparator	-
2676	2018	Ultrasound and Exercises in Knee Osteoarthritis	No	Without intervention/comparator	Without intervention/comparator	-
2677	2018	Variable Resistance Training in Patients With Osteoarthrosis of the Knee	No	Without intervention/comparator	Without intervention/comparator	-
2678	2018	Yoga for Patients With Rheumatoid Arthritis	No	Without intervention/comparator	Without intervention/comparator	-
2679	2019	3 Local Anesthetics for Spinal Anesthesia in Primary Total Hip Arthroplasty	No	Without intervention/comparator	Without intervention/comparator	-
2680	2019	6 Weeks Step Aerobics Training Among Females With Sedentary Life Style	No	Without intervention/comparator	Without intervention/comparator	-
2681	2019	A Pragmatic Trial To Determine the Benefit of Exercise Incentives and Corticosteroid Injections	No	Without intervention/comparator	Without intervention/comparator	-

2682	2019	A Tablet-based Yesple Walking Intervention	No	Without intervention/comparator	Without intervention/comparator	-
2683	2019	Adductor Canal Block in an Enhanced Recovery Program After Total Knee Arthroplasty	No	Without intervention/comparator	Without intervention/comparator	-
2684	2019	Anterior Cruciate Ligament Reconstruction +/- Lateral Tenodesis With Patellar vs Quad Tendon	No	Without intervention/comparator	Without intervention/comparator	-
2685	2019	Antibiotic Loaded Bone Cement in Prevention of Periprosthetic Joint Infections in Primary Tota	No	Without intervention/comparator	Without intervention/comparator	-
2686	2019	Assessing if Cryoneurolysis Improves Prehabilitation and Decreases Pain After Surgery With Le	No	Without intervention/comparator	Without intervention/comparator	-
2687	2019	Balance Training Using Biodex Stability System in Knee Osteoarthritis	No	Without intervention/comparator	Without intervention/comparator	-
2688	2019	Blood Flow Restriction Following Hip Arthroscopy	No	Other populations/diseases	Other populations/diseases	-
2689	2019	Care of AcromioClavicular Arthropathy in Manual Medicine Versus Corticosteroid Infiltration (	No	Without intervention/comparator	Without intervention/comparator	-
2690	2019	Chinese Massage for Treatment of KOA	No	Without intervention/comparator	Without intervention/comparator	-
2691	2019	Collaborative Model of Care Between Orthopaedics and Allied Healthcare Professionals Trial (C	No	Without intervention/comparator	Without intervention/comparator	-
2692	2019	Comparison of Stemless and Stemmed Shoulder Arthroplasty for Osteoarthritis	No	Without intervention/comparator	Without intervention/comparator	-
2693	2019	Comparison of The Effects of Concentric And Eccentric Isokinetic Exercises in Patients With K	No	Without intervention/comparator	Without intervention/comparator	-
2694	2019	Decrease in Blood Pressure in PARQVE	No	Without intervention/comparator	Without intervention/comparator	-
2695	2019	Determination of the Effectiveness of Certain Physical Methods in the Treatment of Knee Osteo	No	Without intervention/comparator	Without intervention/comparator	-
2696	2019	Digital Patient Journey Solution for Patients Undergoing Elective Hip and Knee Arthroplasty Du	No	Without intervention/comparator	Without intervention/comparator	-
2697	2019	Distraction vs Interposition Arthroplasty for Basilar Thumb Osteoarthritis	No	Without intervention/comparator	Without intervention/comparator	-
2698	2019	Effect of Energy Drink on Sport Performance and Psycho-Physiological Responses	No	Without intervention/comparator	Without intervention/comparator	-
2699	2019	EFFECT OF QUINOA AND FLAXSEED IN CITOTOXICITY AND GLICEMYC CONTROL I	No	Other populations/diseases	Other populations/diseases	-
2700	2019	Effectiveness of a Mobile App for Individuals With or at Risk of Knee Osteoarthritis	No	Without intervention/comparator	Without intervention/comparator	-
2701	2019	Effectiveness of Curcumin-based Food Supplement in Reducing Pain and Inflammatory Compo	No	Without intervention/comparator	Without intervention/comparator	-
2702	2019	Effects of Action Observation Therapy on Pain and Brain Hemodynamics in Patients With Knee	No	Without intervention/comparator	Without intervention/comparator	-
2703	2019	Effects of Body Awareness Therapy in Knee Osteoarthritis	No	Without intervention/comparator	Without intervention/comparator	-
2704	2019	Effects of Clinical Pilates Exercises in Patients With Rheumatoid Arthritis	No	Without intervention/comparator	Without intervention/comparator	-
2705	2019	Effects of Closed and Open Kinetic Chain Exercises	No	Without intervention/comparator	Without intervention/comparator	-
2706	2019	Effects of Diet Control and Resistance Exercise Training on Obesity Adults With Knee Osteoart	No	Without intervention/comparator	Without intervention/comparator	-
2707	2019	Effects of Dry Needling on Muscle Function in Patients With Hip Osteoarthritis	No	Without intervention/comparator	Without intervention/comparator	-
2708	2019	Effects of Early Home-based Strength and Sensory-motor Training After THA on Functional Ou	No	Without intervention/comparator	Without intervention/comparator	-
2709	2019	Effects of Exercise and Education in Patients With Chronic Pain After Total Knee Replacement	No	Without intervention/comparator	Without intervention/comparator	-
2710	2019	Effects of Improved Calf Muscle Function on Gait, Balance and Joint Loading in Older Adults	No	Without intervention/comparator	Without intervention/comparator	-
2711	2019	Effects of Sulfur Water and Mud Therapy on Serotonin Activity and Biochemical Parameters in	No	Without intervention/comparator	Without intervention/comparator	-
2712	2019	Effects of Two Different Dry-Needling Techniques for Low Back Pain	No	Without intervention/comparator	Without intervention/comparator	-
2713	2019	Efficacy of an Exercise Program for Patients With Femoro-acetabular Impingement	No	Without intervention/comparator	Without intervention/comparator	-
2714	2019	Efficacy of Magnetotherapy in Hand Erosive Osteoarthritis	No	Without intervention/comparator	Without intervention/comparator	-
2715	2019	Elastic Band Resistance Exercise on Glycated Haemoglobin and Muscle Strength, Balance, and	No	Without intervention/comparator	Without intervention/comparator	-
2716	2019	Electromyographic Biofeedback on Performance of Vastus Medialis Oblique Muscle in Knee Os	No	Without intervention/comparator	Without intervention/comparator	-
2717	2019	Evaluation of NMES for Reducing Pain and Improving Functional Outcomes in Knee OA Patien	No	Without intervention/comparator	Without intervention/comparator	-
2718	2019	Evaluation of Safety and Efficacy of ReHub in Patients Who Underwent Primary TKA (REHAP	No	Without intervention/comparator	Without intervention/comparator	-
2719	2019	Footwear and Exercise for Knee Osteoarthritis (FiREwORK Trial)	No	Without intervention/comparator	Without intervention/comparator	-
2720	2019	Gait Retraining Enhances Athletes' Technique	No	Without intervention/comparator	Without intervention/comparator	-
2721	2019	Immediate Effects Of Alliance Therapy In Modulation Of Pain And Disability In Subjects With	No	Without intervention/comparator	Without intervention/comparator	-
2722	2019	Impact of Volunteerism in the Acute Setting	No	Without intervention/comparator	Without intervention/comparator	-
2723	2019	Improving Physical Activity and Gait Symmetry After Total Knee Arthroplasty	No	Without intervention/comparator	Without intervention/comparator	-
2724	2019	Independent Weight Loss Maintenance for Communities With Arthritis in North Carolina: the I-	No	Without intervention/comparator	Without intervention/comparator	-
2725	2019	Individualized Comprehensive Rehabilitation Program in Chronic Knee Osteoarthritis	No	Without intervention/comparator	Without intervention/comparator	-

2726	2019	Influence of Single Session of Aerobic Exercise on Acute Pain and Function in Patients After To	No	Without intervention/comparator	Without intervention/comparator	-
2727	2019	Intramedullary Nailing vs External Ring Fixation for the Treatment of Tibial Shaft Fractures	No	Other populations/diseases	Other populations/diseases	-
2728	2019	Investigating the Effectiveness of Vibration Therapy on Sarcopenia in Osteoarthritis Knee Patier	No	Without intervention/comparator	Without intervention/comparator	-
2729	2019	Knee Biofeedback Rehabilitation Interface for Game-based Home Therapy for Patients With Kn	No	Without intervention/comparator	Without intervention/comparator	-
2730	2019	Knee Osteoarthritis: platelet Rich Plasma or Hyaluronic Acid	No	Without intervention/comparator	Without intervention/comparator	-
2731	2019	Laser + Cryo-thermal Therapy Following Total Knee Replacement Surgery	No	Without intervention/comparator	Without intervention/comparator	-
2732	2019	LRTI vs Internal Brace for CMC OA	No	Without intervention/comparator	Without intervention/comparator	-
2733	2019	Mulligan Manual Therapy and Trunk Stabilization Exercises Versus Isometric Knee Strengtheni	No	Without intervention/comparator	Without intervention/comparator	-
2734	2019	Multi-modal Exercise Program in Older Aduls With Knee Osteoarthritis	No	Without intervention/comparator	Without intervention/comparator	-
2735	2019	Neuro-muscular Exercise Training Verses Quadriceps Training in Mild to Moderate Knee Osteo	No	Without intervention/comparator	Without intervention/comparator	-
2736	2019	Norwegian Distal Ulna Resection - Replacement Study	No	Without intervention/comparator	Without intervention/comparator	-
2737	2019	Pain & Aging: combined Interventions for Fitness in the Community Study	No	Without intervention/comparator	Without intervention/comparator	-
2738	2019	PARQVE Prior to Total Knee Replacement	No	Without intervention/comparator	Without intervention/comparator	-
2739	2019	Physical Therapy Following Total Hip Arthroplasty	No	Without intervention/comparator	Without intervention/comparator	-
2740	2019	Physiotherapy and Psychological Among Refugees From Syria	No	Other populations/diseases	Other populations/diseases	-
2741	2019	Portuguese Fit & Strong! for Older Adults With Osteoarthritis	No	Without intervention/comparator	Without intervention/comparator	-
2742	2019	Progressive Resistance Training Versus Total Hip Arthroplasty in Patients With Hip Osteoarthrit	No	Without intervention/comparator	Without intervention/comparator	-
2743	2019	Project Arthritis Recovering Quality of Life Through Education 70+	No	Without intervention/comparator	Without intervention/comparator	-
2744	2019	Radial Versus Focused Extracorporeal Shock Wave in the Treatment of Knee Osteoarthritis	No	Without intervention/comparator	Without intervention/comparator	-
2745	2019	RCT of Vibration Effect on Vertebral BMD in Disabled Patients	No	Without intervention/comparator	Without intervention/comparator	-
2746	2019	Shoulder Mobilization Following Supra Scapular Nerve Block in Adhesive Capsulitis	No	Without intervention/comparator	Without intervention/comparator	-
2747	2019	Strengthening Exercises Versus Proprioception and Balance Exercises in the Treatment of Knee	No	Without intervention/comparator	Without intervention/comparator	-
2748	2019	Strengthening Program for Foot-ankle Muscles in People With Knee Osteoarthritis	No	Without intervention/comparator	Without intervention/comparator	-
2749	2019	Study to Evaluate the Efficacy and Safety of FX006 in Patients With Glenohumeral Osteoarthriti	No	Without intervention/comparator	Without intervention/comparator	-
2750	2019	The Effect of Exercise Training on Balance and Functional Status in Individuals With Osteoarth	No	Without intervention/comparator	Without intervention/comparator	-
2751	2019	The Effect Of Pulsed Electromagnetic Field And Progressive Resistance Exercise On Knee Osteo	No	Without intervention/comparator	Without intervention/comparator	-
2752	2019	The Effect of Using Smart Phone Application for Enhancing Adherence to Home Exercise	No	Without intervention/comparator	Without intervention/comparator	-
2753	2019	The Effectiveness of Progressive Relaxation Exercises	No	Without intervention/comparator	Without intervention/comparator	-
2754	2019	The Effectiveness of Virtual Reality Based Rehabilitation in Patients With Knee Osteoarthritis	No	Without intervention/comparator	Without intervention/comparator	-
2755	2019	The Efficacy of Low-load Blood Flow Restricted Resistance Before TKR	No	Other populations/diseases	Other populations/diseases	-
2756	2019	The Use of Intra-articular Corticosteroid Injection to Treat Osteoarthritis of the Carpometacarpal	No	Without intervention/comparator	Without intervention/comparator	-
2757	2019	The Whole Body Vibration Training for Total Knee arthroplasty-the Improvement of the Lower I	No	Without intervention/comparator	Without intervention/comparator	-
2758	2019	To Determine the Gait and Functional Improvement in Total Knee Arthroplasty	No	Without intervention/comparator	Without intervention/comparator	-
2759	2019	To Evaluate the Optimum Dose of Platelet Rich Plasma in Knee Osteoarthritis and Compare Eff	No	Without intervention/comparator	Without intervention/comparator	-
2760	2019	Ultrasound-Guided Adductor Canal Block for Total Knee Arthroplasty Surgery	No	Without intervention/comparator	Without intervention/comparator	-
2761	2019	Uncemented Tritanium TKR vs Cemented Triathlon TKR	No	Without intervention/comparator	Without intervention/comparator	-
2762	2019	Usability and Effectiveness of ReHub in Patients After Total Hip Arthroplasty	No	Without intervention/comparator	Without intervention/comparator	-
2763	2019	Virtual Environment Rehabilitation for Patients With Motor Neglect Trial	No	Other populations/diseases	Other populations/diseases	-
2764	2019	Vitamin D Supplementation in Knee Osteoarthritis	No	Without intervention/comparator	Without intervention/comparator	-
2765	2019	Whole-body Vibration Training on Functional Performance of the Elderly With Knee Osteoarthr	No	Without intervention/comparator	Without intervention/comparator	-
2766	2020	Azithromycin Added to Hydrochloroquine in Patients Admitted to Intensive Care With COVID-	No	Other populations/diseases	Other populations/diseases	-
2767	2020	BFRT vs Standard PT After Total Knee Arthroplasty	No	Other populations/diseases	Other populations/diseases	-
2768	2020	Biofreeze and the Effect on Knee Osteoarthritis	No	Without intervention/comparator	Without intervention/comparator	-
2769	2020	Central Sensitization in Knee Osteoarthritis	No	Without intervention/comparator	Without intervention/comparator	-

2770	2020	Clinical Study of Intra Articular Injection of Catholic MASTER Cell (Bone Marrow Derived Me	No	Without intervention/comparator	Without intervention/comparator	-
2771	2020	Comparison of Training Load With/Out Blood Flow Restriction Training in Rheumatoid Popula	No	Other populations/diseases(Myositis,RA	Other populations/diseases(Myositis,RA,	-
2772	2020	Determining the Effectiveness of a New Phototherapy Treatment for the Knee Osteoarthritis	No	Without intervention/comparator	Without intervention/comparator	-
2773	2020	Digital Home-Exercise Therapy Application For Patients With Non-Surgical Knee Injuries	No	Without intervention/comparator	Without intervention/comparator	-
2774	2020	Effectiveness of an Exercise Program and Education Through a Mobile Application for the Man	No	Without intervention/comparator	Without intervention/comparator	-
2775	2020	Effects of Anti-gravity Treadmill Training on Knee Osteoarthritis in Geriatric Population	No	Without intervention/comparator	Without intervention/comparator	-
2776	2020	Effects of Creatine and Glucoseamine/ Chondritin Sulfate Co-Supplementation in Addition to E:	No	Without intervention/comparator	Without intervention/comparator	-
2777	2020	Effects of Creatine Supplementation in Addition to Resistance Exercise Training in Patients Wit	No	Without intervention/comparator	Without intervention/comparator	-
2778	2020	Electroacupuncture vs Topical Diclofenac Sodium Gel for Patients With Hand Osteoarthritis	No	Without intervention/comparator	Without intervention/comparator	-
2779	2020	Evaluation of the Effectiveness of a Muscular Strengthening Protocol With an Instrumented Ortl	No	Without intervention/comparator	Without intervention/comparator	-
2780	2020	Exercise Dosages and Exercise Adherence With Patients With Knee Osteoarthritis	No	Without intervention/comparator	Without intervention/comparator	-
2781	2020	Exercise Therapy for Osteoarthritis Pain: how Does it Work?	No	Without intervention/comparator	Without intervention/comparator	-
**2782**	**2020**	**Incorporation of Photobiomodulation Therapy in an Exercise Program With Blood Flow I**	**Yes**	**Full**	**Full**	**-**
2783	2020	Investigation of the Effects of Physiotherapy in Patients With Platelet Rich Plasma (PRP) Applie	No	Without intervention/comparator	Without intervention/comparator	-
2784	2020	Knee Arthritis Treatment With Autologous Fragmented Adipose Tissue and PRP - Comparison c	No	Without intervention/comparator	Without intervention/comparator	-
2785	2020	Motor Unit Abnormalities After Experimentally Induced Sensitization	No	Without intervention/comparator	Without intervention/comparator	-
2786	2020	MySlainte: testing the Effect of Involving Partners in a CVD Prevention Community Lifestyle Pı	No	Without intervention/comparator	Without intervention/comparator	-
2787	2020	Orthotic Management of CMC Osteoarthritis	No	Without intervention/comparator	Without intervention/comparator	-
2788	2020	Osteoarthritis Running & Cartilage Assessment	No	Without intervention/comparator	Without intervention/comparator	-
2789	2020	Peanut Protein Supplementation to Prevent Muscle Atrophy and Improve Recovery Following T	No	Without intervention/comparator	Without intervention/comparator	-
2790	2020	Preventing Knee Osteoarthritis Through Exercise and Education Following Knee Injury	No	Without intervention/comparator	Without intervention/comparator	-
2791	2020	Rate of Torque Development and Voluntary Quadriceps Activation in Patients With Knee Osteo	No	Without intervention/comparator	Without intervention/comparator	-
2792	2020	Reduce Pain and Improve Quality of Life in Patient With Knee Osteoarthritis by Light, Sound ar	No	Without intervention/comparator	Without intervention/comparator	-
2793	2020	Safety and Efficacy of JointAliveâ¢ on the Knee-joint Function in Adults With Knee Arthritis	No	Without intervention/comparator	Without intervention/comparator	-
2794	2020	Stimulate Brain and Reduce Knee Pain Due to Degeneration	No	Without intervention/comparator	Without intervention/comparator	-
2795	2020	Study Assessing Vagus Nerve Stimulation in CoViD-19 Respiratory Symptoms	No	Other populations/diseases	Other populations/diseases	-
2796	2020	The Effect of Zoledronic Acid on Patients With Osteoarthritis of the Hip	No	Without intervention/comparator	Without intervention/comparator	-
2797	2020	The Effects Of Jaw Sensorimotor System In The Treatment Of Neck Pain And Dysfunction	No	Without intervention/comparator	Without intervention/comparator	-
2798	2020	The Impact of Therapeutic Alliance in the Rehabilitation of Knee Osteoarthritis	No	Without intervention/comparator	Without intervention/comparator	-
2799	2020	The Use of Cannabinoid Patch for Knee Osteoarthritis	No	Without intervention/comparator	Without intervention/comparator	-
2800	2020	Transcutaneous Vagal Stimulation in Knee Osteoarthritis (TRAVKO)	No	Without intervention/comparator	Without intervention/comparator	-
2801	2020	Treatment Using 448 kHz CRMRF in Subacromial Syndrome	No	Without intervention/comparator	Without intervention/comparator	-
2802	2020	Use of the Orthoglide for Improved Patient Outcome Following Total Knee Replacement	No	Without intervention/comparator	Without intervention/comparator	-
2803	2020	Weight Loss and Exercise To Improve Rheumatoid Arthritis Cardiovascular Risk	No	Without intervention/comparator	Without intervention/comparator	-
2804	2018	Influence of isometric exercise on pressure pain sensitivity in knee osteoarthritis	No	Without intervention/comparator	Without intervention/comparator	-
2805	2017	A clinical study to evaluate the efficacy of Hingwadi Churna and Rasnadashmula Kwatha in Am	No	Without intervention/comparator	Without intervention/comparator	-
2806	2017	A clinical study to evaluate the efficacy of Saindhavadi Taila Matra Vasti in the management of	No	Without intervention/comparator	Without intervention/comparator	-
2807	2018	Getting fit for hip and knee replacement: a protocol for the Fit-Joints pilot randomized controllec	No	Without intervention/comparator	Without intervention/comparator	-
2808	2015	Patient outcomes using Wii-enhanced rehabilitation after total knee replacement - the TKR-POW	No	Without intervention/comparator	Without intervention/comparator	-
2809	2015	The effects of aquatic exercise and joint supplements on pain and function of women with knee	No	Without intervention/comparator	Without intervention/comparator	-
2810	2019	Effect of a short message service (SMS) intervention on adherence to a physiotherapist-prescribe	No	Without intervention/comparator	Without intervention/comparator	-
2811	2019	Effectiveness of internet-delivered education and home exercise supported by behaviour change	No	Without intervention/comparator	Without intervention/comparator	-
2812	2017	Use of cationic contrast-enhanced computed tomography detects subtle equine articular cartilage	No	Without intervention/comparator	Without intervention/comparator	-
2813	2020	Telerehabilitation is non-inferior to usual care following total hip replacement Ã¢â¬â a randomiz	No	Without intervention/comparator	Without intervention/comparator	-

2814	2018	Evaluation of a digital platform for osteoarthritis treatment: study protocol for a randomised clin	No	Without intervention/comparator	Without intervention/comparator	-
2815	2015	Effectiveness of a progressive resistence strength programme on hand osteoarthritis: a randomize	No	Without intervention/comparator	Without intervention/comparator	-
2816	2016	Effectiveness of a progressive resistance strength program on hand osteoarthritis: a randomised c	No	Without intervention/comparator	Without intervention/comparator	-
2817	2010	Mini-midvastus vs standard medial parapatellar approach: a prospective, randomized, double-bli	No	Without intervention/comparator	Without intervention/comparator	-
2818	2007	Predictors of exercise and effects of exercise on symptoms, function, aerobic fitness, and disease	No	Without intervention/comparator	Without intervention/comparator	-
2819	2011	Beneficial effect of creatine supplementation in knee osteoarthritis	No	Without intervention/comparator	Without intervention/comparator	-
2820	2010	Efficacy of a progressive walking program and glucosamine sulphate supplementation on osteoa	No	Without intervention/comparator	Without intervention/comparator	-
2821	2010	Tai chi improves physical function in older Chinese women with knee osteoarthritis	No	Without intervention/comparator	Without intervention/comparator	-
2822	2013	Factors affecting adherence to the sarah trial hand exercise programme for rheumatoid arthritis: a	No	Without intervention/comparator	Without intervention/comparator	-
2823	2012	Six and 12 months' effects of individual joint protection education in people with rheumatoid art	No	Without intervention/comparator	Without intervention/comparator	-
2824	2018	The effect of intra-articular glucocorticosteroids and exercise on symptoms and bone marrow les	No	Without intervention/comparator	Without intervention/comparator	-
2825	2012	Efficacy of stabilisation splint treatment on temporomandibular disorders	No	Without intervention/comparator	Without intervention/comparator	-
2826	2017	HPR rehabilitation in warm climate for young adults with inflammatory rheumatic disease. A 12	No	Without intervention/comparator	Without intervention/comparator	-
2827	2010	The Artelon CMC spacer compared with tendon interposition arthroplasty	No	Without intervention/comparator	Without intervention/comparator	-
2828	2014	Effect of supplementation with a combination of b-hydroxy b-methylbutyrate, L-arginine, and L-	No	Without intervention/comparator	Without intervention/comparator	-
2829	2015	Effects of supplementation with a combination of Î²-hydroxy-Î²-methyl butyrate, L-arginine, and	No	Without intervention/comparator	Without intervention/comparator	-
2830	2018	Effects of strength training at different modalities and intensities on senescence-prone t-cells in p	No	Without intervention/comparator	Without intervention/comparator	-
2831	2018	Training-induced decrease in senescence-prone T-lymphocytes in peripheral blood: does cytome	No	Without intervention/comparator	Without intervention/comparator	-
2832	2019	Plants for Joints ACPA positive arthralgia	No	Other populations/diseases	Other populations/diseases	-
2833	2019	Plants for Joints OA	No	Without intervention/comparator	Without intervention/comparator	-
2834	2019	Plants for Joints RA	No	Without intervention/comparator	Without intervention/comparator	-
2835	2004	Efficacy of Intra-articular Sodium Hyaluronate in Patients with Osteoarthritis of the Knee	No	Without intervention/comparator	Without intervention/comparator	-
2836	2019	Anterior cruciate ligament reconstruction using an all-inside short-graft technique vs. a conventi	No	Without intervention/comparator	Without intervention/comparator	-
2837	1997	Effectiveness of aquatic exercise in the treatment of women with osteoarthritis	No	Without intervention/comparator	Without intervention/comparator	-
2838	2018	Prospective randomized controlled trial in the treatment of lateral epicondylitis with a new dynar	No	Other populations/diseases	Other populations/diseases	-
2839	2006	The efficacy of wrist working splints in patients with non-destructive wrist arthritis	No	Without intervention/comparator	Without intervention/comparator	-
2840	2007	The efficacy of a multidisciplinary treatment program in patients with osteoarthritis of the hands	No	Without intervention/comparator	Without intervention/comparator	-
2841	2008	Improving knee joint stability in osteoarthritis with exercise therapy: does it work?	No	Without intervention/comparator	Without intervention/comparator	-
2842	2009	The treatment of joint degeneration of the thumb with two different operation techniques	No	Without intervention/comparator	Without intervention/comparator	-
2843	2010	Preoperative strength training for patients awaiting total knee arthroplasty	No	Without intervention/comparator	Without intervention/comparator	-
2844	2010	The effectiveness of an internet physical activity/self-management program in patients with oste	No	Without intervention/comparator	Without intervention/comparator	-
2845	2010	Validity and feasability of self-assessment of joints by patients with rheumatoid arthritis and the	No	Without intervention/comparator	Without intervention/comparator	-
2846	2011	Comorbidity and exercise therapy in patients with knee osteoarthritis: RCT	No	Without intervention/comparator	Without intervention/comparator	-
2847	2011	Effect of Pregabaline and S-Ketamine on knee function after total knee arthroplasty	No	Without intervention/comparator	Without intervention/comparator	-
2848	2013	Kosteneffectiviteit van vroege chirurgie versus fysiotherapie met optionele verlate meniscectomi	No	Without intervention/comparator	Without intervention/comparator	-
2849	2013	Proeftuin Integrative Medicine	No	Without intervention/comparator	Without intervention/comparator	-
2850	2014	The effect of high-resistance muscle strength training and vitamin D supplementation in persons	No	Without intervention/comparator	Without intervention/comparator	-
2851	2015	activity coach in TKA following a homebased exercise program	No	Without intervention/comparator	Without intervention/comparator	-
2852	2015	Hand Osteoarthritis Prednisolone Efficacy study (HOPE)	No	Without intervention/comparator	Without intervention/comparator	-
2853	2017	Exercise in patients with Rheumatoid Arthritis and Cardiovascular risks	No	Without intervention/comparator	Without intervention/comparator	-
2854	2018	Better Involved (Beter Betrokken)	No	Without intervention/comparator	Without intervention/comparator	-
2855	2018	Manometric TMC brace: a randomised crossover trial	No	Without intervention/comparator	Without intervention/comparator	-
2856	2018	Physiotherapeutic Treat-to-target Intervention after Orthopaedic surgery; a cost-effectiveness stu	No	Without intervention/comparator	Without intervention/comparator	-
2857	2014	Short term recovery of function following total knee arthroplasty: a randomised study of the med	No	Without intervention/comparator	Without intervention/comparator	-

2858	2017	Anti-inflammatory effects of resistance training: dose-response relationship	No	Without intervention/comparator	Without intervention/comparator	-
2859	2013	The effect of action and coping plans on exercise adherence in people with lower limb osteoarthı	No	Without intervention/comparator	Without intervention/comparator	-
2860	2018	Improving physical activity, pain and function in patients waiting for hip and knee arthroplasty b	No	Without intervention/comparator	Without intervention/comparator	-
2861	2016	Effectiveness of a healthy lifestyle intervention for low back pain and osteoarthritis of the knee:]	No	Without intervention/comparator	Without intervention/comparator	-
2862	2011	Systematic review and meta-analysis: effects of walking exercise in chronic musculoskeletal pair	No	Review/Systematic Review/Meta-Analys	Review/Systematic Review/Meta-Analys	-
2863	2014	Is telephysiotherapy an option for improved quality of life in patients with osteoarthritis of the kı	No	Without intervention/comparator	Without intervention/comparator	-
2864	2019	"Partner in Prime"? Effects of repeated mobile security priming on attachment security and perc	No	Without intervention/comparator	Without intervention/comparator	-
2865	2018	Effect of a dynamic exercise program in combination with a mediterranean diet in weight and ha	No	Without intervention/comparator	Without intervention/comparator	-
2866	2018	Does kinesiology taping improve muscle strength and function in knee osteoarthritis? A single-b	No	Without intervention/comparator	Without intervention/comparator	-
2867	2013	Acupuncture for treatment of arthralgia secondary to aromatase inhibitor therapy in women with	No	Without intervention/comparator	Without intervention/comparator	-
2868	2016	Comparison of Treatments for Superior Labrum-Biceps Complex Lesions With Concomitant Ro	No	Without intervention/comparator	Without intervention/comparator	-
2869	2018	Arthroscopic Wafer Procedure Versus Ulnar Shortening Osteotomy as a Surgical Treatment for I	No	Without intervention/comparator	Without intervention/comparator	-
2870	2020	Physical fitness in individuals with knee osteoarthritis compared to normative data - a cross-sect	No	Without intervention/comparator	Without intervention/comparator	-
2871	2013	Efficacy of strength and aerobic exercise on patient-reported outcomes and structural changes in	No	Without intervention/comparator	Without intervention/comparator	-
2872	2016	Comparative effects of proprioceptive and isometric exercises on pain intensity and difficulty in	No	Without intervention/comparator	Without intervention/comparator	-
2873	2013	An assessment of the impact of behavioural cognitions on function in patients partaking in a tria	No	Without intervention/comparator	Without intervention/comparator	-
2874	2016	Does an early home-based progressive resistance training program improve function following to	No	Without intervention/comparator	Without intervention/comparator	-
2875	2016	Effectiveness of combined chain exercises on pain and function in patients with knee osteoarthri	No	Without intervention/comparator	Without intervention/comparator	-
2876	2008	Efficacy evaluation of nonpharmacological treatment (regular exercise), pharmacotherapy (gluco	No	Other populations/diseases	Other populations/diseases	-
2877	2017	Patient-Controlled Fentanyl Iontophoretic Transdermal System Improved Postoperative Mobility	No	Without intervention/comparator	Without intervention/comparator	-
2878	2012	Uselfulnes of chondroitin sulfate in the treament of hand osteothritis	No	Without intervention/comparator	Without intervention/comparator	-
2879	2012	Impact of exercise on the functional capacity and pain of patients with knee osteoarthritis: a ranc	No	Without intervention/comparator	Without intervention/comparator	-
2880	2016	The John Insall Award: no Functional Benefit After Unicompartmental Knee Arthroplasty Perfor	No	Without intervention/comparator	Without intervention/comparator	-
2881	2013	Efficacy of topical nonsteroidal anti-inflammatory drug (nsaids) adhesive skin patch in quadrice	No	Without intervention/comparator	Without intervention/comparator	-
2882	2017	Effects of combined intervention of quadriceps exercise and green tea catechins on physical func	No	Without intervention/comparator	Without intervention/comparator	-
2883	2018	Effects of combined intervention of quadriceps exercise and green tea catechins on physical func	No	Without intervention/comparator	Without intervention/comparator	-
2884	1996	The effect of tenoxicam on the changes of prostaglandin E2 levels in cases of osteoarthritis of the	No	Without intervention/comparator	Without intervention/comparator	-
2885	2011	Acute effects of combination of glucosamine sulphate lontophoresis with exercise on fasting plas	No	Without intervention/comparator	Without intervention/comparator	-
2886	2019	Musculoskeletal ultrasound in symptomatic thumb-base osteoarthritis: clinical, functional, radio	No	Without intervention/comparator	Without intervention/comparator	-
2887	2015	Feasibility of an intensive therapeutic exercise program for frail elderly prior to total hip arthropl	No	Without intervention/comparator	Without intervention/comparator	-
2888	2013	Cost-effectiveness of joint protection and hand exercise for hand osteoarthritis	No	Without intervention/comparator	Without intervention/comparator	-
2889	2014	Joint protection and hand exercises for hand osteoarthritis: an economic evaluation comparing m	No	Without intervention/comparator	Without intervention/comparator	-
2890	2019	Knee joint biomechanics in transtibial amputees in gait, cycling, and elliptical training	No	Without intervention/comparator	Without intervention/comparator	-
2891	2013	Comparative efficacy of gym high-intensity dynamic exercises and therapeutic exercises in patie	No	Without intervention/comparator	Without intervention/comparator	-
2892	2015	Comparative efficacy of two exercises programs in patients with early rheumatoid arthritis: 6-mo	No	Without intervention/comparator	Without intervention/comparator	-
2893	2015	Efficacy of 6-month complex rehabilitation program in patients with early rheumatoid arthritis r	No	Without intervention/comparator	Without intervention/comparator	-
2894	2015	Evaluation of influence of complex rehabilitation program on functional ability of rheumatoid ar	No	Without intervention/comparator	Without intervention/comparator	-
2895	2016	12-month complex rehabilitation program for rheumatoid arthritis patients receiving biologic dn	No	Without intervention/comparator	Without intervention/comparator	-
2896	2016	Efficacy of two long-term exercise programs in patients with early rheumatoid arthritis: results o	No	Without intervention/comparator	Without intervention/comparator	-
2897	2008	Efficacy of progressive resistance training on balance performance in older adults: a systematic r	No	Review/Systematic Review/Meta-Analys	Review/Systematic Review/Meta-Analys	-
2898	2015	Differences in physical fitness, disability, and health-related quality of life between physical acti	No	Other populations/diseases	Other populations/diseases	-
2899	2011	High dosage medical exercise therapy or arthroscopic treatment for patients with degenerative m	No	Without intervention/comparator	Without intervention/comparator	-
2900	2014	Exercise programme with telephone follow-up for people with hand osteoarthritis - Protocol for a	No	Without intervention/comparator	Without intervention/comparator	-
2901	2015	Implementing international osteoarthritis treatment guidelines in primary health care: study proto	No	Without intervention/comparator	Without intervention/comparator	-

2902	2020	Higher quality of care and less surgery after implementing osteoarthritis guidelines in primary ca	No	Without intervention/comparator	Without intervention/comparator	-
2903	2014	Effect of a combination of whole body vibration exercise and squat training on body balance, mu	No	Without intervention/comparator	Without intervention/comparator	-
2904	2017	Preliminary study of modifications of gait and adl to reduce knee pain and improve knee joint fu	No	Without intervention/comparator	Without intervention/comparator	-
2905	2013	Severe skeletal toxicity from protracted etidronate therapy for generalized arterial calcification o	No	Other populations/diseases	Other populations/diseases	-
2906	2016	The curative efficacy of arthroscopic therapy in treating anterior cruciate ligament rupture with s	No	Without intervention/comparator	Without intervention/comparator	-
2907	2010	(Cost) effectiveness of surgery versus prolonged conservative treatment in lumbar stenosis: desig	No	Without intervention/comparator	Without intervention/comparator	-
2908	2019	Effectiveness of continuous versus pulsed short-wave diathermy in the management of knee oste	No	Without intervention/comparator	Without intervention/comparator	-
2909	2010	Additional effect of pulsed electromagnetic field therapy on knee osteoarthritis treatment: a rand	No	Without intervention/comparator	Without intervention/comparator	-
2910	2018	The effectiveness of peloidotherapy and aquatic exercise in knee osteoarthritis treatment; A rand	No	Without intervention/comparator	Without intervention/comparator	-
2911	2015	THE EFFECTS OF A SIX-WEEK PHYSIOTHERAPIST-LED EXERCISE AND EDUCATION	No	Without intervention/comparator	Without intervention/comparator	-
2912	2017	Effects of KBA and proprioceptive exercises on knee osteoarthritis individuals	No	Without intervention/comparator	Without intervention/comparator	-
2913	2017	Efficacy of physical agent in the management of osteoarthritis	No	Without intervention/comparator	Without intervention/comparator	-
2914	2017	Efficacy of static stretching exercises versus proprioceptive neuromuscular facilitation stretching	No	Without intervention/comparator	Without intervention/comparator	-
2915	2017	Ultrasound-Guided Adductor Canal Block for Treatment of Chronic Osteoarthritis Knee Pain	No	Without intervention/comparator	Without intervention/comparator	-
2916	2018	Cost-effectiveness of an outpatient self-management program for individuals with knee osteoartl	No	Without intervention/comparator	Without intervention/comparator	-
2917	2018	Effect of Glucosamine Sulfate, Chondrotin Sulfate, Methylsulfonylmethane Phonophoresis on Pa	No	Without intervention/comparator	Without intervention/comparator	-
2918	2018	Effect of Magnetic Stimulation on Cross-Sectional Area of Quadriceps Muscle in Knee Osteoart	No	Without intervention/comparator	Without intervention/comparator	-
2919	2018	Effects of Kinesio Taping Plus Combined Chain Exercises Compared to Combined Chain Exerc	No	Without intervention/comparator	Without intervention/comparator	-
2920	2018	High Intensity Laser versus Low Intensity Pulse Ultrasound on Pain, Range of motion, Posi-tion	No	Without intervention/comparator	Without intervention/comparator	-
2921	2019	Effectiveness of Manual therapy on pain, activity and quality of life in beninese hip osteoarthriti	No	Without intervention/comparator	Without intervention/comparator	-
2922	2018	Protocol for a single-centre, parallel-arm, double-blind randomised trial evaluating the effects of	No	Without intervention/comparator	Without intervention/comparator	-
2923	2019	Arthroscopic hip surgery compared with physiotherapy and activity modification for the treatme	No	Without intervention/comparator	Without intervention/comparator	-
2924	2010	Short-term effects of Transcutaneous Electrical Nerve Stimulation (TENS) and exercise on knee	No	Without intervention/comparator	Without intervention/comparator	-
2925	2011	Blinding of transcutaneous electrical nerve stimulation (TENS): insights from a randomised plac	No	Without intervention/comparator	Without intervention/comparator	-
2926	2014	Transcutaneous electrical nerve stimulation as an adjunct to education and exercise for knee oste	No	Without intervention/comparator	Without intervention/comparator	-
2927	2010	A clinical trial of neuromuscular electrical stimulation in improving quadriceps muscle strength	No	Without intervention/comparator	Without intervention/comparator	-
2928	2015	Effects of Osteoarthritis on Quality of life in Elderly Population of Bhubaneswar, India: a Prospe	No	Without intervention/comparator	Without intervention/comparator	-
2929	2012	The chaos falls clinic in preventing falls and injuries among home-dwelling older adults: a rando	No	Other populations/diseases	Other populations/diseases	-
2930	2014	Synergic effects of ultrasound and laser on the pain relief in women with hand osteoarthritis	No	Without intervention/comparator	Without intervention/comparator	-
2931	2015	Synergic effects of ultrasound and laser on the pain relief in women with hand osteoarthritis	No	Without intervention/comparator	Without intervention/comparator	-
2932	2018	Ultrasound plus low-level laser therapy for knee osteoarthritis rehabilitation: a randomized, plac	No	Without intervention/comparator	Without intervention/comparator	-
2933	2018	Ultrasound plus low-level laser therapy for knee osteoarthritis rehabilitation: a randomized, plac	No	Without intervention/comparator	Without intervention/comparator	-
2934	2013	Differences in fixation stability between spacer plate and plate fixator following high tibial osteo	No	Without intervention/comparator	Without intervention/comparator	-
2935	2015	A knee monitoring device and the preferences of patients living with osteoarthritis: a qualitative	No	Without intervention/comparator	Without intervention/comparator	-
2936	2018	Mathematical evaluation of melatonin secretion in hypoxic ischemic encephalopathy	No	Other populations/diseases	Other populations/diseases	-
2937	2018	Immediate effect of taping in physical performance of osteoarthritis of knee joint	No	Without intervention/comparator	Without intervention/comparator	-
2938	2003	Preoperative predictors of locomotor ability two months after total knee arthroplasty for severe o	No	Without intervention/comparator	Without intervention/comparator	-
2939	2006	The effects of exercise following a corticosteroid injection for knee osteoarthritis: a pilot study	No	Without intervention/comparator	Without intervention/comparator	-
2940	2017	Cerebral Blood Flow Responses to Aquatic Treadmill Exercise	No	Without intervention/comparator	Without intervention/comparator	-
2941	2019	Effects of Robotic (RT) vs Treadmill Training (TT) on Pain and Neuromechanical Charateristics	No	Without intervention/comparator	Without intervention/comparator	-
2942	2014	The effect of Sit 'n' Fit Chair Yoga among community-dwelling older adults with osteoarthritis	No	Without intervention/comparator	Without intervention/comparator	-
2943	2015	Strategies of recruitment and retention of older adults with osteoarthritis for a yoga intervention:	No	Without intervention/comparator	Without intervention/comparator	-
2944	2016	The Effect of Chair Yoga on Biopsychosocial Changes in English- and Spanish-Speaking Comn	No	Without intervention/comparator	Without intervention/comparator	-
2945	2017	A Pilot Randomized Controlled Trial of the Effects of Chair Yoga on Pain and Physical Functior	No	Without intervention/comparator	Without intervention/comparator	-

2946	2020	What are the clinical, methodological, logistical and operational challenges when designing and	No	Without intervention/comparator	Without intervention/comparator	-
2947	2013	A randomised controlled trial to evaluate the efficacy of a health maintenance clinic intervention	No	Without intervention/comparator	Without intervention/comparator	-
2948	2020	Clinical and cost-effectiveness of ultrasound-guided intra-articular corticosteroid and local anaes	No	Without intervention/comparator	Without intervention/comparator	-
2949	2016	Expanding treatment of chronic pain in older adults: community- based exercise + CBT	No	Without intervention/comparator	Without intervention/comparator	-
2950	2018	A pragmatic pilot randomized controlled trial of the OA go away, a symptom and exercise tracki	No	Without intervention/comparator	Without intervention/comparator	-
2951	2013	Intra-articular injection of platelet-rich plasma in patients with knee osteoarthritis: a randomised	No	Without intervention/comparator	Without intervention/comparator	-
2952	2016	Intra-articular injection of photo-activated platelet-rich plasma in patients with knee osteoarthriti	No	Without intervention/comparator	Without intervention/comparator	-
2953	2018	Efficacy of intra-articular injections of platelet-rich plasma as a symptom- and disease-modifying	No	Without intervention/comparator	Without intervention/comparator	-
2954	2018	Footwear for self-managing knee osteoarthritis symptoms: protocol for the Footstep randomised	No	Without intervention/comparator	Without intervention/comparator	-
2955	2018	Moderators and mediators of effects of unloading shoes on knee pain in people with knee osteoa	No	Without intervention/comparator	Without intervention/comparator	-
2956	2019	A multi-faceted, non-drug, non-surgical intervention compared to usual GP care for symptomatic	No	Without intervention/comparator	Without intervention/comparator	-
2957	2019	Podiatry intervention versus usual general practitioner care for symptomatic radiographic osteoa	No	Without intervention/comparator	Without intervention/comparator	-
2958	2020	Footwear for osteoarthritis of the lateral knee: protocol for the FOLK randomised controlled trial	No	Without intervention/comparator	Without intervention/comparator	-
2959	2010	Randomized controlled trial of exercise fall prevention program in elderly women with osteoporo	No	Without intervention/comparator	Without intervention/comparator	-
2960	2015	Efficacy and safety of rumalaya forte to patients with hand osteoarthritis (HOA)	No	Without intervention/comparator	Without intervention/comparator	-
2961	2017	Effects of pilates exercises on physical functioning in postmenopausal osteopo-rosis women	No	Other populations/diseases	Other populations/diseases	-
2962	2017	Effects of tai chi exercise on muscle strength and balance in postmenopau-salwomen with osteop	No	Without intervention/comparator	Without intervention/comparator	-
2963	2018	Effects of an intensive exercise program on rheumatoid arthritis patients	No	Without intervention/comparator	Without intervention/comparator	-
2964	2019	Exercise-therapy and education for early-onset knee osteoarthritis following ACL reconstruction	No	Without intervention/comparator	Without intervention/comparator	-
2965	1993	Protocol development for combination therapy with disease-modifying antirheumatic drugs	No	Without intervention/comparator	Without intervention/comparator	-
2966	2012	The effect of low frequency pulsing electromagnetic field in treatment of patients with knee joint	No	Without intervention/comparator	Without intervention/comparator	-
2967	2018	A Feasibility Study for Improved Physical Activity After Total Knee Arthroplasty	No	Without intervention/comparator	Without intervention/comparator	-
2968	2018	Safety and feasibility of high speed resistance training with and without balance exercises for kn	No	Without intervention/comparator	Without intervention/comparator	-
2969	2019	Effectiveness of Watsu therapy in patients with juvenile idiopathic arthritis. A parallel, randomiz	No	Without intervention/comparator	Without intervention/comparator	-
2970	2017	Effectiveness of a fine motor skills rehabilitation program on upper limb disability, manual dexte	No	Without intervention/comparator	Without intervention/comparator	-
2971	2019	Maximal heart rate assessment in recreational football players: a study involving a multiple testi	No	Other populations/diseases	Other populations/diseases	-
2972	2015	Alpine Skiing With total knee ArthroPlasty (ASWAP): effects on strength and cardiorespiratory	No	Without intervention/comparator	Without intervention/comparator	-
2973	2019	Neural manual mobilization vs robotic assisted mobilization to reduce pain hypersensitivity in ha	No	Without intervention/comparator	Without intervention/comparator	-
2974	2019	The effects of neurodynamic mobilizations on pain hypersensitivity in patients with hand osteoa	No	Without intervention/comparator	Without intervention/comparator	-
2975	2010	A walking model to assess the onset of analgesia in osteoarthritis knee pain	No	Without intervention/comparator	Without intervention/comparator	-
2976	2014	Continuous femoral nerve block versus intravenous patient controlled analgesia for knee mobility	No	Without intervention/comparator	Without intervention/comparator	-
2977	2015	A MULTICENTER, RANDOMIZED, DOUBLE-BLIND, PLACEBO-CONTROLLED, PARAL	No	Without intervention/comparator	Without intervention/comparator	-
2978	2014	The effects of end-of-range grade A+ mobilisation following acute primary TKA	No	Without intervention/comparator	Without intervention/comparator	-
2979	2015	The effects of end-of-range grade a+ mobilisation following acute primary TKA	No	Other populations/diseases	Other populations/diseases	-
2980	2016	Effectiveness of a fine motor skills rehabilitation program on upper limb disability, manual dexte	No	Other populations/diseases	Other populations/diseases	-
2981	2017	Safety of buprenorphine transdermal system in the management of pain in older adults	No	Other populations/diseases	Other populations/diseases	-
2982	2020	Metformin to reduce metabolic complications and inflammation in patients on systemic glucoco	No	Other populations/diseases	Other populations/diseases	-
2983	2012	The influence of sport participation on physical function in patients with osteoarthritis during an	No	Without intervention/comparator	Without intervention/comparator	-
2984	2018	Falls among persons with Alzheimer disease: description, risk factors, and exercise interacting th	No	Other populations/diseases	Other populations/diseases	-
2985	2015	The effectiveness of the Push Braceâ¢ orthosis and corticosteroid injection for managing first ca	No	Other populations/diseases	Other populations/diseases	-
2986	2010	Glucosamine but not ibuprofen alters cartilage turnover in osteoarthritis patients in response to p	No	Without intervention/comparator	Without intervention/comparator	-
2987	2011	Exercise and NSAIDs: effect on muscle protein synthesis in patients with knee osteoarthritis	No	Without intervention/comparator	Without intervention/comparator	-
2988	2014	The Patella Pro study - effect of a knee brace on patellofemoral pain syndrome: design of a rando	No	Other populations/diseases	Other populations/diseases	-
2989	2016	Evaluating the potential synergistic benefit of a realignment brace on patients receiving exercise	No	Other populations/diseases	Other populations/diseases	-

2990	2017	Measuring postural control during mini-squat posture in men with early knee osteoarthritis	No	Without intervention/comparator	Without intervention/comparator	-
2991	2000	Is exercise effective treatment for osteoarthritis of the knee?	No	Without intervention/comparator	Without intervention/comparator	-
2992	2012	Randomized, double-blind control trial of peri-articular hyaluronic acid: botulinus toxin injectio	No	Other populations/diseases	Other populations/diseases	-
2993	2014	The effectiveness of Thai exercise with traditional massage on the pain, walking ability and QOI	No	Without intervention/comparator	Without intervention/comparator	-
2994	2016	The effect of cardiac rehabilitation program on disease activity, functional outcome and cardiova	No	Without intervention/comparator	Without intervention/comparator	-
2995	2017	[Physical Activity Recommendations for Adults with a Chronic Disease: Methods, Database and	No	Other populations/diseases	Other populations/diseases	-
2996	2019	Addition of transcutaneous electrical nerve stimulation to therapeutic exercise and activities of d	No	Other populations/diseases	Other populations/diseases	-
2997	2020	Using TENS to Enhance Therapeutic Exercise in Individuals with Knee Osteoarthritis	No	Without intervention/comparator	Without intervention/comparator	-
2998	2009	Immediate effects of transcutaneous electrical nerve stimulation and focal knee joint cooling on	No	Without intervention/comparator	Without intervention/comparator	-
2999	2010	Contralateral effects of disinhibitory TENS on quadriceps function in people with knee osteoartl	No	Without intervention/comparator	Without intervention/comparator	-
3000	2010	Validity and feasability of self-assessment of joints by patients with rheumatoid arthritis and the	No	Without intervention/comparator	Without intervention/comparator	-
3001	2011	Effects of transcutaneous electrical nerve stimulation and therapeutic exercise on quadriceps acti	No	Without intervention/comparator	Without intervention/comparator	-
3002	2012	Changes in voluntary quadriceps activation predict changes in quadriceps strength after therapeu	No	Without intervention/comparator	Without intervention/comparator	-
3003	2014	Telemedicine applied to kinesiotherapy for hand dysfunction in patients with systemic sclerosis a	No	Without intervention/comparator	Without intervention/comparator	-
3004	2018	Level of participation in physical therapy or an internet-based exercise training program: associa	No	Without intervention/comparator	Without intervention/comparator	-
3005	2018	Engagement with physical therapy or an internet-based exercise training program and associatio	No	Without intervention/comparator	Without intervention/comparator	-
3006	1993	Limitations of randomized clinical trials to recognize possible advantages of combination therap	No	Without intervention/comparator	Without intervention/comparator	-
3007	2016	Effect of a dynamic exercise program in combination with a mediterranean diet in strength, joint	No	Without intervention/comparator	Without intervention/comparator	-
3008	2020	Concurrent training after photobiomodulation in patients with knee osteoarthritis	No	Without intervention/comparator	Without intervention/comparator	-
3009	2018	Threshold pain after photobiomodulation associated to neuromuscular training in patients with g	No	Without intervention/comparator	Without intervention/comparator	-
3010	2020	A randomized controlled trial to reduce sedentary time in rheumatoid arthritis: protocol and ratic	No	Without intervention/comparator	Without intervention/comparator	-
3011	2013	Manual therapy, exercise therapy, or both, in addition to usual care, for osteoarthritis of the hip c	No	Without intervention/comparator	Without intervention/comparator	-
3012	2013	Manual therapy, exercise therapy, or both, in addition to usual care, for osteoarthritis of the hip c	No	Without intervention/comparator	Without intervention/comparator	-
3013	2013	Effectiveness of an interactive virtual telerehabilitation system in patients after total knee arthopl	No	Without intervention/comparator	Without intervention/comparator	-
3014	2011	Long-term effect of exercise therapy in patients with osteoarthritis: a randomized controlled trial	No	Without intervention/comparator	Without intervention/comparator	-
3015	2007	Long-term effectiveness of exercise therapy in patients with osteoarthritis of the hip or knee: a sy	No	Review/Systematic Review/Meta-Analys	Review/Systematic Review/Meta-Analys	-
3016	2010	Behavioural graded activity results in better exercise adherence and more physical activity than u	No	Without intervention/comparator	Without intervention/comparator	-
3017	2010	Long-term effectiveness of exercise therapy in patients with osteoarthritis of the hip or knee: a ra	No	Without intervention/comparator	Without intervention/comparator	-
3018	2007	Spa water more effective than tap water for knee osteoarthritis: commentary	No	Without intervention/comparator	Without intervention/comparator	-
3019	2010	Spa therapy for knee osteoarthritis - A fair test?	No	Without intervention/comparator	Without intervention/comparator	-
3020	2010	A balance exercise program appears to improve function for patients with total knee arthroplasty	No	Without intervention/comparator	Without intervention/comparator	-
3021	2011	Contribution of hip abductor strength to physical function in patients with total knee arthroplasty	No	Without intervention/comparator	Without intervention/comparator	-
3022	2015	A randomized trial to compare exercise treatment methods for patients after total knee replaceme	No	Without intervention/comparator	Without intervention/comparator	-
3023	2018	Randomized trial on exercise at late-stage after total knee replacement	No	Without intervention/comparator	Without intervention/comparator	-
3024	2019	Effectiveness of Later-Stage Exercise Programs vs Usual Medical Care on Physical Function and	No	Without intervention/comparator	Without intervention/comparator	-
3025	2019	Neuromuscular Electrical Stimulation Compared to Volitional Exercise for Improving Muscle Fu	No	Without intervention/comparator	Without intervention/comparator	-
3026	2014	Immediate effects of electroacupuncture and manual acupuncture on pain, mobility and muscle s	No	Without intervention/comparator	Without intervention/comparator	-
3027	2015	Impact of diet and/or exercise intervention on infrapatellar fat pad morphology-data from the int	No	Without intervention/comparator	Without intervention/comparator	-
3028	2016	At which location is the infrapatellar fat pad most sensitive to reduction by diet and exercise inte	No	Without intervention/comparator	Without intervention/comparator	-
3029	2017	Impact of Diet and/or Exercise Intervention on Infrapatellar Fat Pad Morphology: secondary Ana	No	Without intervention/comparator	Without intervention/comparator	-
3030	2017	The effectiveness of transcutaneous electrical nerve stimulation in knee osteoarthritis with neuro	No	Without intervention/comparator	Without intervention/comparator	-
3031	2016	Liposomal bupivacaine adductor canal nerve block, posterior capsule block and lateral genicular	No	Other populations/diseases	Other populations/diseases	-
3032	2016	Effects of postural and respiratory rehabilitation in patients with hyper-kyphosis by osteoporotic	No	Other populations/diseases	Other populations/diseases	-
3033	2013	Subluxation of the shoulder joint in stroke patients and the influence of selected factors on the in	No	Other populations/diseases	Other populations/diseases	-

3034	2014	Physicaltherapy benefits upon functional status and fall risk in osteoporotic women	No	Other populations/diseases	Other populations/diseases	-
3035	2011	Is yoga effective for pain? A systematic review of randomized clinical trials	No	Review/Systematic Review/Meta-Analys	Review/Systematic Review/Meta-Analys	-
3036	2011	Non-surgical treatment of hip osteoarthritis. Hip school, with or without the addition of manual t	No	Without intervention/comparator	Without intervention/comparator	-
3037	2012	Reproducibility of range of motion and muscle strength measurements in patients with hip osteo	No	Without intervention/comparator	Without intervention/comparator	-
3038	2018	The beneficial effect of Maitland's manual therapy on muscle mechanic and knee function durin	No	Without intervention/comparator	Without intervention/comparator	-
3039	2019	Influence of medial and lateral hamstring strengthening exercises in women with osteoarthritis k	No	Without intervention/comparator	Without intervention/comparator	-
3040	2013	The effect of a single high dose of cholecalciferol on motor performance in postmenopausalwom	No	Other populations/diseases	Other populations/diseases	-
3041	1963	Therapeutic trial of a pyrazole derivative, KB-95, for patients with rheumatoid arthritis	No	Without intervention/comparator	Without intervention/comparator	-
3042	2018	HPR inflammatory or rheumatoid arthritis patients' perspectives on the effect of arthritis gloves c	No	Without intervention/comparator	Without intervention/comparator	-
3043	2014	A three month controlled intervention of intermittent whole body vibration designed to improve	No	Without intervention/comparator	Without intervention/comparator	-
3044	2014	Rigid versus semi-rigid orthotic use following TMC arthroplasty: a randomized controlled trial	No	Without intervention/comparator	Without intervention/comparator	-
3045	2019	Intensive management for moderate rheumatoid arthritis: a qualitative study of patients' and prac	No	Without intervention/comparator	Without intervention/comparator	-
3046	2016	Clinical study of warm acupuncture and moxibustion combined with acupoint injection of Cervu	No	Without intervention/comparator	Without intervention/comparator	-
3047	2011	Associations of knee extensor strength and standing balance with physical function in knee osteo	No	Without intervention/comparator	Without intervention/comparator	-
3048	2018	Effect of Tai Chi on the rehabilitation of neck type cervical spondylosis in the elderly	No	Other populations/diseases	Other populations/diseases	-
3049	2019	Acupuncture combined with cinesiotherapy cupping for knee osteoarthritis with qi stagnation an	No	Without intervention/comparator	Without intervention/comparator	-
3050	2015	Causes of changes in carotid intima-media thickness: a literature review	No	Review/Systematic Review/Meta-Analys	Review/Systematic Review/Meta-Analys	-
3051	2016	Can attitudes and beliefs about exercise predict future physical activity level in older adults with	No	Other populations/diseases	Other populations/diseases	-
3052	2016	Is change in physical activity level associated with future clinical outcomes in terms of pain and	No	Other populations/diseases	Other populations/diseases	-
3053	2017	Relationship Between Attitudes and Beliefs and Physical Activity in Older Adults With Knee Pa	No	Other populations/diseases	Other populations/diseases	-
3054	2018	Change in physical activity level and clinical outcomes in older adults with knee pain: a seconda	No	Other populations/diseases	Other populations/diseases	-
3055	2017	Effect of foot insoles in patients with rheumatic joint disease	No	Without intervention/comparator	Without intervention/comparator	-
**3056**	**2019**	**Effects of Strength Training with Reduced Blood Flow in Patients with Wear at the Knee J**	**Yes**	**Full**	**Full**	**-**
3057	2018	The effect of treatment, sex, and severity of knee osteoarthritis on pain, physical function, qualit	No	Without intervention/comparator	Without intervention/comparator	-
3058	2016	Effects of electrical stimulation over the skull and tight together with exercises on knee osteoarth	No	Without intervention/comparator	Without intervention/comparator	-
3059	2018	The effect of auriculotherapy and mechanical vibrations generated on oscillating vibratory platfo	No	Without intervention/comparator	Without intervention/comparator	-
3060	2019	Development of a mobile application to apply questionnaires on diseases in the knees	No	Without intervention/comparator	Without intervention/comparator	-
3061	2018	Does the laser in combination with exercises improve pain and function of patients with knee os	No	Without intervention/comparator	Without intervention/comparator	-
3062	2013	Impact of a Program of Subaquatic Therapeutic Exercises in Elderly Women with Knee Osteoart	No	Without intervention/comparator	Without intervention/comparator	-
3063	2019	Physiotherapeutic treatment in people who underwent hip replacement surgery	No	Without intervention/comparator	Without intervention/comparator	-
3064	2017	Effect of Strength Training associated with muscular electric current on the structure and functic	No	Other populations/diseases	Other populations/diseases	-
3065	2018	Effects of intraarticular infiltration in rheumatoid arthritis womenÂ´s knees	No	Without intervention/comparator	Without intervention/comparator	-
3066	2016	Effectiveness of propolis in reducing the symptoms of rheumatic diseases in humans	No	Without intervention/comparator	Without intervention/comparator	-
3067	2018	Effect of hip muscle strengthening in patients with Osteoarthritis of the knee	No	Without intervention/comparator	Without intervention/comparator	-
3068	2019	Effects of Type II Collagen Association on Muscle Strength in Pain and Function of Patients wit	No	Without intervention/comparator	Without intervention/comparator	-
3069	2014	Laser therapy in patients with knee artrosis	No	Without intervention/comparator	Without intervention/comparator	-
3070	2019	Effects of Occlusion Exercise on blood pressure in patients with Knee Arthrosis	No	Without intervention/comparator	Without intervention/comparator	-
3071	2019	Clay poultice combined with therapeutic exercises for pain reduction in patients with osteoarthri	No	Without intervention/comparator	Without intervention/comparator	-
3072	2016	Aquatic physiotherapy in pain, daily activities and quality of life in older people with knee Osteo	No	Without intervention/comparator	Without intervention/comparator	-
3073	2016	Low Level Laser Therapy and exercise to patients with chronic pain and rheumatic diseases: ran	No	Without intervention/comparator	Without intervention/comparator	-
3074	2019	Effects of treatment by Telecommunication Services on the pain, functionality and amount of fat	No	Without intervention/comparator	Without intervention/comparator	-
3075	2015	Effect of physical therapy in the treatment of knee osteoarthritis	No	Without intervention/comparator	Without intervention/comparator	-
3076	2019	Homeopathy in the integrative treatment of knee osteoarthritis	No	Without intervention/comparator	Without intervention/comparator	-
3077	2019	Effects of a single session of Exercise in the blood pressure of patients with Rheumatoid Arthriti	No	Without intervention/comparator	Without intervention/comparator	-

3078	2018	Laser Therapy on Pain Intensity of patients with advanced knee Osteoarthritis	No	Without intervention/comparator	Without intervention/comparator	-
3079	2019	The effect of movement velocity on the performance of leg press exercise in the musculature of e	No	Without intervention/comparator	Without intervention/comparator	-
3080	2016	Comparison of hydrotherapy anddry land exercise associated with lasertherapy on pain relief, str	No	Without intervention/comparator	Without intervention/comparator	-
3081	2015	Effects of taping in pain and function of patients with knee arthrosis: a blind randomised clinica	No	Without intervention/comparator	Without intervention/comparator	-
**3082**	**2018**	**Light strength exercise associated with Partial Occlusion of blood in the treatment of knee**	**Yes**	**Full**	**Full**	**-**
3083	2018	The effect of knee braces in patients with knee arthrosis	No	Without intervention/comparator	Without intervention/comparator	-
3084	2011	Strengthening exercises for strength and ability to manipulate objects in people with rheumatoid	No	Without intervention/comparator	Without intervention/comparator	-
3085	2014	Effect of elastic bandages on pain, swelling and strength in elderly subjects with knee osteoarthr	No	Without intervention/comparator	Without intervention/comparator	-
3086	2015	Effect of low-level laser and exercise improving balance and functionality in people with knee os	No	Without intervention/comparator	Without intervention/comparator	-
3087	2018	Effects of laser and physical exercise program in patients with knee arthritis	No	Without intervention/comparator	Without intervention/comparator	-
3088	2019	Knee Osteoarthritis: comparison between sensory motor training with sensory information and n	No	Without intervention/comparator	Without intervention/comparator	-
3089	2016	Muscle strengthening and aerobic training associated with phototherapy in knee osteoarthritis	No	Without intervention/comparator	Without intervention/comparator	-
3090	2018	Study of the therapeutic effect of an added gel with Pequi Oil on the functionality of patients wit	No	Without intervention/comparator	Without intervention/comparator	-
3091	2014	The effect of elastic bandage in patients with osteoarthritis of the knee	No	Without intervention/comparator	Without intervention/comparator	-
3092	2016	Impact of hidrotherapy and dry land exercises in daily activities and quality of life of older peopl	No	Without intervention/comparator	Without intervention/comparator	-
3093	2017	The Effect of different electrotherapy modalities on pain, funtion and life quality maintenance or	No	Without intervention/comparator	Without intervention/comparator	-
3094	2017	Measurement of thigh muscle strength in patients with knee arthrosis who will receive a lubricat	No	Without intervention/comparator	Without intervention/comparator	-
3095	2013	Association between disease-specific quality of life and magnetic resonance imaging outcomes i	No	Without intervention/comparator	Without intervention/comparator	-
3096	2013	Clinical and magnetic resonance outcomes in a study of prolotherapy for knee osteoarthritis: evi	No	Without intervention/comparator	Without intervention/comparator	-
3097	2013	Dextrose prolotherapy for knee osteoarthritis: a randomized controlled trial	No	Without intervention/comparator	Without intervention/comparator	-
3098	2015	Hypertonic dextrose injection (prolotherapy) for knee osteoarthritis: long term outcomes	No	Without intervention/comparator	Without intervention/comparator	-
3099	2011	Dextrose prolotherapy for knee osteoarthritis: results of a randomized controlled trial	No	Without intervention/comparator	Without intervention/comparator	-
3100	2018	Evaluation of the Combined Application of Neuromuscular Electrical Stimulation and Volitiona	No	Without intervention/comparator	Without intervention/comparator	-
3101	2012	Deep heating therapy via microwave diathermy relieves pain and improves physical function in p	No	Without intervention/comparator	Without intervention/comparator	-
3102	2015	Effects of focal muscle vibration on physical functioning in patients with knee osteoarthritis: a ra	No	Without intervention/comparator	Without intervention/comparator	-
3103	2020	In stable CAD with type 2 diabetes, adding ticagrelor to aspirin reduced CV events but increased	No	Other populations/diseases	Other populations/diseases	-
3104	2015	Knee osteoarthritis injection choices: platelet-rich plasma (PRP) versus hyaluronic acid (A one-y	No	Without intervention/comparator	Without intervention/comparator	-
3105	2018	Does platelet rich plasma change the volumetric findings of mri imaging? (a randomised clinical	No	Other populations/diseases	Other populations/diseases	-
3106	2018	The efficacy of electromyographic biofeedback on pain, function, and maximal thickness of vast	No	Without intervention/comparator	Without intervention/comparator	-
3107	2018	Volumetric findings of MRI after platelet rich plasma injection in knee osteoarthritis (A randomi	No	Without intervention/comparator	Without intervention/comparator	-
3108	1967	A three-month trial of indomethacin in rheumatoid arthritis, with special reference to analysis an	No	Without intervention/comparator	Without intervention/comparator	-
3109	2013	Efficacy of physical exercice in patients with knee osteoarthritis	No	Without intervention/comparator	Without intervention/comparator	-
3110	2019	Kinesio Taping Improves Perceptions of Pain and Function of Patients With Knee Osteoarthritis	No	Without intervention/comparator	Without intervention/comparator	-
3111	2012	Effects of strengthening and aerobic exercises on pain severity and function in patients with knee	No	Without intervention/comparator	Without intervention/comparator	-
3112	2017	The effect of injury to anterolateral capsular structures on outcomes of acl injured patients 24 mo	No	Other populations/diseases	Other populations/diseases	-
3113	2011	Pathomechanisms and complications related to patient positioning and anesthesia during should	No	Without intervention/comparator	Without intervention/comparator	-
3114	2018	Effect of isokinetic training on quadriceps muscle strength in osteoarthritis of knee	No	Without intervention/comparator	Without intervention/comparator	-
3115	2014	Transcutaneous electrical nerve stimulation for the control of pain during rehabilitation after tota	No	Without intervention/comparator	Without intervention/comparator	-
3116	1996	The effect of progressive resistance training in rheumatoid arthritis. Increased strength without c	No	Without intervention/comparator	Without intervention/comparator	-
3117	2015	Effect of cycle ergometer and conventional exercises on rehabilitation of older patients with total	No	Without intervention/comparator	Without intervention/comparator	-
3118	1998	Does a structured exercise program benefit elderly people with knee osteoarthritis?	No	Without intervention/comparator	Without intervention/comparator	-
3119	2009	Efficiency of Vibration or Strength Training for Knee Stability in Osteoarthritis of the Knee	No	Without intervention/comparator	Without intervention/comparator	-
3120	2019	Comparisons of Neuromuscular Training Versus Quadriceps Training on Gait and WOMAC Ind	No	Without intervention/comparator	Without intervention/comparator	-
3121	2014	Does intra articular platelet rich plasma injection improve function, pain and quality of life in pa	No	Without intervention/comparator	Without intervention/comparator	-

3122	2013	Pharmacological management : osteoporosis and osteoarthritis, Yesilarities and differences	No	Without intervention/comparator	Without intervention/comparator	-
3123	2014	Efficacy and safety of strontium ranelate in the treatment of knee osteoarthritis: results of a doub	No	Without intervention/comparator	Without intervention/comparator	-
3124	2011	A qualitative study of exercise habits of individuals with rheumatoid arthritis taking anti-tnfmed	No	Without intervention/comparator	Without intervention/comparator	-
3125	2011	Effect of dynamic exercise on function in people with rheumatoid arthritis taking anti-TNFalpha	No	Without intervention/comparator	Without intervention/comparator	-
3126	2011	Randomised controlled trial examining the effect of exercise in people with rheumatoid arthritis	No	Without intervention/comparator	Without intervention/comparator	-
3127	2011	Effects of a six week lower limb stretching programme on range of motion, peak passive torque a	No	Without intervention/comparator	Without intervention/comparator	-
3128	2013	Higher leg extensor muscle power output is associated with reduced pain and better quality of lif	No	Without intervention/comparator	Without intervention/comparator	-
3129	2015	Muscle power is an independent determinant of pain and quality of life in knee osteoarthritis	No	Without intervention/comparator	Without intervention/comparator	-
3130	2012	Exercise prescription for hospitalized people with chronic obstructive pulmonary disease and co	No	Without intervention/comparator	Without intervention/comparator	-
3131	2011	ESC/EAS Guidelines for the management of dyslipidaemias: the Task Force for the managemen	No	Without intervention/comparator	Without intervention/comparator	-
3132	2014	Effects of whole body vibration exercise associated with quadriceps resistance exercise on functi	No	Without intervention/comparator	Without intervention/comparator	-
3133	2014	Intraarticular analgesia versus epidural plus femoral nerve block after TKA: a randomized, doubl	No	Without intervention/comparator	Without intervention/comparator	-
3134	2020	Effect of microscope intervention on taijiquan on clinical rehabilitation of elderly patients with k	No	Without intervention/comparator	Without intervention/comparator	-
3135	2015	Comparison of minimally invasive approach versus conventional anterolateral approach for total	No	Without intervention/comparator	Without intervention/comparator	-
3136	2016	Effect of aqua-cycling on pain and physical functioning compared with usual care in patients wi	No	Without intervention/comparator	Without intervention/comparator	-
3137	2020	Aquatic Cycling Improves Knee Pain and Physical Functioning in Patients With Knee Osteoarth	No	Without intervention/comparator	Without intervention/comparator	-
3138	2020	Effect of 8-week aerobic walking program on sexual function in women with rheumatoid arthriti	No	Without intervention/comparator	Without intervention/comparator	-
3139	2013	Parqve A- project arthritis recovering quality of lifeby means of education A- a pilot study in bra	No	Without intervention/comparator	Without intervention/comparator	-
3140	2015	Analysis and correlation of physical therapy tests, pain, function and quality of life questionnair	No	Without intervention/comparator	Without intervention/comparator	-
3141	2015	Two year results of multiprofessional care and education in osteoarthritis	No	Without intervention/comparator	Without intervention/comparator	-
3142	2018	Educational program improves function, strength and quality of life. Two-year results of parve ii	No	Without intervention/comparator	Without intervention/comparator	-
3143	2018	Educational program in subjects with knee osteoarthritis improves adherence, aerobic and functi	No	Without intervention/comparator	Without intervention/comparator	-
3144	2019	Differences between an exclusive educational and the adding of multimodal and multiprofession	No	Without intervention/comparator	Without intervention/comparator	-
3145	2020	During two years, what is the difference between an exclusive two-day educational program on (	No	Without intervention/comparator	Without intervention/comparator	-
3146	2020	Four-year follow-up of parqve study - a two day educational program about oa in the Brazilian p	No	Without intervention/comparator	Without intervention/comparator	-
3147	2018	Two-year results of a two-day educational program about oa parqve ii (project arthritis recoverin	No	Without intervention/comparator	Without intervention/comparator	-
3148	2015	Efficacy of action observation pre-operative training in functional recovery after hip and knee pr	No	Without intervention/comparator	Without intervention/comparator	-
3149	2019	Corticosteroid injection versus a physical therapy approach for the management of knee osteoart	No	Without intervention/comparator	Without intervention/comparator	-
3150	2006	Exercise therapy as a treatment in osteoarthritis of the hip: a review of randomized clinical trials	No	Without intervention/comparator	Without intervention/comparator	-
3151	2002	Three surgical methods of anterior cruciate ligament reconstruction were equally effective	No	Other populations/diseases	Other populations/diseases	-
3152	2015	Alpine Skiing With total knee ArthroPlasty (ASWAP): muscular adaptations	No	Without intervention/comparator	Without intervention/comparator	-
3153	2016	The effects of intra-articular glucocorticoids and exercise on pain and synovitis assessed on stati	No	Without intervention/comparator	Without intervention/comparator	-
3154	2016	The effects of intra-articular glucocorticoids and exercise on pain and synovitis assessed on stati	No	Without intervention/comparator	Without intervention/comparator	-
3155	2017	The effects of intra-articular glucocorticoids and exercise on pain and synovitis assessed on stati	No	Without intervention/comparator	Without intervention/comparator	-
3156	2015	Automated Internet-based pain coping skills training to manage osteoarthritis pain: a randomize	No	Without intervention/comparator	Without intervention/comparator	-
3157	2018	Radial subluxation in relation to hand strength and radiographic severity in trapeziometacarpal o	No	Without intervention/comparator	Without intervention/comparator	-
3158	2018	Arthroscopic Surgical Procedures Versus Sham Surgery for Patients With Femoroacetabular Imp	No	Without intervention/comparator	Without intervention/comparator	-
3159	2017	HPR facilitators and barriers for participation in physical activities in juvenile idiopathic arthriti	No	Without intervention/comparator	Without intervention/comparator	-
3160	2018	Estimated peak oxygen uptake and submaximal parameters from a submaximal treadmill test are	No	Without intervention/comparator	Without intervention/comparator	-
3161	2017	Stepped care approach for medial tibiofemoral osteoarthritis (STrEAMline): protocol for a rando	No	Without intervention/comparator	Without intervention/comparator	-
3162	2019	Effect of combined conservative therapies on clinical outcomes in patients with thumb base oste	No	Without intervention/comparator	Without intervention/comparator	-
3163	2020	Is a stepped-care intervention effective in overweight and obese people with medial tibiofemoral	No	Without intervention/comparator	Without intervention/comparator	-
3164	2019	Patient-reported outcomes in patients with a germline BRCA mutation and HER2-negative meta	No	Without intervention/comparator	Without intervention/comparator	-
3165	2019	Animal-assisted intervention improves pain perception in polymedicated geriatric patients with (	No	Without intervention/comparator	Without intervention/comparator	-

**3166**	**2020**	**Low-Load Resistance Training With Blood-Flow Restriction in Relation to Muscle Functic**	**Yes**	**Full**	**Full**	**-**
3167	2017	The effect of an intensive smoking cessation intervention on disease activity in patients with rhe	No	Without intervention/comparator	Without intervention/comparator	-
3168	2010	A comparison of dedicated 1.0 T extremity MRI vs large-bore 1.5 T MRI for semiquantitative w	No	Without intervention/comparator	Without intervention/comparator	-
3169	2018	6-and 12-months follow-up of a randomized controlled trial on land-based exercise therapy in hi	No	Without intervention/comparator	Without intervention/comparator	-
3170	2016	Effects of a multicomponent physical activity behavior change intervention on breast cancer surv	No	Without intervention/comparator	Without intervention/comparator	-
3171	2013	PW01-040-Definition of polymorphism C3435T MDR1 gene in JIA	No	Without intervention/comparator	Without intervention/comparator	-
3172	2020	Rehabilitation with the aid of blood flow restriction training	No	Without intervention/comparator	Without intervention/comparator	-
3173	2020	Is a Combination of Exercise and Dry Needling Effective for Knee OA?	No	Without intervention/comparator	Without intervention/comparator	-
3174	2012	Tai chi for osteoarthritis of the knee-a systematic review and meta-analysis	No	Review/Systematic Review/Meta-Analys	Review/Systematic Review/Meta-Analys	-
3175	2011	Effect of an education programme for patients with osteoarthritis in primary care-a randomized c	No	Without intervention/comparator	Without intervention/comparator	-
3176	2019	Considering surgery for your knee problem? Exercise may work just as well!	No	Without intervention/comparator	Without intervention/comparator	-
3177	2017	The predimed studies: a leap forward in nutrition	No	Without intervention/comparator	Without intervention/comparator	-
3178	2012	Comparison of the effectiveness of isokinetic vs isometric therapeutic exercise in patients with o	No	Without intervention/comparator	Without intervention/comparator	-
3179	2014	Efficacy of exercise intervention as determined by the McKenzie System of Mechanical Diagnos	No	Without intervention/comparator	Without intervention/comparator	-
3180	2014	The effect of posterior and lateral approach on patient-reported outcome measures and physical f	No	Without intervention/comparator	Without intervention/comparator	-
3181	2016	The efficacy of modified direct lateral versus posterior approach on gait function and hip muscle	No	Without intervention/comparator	Without intervention/comparator	-
3182	2017	Patient-reported outcome after total hip arthroplasty: comparison between lateral and posterior ap	No	Without intervention/comparator	Without intervention/comparator	-
3183	2020	Outcomes of a conservative care program for knee osteoarthritis through translation of research i	No	Without intervention/comparator	Without intervention/comparator	-
3184	2019	The Effects of Useful Field of View Training on Brain Activity and Connectivity	No	Without intervention/comparator	Without intervention/comparator	-
3185	2013	Feelings of competence and relatedness during physical activity are related to well-being in rheu	No	Without intervention/comparator	Without intervention/comparator	-
3186	2014	Fostering autonomous motivation, physical activity and cardiorespiratory fitness in rheumatoid a	No	Without intervention/comparator	Without intervention/comparator	-
3187	2015	Measuring the positive psychological well-being of people with rheumatoid arthritis: a cross-sec	No	Without intervention/comparator	Without intervention/comparator	-
3188	2018	The Effect of Adductor Canal Block on Knee Extensor Muscle Strength 6 Weeks After Total Kn	No	Without intervention/comparator	Without intervention/comparator	-
3189	2017	Fracture risk after ulnohumeral arthroplasty: a biomechanical study	No	Without intervention/comparator	Without intervention/comparator	-
3190	2020	Associations of diet quality with knee joint structures, symptoms, and systematic measures in pa	No	Without intervention/comparator	Without intervention/comparator	-
3191	2012	Oral glucosamine sulphate for the prevention of knee osteoarthritis in overweight females; the fi	No	Without intervention/comparator	Without intervention/comparator	-
3192	2015	Prevention of knee osteoarthritis in overweight females: the first preventive randomized controlle	No	Without intervention/comparator	Without intervention/comparator	-
3193	2016	The role of diet and exercise and of glucosamine sulfate in the prevention of knee osteoarthritis:	No	Without intervention/comparator	Without intervention/comparator	-
3194	2019	Inflammatory cytokines mediate the effects of diet and exercise on pain and function in knee ost	No	Without intervention/comparator	Without intervention/comparator	-
3195	2013	Lateral wedges alter mediolateral load distributions at the knee joint in obese individuals	No	Without intervention/comparator	Without intervention/comparator	-
3196	2011	Internet-based outpatient telerehabilitation for patients following total knee arthroplasty: a rando	No	Without intervention/comparator	Without intervention/comparator	-
3197	2016	Efficacy of intra-articular hyaluronic acid injections and exercise-based rehabilitation programm	No	Without intervention/comparator	Without intervention/comparator	-
3198	2019	The effectiveness of narrative writing on the moral distress of intensive care nurses	No	Without intervention/comparator	Without intervention/comparator	-
3199	2018	A theory of planned behavior-based intervention to improve quality of life in patients with knee/	No	Without intervention/comparator	Without intervention/comparator	-
3200	2019	A consensus-based process identifying physical therapy and exercise treatments for patients with	No	Without intervention/comparator	Without intervention/comparator	-
3201	2019	Effects of two different aquatic exercise trainings on cardiopulmonary endurance and emotional	No	Without intervention/comparator	Without intervention/comparator	-
3202	2015	Balneotherapy in the treatment of knee osteoarthritis: a controlled study	No	Without intervention/comparator	Without intervention/comparator	-
3203	2013	Comparison of the efficacy & safety of minocycline versus hydroxychloroquine as an add-on the	No	Without intervention/comparator	Without intervention/comparator	-
3204	2015	Development of new bioassay system measuring inhibitory strength of IL-6/STAT3 signal under	No	Without intervention/comparator	Without intervention/comparator	-
3205	2012	The effects of group cycling on gait and pain-related disability in individuals with mild-to-mode	No	Without intervention/comparator	Without intervention/comparator	-
3206	2019	Application of lateral wedge in knee osteoarthritis for improving pain and quality of life	No	Without intervention/comparator	Without intervention/comparator	-
3207	2020	G-CSF (filgrastim) treatment for amyotrophic lateral sclerosis: protocol for a phase II randomise	No	Without intervention/comparator	Without intervention/comparator	-
3208	2012	Six year outcome excision of the trapezium for trapeziometacarpal joint osteoarthritis: is it impro	No	Without intervention/comparator	Without intervention/comparator	-
3209	2019	Prospective Clinical Trial Comparing Trapezial Denervation With Trapeziectomy for the Surgica	No	Without intervention/comparator	Without intervention/comparator	-

3210	2010	The effect of two exercise programs on various functional outcome measures in patients with ost	No	Without intervention/comparator	Without intervention/comparator	-
3211	2015	Acceptability of a novel physical activity and self-management intervention for managing fatigu	No	Without intervention/comparator	Without intervention/comparator	-
3212	2004	First Clinical Experience with BION Implants for Therapeutic Electrical Stimulation	No	Other populations/diseases	Other populations/diseases	-
3213	2015	Routine initial exercise stress testing for treatment stratification in comprehensive cardiac rehabi	No	Without intervention/comparator	Without intervention/comparator	-
3214	2014	Effects of non weight bearing strength training for knee osteoarthritis	No	Without intervention/comparator	Without intervention/comparator	-
3215	2015	The effect of isokinetic and aerobic exercises on serum interleukin-6 and tumor necrosis factor a	No	Without intervention/comparator	Without intervention/comparator	-
3216	2019	Is a Combination of Exercise and Dry Needling Effective for Knee OA?	No	Without intervention/comparator	Without intervention/comparator	-
3217	2020	Partial Versus Total Trapeziectomy With Interposition Arthroplasty for Trapeziometacarpal Oste	No	Without intervention/comparator	Without intervention/comparator	-
3218	2015	Exploring the effect of space and place on response to exercise therapy for knee and hip pain--a	No	Without intervention/comparator	Without intervention/comparator	-
3219	2016	Pain trajectory and exercise-induced pain flares during 8 weeks of neuromuscular exercise in ind	No	Without intervention/comparator	Without intervention/comparator	-
3220	2008	A multidisciplinary assessment and intervention for patients awaiting total hip replacement to in	No	Other populations/diseases	Other populations/diseases	-
3221	2015	The effects of high intensity interval training in women with rheumatic disease: a pilot study	No	Without intervention/comparator	Without intervention/comparator	-
3222	2013	Muscle strength, physical fitness and well-being in children and adolescents with juvenile idiopa	No	Without intervention/comparator	Without intervention/comparator	-
3223	2013	PReS-FINAL-2049: bone health, muscle strength, activity	No	Without intervention/comparator	Without intervention/comparator	-
3224	2017	A comparative study on effects of combined applications of Russian current and various strength	No	Without intervention/comparator	Without intervention/comparator	-
3225	2018	An easy and safe training method for trunk function improves mobility in total knee arthroplasty	No	Without intervention/comparator	Without intervention/comparator	-
3226	2016	Balneotherapy and rheumatoid arthritis: a randomized control trial	No	Without intervention/comparator	Without intervention/comparator	-
3227	2015	The Effect of Multifactorial Intervention Programs on Health Behavior and Symptom Control Ar	No	Without intervention/comparator	Without intervention/comparator	-
3228	2018	Effect photobiomodulation in addition with a balance protocol in functionality patients with adv	No	Without intervention/comparator	Without intervention/comparator	-
3229	2017	HPR a better way to decrease knee swelling in patients with knee osteoarthritis: intermittent pne	No	Without intervention/comparator	Without intervention/comparator	-
3230	2019	A Better Way to Decrease Knee Swelling in Patients with Knee Osteoarthritis: a Single-Blind Ra	No	Without intervention/comparator	Without intervention/comparator	-
3231	2016	Prospective Randomized Study of Objective and Subjective Clinical Results Between Double-Bu	No	Without intervention/comparator	Without intervention/comparator	-
3232	2019	Pedaling-Based Protocol Superior to a 10-Exercise, Non-Pedaling Protocol for Postoperative Rel	No	Without intervention/comparator	Without intervention/comparator	-
3233	2018	Addressing osteoarthritis pain among older adult women through aquatic therapy: a new look at	No	Without intervention/comparator	Without intervention/comparator	-
3234	2018	External Fixation Versus Volar Locking Plate for Unstable Dorsally Displaced Distal Radius Fra	No	Without intervention/comparator	Without intervention/comparator	-
3235	2012	Clodronate and hydroxychloroquine in erosive osteoarthritis: a 24-month open randomized pilot	No	Without intervention/comparator	Without intervention/comparator	-
3236	2017	Intramuscular clodronate in erosive osteoarthritis of the hand is effective on pain and reduces ser	No	Without intervention/comparator	Without intervention/comparator	-
3237	2015	The effects of a six-week physiotherapist-led exercise and education intervention in patients with	No	Without intervention/comparator	Without intervention/comparator	-
3238	2016	Significant improvements in pain after a six-week physiotherapist-led exercise and education int	No	Without intervention/comparator	Without intervention/comparator	-
3239	2012	Effect of high-speed power training on muscle performance, function, and pain in older adults w	No	Without intervention/comparator	Without intervention/comparator	-
3240	2012	Effect of high-speed power training on muscle performance, function, and pain in older adults w	No	Without intervention/comparator	Without intervention/comparator	-
3241	2018	Effects of dry needling in an exercise program for older adults with knee osteoarthritis: a pilot cl	No	Without intervention/comparator	Without intervention/comparator	-
3242	2011	Experimental knee joint pain during strength training increases muscle strength gain in healthy s	No	Without intervention/comparator	Without intervention/comparator	-
3243	2016	Does the addition of hip strengthening exercises improve outcomes following total knee arthropl	No	Without intervention/comparator	Without intervention/comparator	-
3244	2019	Incorporating hip abductor strengthening exercises into a rehabilitation program did not improve	No	Without intervention/comparator	Without intervention/comparator	-
3245	2009	A comparison of Kneipp hydrotherapy with conventional physiotherapy in the treatment of osteo	No	Without intervention/comparator	Without intervention/comparator	-
3246	2016	Sex Variations in the Effects of Arthritis and Activity Limitation on First Heart Disease Event O	No	Without intervention/comparator	Without intervention/comparator	-
3247	2011	Improving physical activity and function in overweight and obese older adults with osteoarthritis	No	Without intervention/comparator	Without intervention/comparator	-
3248	2011	Disease-drug-drug interaction involving tocilizumab and Yesvastatin in patients with rheumatoi	No	Without intervention/comparator	Without intervention/comparator	-
3249	2014	Assessment of combination therapy responses in early rheumatoid arthritis defined by presence o	No	Without intervention/comparator	Without intervention/comparator	-
3250	2014	Assessment of combination therapy responses in early rheumatoid arthritis defined by presence o	No	Without intervention/comparator	Without intervention/comparator	-
3251	2014	Assessment of combination therapy responses in early rheumatoid arthritis defined by presence o	No	Without intervention/comparator	Without intervention/comparator	-
3252	2016	The impact of intensive treatment and remission on health-related quality of life in rheumatoid a	No	Without intervention/comparator	Without intervention/comparator	-
3253	2019	An integrated motivational interviewing and cognitive-behavioural intervention promoting physi	No	Without intervention/comparator	Without intervention/comparator	-

3254	2019	Bros Will Be Bros? The Effect of Fraternity Membership on Perceived Culpability for Sexual As	No	Without intervention/comparator	Without intervention/comparator	-
3255	2016	Evaluation of the effect of acupuncture on hand pain, functional deficits and health-related quali	No	Without intervention/comparator	Without intervention/comparator	-
3256	2019	Effectiveness of Acupuncture on Pain, Functional Disability, and Quality of Life in Rheumatoid	No	Without intervention/comparator	Without intervention/comparator	-
3257	2019	Uphill treadmill walking plus physical therapy versus physical therapy alone in the management	No	Without intervention/comparator	Without intervention/comparator	-
3258	2018	The efficacy of electromyographic biofeedback on pain, function and maximal thickness of vastu	No	Without intervention/comparator	Without intervention/comparator	-
3259	2016	Impact of a behavioral weight loss intervention on comorbidities in overweight and obese breast	No	Without intervention/comparator	Without intervention/comparator	-
**3260**	**2015**	**Efficacy of Blood Flow-Restricted Low-Load Resistance Training For Quadriceps Strengt**	**Yes**	**Full**	**Full**	**-**
3261	2010	Intensive gait training improves pain and mobility in older adults with symptomatic knee osteoar	No	Without intervention/comparator	Without intervention/comparator	-
3262	2013	Vibration platform training in women at risk for symptomatic knee osteoarthritis	No	Without intervention/comparator	Without intervention/comparator	-
**3263**	**2015**	**Efficacy of Blood Flow-Restricted, Low-Load Resistance Training in Women with Risk Fa**	**Yes**	**Full**	**Full**	**-**
3264	2015	Intensive Gait Training for Older Adults with Symptomatic Knee Osteoarthritis	No	Without intervention/comparator	Without intervention/comparator	-
3265	2016	A randomised trial comparing the efficacy and safety of topical ketoprofen in Transfersome(Â®)	No	Without intervention/comparator	Without intervention/comparator	-
3266	2012	Efficacy and safety of alogliptin added to sulfonylurea in Japanese patients with type 2 diabetes:	No	Without intervention/comparator	Without intervention/comparator	-
3267	2010	Assessing physical activity in persons with rheumatoid arthritis using accelerometry	No	Without intervention/comparator	Without intervention/comparator	-
3268	2018	Targeted gluteal exercise versus sham exercise on self-reported physical function for people with	No	Without intervention/comparator	Without intervention/comparator	-
3269	2014	Comparison of a 12-week partly supervised exercise programme with a self-administered exercis	No	Without intervention/comparator	Without intervention/comparator	-
3270	2015	Comparable effect of partly supervised and self-administered exercise programme in early rheum	No	Without intervention/comparator	Without intervention/comparator	-
3271	2016	Effects of dextrose prolotherapy in the treatment of patients with knee osteoarthritis: a randomiz	No	Without intervention/comparator	Without intervention/comparator	-
3272	2020	The Effects of Dextrose Prolotherapy in Symptomatic Knee Osteoarthritis: a Randomized Contro	No	Without intervention/comparator	Without intervention/comparator	-
3273	2009	Cost-effectiveness of exercise and diet in overweight and obese adults with knee osteoarthritis	No	Without intervention/comparator	Without intervention/comparator	-
3274	2017	Comparison of functional training and strength training in improving knee extension lag after fir	No	Without intervention/comparator	Without intervention/comparator	-
3275	2019	Emotion regulation and intimacy quality: the consequences of emotional integration, emotional c	No	Without intervention/comparator	Without intervention/comparator	-
3276	2018	Efficacy of Bingocize: a Game-Centered Mobile Application to Improve Physical and Cognitive	No	Other populations/diseases	Other populations/diseases	-
3277	2017	Effects of supervised structured aerobic exercise training program on high and low density lipop	No	Without intervention/comparator	Without intervention/comparator	-
3278	2019	Effects of infrared radiation and shortwave diathermy on the patients with chronic low back pair	No	Without intervention/comparator	Without intervention/comparator	-
3279	2017	The Association of Vibratory Perception and Muscle Strength With the Incidence and Worsening	No	Without intervention/comparator	Without intervention/comparator	-
3280	2019	Prospective randomized controlled study on improving sleep quality and impact of zolpidem afte	No	Without intervention/comparator	Without intervention/comparator	-
3281	2015	A randomized trial of vitamin D3 in aromatase inhibitor-associated musculoskeletal symptoms	No	Without intervention/comparator	Without intervention/comparator	-
3282	2016	Effects of aerobic exercise on hematologic indices of women with rheumatoid arthritis: a random	No	Without intervention/comparator	Without intervention/comparator	-
3283	2014	Outcomes and complications of fractures of distal radius (AO type B and C): volar plating versu	No	Without intervention/comparator	Without intervention/comparator	-
3284	2002	Nonpharmacologic management of osteoarthritis	No	Without intervention/comparator	Without intervention/comparator	-
3285	2020	[Chronic pain syndrome in diseases of periarticular tissues]	No	Without intervention/comparator	Without intervention/comparator	-
3286	2019	Effect of a Ketogenic Diet on Submaximal Exercise Capacity and Efficiency in Runners	No	Without intervention/comparator	Without intervention/comparator	-
3287	2017	Is there a synergistic role for vitamin K and vitamin D in lower extremity function related to kne	No	Without intervention/comparator	Without intervention/comparator	-
3288	2010	The effect of randomization to weight loss on total mortality in older overweight and obese adult	No	Without intervention/comparator	Without intervention/comparator	-
3289	2014	Postoperative shoulder function after different types of neck dissection in head and neck cancer	No	Without intervention/comparator	Without intervention/comparator	-
3290	2019	Innovative Exercise as an Intervention for Older Adults with Knee Osteoarthritis: a Pilot Feasibil	No	Without intervention/comparator	Without intervention/comparator	-
3291	2011	Assessment of clinical effect of therapy combining disease with syndrome on rheumatoid arthriti	No	Without intervention/comparator	Without intervention/comparator	-
3292	2017	Juvenile idiopathic arthritis in relation to perinatal and maternal characteristics	No	Without intervention/comparator	Without intervention/comparator	-
3293	2019	Effect of tart cherry on aromatase inhibitor-induced arthralgia (AIA) in nonmetastatic hormone-p	No	Without intervention/comparator	Without intervention/comparator	-
3294	2020	Meniscus Injuries: A Review of Rehabilitation and Return to Play	No	Review/Systematic Review/Meta-Analys	Review/Systematic Review/Meta-Analys	-
3295	2018	Immediate Effects of Transcutaneous Electrical Nerve Stimulation on Pain and Physical Perform	No	Without intervention/comparator	Without intervention/comparator	-
3296	2015	The exercise therapy decreases the serum interleukin-6 levels in patients with knee OA	No	Without intervention/comparator	Without intervention/comparator	-
3297	2015	The beneficial effects of Tai Chi exercise on endothelial function and arterial stiffness in elderly	No	Without intervention/comparator	Without intervention/comparator	-

3298	2017	Effect of Therapeutic Exercise Programme in Adults with Early Rheumatoid Arthritis	No	Without intervention/comparator	Without intervention/comparator	-
3299	2008	The feasibility of a randomized trial using a progressive exercise program in patients with severe	No	Without intervention/comparator	Without intervention/comparator	-
3300	2010	Effectiveness of different quadriceps strengthening protocols in improvement of extensor lag in	No	Without intervention/comparator	Without intervention/comparator	-
3301	2015	Rehabilitation of the patients with osteoarthritis	No	Without intervention/comparator	Without intervention/comparator	-
3302	2012	Effect of whole body vibration training on physical performance among institutionalized older p	No	Without intervention/comparator	Without intervention/comparator	-
3303	2013	Arthroscopic partial meniscectomy versus sham surgery for a degenerative meniscal tear	No	Without intervention/comparator	Without intervention/comparator	-
3304	2017	Arthroscopic partial meniscectomy versus placebo surgery for a degenerative meniscus tear: a 2-	No	Without intervention/comparator	Without intervention/comparator	-
3305	2018	Arthroscopic partial meniscectomy versus placebo surgery for a degenerative meniscus tear: a 2-	No	Without intervention/comparator	Without intervention/comparator	-
3306	2011	Comparison of two carpometacarpal stabilizing splints for individuals with thumb osteoarthritis	No	Without intervention/comparator	Without intervention/comparator	-
3307	2015	Resistance training in patients with psoriatic arthritis improves function, disease activity and qua	No	Without intervention/comparator	Without intervention/comparator	-
3308	2017	HPR comparison of the effectiveness of functional and night splint for rhizarthrosis: one-year fol	No	Without intervention/comparator	Without intervention/comparator	-
3309	2018	Effectiveness of night-time orthoses in the pain for women with hand osteoarthritis: randomized	No	Without intervention/comparator	Without intervention/comparator	-
3310	2013	Comparison of the underwater treadmill, land-based treadmill, and exercise cycle on patient repo	No	Without intervention/comparator	Without intervention/comparator	-
3311	2012	Functional performance and inflammatory cytokines after squat exercises and whole-body vibrat	No	Without intervention/comparator	Without intervention/comparator	-
3312	2019	Whole body vibration training on muscle strength and brain-derived neurotrophic factor levels in	No	Without intervention/comparator	Without intervention/comparator	-
3313	2012	Functional performance and inflammatory cytokines after squat exercises and whole-body vibrat	No	Without intervention/comparator	Without intervention/comparator	-
3314	2020	Osteoarthritis Preoperative Package for care of Orthotics, Rehabilitation, Topical and oral agent	No	Without intervention/comparator	Without intervention/comparator	-
3315	2019	Managing behavioral and psychological symptoms in Chinese elderly with dementia via group-b	No	Without intervention/comparator	Without intervention/comparator	-
3316	2015	Application of yoga therapy to psychosamatic disorders	No	Without intervention/comparator	Without intervention/comparator	-
3317	2016	Effectiveness of hip abductor strengthening on health status, strength, endurance and six minute	No	Without intervention/comparator	Without intervention/comparator	-
3318	2012	Water exercises do not improve body composition in women with rheumatoid arthritis: the hydra	No	Without intervention/comparator	Without intervention/comparator	-
3319	2017	Effectiveness of Aquatic Exercises in Women With Rheumatoid Arthritis: a Randomized, Contro	No	Without intervention/comparator	Without intervention/comparator	-
3320	2018	Clinic-Based Patellar Mobilization Therapy for Knee Osteoarthritis: a Randomized Clinical Tria	No	Without intervention/comparator	Without intervention/comparator	-
3321	2018	Clinical effectiveness of patella mobilisation therapy versus a waiting list control for knee osteoa	No	Without intervention/comparator	Without intervention/comparator	-
3322	2010	Physical-activity coaching and health status in rheumatoid arthritis: a person-oriented approach	No	Without intervention/comparator	Without intervention/comparator	-
3323	2011	Physical activity coaching of patients with rheumatoid arthritis in everyday practice: a long-term	No	Without intervention/comparator	Without intervention/comparator	-
3324	2016	Efficacy of Preoperative Progressive Resistance Training on Postoperative Outcomes in Patients	No	Without intervention/comparator	Without intervention/comparator	-
3325	2018	No Exacerbation of Knee Joint Pain and Effusion Following Preoperative Progressive Resistance	No	Other populations/diseases	Other populations/diseases	-
3326	2020	Efficacy of preoperative progressive resistance training in patients undergoing total knee arthrop	No	Without intervention/comparator	Without intervention/comparator	-
3327	2012	Total knee replacement plus physical and medical therapy or treatment with physical and medica	No	Without intervention/comparator	Without intervention/comparator	-
3328	2014	Association of knee confidence with pain, knee instability, muscle strength, and dynamic varus-	No	Without intervention/comparator	Without intervention/comparator	-
3329	2015	A Randomized, Controlled Trial of Total Knee Replacement	No	Without intervention/comparator	Without intervention/comparator	-
3330	2015	The combined efficacy of multimodal non-surgical treatment on pain and sensitization in patient	No	Without intervention/comparator	Without intervention/comparator	-
3331	2015	The efficacy of 12 weeks non-surgical treatment for patients not eligible for total knee replaceme	No	Without intervention/comparator	Without intervention/comparator	-
3332	2016	The effects of total knee replacement and non-surgical treatment on pain sensitization and clinic	No	Without intervention/comparator	Without intervention/comparator	-
3333	2016	The efficacy of non-surgical treatment on pain and sensitization in patients with knee osteoarthri	No	Without intervention/comparator	Without intervention/comparator	-
3334	2016	The two-year efficacy of 12-weeks non-surgical treatment for patients not eligible for total knee	No	Without intervention/comparator	Without intervention/comparator	-
3335	2016	Total knee replacement followed by a non-surgical treatment program reduce localized and sprea	No	Without intervention/comparator	Without intervention/comparator	-
3336	2017	Study protocol for a randomised controlled trial of meniscal surgery compared with exercise and	No	Other populations/diseases	Other populations/diseases	-
3337	2017	Two year outcome from two parallel randomized trials on total knee replacement and non-surgic	No	Without intervention/comparator	Without intervention/comparator	-
3338	2018	Total knee replacement and non-surgical treatment of knee osteoarthritis: 2-year outcome from t	No	Without intervention/comparator	Without intervention/comparator	-
3339	2019	2-year cost-effectiveness of total knee replacement: results from the first randomized trial on tota	No	Without intervention/comparator	Without intervention/comparator	-
3340	2020	Cost-effectiveness of 12 weeks of supervised treatment compared to written advice in patients w	No	Without intervention/comparator	Without intervention/comparator	-
3341	2020	Cost-effectiveness of total knee replacement in addition to non-surgical treatment: a 2-year outco	No	Without intervention/comparator	Without intervention/comparator	-

3342	2019	The impact of Co-actors on cognitive load: when the mere presence of others makes learning mo	No	Without intervention/comparator	Without intervention/comparator	-
3343	2008	Mind on arthritis study	No	Without intervention/comparator	Without intervention/comparator	-
3344	2014	Muscle force steadiness in older adults before and after total knee arthroplasty	No	Without intervention/comparator	Without intervention/comparator	-
3345	2018	A pilot randomized trial of meniscal allograft transplantation versus personalized physiotherapy	No	Without intervention/comparator	Without intervention/comparator	-
3346	2020	Comparing of reliability, construct validity and responsiveness of the IPAQ-SF and pase in adult	No	Without intervention/comparator	Without intervention/comparator	-
3347	2019	A Randomized Study of Exercise and Fitness Trackers in Obese Patients After Total Knee Arthro	No	Without intervention/comparator	Without intervention/comparator	-
3348	2014	Fit and Strong! Plus: design of a comparative effectiveness evaluation of a weight management p	No	Without intervention/comparator	Without intervention/comparator	-
3349	2010	Randomized trial assessing the impact of a musculoskeletal intervention for pain before participa	No	Without intervention/comparator	Without intervention/comparator	-
3350	2015	The effects of a community aquatic exercise programme for chinese people with knee osteoarthri	No	Without intervention/comparator	Without intervention/comparator	-
3351	2018	Preoperative physical therapy education reduces time to meet functional milestones after total jo	No	Without intervention/comparator	Without intervention/comparator	-
3352	2015	A low-fat yoghurt supplemented with a rooster comb extract on muscle joint function in adults w	No	Without intervention/comparator	Without intervention/comparator	-
3353	2020	Knee Arthroscopic Surgery in Middle-Aged Patients With Meniscal Symptoms: a 5-Year Follow	No	Without intervention/comparator	Without intervention/comparator	-
3354	2009	Effects of Tai Chi or Self-help Program on Balance, Flexibility, Oxygen Consumption, and Musc	No	Without intervention/comparator	Without intervention/comparator	-
3355	2010	A randomized study of the effects of t'ai chi on muscle strength, bone mineral density, and fear o	No	Without intervention/comparator	Without intervention/comparator	-
3356	2010	Severe knee osteoarthritis: a study of combined acupuncture and physiotherapy vs home exercise	No	Without intervention/comparator	Without intervention/comparator	-
3357	2012	Supervised exercise plus acupuncture for moderate to severe knee osteoarthritis: a small randomi	No	Without intervention/comparator	Without intervention/comparator	-
3358	2019	Effect of two behavioural 'nudging' interventions on management decisions for low back pain: a	No	Without intervention/comparator	Without intervention/comparator	-
3359	2019	Randomized, double-blind, placebo-controlled phase ii clinical trial on the use of uncaria toment	No	Without intervention/comparator	Without intervention/comparator	-
3360	2016	Intra-Articular Corticosteroids in Addition to Exercise for Reducing Pain Sensitivity in Knee Os	No	Without intervention/comparator	Without intervention/comparator	-
3361	2019	Intermittent Claudication in Physiotherapists' Practice	No	Other populations/diseases	Other populations/diseases	-
3362	2016	A randomized controlled trial of rheumatoid arthritis risk disclosure personalized to genetics, aut	No	Without intervention/comparator	Without intervention/comparator	-
3363	2018	Disclosure of Personalized Rheumatoid Arthritis Risk Using Genetics, Biomarkers, and Lifestyle	No	Without intervention/comparator	Without intervention/comparator	-
3364	2015	The effect of a bone tunnel during ligament reconstruction for trapeziometacarpal osteoarthritis:	No	Without intervention/comparator	Without intervention/comparator	-
3365	2019	Educational outreach visits to improve knee osteoarthritis management in primary care	No	Without intervention/comparator	Without intervention/comparator	-
3366	2015	Effectiveness of Triamcinolone Hexacetonide Intraarticular Injection in Interphalangeal Joints: a	No	Without intervention/comparator	Without intervention/comparator	-
3367	2015	Blood flow restriction training and the exercise pressor reflex: a call for concern	No	Other populations/diseases	Other populations/diseases	-
3368	2013	Task-oriented training with computer gaming in people with rheumatoid arthritisor osteoarthritis	No	Without intervention/comparator	Without intervention/comparator	-
3369	2015	Expanding the horizon-platelet rich plasma in rheumatology	No	Without intervention/comparator	Without intervention/comparator	-
3370	2015	Does yoga improve pain, stiffness and physical disability in knee osteoarthritis?-A randomize co	No	Without intervention/comparator	Without intervention/comparator	-
3371	2011	Functional outcome following intramedullary nailing of the femur: a prospective randomized con	No	Other populations/diseases	Other populations/diseases	-
3372	2020	Effect of exercise on knee joint contact forces in people following medial partial meniscectomy:	No	Other populations/diseases	Other populations/diseases	-
3373	2018	The impact of a physiotherapy tele-rehabilitation program on children with juvenile idiopathic ar	No	Other populations/diseases	Other populations/diseases	-
3374	2012	Ã‰valuation d'un protocole spÃ©cifique de kinÃ©sithÃ©rapie ambulatoire de la coxarthrose	No	Without intervention/comparator	Without intervention/comparator	-
3375	2010	Whole body vibration compared to conventional physiotherapy in patients with gonarthrosis: a p	No	Without intervention/comparator	Without intervention/comparator	-
3376	2012	Feasibility and efficacy of an 8-week progressive home-based strengthening exercise program in	No	Without intervention/comparator	Without intervention/comparator	-
3377	2017	Exercise therapy in patients with hip osteoarthritis: effect on hip muscle strength and safety aspe	No	Without intervention/comparator	Without intervention/comparator	-
3378	2010	The feasibility of a progressive twelve-week neuromuscular and strength training program in mid	No	Other populations/diseases	Other populations/diseases	-
3379	2015	Effect of exercise therapy compared with arthroscopic surgery on knee muscle strength and func	No	Other populations/diseases	Other populations/diseases	-
3380	2012	A family based pedometer walking program in an adolescent population with juvenile idiopathic	No	Other populations/diseases	Other populations/diseases	-
3381	2016	The efficacy and safety of a combination of glucosamine hydrochloride, chondroitin sulfate and	No	Without intervention/comparator	Without intervention/comparator	-
3382	2001	Muscle strength, pain and disability in patients with osteoarthritis	No	Without intervention/comparator	Without intervention/comparator	-
3383	2002	Avoidance of activity and disability in patients with osteoarthritis of the knee: the mediating role	No	Without intervention/comparator	Without intervention/comparator	-
3384	2018	Gehtraining verbessert mit und ohne <> die Gehstrecke bei Patienten mit einer PAVK	No	Other populations/diseases	Other populations/diseases	-
3385	2011	Comparison of self-reported knee injury and osteoarthritis outcome score to performance measur	No	Without intervention/comparator	Without intervention/comparator	-

3386	2012	Minimally invasive total knee arthroplasty improves early knee strength but not functional perfo	No	Without intervention/comparator	Without intervention/comparator	-
3387	2015	Cementless or cemented fixation in proximal interphalangeal joint surface replacement arthropla	No	Without intervention/comparator	Without intervention/comparator	-
3388	2017	A randomized controlled trial (RCT) of an internet-based self-management program for adolesce	No	Without intervention/comparator	Without intervention/comparator	-
3389	2017	An internet-based self-management program for adolescents with juvenile idiopathic arthritis - A	No	Without intervention/comparator	Without intervention/comparator	-
3390	2018	Functional consultation and exercises improve grip strength in osteoarthritis of the hand - a rand	No	Without intervention/comparator	Without intervention/comparator	-
3391	2001	Effect of manual therapy and exercise on pain, stiffness and function in persons with knee osteo	No	Without intervention/comparator	Without intervention/comparator	-
3392	2011	The effects of strength and endurance training in patients with rheumatoid arthritis	No	Without intervention/comparator	Without intervention/comparator	-
3393	2015	Impact of body weight loss on the clinical manifestations of knee osteoarthritis and metabolic sy	No	Without intervention/comparator	Without intervention/comparator	-
3394	2017	Effect of pharmacological and non-pharmacological therapy of obesity on the clinical manifestat	No	Without intervention/comparator	Without intervention/comparator	-
3395	2018	Pharmacotherapy of obesity in patients with knee osteoarthritis and metabolic syndrome	No	Without intervention/comparator	Without intervention/comparator	-
3396	2018	The role of mtor gene expression, apoptosis and inflammation in obese patients with knee osteoa	No	Without intervention/comparator	Without intervention/comparator	-
3397	2015	A phase 3, open-label, randomized trial to evaluate the safety and efficacy of levofloxacin inhala	No	Without intervention/comparator	Without intervention/comparator	-
3398	2011	Determinants of limitations in activities in patients with osteoarthritis of hands	No	Without intervention/comparator	Without intervention/comparator	-
3399	2011	A multidisciplinary and multidimensional program for hand osteoarthritis is not effective: results	No	Without intervention/comparator	Without intervention/comparator	-
3400	2013	A multidisciplinary and multidimensional program for hand osteoarthritis is not effective: results	No	Without intervention/comparator	Without intervention/comparator	-
3401	2014	In patients with hand osteoarthritis there is no evidence that a booster session after multidiscipli	No	Without intervention/comparator	Without intervention/comparator	-
3402	2011	The effects of traditional strengthening exercises versus functional task training on pain, strengtl	No	Without intervention/comparator	Without intervention/comparator	-
3403	2012	A prospective, multi-center, randomised trial to evaluate the efficacy of a cryopneumatic device	No	Without intervention/comparator	Without intervention/comparator	-
3404	2013	Effect of taping and closed kinetic chain versus traditional approach in osteoarthritis knee	No	Without intervention/comparator	Without intervention/comparator	-
3405	2019	Slow speed resistance exercise training in children with polyarticular juvenile idiopathic arthritis	No	Other populations/diseases	Other populations/diseases	-
3406	2018	The TeMPO trial (treatment of meniscal tears in osteoarthritis): rationale and design features for	No	Without intervention/comparator	Without intervention/comparator	-
3407	2015	Compound Xuanju Capsule combined with intra-articular injection of tumor necrosis factor anta	No	Without intervention/comparator	Without intervention/comparator	-
3408	2019	Experimental Study on the Prevention of Posttraumatic Osteoarthritis in the Rabbit Knee Using a	No	Without intervention/comparator	Without intervention/comparator	-
3409	2012	Double-bundle versus single-bundle anterior cruciate ligament reconstruction: a prospective ranc	No	Without intervention/comparator	Without intervention/comparator	-
3410	2008	Risk factors of musculoskeletal ambulation disability symptom complex (MADS). Frequent falls	No	Other populations/diseases	Other populations/diseases	-
3411	2019	Home exercise therapy to improve muscle strength and joint flexibility effectively treats pre-radi	No	Without intervention/comparator	Without intervention/comparator	-
3412	2012	Reliability and validity of the Physical Activity Scale for the Elderly (PASE) in patients with hip	No	Without intervention/comparator	Without intervention/comparator	-
3413	2013	Exercise therapy may postpone total hip replacement surgery in patients with hip osteoarthritis: a	No	Without intervention/comparator	Without intervention/comparator	-
3414	2015	Exercise therapy may postpone total hip replacement surgery in patients with hip osteoarthritis: a	No	Without intervention/comparator	Without intervention/comparator	-
3415	2016	Long-Term Effect of Exercise Therapy and Patient Education on Impairments and Activity Limi	No	Without intervention/comparator	Without intervention/comparator	-
3416	2010	Long term effect of a supervised exercise program and patient education for patients with hip os	No	Without intervention/comparator	Without intervention/comparator	-
3417	2013	Physical performance and clinical outcomes after exercise therapy and patient education in patie	No	Without intervention/comparator	Without intervention/comparator	-
3418	2019	Better before-better after: efficacy of prehabilitation for older patients with osteoarthritis awaitin	No	Without intervention/comparator	Without intervention/comparator	-
3419	2011	Prehabilitation before total knee arthroplasty increases strength and function in older adults with	No	Without intervention/comparator	Without intervention/comparator	-
3420	2018	Effectiveness of aquatic exercises compared to patient-education on health status in individuals	No	Without intervention/comparator	Without intervention/comparator	-
3421	2011	A novel technology to decrease knee pain and joint load in early-onset knee osteoarthritis	No	Without intervention/comparator	Without intervention/comparator	-
3422	2013	Lower body positive pressure: an emerging technology in the battle against knee osteoarthritis?	No	Without intervention/comparator	Without intervention/comparator	-
3423	2017	Dynamic Balance Training Improves Physical Function in Individuals With Knee Osteoarthritis:	No	Without intervention/comparator	Without intervention/comparator	-
3424	2016	T1rho relaxation mapping with MRI as a measure of the therapeutic effect of conservative treatn	No	Without intervention/comparator	Without intervention/comparator	-
3425	2018	Effect of amlexin (acacia catechu & morus alba) on redox balance and subjective pain in healthy	No	Other populations/diseases	Other populations/diseases	-
3426	2018	Effect of Univestin (Scutellaria baicalensis root & Acacia catechu heartwood) on post-exercise n	No	Other populations/diseases	Other populations/diseases	-
3427	2018	The efficacy of land versus water exercise program on body composition in obese patients with k	No	Without intervention/comparator	Without intervention/comparator	-
3428	2018	Aquatic versus land-based exercise for cardiorespiratory endurance and quality of life in obese p	No	Without intervention/comparator	Without intervention/comparator	-
3429	2014	Cost-utility of exercise therapy added to general practitioners' care versus general practitioners' c	No	Without intervention/comparator	Without intervention/comparator	-

3430	2016	Cost-utility of exercise therapy in patients with hip osteoarthritis in primary care	No	Without intervention/comparator	Without intervention/comparator	-
3431	2018	Effect of Continuous Compression Stimulation on Pressure-Pain Threshold and Muscle Spasms	No	Without intervention/comparator	Without intervention/comparator	-
3432	2019	Effect of an intensive functional rehabilitation program on the recovery of activities of daily livi	No	Without intervention/comparator	Without intervention/comparator	-
3433	2017	Improvement of walking ability during postoperative rehabilitation with the hybrid assistive limb	No	Without intervention/comparator	Without intervention/comparator	-
3434	2016	Biomarker exposure-response relationships as the basis for rational dose selection: lessons from a	No	Other populations/diseases	Other populations/diseases	-
3435	2011	Functional reconstruction of ischemic contracture in the lower limb	No	Other populations/diseases	Other populations/diseases	-
3436	2013	The efficacy of land-based home exercise program in patients with juvenile idiopathic arthritis: a	No	Other populations/diseases	Other populations/diseases	-
3437	2019	Leap Motion Controllerâbased training for upper extremity rehabilitation in children and adolesc	No	Other populations/diseases	Other populations/diseases	-
3438	2003	Comparison of low dose methotrexate and combination therapy with methotrexate and sulphasal	No	Without intervention/comparator	Without intervention/comparator	-
3439	2012	Oral administration of polymer hyaluronic acid alleviates symptoms of knee osteoarthritis: a dou	No	Without intervention/comparator	Without intervention/comparator	-
3440	2009	Patellar tendon versus hamstring tendon autografts for anterior cruciate ligament reconstruction:	No	Other populations/diseases	Other populations/diseases	-
3441	2013	The effect of circuit training in a modified constraint induced movement therapy program	No	Other populations/diseases	Other populations/diseases	-
3442	2019	Cardiovascular Safety During Treatment With Baricitinib in Rheumatoid Arthritis	No	Without intervention/comparator	Without intervention/comparator	-
3443	2018	Cognitive Mediators of Change in Physical Functioning in Response to a Multifaceted Interventi	No	Without intervention/comparator	Without intervention/comparator	-
3444	2009	Correlation between range of motion and implant fracture: a 5 year follow-up of 72 joints in 18 p	No	Other populations/diseases	Other populations/diseases	-
3445	2015	The Comparison of Local Infiltration Analgesia versus Adductor canal block on Postoperative pa	No	Without intervention/comparator	Without intervention/comparator	-
3446	2016	Effects of Progressive Resistance, Non-Weight Bearing Exercise Program and Participatory Ergo	No	Without intervention/comparator	Without intervention/comparator	-
3447	2017	Comparisons between effects of hydrotherapy and land-based exercise on mobility and quality o	No	Without intervention/comparator	Without intervention/comparator	-
3448	2017	Effect of elastic band exercise on muscle strength and physical function in severe knee osteoarth	No	Without intervention/comparator	Without intervention/comparator	-
3449	2017	Effects of Leg Lifting Training Using a Lower Body Position Visual Feedback Device on Balanc	No	Without intervention/comparator	Without intervention/comparator	-
3450	2017	The efficacy of Underwater treadmill exercise for pain relief and functional improvement in obes	No	Without intervention/comparator	Without intervention/comparator	-
3451	2019	Effects of oral coenzyme Q10 supplementation and ischemic preconditioning on tourniquet-indu	No	Without intervention/comparator	Without intervention/comparator	-
3452	2019	Efficacy of fluoroscopic and ultrasound guided genicular nerve ablation for chronic knee osteoar	No	Without intervention/comparator	Without intervention/comparator	-
3453	2019	Fixed- and Mobile-bearing Medial UKA provided Yesilar immediate to early postoperative outc	No	Without intervention/comparator	Without intervention/comparator	-
3454	2019	The efficacy and safety of the pneumatic partial weight support treadmill compared to underwate	No	Without intervention/comparator	Without intervention/comparator	-
3455	2016	Comparison of center-based and home-based exercises on rheumatoid arthritis patients experienc	No	Without intervention/comparator	Without intervention/comparator	-
3456	2014	Effectiveness of exercise therapy added to general practitioners' care versus general practitioners	No	Without intervention/comparator	Without intervention/comparator	-
3457	2018	Daily pain measurements and retrospective pain measurements in hip osteoarthrtitis patients with	No	Without intervention/comparator	Without intervention/comparator	-
3458	2016	Effectiveness of exercise therapy added to general practitioner care in patients with hip osteoarth	No	Without intervention/comparator	Without intervention/comparator	-
3459	2020	Does eccentric-concentric resistance training improve early functional outcomes compared to co	No	Without intervention/comparator	Without intervention/comparator	-
3460	2013	Short-term aerobic exercise attenuate osteoporosis in postmenopausal women: changes of inflam	No	Other populations/diseases	Other populations/diseases	-
3461	2011	Effects of impairment-based exercise on performance of specific self-reported functional tasks in	No	Without intervention/comparator	Without intervention/comparator	-
3462	2017	Blood flow restriction training after knee arthroscopy: A randomized controlled pilot study	No	Other populations/diseases	Other populations/diseases	-
3463	2019	A retrospective observational study of glucosamine sulfate in addition to conventional therapy in	No	Without intervention/comparator	Without intervention/comparator	-
3464	2020	Sulfurous-arsenical-ferruginous balneotherapy for osteoarthritis of the hand: results from a retros	No	Without intervention/comparator	Without intervention/comparator	-
3465	2017	Evaluation of Short-Wave Diathermy and Ultrasound Treatments as Combined Physical Treatme	No	Without intervention/comparator	Without intervention/comparator	-
3466	2009	A randomized single blind crossover trial comparing leather and commercial wrist splints for trea	No	Other populations/diseases	Other populations/diseases	-
3467	2009	Comparison of the accuracy of quadriceps isometric exercise between using quadriceps educatio	No	Without intervention/comparator	Without intervention/comparator	-
3468	2017	Comparison between diary-actuated rehabilitation program and conventional physical therapy on	No	Without intervention/comparator	Without intervention/comparator	-
3469	2015	Aqua-cycling, a supporting power; preliminary results	No	Without intervention/comparator	Without intervention/comparator	-
3470	2017	Differential knee joint loading patterns during gait for individuals with tibiofemoral and patellof	No	Without intervention/comparator	Without intervention/comparator	-
3471	2002	Home based exercise programme for knee pain and knee osteoarthritis: randomised controlled tr	No	Without intervention/comparator	Without intervention/comparator	-
3472	2019	A novel, theory based intervention to promote engagement in physical activity in early rheumato	No	Without intervention/comparator	Without intervention/comparator	-
3473	2019	A Pilot Randomized Controlled Trial for Aerobic and Strengthening Exercises on Physical Funct	No	Without intervention/comparator	Without intervention/comparator	-

3474	2016	Motivational counselling and SMS-reminders for reduction of daily sitting time in patients with	No	Without intervention/comparator	Without intervention/comparator	-
3475	2017	The efficacy of motivational counselling and SMS reminders on daily sitting time in patients wit	No	Without intervention/comparator	Without intervention/comparator	-
3476	2019	Trapeziectomy with LRTI or joint replacement for CMC1 arthritis, a randomised controlled trial	No	Without intervention/comparator	Without intervention/comparator	-
3477	2017	Effectivenessofan unloading knee brace in the treatment of patients with knee osteoarthritis: a pl	No	Without intervention/comparator	Without intervention/comparator	-
3478	2017	Efficacy and safety of rebel relieverÂ® brace in patients with knee osteoarthritis e a phase iii rar	No	Without intervention/comparator	Without intervention/comparator	-
3479	2014	Evaluation of a polyacrylamide hydrogel in the treatment of induced osteoarthritis in a goat mod	No	Without intervention/comparator	Without intervention/comparator	-
3480	2010	Effectiveness of video-based home exercise for osteoarthritis of the knee: a randomized controlle	No	Without intervention/comparator	Without intervention/comparator	-
3481	2011	Effects of video-based home exercise on clinical and radiographic outcomes in adults with knee	No	Without intervention/comparator	Without intervention/comparator	-
3482	2011	The effects of electrical stimulation combined with continuous passive motion versus isometric e	No	Without intervention/comparator	Without intervention/comparator	-
3483	2019	Diverse Exercises Yesilarly Reduce Older Adults' Mobility Limitations	No	Without intervention/comparator	Without intervention/comparator	-
3484	2016	Proceedings of the 3rd IPLeiria's International Health Congress : Leiria, Portugal. 6-7 May 2016	No	other topics	other topics	-
3485	2019	The Effects of Exercise on Falls in Older People With Dementia Living in Nursing Homes: a Ra	No	Other populations/diseases	Other populations/diseases	-
3486	2009	The effect of prehabilitation exercise on strength and functioning after total knee arthroplasty	No	Other populations/diseases	Other populations/diseases	-
3487	2018	How does exercise dose affect patients with long-term osteoarthritis of the knee? A study protoco	No	Without intervention/comparator	Without intervention/comparator	-
3488	2012	Whole-body vibration decreases the proliferative response of TCD4+ cells in elderly individuals	No	Without intervention/comparator	Without intervention/comparator	-
3489	2012	Whole-body vibration decreases the proliferativeb response of TCD4(+) cells in elderly individu	No	Without intervention/comparator	Without intervention/comparator	-
3490	2010	Efficacy of a single dose transdermal flurbiprofen administration in patients with knee osteoarth	No	Without intervention/comparator	Without intervention/comparator	-
3491	2019	Impact of comorbidities on fatigue in rheumatoid arthritis patients: results from a nurse-led prog	No	Without intervention/comparator	Without intervention/comparator	-
3492	1995	Osteoporosis in rheumatoid arthritis: findings in the metacarpal, spine, and hip and a study of th	No	Without intervention/comparator	Without intervention/comparator	-
3493	2011	The FRAXÂ® based algorithm management in postmenopausal osteoporosis romanian females	No	Other populations/diseases	Other populations/diseases	-
3494	2016	The role of rehabilitation in active patients with atraumatic osteonecrosis of the femur head	No	Without intervention/comparator	Without intervention/comparator	-
3495	2016	The role of rehabilitation in avascular necrosis of the humeral head-randomized study	No	Without intervention/comparator	Without intervention/comparator	-
3496	2017	The roleofrehabilitationinsymptomatic lumbosacral facet syndrome	No	Other populations/diseases	Other populations/diseases	-
3497	2018	Home training and dorsal pain in osteoporotic females with rheumatoid arthritis: a randomized c	No	Without intervention/comparator	Without intervention/comparator	-
3498	2010	Rehabilitation and exercise tolerance Abstract	No	Other populations/diseases	Other populations/diseases	-
3499	2010	The importance of rehabilitation program in osteoarthritis patients with knee-spine syndrome	No	Without intervention/comparator	Without intervention/comparator	-
3500	2013	Chronic low back pain in osteoporotic females with rheumatoid arthritis a randomized clinical tr	No	Without intervention/comparator	Without intervention/comparator	-
3501	2015	Rehabilitation and quality of life in old females with type 2 diabetes mellitus and osteoporosis	No	Other populations/diseases	Other populations/diseases	-
3502	2015	The role of exercise spa therapy in symptomatic dish patients	No	Other populations/diseases	Other populations/diseases	-
3503	2009	Effect of whole body vibration exercise on muscle strength and proprioception in females with k	No	Without intervention/comparator	Without intervention/comparator	-
3504	2019	The effect of N-acetylcysteine on the myeloperoxidase and Tei index in patients with acute myo	No	Other populations/diseases	Other populations/diseases	-
3505	2019	The effect of oral N-acetylcysteine on galectin-3 and global longitudinal strain in patients with a	No	Other populations/diseases	Other populations/diseases	-
3506	2011	A randomized controlled trial of music-based multitask training on gait, balance and fall risk	No	Other populations/diseases	Other populations/diseases	-
3507	2013	Long-term exercise intervention in older adults: 4-year follow-up of a randomized controlled tria	No	Other populations/diseases	Other populations/diseases	-
3508	2010	Relationships between postural orientation and self reported function, hop performance and mus	No	Without intervention/comparator	Without intervention/comparator	-
3509	2007	Effects of aquatic exercise on flexibility, strength and aerobic fitness in adults with osteoarthritis	No	Without intervention/comparator	Without intervention/comparator	-
3510	2015	Effects of Off-Axis Elliptical Training on Reducing Pain and Improving Knee Function in Indivi	No	Without intervention/comparator	Without intervention/comparator	-
3511	2009	Enrolling older adults with cognitive impairment in research: lessons from a study of Tai Chi for	No	Without intervention/comparator	Without intervention/comparator	-
3512	2019	Combined Application of Electrically Stimulated Antagonist Muscle Contraction and Volitional	No	Without intervention/comparator	Without intervention/comparator	-
3513	2011	Stepping exercise improves muscle strength in the early postoperative phase after total hip arthro	No	Without intervention/comparator	Without intervention/comparator	-
3514	2016	Predictors of Exercise Adherence in Patients With Meniscal Tear and Osteoarthritis	No	Without intervention/comparator	Without intervention/comparator	-
3515	2010	Hospital-based versus home-based proprioceptive and strengthening exercise programs in knee o	No	Without intervention/comparator	Without intervention/comparator	-
3516	2010	Tramadol iontophoresis added to treatment of Knee Osteoarthritis	No	Without intervention/comparator	Without intervention/comparator	-
3517	2005	BPPV: controlled trials, contraindications, post-manoeuvre instructions, complications, imbalan	No	other topics	other topics	-

3518	2004	Effectiveness of two different physical therapy programmes in the treatment of knee osteoarthriti	No	Without intervention/comparator	Without intervention/comparator	-
3519	2012	How can we improve adherence to exercise programs in patients with osteoarthritis?: a randomiz	No	Without intervention/comparator	Without intervention/comparator	-
3520	2019	Short-term effect of occupational therapy intervention on hand function and pain in patients witl	No	Without intervention/comparator	Without intervention/comparator	-
3521	2020	Cost-utility analysis of multimodal occupational therapy in patients with thumb base osteoarthrit	No	Without intervention/comparator	Without intervention/comparator	-
3522	2019	Short-term effect of occupational therapy intervention on hand function and pain in patients witl	No	Without intervention/comparator	Without intervention/comparator	-
3523	2014	Microfracture technique versus osteochondral autologous transplantation mosaicplasty in patient	No	Without intervention/comparator	Without intervention/comparator	-
3524	2012	Therapeutic ultrasound versus sham ultrasound for the management of patients with knee osteoa	No	Without intervention/comparator	Without intervention/comparator	-
3525	2014	Rehabilitation following total hip arthroplasty evaluation over short follow-up time: randomized	No	Without intervention/comparator	Without intervention/comparator	-
3526	2016	The effects of cognitive exercise therapy approach on symptoms and antiinflammatory status in	No	Without intervention/comparator	Without intervention/comparator	-
3527	2014	Gait analysis of fixed bearing and mobile bearing total knee prostheses during walking: do mobi	No	Without intervention/comparator	Without intervention/comparator	-
3528	2018	Comparison of Efficiency Between Corticosteroid and Platelet Rich Plasma Injection Therapies	No	Without intervention/comparator	Without intervention/comparator	-
3529	2020	Effects of radial extracorporeal shock wave therapy on clinical variables and isokinetic performa	No	Without intervention/comparator	Without intervention/comparator	-
3530	2019	EFFECT OF FLAMINGO EXERCISES ON BALANCE IN PATIENTS WITH BALANCE IMP.	No	Without intervention/comparator	Without intervention/comparator	-
3531	2020	Effects of single-task versus dual-task training on balance performance in elderly patients with k	No	Without intervention/comparator	Without intervention/comparator	-
3532	2017	Preoperative Hypoalgesia After Cold Pressor Test and Aerobic Exercise is Associated With Pain	No	Without intervention/comparator	Without intervention/comparator	-
3533	2016	Acute Effects of Foot Rotation in Healthy Adults during Running on Knee Moments and Lateral	No	Without intervention/comparator	Without intervention/comparator	-
3534	2010	Effects of aquatic resistance training on mobility limitation and lower-limb impairments after kn	No	Without intervention/comparator	Without intervention/comparator	-
3535	2011	Maintenance of aquatic training-induced benefits on mobility and lower-extremity muscles amo	No	Without intervention/comparator	Without intervention/comparator	-
3536	2015	Effects of preoperative aquatic resistance training on knee pain, mobility limitation and muscle i	No	Without intervention/comparator	Without intervention/comparator	-
3537	2014	Self-reported adherence to cardiovascular risk reduction intervention of patients with rheumatoic	No	Without intervention/comparator	Without intervention/comparator	-
3538	2015	Adherence to cardiovascular prevention strategies in patients with rheumatoid arthritis	No	Without intervention/comparator	Without intervention/comparator	-
3539	2018	Effect of Early Surgery vs Physical Therapy on Knee Function Among Patients With Nonobstruc	No	Other populations/diseases	Other populations/diseases	-
3540	2013	In patients with hand OA there is no evidence that a booster session after multidisciplinary treatr	No	Without intervention/comparator	Without intervention/comparator	-
3541	2007	Expert agreement confirms that negative changes in hand and foot radiographs are a surrogate fc	No	Without intervention/comparator	Without intervention/comparator	-
3542	2010	The cost-effectiveness of exercise therapy added to general practitioners' care for hip osteoarthrit	No	Without intervention/comparator	Without intervention/comparator	-
3543	2011	Cost-effectiveness of exercise therapy added to general practitioners' care for hip osteoarthritis c	No	Without intervention/comparator	Without intervention/comparator	-
3544	2011	Cost-effectiveness of exercise therapy versus general practitioner care for osteoarthritis of the hi	No	Without intervention/comparator	Without intervention/comparator	-
3545	2010	Isolated patellofemoral osteoarthritis: a systematic review of treatment options using the GRADE	No	Review/Systematic Review/Meta-Analy:	Review/Systematic Review/Meta-Analys	-
3546	2014	Preoperative strength training for elderly patients awaiting total knee arthroplasty	No	Without intervention/comparator	Without intervention/comparator	-
3547	2012	Effect of exercise on cardiac autonomic function in females with rheumatoid arthritis	No	Without intervention/comparator	Without intervention/comparator	-
3548	2017	Strength gain and functional benefits of resistance training: dose-response relationship	No	Other populations/diseases	Other populations/diseases	-
3549	2015	Optimization of analgesics allows patients with knee osteoarthritis and severe pain to participate	No	Without intervention/comparator	Without intervention/comparator	-
3550	2015	Exercise therapy in patients with knee osteoarthritis and severe pain is enabled by optimization c	No	Without intervention/comparator	Without intervention/comparator	-
3551	2017	Feasibility and efficacy of a robotic device for hand rehabilitation in hemiplegic stroke patients:	No	Other populations/diseases	Other populations/diseases	-
3552	2017	Feasibility and efficacy of a robotic device for hand rehabilitation in hemiplegic stroke patients:	No	Other populations/diseases	Other populations/diseases	-
3553	2020	The effect of balneotherapy and peloid therapy on changes in the functional state of patients witl	No	Without intervention/comparator	Without intervention/comparator	-
3554	2020	Level of pain, muscle strength and posture: effects of PBM on an exercise program in women wi	No	Without intervention/comparator	Without intervention/comparator	-
3555	2020	Photobiomodulation via a cluster device associated with a physical exercise program in the level	No	Without intervention/comparator	Without intervention/comparator	-
3556	2011	The association between obesity and functioning of patients with osteoarthritis of hip or knee	No	Without intervention/comparator	Without intervention/comparator	-
3557	2012	The influence of sport participation on physical function in patients with osteoarthritis during an	No	Without intervention/comparator	Without intervention/comparator	-
3558	2017	The effect of two different orthoses on pain, hand function, patient satisfaction and preference in	No	Without intervention/comparator	Without intervention/comparator	-
3559	2017	Efficacy of quadriceps vastus medialis dry needling in a rehabilitation protocol after surgical rec	No	Without intervention/comparator	Without intervention/comparator	-
3560	2019	Somatosensory electrical stimulation does not improve motor coordination in patients with unila	No	Without intervention/comparator	Without intervention/comparator	-
3561	2018	Functional limitation and chronic diseases are associated with food insecurity among U.S. adults	No	Other populations/diseases	Other populations/diseases	-

3562	2011	A randomized prospective clinical study on conventional total knee arthroplasty and mini mid-va	No	Without intervention/comparator	Without intervention/comparator	-
3563	2014	Comparison of arthroplasties with or without bone tunnel creation for thumb basal joint arthritis	No	Without intervention/comparator	Without intervention/comparator	-
3564	2014	Trapeziometacarpal arthrodesis or trapeziectomy with ligament reconstruction in primary trapezi	No	Without intervention/comparator	Without intervention/comparator	-
3565	2019	A randomised pilot equivalence trial to evaluate diamagnetically enhanced transdermal delivery	No	Other populations/diseases	Other populations/diseases	-
3566	2011	Neuromuscular exercise improves functional performance in patients with severe hip osteoarthrit	No	Without intervention/comparator	Without intervention/comparator	-
3567	2012	Neuromuscular exercise (NEMEX-TJR) improves knee extension muscle power and chair stands	No	Without intervention/comparator	Without intervention/comparator	-
3568	2013	Postoperative effects of neuromuscular exercise prior to hip orknee arthroplasty - A randomised	No	Without intervention/comparator	Without intervention/comparator	-
3569	2014	Immediate efficacy of neuromuscular exercise in patients with severe osteoarthritis of the hip or	No	Without intervention/comparator	Without intervention/comparator	-
3570	2014	Postoperative effects of neuromuscular exercise prior to hip or knee arthroplasty: a randomised c	No	Without intervention/comparator	Without intervention/comparator	-
3571	2011	Hypoalgesic and motor effects of kaltenborn mobilization on elderly patients with secondary thu	No	Without intervention/comparator	Without intervention/comparator	-
3572	2012	Effect of thumb joint mobilization on pressure pain threshold in elderly patients with thumb carp	No	Without intervention/comparator	Without intervention/comparator	-
3573	2012	Radial nerve mobilization decreases pain sensitivity and improves motor performance in patients	No	Without intervention/comparator	Without intervention/comparator	-
3574	2013	Bilateral sensory effects of unilateral passive accessory mobilization in patients with thumb carp	No	Without intervention/comparator	Without intervention/comparator	-
3575	2013	The effectiveness of a manual therapy and exercise protocol in patients with thumb carpometaca	No	Without intervention/comparator	Without intervention/comparator	-
3576	2019	Thumb carpometacarpal osteoarthritis: a musculoskeletal physiotherapy perspective	No	Without intervention/comparator	Without intervention/comparator	-
3577	2010	External qigong for chronic pain	No	Other populations/diseases	Other populations/diseases	-
3578	2018	Collagen biomarker responses and pain in obese and nonobese individuals with knee OA after re	No	Without intervention/comparator	Without intervention/comparator	-
3579	2018	Comparative efficacy of eccentric and concentric-focused resistance exercise on pain, leg strengt	No	Without intervention/comparator	Without intervention/comparator	-
3580	2012	Progressive enhanced eccentric or concentric resistance exercise training for knee osteoarthritis:	No	Without intervention/comparator	Without intervention/comparator	-
3581	2013	Progressive enhanced eccentric or concentric resistance exercise training for knee osteoarthriti	No	Without intervention/comparator	Without intervention/comparator	-
3582	2019	Eccentric and Concentric Resistance Exercise Comparison for Knee Osteoarthritis	No	Without intervention/comparator	Without intervention/comparator	-
3583	2020	Concentric and Eccentric Resistance Training Comparison on Physical Function and Functional	No	Without intervention/comparator	Without intervention/comparator	-
3584	2019	Eccentric and Concentric Resistance Exercise Comparison for Knee Osteoarthritis	No	Without intervention/comparator	Without intervention/comparator	-
3585	2019	Theoryâbased diabetes selfâmanagement education with preâselection of participants: a randomi	No	Other populations/diseases	Other populations/diseases	-
3586	2014	Efficacy of a 12-month, monitored home exercise programme compared with normal care comm	No	Without intervention/comparator	Without intervention/comparator	-
3587	2014	Three months of moderate-intensity exercise reduced plasma 3-nitrotyrosine in rheumatoid arthr	No	Without intervention/comparator	Without intervention/comparator	-
3588	2015	Effects of kinesio-taping in pain and quality of life in the elderly with knee osteoarthritis-a rando	No	Without intervention/comparator	Without intervention/comparator	-
3589	2016	Kinesio Taping does not improve the symptoms or function of older people with knee osteoarthr	No	Without intervention/comparator	Without intervention/comparator	-
3590	2017	The efficacy of single high repetition quadriceps training for patients with knee osteoarthritis	No	Without intervention/comparator	Without intervention/comparator	-
3591	2013	Effects of a progressive aquatic resistance exercise program on the biochemical composition and	No	Without intervention/comparator	Without intervention/comparator	-
3592	2017	Effects of high intensity resistance aquatic training on body composition and walking speed in w	No	Without intervention/comparator	Without intervention/comparator	-
3593	2011	Pre-operative interventions (non-surgical and non-pharmacological) for patients with hip or knee	No	Without intervention/comparator	Without intervention/comparator	-
3594	2017	A walking program for people with severe knee osteoarthritis did not reduce pain but may have b	No	Without intervention/comparator	Without intervention/comparator	-
3595	2008	Neuromuscular electrical stimulation improves preoperative strength and function in total knee a	No	Without intervention/comparator	Without intervention/comparator	-
3596	2013	Exercise and self-management for people with chronic knee, hip or lower back pain: a cluster ran	No	Without intervention/comparator	Without intervention/comparator	-
3597	2014	Multiple joint osteoarthritis: patient preferences for a generic exercise and self-management prog	No	Without intervention/comparator	Without intervention/comparator	-
3598	2012	The effects of intensive diet and exercise on bone density in older adults with knee osteoarthritis	No	Without intervention/comparator	Without intervention/comparator	-
3599	2016	Xinfeng capsule for the treatment of rheumatoid arthritis patients with decreased pulmonary func	No	Without intervention/comparator	Without intervention/comparator	-
3600	2010	Influence of Tai Chi exercise on proprioception in patients with Knee Osteoarthritis: results from	No	Without intervention/comparator	Without intervention/comparator	-
3601	2010	Tai chi is useful for treating knee osteoarthritis	No	Without intervention/comparator	Without intervention/comparator	-
3602	2014	Assessing the comparative effectiveness of Tai Chi versus physical therapy for knee osteoarthriti	No	Without intervention/comparator	Without intervention/comparator	-
3603	2015	Comparative effectiveness of Tai Chi versus physical therapy in treating knee osteoarthritis: a ra	No	Without intervention/comparator	Without intervention/comparator	-
3604	2016	Comparative Effectiveness of Tai Chi Versus Physical Therapy for Knee Osteoarthritis: a Rando	No	Without intervention/comparator	Without intervention/comparator	-
3605	2004	Small needle-scalpel therapy combined with movement exercise for osteoarthritis of knee joint: a	No	Without intervention/comparator	Without intervention/comparator	-

3606	2009	Gait analysis after bi-compartmental knee replacement	No	Other populations/diseases	Other populations/diseases	-
3607	2018	Effects of exercise therapy for knee osteoarthritis	No	Without intervention/comparator	Without intervention/comparator	-
3608	2018	Construction of an adherence rating scale for exercise therapy for patients with knee osteoarthrit	No	Without intervention/comparator	Without intervention/comparator	-
3609	2019	Hip abductor strength-based exercise therapy in treating women with moderate-to-severe knee os	No	Without intervention/comparator	Without intervention/comparator	-
3610	2020	Hip abductor strengthâbased exercise therapy in treating women with moderate-to-severe knee o	No	Without intervention/comparator	Without intervention/comparator	-
3611	2013	People's perceptions and beliefs about their ability to exercise with rheumatoid arthritis	No	Without intervention/comparator	Without intervention/comparator	-
3612	2016	Effects of Whole Body Vibration Exercise associated with Quadriceps Resistance Exercise on fu	No	Without intervention/comparator	Without intervention/comparator	-
3613	2016	Effects of whole-body vibration training with quadriceps strengthening exercise on functioning a	No	Without intervention/comparator	Without intervention/comparator	-
3614	2014	Effect of the knee position during wound closure after total knee arthroplasty on early knee funct	No	Without intervention/comparator	Without intervention/comparator	-
3615	2011	Comparing the efficacy of aquatic exercises and land-based exercises for patients with knee oste	No	Without intervention/comparator	Without intervention/comparator	-
3616	2004	Aquatic exercise improves flexibility, strength, and walk time in osteoarthritis	No	Without intervention/comparator	Without intervention/comparator	-
3617	2017	Warm-needling moxibustion for knee osteoarthritis: a randomized controlled trial	No	Without intervention/comparator	Without intervention/comparator	-
3618	2020	Effects of tai chi on postural control during dual-task stair negotiation in knee osteoarthritis: a ra	No	Without intervention/comparator	Without intervention/comparator	-
3619	2013	Effects of tai chi program on neuromuscular function for patients with knee osteoarthritis: study	No	Without intervention/comparator	Without intervention/comparator	-
3620	2015	Does statin use have a disease modifying effect in symptomatic knee osteoarthritis? Study protoc	No	Without intervention/comparator	Without intervention/comparator	-
3621	2018	Application of interrupted suture at exercise position in total knee arthroplasty	No	Without intervention/comparator	Without intervention/comparator	-
3622	2012	Comparative efficacy of methyl salicylate iontophoresis and moist heat pack in the management	No	Without intervention/comparator	Without intervention/comparator	-
3623	2018	A New Mobile Application to Reduce Anxiety in Pediatric Patients Before Bone Marrow Aspira	No	Other populations/diseases	Other populations/diseases	-
3624	2011	Bicruciate-stabilised total knee replacements produce more normal sagittal plane kinematics thar	No	Other populations/diseases	Other populations/diseases	-
3625	2003	Letter to the editor (multiple letters)	No	other topics	other topics	-
3626	2018	Improved early recovery after TKA through an app-based, feedback-controlled active muscle trai	No	Without intervention/comparator	Without intervention/comparator	-
3627	2013	The John Insall award: no benefit of minimally invasive TKA on gait and strength outcomes: a ra	No	Without intervention/comparator	Without intervention/comparator	-
3628	2017	Performance Comparison of Single-Radius Versus Multiple-Curve Femoral Component in Total	No	Without intervention/comparator	Without intervention/comparator	-
3629	2009	Effects of different stretching techniques on the outcomes of isokinetic exercise in patients with	No	Without intervention/comparator	Without intervention/comparator	-
3630	2006	Physical therapy and exercise for arthritis: do they work?	No	Without intervention/comparator	Without intervention/comparator	-
3631	2009	Post-acute physiotherapy for primary total knee arthroplasty: a cochrane systematic review	No	Other populations/diseases	Other populations/diseases	-
3632	2014	ACL-RSI and KOOS Measures Predict Normal Knee Function after ACL-SPORTS Training	No	Without intervention/comparator	Without intervention/comparator	-
3633	2010	Does needling sensation (de qi) affect treatment outcome in pain? Analysis of data from a larger	No	Without intervention/comparator	Without intervention/comparator	-
3634	2019	An equine-assisted therapy intervention to improve pain, range of motion, and quality of life in a	No	Without intervention/comparator	Without intervention/comparator	-
3635	2011	Cost-effectiveness of acupuncture care as an adjunct to exercise-based physical therapy for osteo	No	Without intervention/comparator	Without intervention/comparator	-
3636	2011	The cost-effectiveness of acupuncture as an adjunct to exercise-based physiotherapy for osteoart	No	Without intervention/comparator	Without intervention/comparator	-
3637	2015	Can creatine supplementation improve body composition and objective physical function in rheu	No	Without intervention/comparator	Without intervention/comparator	-
3638	2016	Can Creatine Supplementation Improve Body Composition and Objective Physical Function in R	No	Without intervention/comparator	Without intervention/comparator	-
3639	2019	Causal mechanisms of a healthy lifestyle intervention for patients with musculoskeletal pain who	No	Without intervention/comparator	Without intervention/comparator	-
3640	2013	Sarah: strengthening and stretching for people with rheumatoid arthritis of the hands: a randomiz	No	Without intervention/comparator	Without intervention/comparator	-
3641	2015	Strengthening And stretching for Rheumatoid Arthritis of the Hand (SARAH). A randomised co	No	Without intervention/comparator	Without intervention/comparator	-
3642	2009	Activity Increase Despite Arthritis (AIDA): design of a Phase II randomised controlled trial evalu	No	Without intervention/comparator	Without intervention/comparator	-
3643	2011	Activity Increase Despite Arthritis (AÃDA): phase II randomised controlled trial of an active ma	No	Without intervention/comparator	Without intervention/comparator	-
3644	2011	Activity increase despite arthritis (AIDA): phase II randomised controlled trial of an active mana	No	Without intervention/comparator	Without intervention/comparator	-
3645	2012	Measuring illness and exercise beliefs in osteoarthritis of the hip or knee: psychometric propertie	No	Without intervention/comparator	Without intervention/comparator	-
3646	2015	Physical therapy vs. internet-based exercise training (PATH-IN) for patients with knee osteoarthr	No	Without intervention/comparator	Without intervention/comparator	-
3647	2015	Sarah: strengthening and stretching for people with rheumatoid arthritis of the hands: a randomis	No	Without intervention/comparator	Without intervention/comparator	-
3648	2017	Hand exercises for patients with rheumatoid arthritis: an extended follow-up of the SARAH ranc	No	Without intervention/comparator	Without intervention/comparator	-
3649	2018	A randomized controlled trial on maximal strength training in 60 patients undergoing total hip ar	No	Without intervention/comparator	Without intervention/comparator	-

3650	2018	A randomized controlled trial on maximal strength training in 60 patients undergoing total hip ar	No	Without intervention/comparator	Without intervention/comparator	-
3651	2010	Predictors of weight loss in overweight veterans with knee osteoarthritis who participated in a cl	No	Without intervention/comparator	Without intervention/comparator	-
3652	2009	Effect of accelerated weightbearing after matrix-associated autologous chondrocyte implantation	No	Other populations/diseases	Other populations/diseases	-
3653	2016	Impairment-targeted exercises for older adults with knee pain: a proof-of-principle study (TargE'	No	Without intervention/comparator	Without intervention/comparator	-
3654	2016	A Phase 2 Randomized Study Investigating the Efficacy and Safety of Myostatin Antibody LY24	No	Without intervention/comparator	Without intervention/comparator	-
3655	2013	Effects of resistance and Tai Ji training on mobility and symptoms in knee osteoarthritis patients	No	Without intervention/comparator	Without intervention/comparator	-
3656	2017	A randomized, blinded, comparator-controlled trial investigating a 4-week course of lyrica in sul	No	Without intervention/comparator	Without intervention/comparator	-
3657	2011	A comparison of 3 methodological approaches to defining major clinically important improveme	No	Without intervention/comparator	Without intervention/comparator	-
3658	2011	Predictors of response to physical therapy intervention in patients with primary hip osteoarthritis	No	Without intervention/comparator	Without intervention/comparator	-
3659	2017	Perioperative testosterone supplementation increases lean mass in healthy men undergoing anter	No	Without intervention/comparator	Without intervention/comparator	-
3660	2017	Moderate-to-vigorous physical activity is more important than sedentary time for improving mus	No	Without intervention/comparator	Without intervention/comparator	-
3661	2014	Elective unilateral total knee replacement using continuous femoral nerve blockade versus conve	No	Without intervention/comparator	Without intervention/comparator	-
3662	2018	Effects of Platelet-Rich Plasma on Pain and Muscle Strength in Patients With Knee Osteoarthriti	No	Without intervention/comparator	Without intervention/comparator	-
3663	2019	Telephone-Delivered Cognitive Behavioural Therapy for Treating Symptoms of Anxiety and Dep	No	Other populations/diseases	Other populations/diseases	-
3664	2011	Pain assessment: the relationship between pain thresholds and pain severity in osteoarthritis	No	Without intervention/comparator	Without intervention/comparator	-
3665	2014	Effectiveness and cost-effectiveness of a group-based outpatient physiotherapy intervention follo	No	Without intervention/comparator	Without intervention/comparator	-
3666	2014	Effectiveness and cost-effectiveness of a group-based pain self-management intervention for pat	No	Without intervention/comparator	Without intervention/comparator	-
3667	2016	Effectiveness and cost-effectiveness of outpatient physiotherapy after knee replacement for osteo	No	Without intervention/comparator	Without intervention/comparator	-
3668	2018	Foot orthoses and footwear in individuals with patellofemoral osteoarthritis: a pilot randomised t	No	Without intervention/comparator	Without intervention/comparator	-
3669	2018	EFFECT OF THERAPEUTIC EXERCISE ON KNEE OSTEOARTHRITIS AFTER INTRA-AR'	No	Without intervention/comparator	Without intervention/comparator	-
3670	2019	Day Ward Glaucoma Patients Have Lower Depression Levels and Higher Glaucoma Knowledge	No	Other populations/diseases	Other populations/diseases	-
3671	2018	Quadriceps combined with hip abductor strengthening versus quadriceps strengthening in treatir	No	Without intervention/comparator	Without intervention/comparator	-
3672	2015	A novel non-invasive adjuvant biomechanical treatment for patients with altered rehabilitation a	No	Without intervention/comparator	Without intervention/comparator	-
3673	2019	Effect of mechanical traction versus Kaltenborn traction with mobilization on post operative kne	No	Without intervention/comparator	Without intervention/comparator	-
3674	2013	Is the muscle strength improvement affected by knee osteoarthritis severity?, Diz Osteoratrit Sid	No	Without intervention/comparator	Without intervention/comparator	-
3675	2011	Effectiveness of transcutaneous electrical nerve stimulation, therapeutic exercise and the pursuit	No	Without intervention/comparator	Without intervention/comparator	-
3676	2017	Clinical effects of rehabilitation exercise in the treatment of knee osteoarthritis based on the theo	No	Without intervention/comparator	Without intervention/comparator	-
3677	2013	Rehabilitation for the management of knee osteoarthritis using comprehensive traditional Chines	No	Without intervention/comparator	Without intervention/comparator	-
3678	2018	Correlation study between proprioception and isokinetic strength of knee osteoarthritis	No	Without intervention/comparator	Without intervention/comparator	-
3679	2005	Assessment of curative effect of aerobic exercise with quality of life questionnaire for patients w	No	Without intervention/comparator	Without intervention/comparator	-
3680	2018	Vaspin alleviates myocardial ischaemia/reperfusion injury via activating autophagic flux and res	No	Other populations/diseases	Other populations/diseases	-
3681	2018	Oral Glucosamine Hydrochloride Combined With Hyaluronate Sodium Intra-Articular Injection	No	Other populations/diseases	Other populations/diseases	-
3682	2018	A prospective randomized controlled study of total knee arthroplasty via mini-subvastus and con	No	Without intervention/comparator	Without intervention/comparator	-
3683	2011	Comparison of isokinetic exercise with treatments of laser and iontophoresis for patients with os	No	Without intervention/comparator	Without intervention/comparator	-
3684	2013	Compared effects and effectiveness in applications of isokinetic exercise, laser, and diclophenac	No	Without intervention/comparator	Without intervention/comparator	-
3685	2015	Effects of progressive muscle relaxation on the quality of life of patients with rheumatoid arthriti	No	Without intervention/comparator	Without intervention/comparator	-
3686	2013	The PICO project: aquatic exercise for knee osteoarthritis in overweight and obese individuals	No	Without intervention/comparator	Without intervention/comparator	-
3687	2020	The Effects of Baduanjin Qigong on Postural Stability, Proprioception, and Symptoms of Patien	No	Without intervention/comparator	Without intervention/comparator	-
3688	2011	A comparison study of the clinical efficacy of ketoprophen phonophoresis and ultrasound therap	No	Without intervention/comparator	Without intervention/comparator	-
3689	2013	Effectiveness of ultrasound therapy in knee osteoarthritis, Diz Osteoartritli Hastalarda Ultrason T	No	Without intervention/comparator	Without intervention/comparator	-
3690	2015	The effectiveness of ultrasound treatment for the management of knee osteoarthritis: a randomize	No	Without intervention/comparator	Without intervention/comparator	-
3691	2019	Effect of game based exercise programs on pain, functional mobility and balance in patients with	No	Without intervention/comparator	Without intervention/comparator	-
3692	2018	Effectiveness of home based exercise program taught by physiotherapist on pain and function in	No	Without intervention/comparator	Without intervention/comparator	-
3693	2019	Comparison of effectiveness of the home exercise program and the home exercise program taugh	No	Without intervention/comparator	Without intervention/comparator	-

3694	2010	Efficacy of EMG-biofeedback in knee osteoarthritis	No	Without intervention/comparator	Without intervention/comparator	-
3695	2009	Efficacy of Physiotherapist after Total Knee Arthroplasty	No	Without intervention/comparator	Without intervention/comparator	-
3696	2014	Case-controll study on the synergistic effects of electroacupuncture on knee osteoarthritis after a	No	Without intervention/comparator	Without intervention/comparator	-
3697	2017	Comparison of clinical outcomes and second-look arthroscopic findings after ACL reconstructio	No	Without intervention/comparator	Without intervention/comparator	-
3698	2017	Effect of whole-body-vibration with maslinic-acid on knee strength and pain in elderly with kne	No	Without intervention/comparator	Without intervention/comparator	-
3699	2018	Evaluation of maslinic acid with whole-body vibration training in elderly women with knee oste	No	Without intervention/comparator	Without intervention/comparator	-
3700	2019	Combination effect of exercise instruction and biologics on rheumatoid arthritis patients-a rando	No	Without intervention/comparator	Without intervention/comparator	-
3701	2017	Comparison of the Effect of Sensory-Level and Conventional Motor-Level Neuromuscular Elect	No	Without intervention/comparator	Without intervention/comparator	-
3702	2017	Effects of High- and Low-Velocity Resistance Training on Gait Kinematics and Kinetics in Indiv	No	Without intervention/comparator	Without intervention/comparator	-
3703	2017	The Effect of Early Progressive Resistive Exercise Therapy on Balance Control of Patients with	No	Without intervention/comparator	Without intervention/comparator	-
3704	2015	Subjective and objective levels of physical activity and their association with cardiorespiratory f	No	Without intervention/comparator	Without intervention/comparator	-
3705	2018	Effects of Platelet-Rich Plasma on Pain and Muscle Strength in Patients With Knee Osteoarthriti	No	Without intervention/comparator	Without intervention/comparator	-
3706	2013	No difference in gait recovery after THA with different head diameters: a prospective randomize	No	Without intervention/comparator	Without intervention/comparator	-
3707	2015	An epidemiological survey on knee osteoarthritis and early ladder-like treatment in Zoucheng cit	No	Without intervention/comparator	Without intervention/comparator	-
3708	2016	Comparison of the Results of Lundborg's and Sirotakova's Resection-Suspension Arthroplasty fo	No	Without intervention/comparator	Without intervention/comparator	-
3709	2015	Comparison of the results of quantitative computed tomography and dualenergy X-ray absorptio	No	Without intervention/comparator	Without intervention/comparator	-
3710	2016	The effect of aquatic exercise on balance function, risk of fall, knee pain and quality of life in el	No	Without intervention/comparator	Without intervention/comparator	-
3711	2016	The effects of aquatic exercise in two different depths of pool on pain and biomechanical factors	No	Without intervention/comparator	Without intervention/comparator	-
3712	2012	Denosumab reduces intracortical porosity more than alendronate in the compact-appearing corte	No	Other populations/diseases	Other populations/diseases	-
3713	2015	A randomized controlled trial: preoperative home-based combined Tai Chi and Strength Training	No	Without intervention/comparator	Without intervention/comparator	-
3714	2014	Comparison between drainage and non-drainage after total hip arthroplasty in Chinese subjects	No	Without intervention/comparator	Without intervention/comparator	-
3715	2016	A prospective pilot study to evaluate an animated home-based physical exercise program as a tre	No	Without intervention/comparator	Without intervention/comparator	-
3716	2017	Community-Based Healthy Aging Interventions for Older Adults with Arthritis and Multimorbid	No	Without intervention/comparator	Without intervention/comparator	-
3717	2016	Partial versus Intact Posterior Cruciate Ligament-retaining Total Knee Arthroplasty: a Comparat	No	Without intervention/comparator	Without intervention/comparator	-
3718	2015	Clinical observation of moxibustion combined with knee joint rehabilitation for the treatment of	No	Without intervention/comparator	Without intervention/comparator	-
3719	2014	Application of transcutaneous electrical nerve stimulation to multimodal analgesia after total kn	No	Without intervention/comparator	Without intervention/comparator	-
3720	2008	The placebo effect and its determinants in osteoarthritis: meta-analysis of randomised controlled	No	Review/Systematic Review/Meta-Analys	Review/Systematic Review/Meta-Analys	-
3721	2012	Training self-administered acupressure exercise among postmenopausal women with osteoarthrit	No	Without intervention/comparator	Without intervention/comparator	-
3722	2013	Exploring the effects of self-managed acupressure on pain, function and osteoarthritis biomarker	No	Without intervention/comparator	Without intervention/comparator	-
3723	2014	Patients' values and preferences of the expected efficacy of hip arthroscopy for osteoarthritis: a p	No	Without intervention/comparator	Without intervention/comparator	-
3724	2016	A randomized control clinical study on small-needle-knife therapy combined with exercise thera	No	Without intervention/comparator	Without intervention/comparator	-
3725	2017	Comparision for clinical efficiency of continuous adductor canal block and femoral nerve block	No	Without intervention/comparator	Without intervention/comparator	-
3726	2015	Zhuifeng Tougu Capsules combined with methotrexate on the treatment of rheumatoid arthritis f	No	Without intervention/comparator	Without intervention/comparator	-
3727	2019	A Randomized Controlled Trial on the Effects of Low-Dose Extracorporeal Shockwave Therapy	No	Without intervention/comparator	Without intervention/comparator	-
3728	2018	The role of personality in patients with knee osteoarthritis	No	Without intervention/comparator	Without intervention/comparator	-
3729	2008	Acupuncture as an adjunct to exercise-based physiotherapy does not improve the pain of knee os	No	Without intervention/comparator	Without intervention/comparator	-
3730	1999	Compared observation on the effect of Sanbi Decoction combined with exercises for rheumatoid	No	Without intervention/comparator	Without intervention/comparator	-
3731	2016	Effects of Tai Ji Quan training on gait kinematics in older Chinese women with knee osteoarthri	No	Without intervention/comparator	Without intervention/comparator	-
3732	2017	Effect of Taijiquan practice versus wellness education on knee proprioception in patients with kn	No	Without intervention/comparator	Without intervention/comparator	-
3733	2017	Interactions between physical activity and risk factors of osteoarthritis on mri-detected osteophy	No	Without intervention/comparator	Without intervention/comparator	-
3734	2017	Interactions between steps per day and risk factors for osteoarthritis on MRI-detected osteophyte	No	Without intervention/comparator	Without intervention/comparator	-
3735	2017	MRI-detected osteophytes on knee: natural history and structural risk factors affecting change	No	Without intervention/comparator	Without intervention/comparator	-
3736	2019	Proprietary Milk Protein Concentrate Reduces Joint Discomfort While Improving Exercise Perfo	No	Without intervention/comparator	Without intervention/comparator	-
3737	2013	Femoral neck shortening after internal fixation of a femoral neck fracture	No	Without intervention/comparator	Without intervention/comparator	-

3738	2014	Functional outcome after successful internal fixation versus salvage arthroplasty of patients with	No	Without intervention/comparator	Without intervention/comparator	-
3739	2014	Functional outcome after successful internal fixation versus salvage arthroplasty of patients with	No	Without intervention/comparator	Without intervention/comparator	-
3740	2015	Effect of adding one 15-minute-walk on the day of surgery to fast-track rehabilitation after total	No	Without intervention/comparator	Without intervention/comparator	-
3741	2016	Mechanical loading activates vimentin, ankyrin, and vam6/vps39-like protein in primary human	No	Without intervention/comparator	Without intervention/comparator	-
3742	2017	Exploring the relationship between inflammatory markers and metabolic intermediates in older a	No	Without intervention/comparator	Without intervention/comparator	-
3743	2016	ØªØ§Ø«ÛŒØ± ØªÙ…Ø±ÛŒÙ† Ø¯Ø± Ø¢Ø¨ Ø¨Ø± Ù¾Ø§Ø³Ø® Ù‡Ø§ÛŒ Ø§ÛŒÙ†ØªØ±Ù„ÙˆÚ©ÛŒÙ† Ù¾Ø±ÙˆØªØ¦ÛŒÙ† Ù	No	other topics	other topics	-
3744	2021	Effects of Tissue Flossing on the Healthy and Impaired Musculoskeletal System: A Scoping Rev	No	Without intervention/comparator	Without intervention/comparator	-
3745	2021	Does blood flow restriction training enhance clinical outcomes in knee osteoarthritis: A systema	No	Review/Systematic Review/Meta-Analy:	Review/Systematic Review/Meta-Analys	-
3746	2021	Ventricular Tachycardia Has Mainly Non-Ischaemic Substrates in Patients with Autoimmune R	No	Without intervention/comparator	Without intervention/comparator	-
3747	2021	Guidelines for the use and interpretation of assays for monitoring autophagy (4th edition)(1).	No	Without intervention/comparator	Without intervention/comparator	-
3748	2021	The effect of the ischaemic compression technique on pain and functionality in temporomandib	No	Other populations/diseases	Other populations/diseases	-
**3749**	**2021**	**Feasibility and estimated efficacy of blood flow restricted training in female patients with**	**Yes**	**Full**	**Full**	**-**
3750	2021	Integrating blood flow restriction with low-load resistance exercise in a UK specialist military p	No	Other populations/diseases	Other populations/diseases	-
3751	2020	Benefits and harms of exercise therapy in people with multimorbidity: A systematic review and	No	Without intervention/comparator	Without intervention/comparator	-
3752	2020	Dose-response relationship between physical activity and mortality in adults with noncommunic	No	Without intervention/comparator	Without intervention/comparator	-
3753	2020	Robotic therapy for the hemiplegic shoulder pain: a pilot study.	No	Without intervention/comparator	Without intervention/comparator	-
3754	2020	Effects of preoperative low-intensity training with slow movement on early quadriceps weaknes	No	Without intervention/comparator	Without intervention/comparator	-
3755	2020	Irisin reverses intestinal epithelial barrier dysfunction during intestinal injury via binding to the	No	Without intervention/comparator	Without intervention/comparator	-
3756	2020	Comparison of Blood Flow Restriction Training versus Non-Occlusive Training in Patients with	No	Review/Systematic Review/Meta-Analy:	Review/Systematic Review/Meta-Analys	-
3757	2020	The blood flow restriction training effect in knee osteoarthritis people: a systematic review and r	No	Review/Systematic Review/Meta-Analy:	Review/Systematic Review/Meta-Analys	-
3758	2019	The Effects of Blood Flow Restriction on Muscle Activation and Hypoxia in Individuals With C	No	Other populations/diseases	Other populations/diseases	-
3759	2020	How does occupational physical activity influence health? An umbrella review of 23 health outc	No	Without intervention/comparator	Without intervention/comparator	-
3760	2020	Efficacy of low-load blood flow restricted resistance EXercise in patients with Knee osteoarthrit	No	Other populations/diseases	Other populations/diseases	-
3761	2018	Factors contributing to disability in rheumatoid arthritis patients: An Egyptian multicenter study	No	Without intervention/comparator	Without intervention/comparator	-
3762	2010	Joint torques and powers are reduced during ambulation for both limbs in patients with unilatera	No	Without intervention/comparator	Without intervention/comparator	-
3763	2009	Cardiovascular prevention guidelines in daily practice: a comparison of EUROASPIRE I, II, and	No	Without intervention/comparator	Without intervention/comparator	-
3764	2009	[Restoration of active elbow flexion by muscle transfer of the latisYesus dorsi].	No	Without intervention/comparator	Without intervention/comparator	-
3765	2009	Exercise echocardiography in rheumatoid arthritis: a case-control study	No	Without intervention/comparator	Without intervention/comparator	-
3766	2009	The joint impact of smoking and exercise capacity on clinical outcomes among women with sus	No	Other populations/diseases	Other populations/diseases	-
3767	2009	[Causes of death among pilots: "acute myocardial infarction"--are the present examination meth	No	Other populations/diseases	Other populations/diseases	-
3768	2008	The risk of myocardial infarction and pharmacologic and nonpharmacologic myocardial infarcti	No	Without intervention/comparator	Without intervention/comparator	-
3769	2008	Lower limb movement variability in patients with peripheral arterial disease.	No	Other populations/diseases	Other populations/diseases	-
3770	2008	[The factors influencing the functional state recovery in cerebral stroke patients during the secor	No	Other populations/diseases	Other populations/diseases	-
3771	2008	Cardiovascular, rheumatologic, and pharmacologic predictors of stroke in patients with rheumat	No	Without intervention/comparator	Without intervention/comparator	-
3772	2008	Physical frailty in older persons is associated with Alzheimer disease pathology.	No	Other populations/diseases	Other populations/diseases	-
3773	2007	Oxidative stress.	No	Other populations/diseases	Other populations/diseases	-
3774	2007	[Return to work with heart disease].	No	Other populations/diseases	Other populations/diseases	-
3775	2006	An unrecognized cause of recurrent hypercalcemia: immobilization.	No	Other populations/diseases	Other populations/diseases	-
3776	2006	No title [Abstract]	No	Without intervention/comparator	Without intervention/comparator	-
3777	2005	Chronic exertional compartment syndrome: diagnosis and management.	No	Without intervention/comparator	Without intervention/comparator	-
3778	2005	[Arthrodesis of interphalangeal joints by means of external frame fixation].	No	Without intervention/comparator	Without intervention/comparator	-
3779	2005	Rupture of the gastrocnemius muscle in six foals.	No	Other populations/diseases	Other populations/diseases	-
3780	2005	Rationale for testing the cardiovascular risk for patients with COX-2 inhibitors on the basis of b	No	Other populations/diseases	Other populations/diseases	-
3781	2004	Identification of relevant ICF categories in patients with chronic health conditions: a Delphi exe	No	Other populations/diseases	Other populations/diseases	-

3782	2004	Supraspinatus compartment syndrome.	No	Other populations/diseases	Other populations/diseases	-
3783	2004	Approach to the problems of the aged.	No	Without intervention/comparator	Without intervention/comparator	-
3784	2003	[Nonoperative treatment of ischemic contractures of forearm and hand].	No	Other populations/diseases	Other populations/diseases	-
3785	2002	The Institute for Ageing and Health, University of Newcastle, UK.	No	Without intervention/comparator	Without intervention/comparator	-
3786	2002	Case records of the Massachusetts General Hospital. Weekly clinicopathological exercises. Case	No	Other populations/diseases	Other populations/diseases	-
3787	2001	Determinants of locomotor disability in people aged 55 years and over: the Rotterdam Study.	No	Without intervention/comparator	Without intervention/comparator	-
3788	2000	Exercise training for claudicants: changes in blood flow, cardiorespiratory status, metabolic fun	No	Without intervention/comparator	Without intervention/comparator	-
3789	2000	Neuroendocrine regulation of IL-12 and TNF-alpha/IL-10 balance. Clinical implications.	No	Without intervention/comparator	Without intervention/comparator	-
3790	2000	[Complication of exercise therapy].	No	Without intervention/comparator	Without intervention/comparator	-
3791	2000	[Hemorheological aspects of leuko-platelet activation in atheromatous diseases: clinical applica	No	Other populations/diseases	Other populations/diseases	-
3792	2000	Blood pressure level and relation to other cardiovascular risk factors in male hypertensive patien	No	Other populations/diseases	Other populations/diseases	-
3793	1999	Heart attacks and lower-limb function in master endurance athletes.	No	Without intervention/comparator	Without intervention/comparator	-
3794	1999	A research-based use of Tai Chi/movement therapy as a nursing intervention.	No	Without intervention/comparator	Without intervention/comparator	-
3795	1998	Clinical experience of rehabilitation therapists with chronic diseases: a quantitative approach.	No	Without intervention/comparator	Without intervention/comparator	-
3796	1997	Efficacy of low load resistive muscle training in patients with rheumatoid arthritis functional cla	No	Without intervention/comparator	Without intervention/comparator	-
3797	1997	Role of interactions between psychological and clinical factors in determining 6-month mortalit	No	Other populations/diseases	Other populations/diseases	-
3798	1996	The Porous-Coated Anatomic total knee experience. Special emphasis on complications and we	No	Without intervention/comparator	Without intervention/comparator	-
3799	1996	Wrist pain in a young gymnast: unusual radiographic findings and MRI evidence of growth plat	No	Other populations/diseases	Other populations/diseases	-
3800	1996	[Definition of a diagnostic score of malignant hyperthermia using P-31 magnetic resonance spe	No	Other populations/diseases	Other populations/diseases	-
3801	1995	Exercise-induced acute renal failure associated with ibuprofen, hydrochlorothiazide, and triamte	No	Other populations/diseases	Other populations/diseases	-
3802	1995	Muscle strength, symptom intensity, and exercise capacity in patients with cardiorespiratory dis	No	Other populations/diseases	Other populations/diseases	-
3803	1994	Effect of exercise on 99mTc-DTPA clearance from knees with effusions.	No	Without intervention/comparator	Without intervention/comparator	-
3804	1994	ACE-inhibition with perindopril in essential hypertensive patients with concomitant diseases. T	No	Without intervention/comparator	Without intervention/comparator	-
3805	1994	Medical and anthropological study of a world and Olympic champion, long-distance runner, 35	No	Without intervention/comparator	Without intervention/comparator	-
3806	1994	[Hepato-digestive disorders in athletic practice].	No	Other populations/diseases	Other populations/diseases	-
3807	1993	von Willebrand factor, exercise, and ischemia/reperfusion injury.	No	Without intervention/comparator	Without intervention/comparator	-
3808	1992	[A case of adult onset phosphoglucomutase deficiency].	No	Other populations/diseases	Other populations/diseases	-
3809	1990	Exercise and arthritis. The hematology of inactivity.	No	Without intervention/comparator	Without intervention/comparator	-
3810	1988	[Exercise therapy in peripheral arterial occlusive disease in advanced age--a sensible measure?].	No	Other populations/diseases	Other populations/diseases	-
3811	1988	Finger pain.	No	Without intervention/comparator	Without intervention/comparator	-
3812	1986	Primary fibromyalgia. A clinical and laboratory study of 55 patients.	No	Without intervention/comparator	Without intervention/comparator	-
3813	1986	Guidelines for exercise testing. A report of the Joint American College of Cardiology/American	No	Other populations/diseases	Other populations/diseases	-
3814	1986	Is chronic synovitis an example of reperfusion injury?	No	Without intervention/comparator	Without intervention/comparator	-
3815	1982	[Aims and effects, favorable and unfavorable, of sports activities of adults].	No	Other populations/diseases	Other populations/diseases	-
3816	1981	[Muscle contracture induced by ischemic exercise in McArdle's disease (author's transl)].	No	Other populations/diseases	Other populations/diseases	-
3817	1980	[Complex preparation of patients with femoral stumps and concomitant ischemic heart disease f	No	Other populations/diseases	Other populations/diseases	-
3818	1980	Myoadenylate deaminase deficiency--muscle biopsy and muscle culture in a patient with gout.	No	Other populations/diseases	Other populations/diseases	-
3819	1979	Radioimmunoassays of human myoglobin in serum and urine.	No	Without intervention/comparator	Without intervention/comparator	-
3820	1978	[Rheumatic diseases following myocardial infarct].	No	Without intervention/comparator	Without intervention/comparator	-
3821	1976	Some lessons in cardiovascular epidemiology from Framingham.	No	Other populations/diseases	Other populations/diseases	-
3822	1976	[To do or not to do in geriatrics].	No	Without intervention/comparator	Without intervention/comparator	-
3823	1975	[Test for the determination of physical fitness 3 weeks after myocardial infarction].	No	Without intervention/comparator	Without intervention/comparator	-
3824	1975	Gangrene of the newborn. A case report.	No	Other populations/diseases	Other populations/diseases	-
3825	1975	The present status of the problem of pes cavus.	No	Other populations/diseases	Other populations/diseases	-

3826	1974	The medial tibial syndrome: exercise ischaemia in the medial fascial compartment of the leg.	No	Other populations/diseases	Other populations/diseases	-
3827	1973	[Ergometry in ischemic heart disease].	No	Other populations/diseases	Other populations/diseases	-
3828	1971	Case records of the Massachusetts General Hospital. Weekly clinicopathological exercises. Case	No	Other populations/diseases	Other populations/diseases	-
3829	1968	Prophylaxis of coronary heart disease, stroke, and peripheral atherosclerosis.	No	Other populations/diseases	Other populations/diseases	-
3830	1964	[KINESITHERAPY OF REFLEX DYSTROPHIC RHEUMATISM OF THE SHOULDER IN SU	No	Other populations/diseases	Other populations/diseases	-
3831	1959	Crushing injury of the hand; prevention of ischemic contracture.	No	Other populations/diseases	Other populations/diseases	-
3832	1957	March gangrene; ischaemic myositis of the leg muscle from exercise.	No	Other populations/diseases	Other populations/diseases	-

		Full-text studies assessed for eligibility	Decision (Y / N)	Remarks / reason (Reviewer #1)	Remarks / reason (Reviewer #2)	Link
161	2017	Blood flow restriction training for hand osteoarthritis: a randomised controlled trial	No	Article registration only	Article registration only	https://www.anzctr.org.au/Trial/Registration/Trial[illegible]
**458**	**2018**	**Benefits of Resistance Training with Blood Flow Restriction in Knee Osteoarthritis**	**Yes**	**Included**	**Included**	https://pubmed.ncbi.nlm.nih.gov/29266093/
**484**	**2016**	**Exercises with partial vascular occlusion in patients with knee osteoarthritis: a randomized clinical trial**	**Yes**	**Included**	**Included**	https://pubmed.ncbi.nlm.nih.gov/26971109/
486	2015	Kaatsu training to enhance physical function of older adults with knee osteoarthritis: design of a randomized controlled trial	No	Results not yet published / Studies in progress	Results not yet published / Studies in progress	https://pubmed.ncbi.nlm.nih.gov/26111922/
531	2019	Effects of blood flow restriction exercise with very low load and low volume in patients with knee osteoarthritis: Protocol for a randomized trial	No	Results not yet published / Studies in progress	Results not yet published / Studies in progress	https://pubmed.ncbi.nlm.nih.gov/30777115/
**1126**	**2019**	**Blood-flow restriction resistance exercise for older adults with knee osteoarthritis: A pilot randomized clinical trial**	**Yes**	**Included**	**Included**	https://pubmed.ncbi.nlm.nih.gov/30795545/
2289	2011	Assessment of Efficacy of Low Intensity Resistance Training in Men at Risk for Symptomatic Knee Osteoarthritis	No	Article registration only	Article registration only	https://clinicaltrials.gov/ct2/show/NCT01487525
2290	2011	Assessment of Efficacy of Low Intensity Resistance Training in Women at Risk for Symptomatic Knee Osteoarthritis	No	Article registration only	Article registration only	https://clinicaltrials.gov/ct2/show/NCT01440972
2331	2011	Vascular Occlusion and Rheumatoid Arthritis	No	Article registration only	Article registration only	https://clinicaltrials.gov/ct2/show/NCT01483157
2332	2011	Vascular Occlusion in Patients With Osteoarthritis	No	Article registration only	Article registration only	https://clinicaltrials.gov/ct2/show/NCT01483131
2782	2020	Incorporation of Photobiomodulation Therapy in an Exercise Program With Blood Flow Restriction for Knee Osteoarthritis	No	Article registration only	Article registration only	https://clinicaltrials.gov/ct2/show/NCT04247893
3056	2019	Effects of Strength Training with Reduced Blood Flow in Patients with Wear at the Knee Joint	No	Article registration only	Article registration only	https://ensaiosclinicos.gov.br/rg/RBR-3bvgx7

3082	2018	Light strength exercise associated with Partial Occlusion of blood in the treatment of knee osteoarthritis	No	Article registration only	Article registration only	https://ensaiosclinicos.gov.br/rg/RBR-6pcrfm/
**3166**	**2020**	**Low-Load Resistance Training With Blood-Flow Restriction in Relation to Muscle Function, Mass, and Functionality in Women With Rheumatoid Arthritis**	**Yes**	**Included**	**Included**	https://pubmed.ncbi.nlm.nih.gov/31033228/
3260	2015	Efficacy of Blood Flow-Restricted Low-Load Resistance Training For Quadriceps Strengthening in Men at Risk of Symptomatic Knee Osteoarthritis	No	Not diagnosed with RA or AO	Not diagnosed with RA or AO	https://pubmed.ncbi.nlm.nih.gov/26328230/
3263	2015	Efficacy of Blood Flow-Restricted, Low-Load Resistance Training in Women with Risk Factors for Symptomatic Knee Osteoarthritis	No	Not diagnosed with RA or AO	Not diagnosed with RA or AO	https://pubmed.ncbi.nlm.nih.gov/25289840/
**3749**	**2021**	**Feasibility and estimated efficacy of blood flow restricted training in female patients with rheumatoid arthritis: a randomized controlled pilot study.**	**Yes**	**Included**	**Included**	https://pubmed.ncbi.nlm.nih.gov/33300420/

S2 file. Data extraction between reviewers.
