# Supplementary material for: The effects of resistance training with blood flow restriction on muscle strength, muscle hypertrophy and functionality in patients with osteoarthritis and rheumatoid arthritis: A systematic review with meta-analysis
Source: PLoS One. 2021 Nov 10;16(11):e0259574. doi: 10.1371/journal.pone.0259574 (PMC8580240; doi:10.1371/journal.pone.0259574)
Supplement: S3 File — (PDF) [file pone.0259574.s003.pdf]

S3 file (A). Supplemental appendix: Individual analysis of methodological quality (reviewer #1) using the Physiotherapy Evidence Database (PEDro).

Studies	item 1	item 2	item 3	item 4	item 5	item 6	item 7	item 8	item 9	item 10	item 11	Sum
Rodrigues et al.(23)	-	1	0	1	0	0	1	1	1	1	1	**7**
Bryk et al.(25)	-	1	0 *	1	0	0	1	1	1	1	1	**7 ***
Ferraz et al.(24)	-	1	0	1	0	0	0	1	1	1	1	**6**
Harper et al.(22)	-	0	0	1	0	0	1	1	1	1	1	**6**
Jønsson et al. (50)	-	0	1	1	0	0	0	1	1	1	1	**6**

0= Did not score; 1= Scored; Represents the number of "points" of quality The Physiotherapy Evidence Database (PEDro). The maximum possible score was 10 points. Abbreviation: *, difference found between reviewer #1 and reviewer #2.

S3 file (B). Supplemental appendix: Individual analysis of methodological quality (reviewer #2) using the Physiotherapy Evidence Database (PEDro).

Studies	item 1	item 2	item 3	item 4	item 5	item 6	item 7	item 8	item 9	item 10	item 11	Sum
Rodrigues et al.(23)	-	1	0	1	0	0	1	1	1	1	1	**7**
Bryk et al.(25)	-	1	1 *	1	0	0	1	1	1	1	1	**8 ***
Ferraz et al.(24)	-	1	0	1	0	0	0	1	1	1	1	**6**
Harper et al.(22)	-	0	0	1	0	0	1	1	1	1	1	**6**
Jønsson et al. (50)	-	0	1	1	0	0	0	1	1	1	1	**6**

0= Did not score; 1= Scored; Represents the number of "points" of quality The Physiotherapy Evidence Database (PEDro). The maximum possible score was 10 points. Abbreviation: *, difference found between reviewer #1 and reviewer #2.
