# Supplementary material for: The effects of resistance training with blood flow restriction on muscle strength, muscle hypertrophy and functionality in patients with osteoarthritis and rheumatoid arthritis: A systematic review with meta-analysis
Source: PLoS One. 2021 Nov 10;16(11):e0259574. doi: 10.1371/journal.pone.0259574 (PMC8580240; doi:10.1371/journal.pone.0259574)
Supplement: S4 File — (PDF) [file pone.0259574.s004.pdf]

S4 file (A). Supplemental appendix: Individual analysis (Reviewer #1) for the risk of bias of the studies using the risk of bias tool 2.0 (ROB2).

First author name	Randomization process	Deviations from the intended interventions	Missing results data	The measurement result	Selection of the result reported	General trend
Rodrigues et al.(23)	High	Some concerns	Low *	Low	Low	High
Bryk et al.(25)	High	Some concerns	Low	Low	Low	High
Ferraz et al.(24)	High	Some concerns	Low	Some concerns	Low	High
Harper et al.(22)	High	Some concerns	Low	Low	Low	High
Jønsson et al.(50)	Low	Low	Low	Low	Low	Low

Abbreviation: *, difference found between reviewer #1 and reviewer #2.

S4 file (B). Supplemental appendix: Individual analysis (Reviewer #2) for the risk of bias of the studies using the risk of bias tool 2.0 (ROB2).

First author name	Randomization process	Deviations from the intended interventions	Missing results data	The measurement result	Selection of the result reported	General trend
Rodrigues et al.(23)	High	Some concerns	Some concerns *	Low	Low	High
Bryk et al.(25)	High	Some concerns	Low	Low	Low	High
Ferraz et al.(24)	High	Some concerns	Low	Some concerns	Low	High
Harper et al.(22)	High	Some concerns	Low	Low	Low	High
Jønsson et al.(50)	Low	Low	Low	Low	Low	Low

Abbreviation: *****, difference found between reviewer #1 and reviewer #2.
